# A tandem sequence motif acts as a distance-dependent enhancer in a set of genes involved in translation by binding the proteins NonO and SFPQ

**DOI:** 10.1186/1471-2164-12-624

**Published:** 2011-12-20

**Authors:** Stefan Roepcke, Silke Stahlberg, Holger Klein, Marcel H Schulz, Lars Theobald, Sabrina Gohlke, Martin Vingron, Diego J Walther

**Affiliations:** 1 Department of Computational Molecular Biology, Max Planck Institute for Molecular Genetics, Berlin, Germany; 2 Department of Human Molecular Genetics, Max Planck Institute for Molecular Genetics, Berlin, Germany; 3 Department of Biology, Chemistry, and Pharmacy, Free University Berlin, 14195 Berlin, Germany; 4 Nycomed GmbH, Konstanz, Germany

## Abstract

**Background:**

Bioinformatic analyses of expression control sequences in promoters of co-expressed or functionally related genes enable the discovery of common regulatory sequence motifs that might be involved in co-ordinated gene expression. By studying promoter sequences of the human ribosomal protein genes we recently identified a novel highly specific Localized Tandem Sequence Motif (LTSM). In this work we sought to identify additional genes and LTSM-binding proteins to elucidate potential regulatory mechanisms.

**Results:**

Genome-wide analyses allowed finding a considerable number of additional LTSM-positive genes, the products of which are involved in translation, among them, translation initiation and elongation factors, and 5S rRNA. Electromobility shift assays then showed specific signals demonstrating the binding of protein complexes to LTSM in ribosomal protein gene promoters. Pull-down assays with LTSM-containing oligonucleotides and subsequent mass spectrometric analysis identified the related multifunctional nucleotide binding proteins NonO and SFPQ in the binding complex. Functional characterization then revealed that LTSM enhances the transcriptional activity of the promoters in dependency of the distance from the transcription start site.

**Conclusions:**

Our data demonstrate the power of bioinformatic analyses for the identification of biologically relevant sequence motifs. LTSM and the here found LTSM-binding proteins NonO and SFPQ were discovered through a synergistic combination of bioinformatic and biochemical methods and are regulators of the expression of a set of genes of the translational apparatus in a distance-dependent manner.

## Background

During the human genome project it was recognized that the mere number of protein-coding genes is much smaller than expected. In contrast, entirely new molecular mechanisms have been revealed that add to the complexity of gene regulation. In general, gene expression is regulated by protein complexes that assemble on the DNA at transcription factor binding sites, which then interact with the transcriptional apparatus. With the sequencing of the human and other mammalian genomes, diverse projects were started to identify and characterize the transcribed genomic regions including their transcription start sites (TSS) and proximal regulatory sequence regions (promoter).

Bioinformatic analyses of promoter DNA sequences of groups of coexpressed or functionally related genes enable the discovery of gene regulatory mechanisms. In a prior study we investigated the well-characterized promoter sequence set of the essential and highly expressed human ribosomal protein (RP) genes and succeeded in the identification of a novel specific sequence motif [1]. The major characteristics of the motif are the tandem ATC flanks with a seven base pair (bp) spacer, its strict orientation and its localization at approximately 62 bp downstream of the TSS. Therefore, we named it LTSM, for Localized Tandem Sequence Motif. The LTSM is situated in the first intron, except for RPL12 that contains such an element in its 5'-UTR. Moreover, we found occurrences of the motif to be evolutionarily conserved between orthologous human and mouse RP promoters.

The human ribosome consists of four rRNAs and 79 proteins, which are encoded by 80 genes [2-4]. RP genes are highly and co-ordinately expressed and their TSS is rigidly controlled maintaining the 5'-terminal oligo-pyrimidine tract (5'-TOP) [4]. 5'-TOPs are mRNA sequence elements that have been intensively studied for their role in regulation of RP translation [5]. Investigations of the regulation of transcription of this large but relatively homogeneous and essential group of genes established the transcription factors YY1, GABP, hDREF, and SP1 as RP gene expression regulators [6-8].

The first main goal of the present study was the identification of further genes that might be regulated by a LTSM. By studying mouse and zebrafish RP promoters bioinformatically we first refined the definition of the LTSM sequence motif. Finally, we performed a genome-wide search for genes with LTSM-bearing promoters. Interestingly, the additional LTSM-positive genes mostly encode for products, which are involved in translation.

The second main goal of the study was the identification of proteins that bind LTSMs and the functional characterization of the motif. Here we show that the two related nucleotide binding proteins NonO (non-POU domain-containing, octamer-binding) and SFPQ (splicing factor proline/glutamine-rich) [9] bind directly to LTSM sites. Furthermore, we reveal that in this context NonO and SFPQ are not classical transcriptional regulators of RP gene expression, but rather determine the positioning of the transcriptional machinery on RP gene promoters. Therewith LTSMs and its binding proteins seem to contribute to the maintenance of the exact TSS and to the expression level in genes involved in translation.

## Results

### Refined definition of LTSM

Since evolutionary conservation of sequence features provides strong evidence for functional importance, we studied the proximal RP promoters in zebrafish [10]. Although in zebrafish the genomic background was much more AT-rich, key features of RP gene promoters, such as the TATA box and the 5'-TOP signal were comparable between fish and mammals (Additional file 1). In the RP promoters of zebrafish we identified a tandem motif that exhibits similar features to the mammalian LTSM: it consists of two ATC flanks with a seven bp spacer, was strictly oriented and located approximately 62 bp downstream of the TSS. The major difference between the mammalian and the fish motifs was the GC-content of the middle part, which was significantly higher in the mammalian motif. These observations led us to a refined definition of the LTSM: **1**. ATC-ATC tandem motif with a spacer of seven bp, **2**. located between +52 and +82 bp relative to the TSS, **3**. strictly oriented. The base distribution of the middle part is not considered functionally constrained but rather governed by the genomic background of the actual promoter. Searching the human RP promoters (-500 to +500) with the refined motif definition revealed 50 hits in total, 35 at the preferred location and in the right orientation, two at different locations but in the right orientation and 13 on the reverse-complement strand at no preferred location (Additional file 2).

We considered the distance of 10 bp from the first to the second T of the ATC-ATC tandem, which corresponds to approximately one turn of the DNA double helix, an important structural feature of the motif. Further, we speculated that LTSM binding complexes could allow some flexibility in the spacing of the two flanks. Therefore, we searched the human RP promoters for ATC-ATC tandem motifs with a spacing of one bp less and one bp more than the original motif, which contains seven bp between the ATC flanks (Additional file 2). To our surprise, we found the tripartite tandem motif (ATC-8-ATC-6-ATC) in the four genes RPS15a, RPL17, RPL3 and RPS24 in the same orientation and at the typical location relative to the TSS. Each is composed of three ATC submotifs; the first two are separated by eight base pairs and the second and third are separated by six base pairs.

### Identification of additional LTSM-positive genes

**Figure 1:**
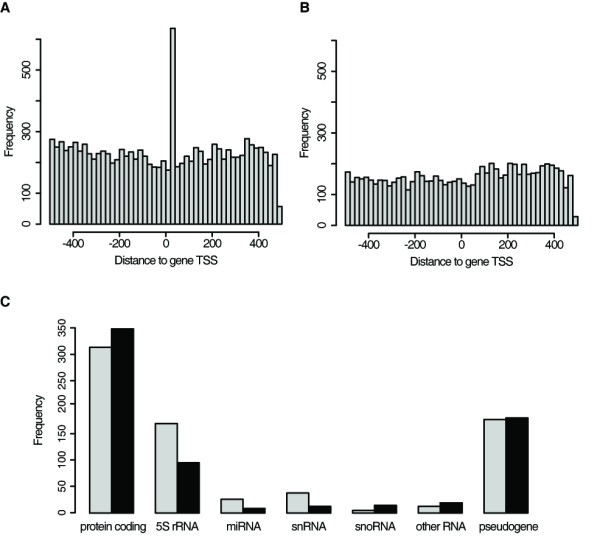
**Genome-wide analysis of LTSM**. **(A, B) **Human transcripts from EnsEMBL55 were searched for LTSM in their promoters (-500 to +500 relative to TSS) on both strands. Histograms of the LTSM - TSS distances in the promoter set (bin width: 20 bp): **(A) **without prior repeat masking and **(B) **with prior repeat masking. **(C) **Number of LTSM-positive genes (LTSM between +21 to +100 bp relative to TSS of human transcripts in EnsEMBL55) without prior repeat masking subdivided into different structural classes (grey bar: forward strand, black bar: reverse-complement strand).

We set out to screen the human genome for LTSM-positive genes to generate hypotheses about the motif's function. An important characteristic of LTSM in RP promoters was its location relative to the TSS. Therefore, we decided to scan EnsEMBL human genome database version 55 (EnsEMBL55) because it contains detailed information about transcripts including TSS and exon-intron structure [11]. The promoter regions, -500 to +500 relative to the annotated TSS, were extracted and, subsequently, the LTSM was searched on these regions in both orientations. Without repeat masking there was an enrichment between positions +21 and +40 bp (Figure 1A), although we did not find genome-wide positional enrichment in the repeat-masked promoter set (Figure 1B). Moreover, we scanned the databases DBTSS and CAGE because they were built on sequence tags from 5'-ends of capped mRNAs [12,13]. Similarly, no genome-wide positional enrichment of LTSMs was found in DBTSS or CAGE derived promoter sets after repeat masking (data not shown). We tested the TSS predictions in the gene and transcript collections of EnsEMBL55, CAGE and DBTSS based on our experimentally verified human RP promoter set (Additional file 3). Choosing the transcripts with the closest TSS from EnsEMBL55 for each RP gene yielded the best result: an average distance of seven bp to the verified TSS.

We decided to focus our search on genes where LTSMs appeared in a similar location relative to the TSS. For each transcript in EnsEMBL55 we scanned the promoter region from +21 to +100 bp relative to the TSS. This resulted in 1460 genes associated to 2078 transcripts that carry LTSM elements (Additional file 4). Note that the larger number of transcripts compared to the number of genes arose mostly through alternative transcripts that contained the same LTSM.

Interestingly, a considerable portion of LTSM-positive genes encode functional RNAs (Figure 1C). Among those were 271 out of the 429 5S ribosomal RNA genes, which are transcribed by RNA polymerase III (Pol-III). These instances were surprising in several aspects. Firstly, the distance to the TSS with about 31 bp was considerably smaller than for the typical LTSM in RNA polymerase II (Pol-II) transcribed genes (62 bp downstream of the TSS). Secondly, the motif was found in both orientations, 158 on the forward strand, 92 on the reverse-complement strand, and 21 5S-rRNA promoters contained LTSM on both strands. There were no LTSM in other rRNA genes in EnsEMBL. The second multicopy RNA gene with many LTSM-positive promoters was U2 spliceosomal RNA. 38 of 68 U2 RNA promoters contained LTSM, exclusively on the forward strand and most of them at position +37 bp. Moreover, five out of six occurrences of the tripartite LTSM elements in the RNA genes of EnsEMBL55 belonged to U2 promoters (Additional file 5).

### LTSM-positive genes encode products belonging to the translational apparatus

A Gene Ontology (GO) enrichment analysis of the LTSM-positive protein-coding genes using the Ontologizer software and the Parent-Child method revealed that only categories related to translation were enriched among LTSM-positive genes, except for three of six genes in the Purkinje cell GO category (Additional file 6)[14,15]. Apart from the above-mentioned RP genes we identified the translation initiation factors 3 subunit D and 4 Gamma 3 (EIF3D, EIF4G3) and the translation elongation factors 1-Gamma and 2 (EEF1G, EEF2) to carry LTSMs in their promoters. Mapping all genes with 5'-TOP elements of a recent study in the human genome to our list of LTSM-positive genes resulted in a minor overlap of 52 between the 1399 LTSM-positive and the 1114 TOP-positive genes including 17 RP genes [16].

### The LTSM is bound by nuclear proteins

**Figure 2:**
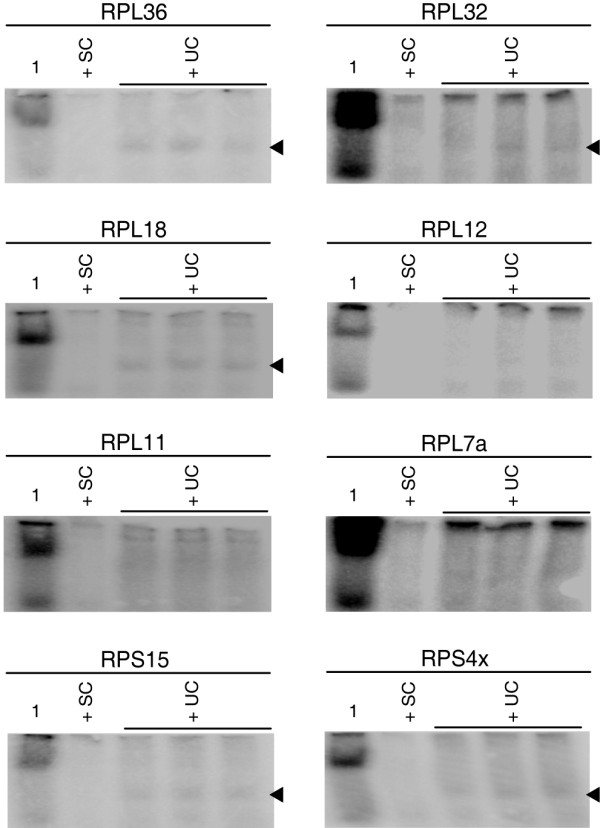
**Electromobility Shift Assays (EMSAs) with LTSM-positive probes**. EMSAs with LTSM-positive probes derived from eight different RP genes. The first lane contains nuclear extracts from HEK293 in the presence of labelled LTSM-positive probes. In the second lane unlabeled specific competitor (SC) probes were added to block specific protein binding. In the last three lanes unspecific competitor (UC) probes of LTSM-negative RPS6 was added. Black arrowheads indicate specific signals in five of the eight EMSAs.

We performed electromobility shift assays (EMSA) with nuclear extracts of SHP77, COS7 and HEK293 cells to show that LTSM are bound sequence-specifically by nuclear proteins. Radiolabelled double stranded DNA oligonucleotides harbouring the LTSM (LTSM-positive probes) derived from several RP promoters and unlabeled dsDNA competitor probes were incubated with nuclear extracts. The sequences of LTSM-negative competitor probes were chosen from the typical LTSM location of the promoters of RPS6 and RPL13a that are devoid of LTSMs. In several experiments, we tested LTSM-positive probes from the eight RP genes RPL36, RPL32, RPL18, RPL12, RPL11, RPL7a, RPS15 and RPS4X in the presence of LTSM-negative competitor probes. We found specific signals for the five genes RPL36, RPL32, RPL18, RPS15 and RPS4X using HEK293 cell nuclear extract (Figure 2, Additional file 7). When repeating the experiments with extracts from SHP77 and COS7 cells the patterns of signal intensities were comparable to those from HEK293 cells (data not shown). Although the signals of the different LTSM-positive probes were specific and of the same molecular size, the intensities varied considerably and for three probes the EMSA never showed a specific signal.

### NonO and SFPQ bind to the LTSM

The probe derived from RPL36 showed consistently the strongest specific signals in EMSA experiments. In order to identify specifically binding proteins, we generated biotinylated LTSM-positive probes from RPL36 and performed a protein pull-down from nuclear extracts in the presence of the unlabeled LTSM-negative RPS6 25mer competitor. Running a protein gel resulted in three prominent bands (Additional file 8). Repeating this experiment with the unlabeled LTSM-negative RPL13a competitor resulted in three prominent bands of similar molecular weight plus an additional band at 35 kDa. Mass spectrometric analyses of the isolated protein probes revealed three predominant proteins independent of the competitor being used: the two related nucleotide binding proteins NonO and SFPQ and the poly(ADP-ribose)-polymerase PARP1 (Additional file 9). All the other identified proteins achieved relatively low scores and occurred in only one of the two experiments. PARP1 is known to bind DNA ends in cellular DNA repair events and to stabilize numerous transcription factors at their target DNA sequences by ribosylation. Thus, we focused on NonO and SFPQ in this work, since we considered them as more likely candidates for sequence-specific LTSM binders.

**Figure 3:**
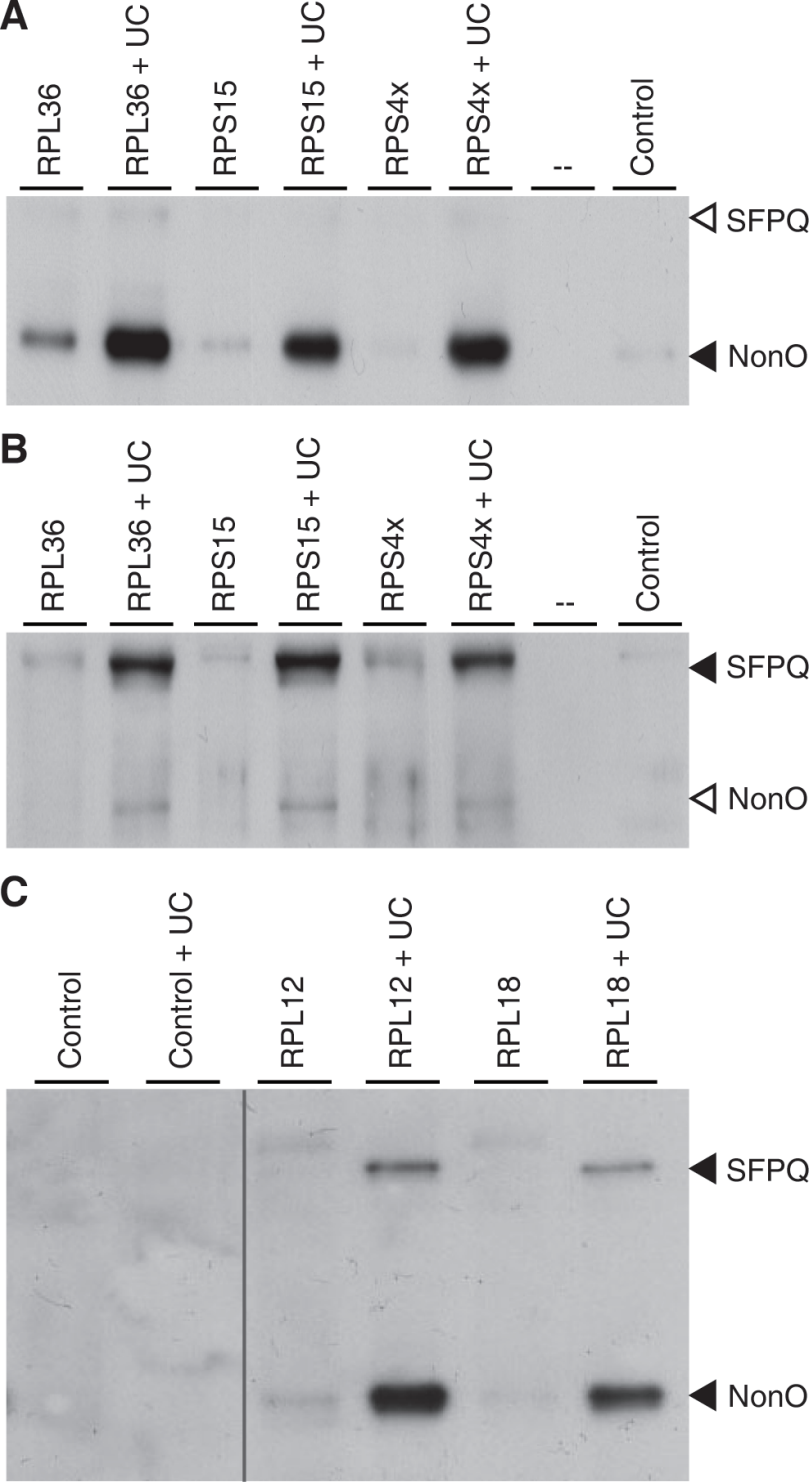
**Pull-down assays with subsequent Western blotting analysis**. Biotinylated LTSM-positive probes of five RP genes were applied to nuclear extracts of HEK293 cells (lane 1 of each probe). Unspecific competitor (UC) probes derived from LTSM-negative RPS6 were applied to block unspecific competitive protein binding that often quenches the signal of the specific binding (lane 2 of each probe). In the lanes indicated with Control the procedure was performed in the absence of LTSM-positive probes. **(A, B) **Filled arrowheads indicate specific signals for the antibodies anti-NonO (A) and anti-SFPQ (B). Open arrowheads indicate unspecific binding of anti-NonO antibody to SFPQ (A) and anti-SFPQ antibody to NonO (B). **(C) **Both specific antibodies were applied in conjunction.

We verified the binding of NonO and SFPQ by pull-down assays using biotinylated LTSM-positive dsDNA probes of the five genes RPL36, RPL18, RPL12, RPS15 and RPS4X, and subsequent Western blotting analyses with NonO and SFPQ specific antibodies (anti-NonO and anti-SFPQ). It should be noted that the LTSM-positive probes of all tested RP genes, including RPL12 and RPL18 that never showed specific signals in EMSA experiments led to the pull-down of NonO and SFPQ (Figure 3), presumably for the much higher sensitivity of Western blotting compared with EMSA. Importantly, anti-NonO also binds SFPQ and anti-SFPQ binds NonO with low affinity (Figure 3A, B). The antibodies recognize epitopes of both proteins that are located in their C-terminal homologous domains, which may explain this cross-specificity [9].

The surprising finding of the tripartite tandem motif (ATC-8-ATC-6-ATC) with the spacers of +1 and -1 in respect to the most common spacer of 7 bp motivated us to test their affinities for NonO and SFPQ binding. Pull-down assays with nuclear extracts from HEK293 cells confirmed that NonO and SFPQ bind to all three tested sequences RPS15a, RPL17 and RPS24 (Additional file 10). Other than these, the list contains one additional ATC tandem motif with an 8 bp spacer at the preferred location in RPL15 and one tri-partite tandem motif with the first spacer of 7 bp and a second one of 6 bp in RPS10. Whether these latter sequence elements are binding sites for NonO and/or SFPQ was not investigated, but it is highly probable.

**Figure 4:**
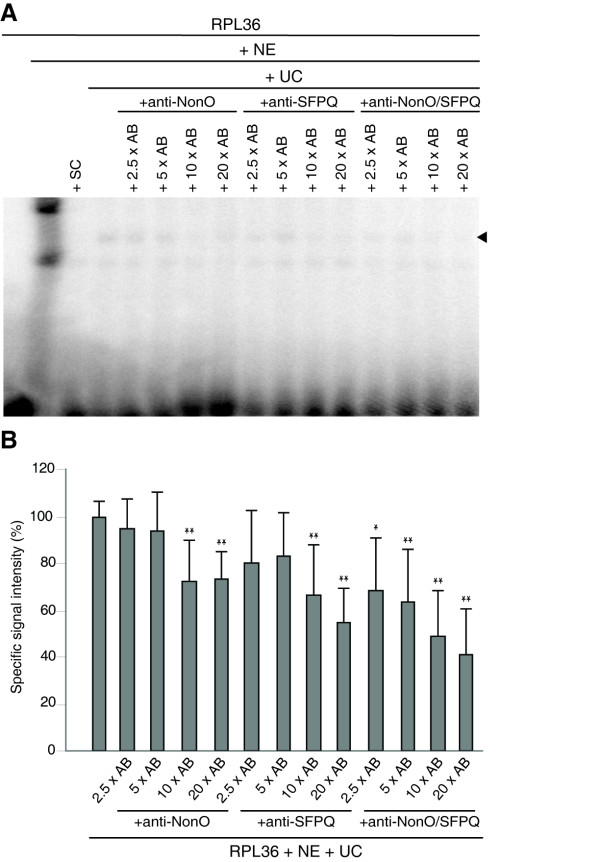
**EMSA with anti-NonO and anti-SFPQ antibodies**. **(A) **EMSA experiment using the LTSM-positive RPL36 probes and different molar excesses of specific anti-NonO and anti-SFPQ antibodies alone or in conjunction (AB). The first lane contained the labelled probe; in the following lanes nuclear extracts (NE) were added. SC indicates unlabeled specific competitor probes and UC unspecific LTSM-negative RPL13A competitor probes. Specific binding is indicated by a black arrowhead. **(B) **Quantitative analysis of the specific binding in EMSA experiments. Statistical relevance refers to the specific RPL36 signal (* p ≤ 0.1/** p ≤ 0.05 in two-sided t-test, n = 6).

Further, we characterized the binding by performing EMSA experiments with nuclear extracts of HEK293 cells incubated with RPL36 derived probes as bait, as described above, in the presence of NonO and SFPQ specific antibodies in various concentrations. We found that a gradual increase in concentration for each antibody led to a gradual decrease in the intensity of the specific signals, suggesting that the antibodies interfere with the DNA-binding ability of NonO and SFPQ (Figure 4). The effect appeared even stronger when adding both antibodies in equal molar amounts to the binding reaction. In the EMSA experiment there appeared a second lower band in each lane, which we considered to be an unspecific signal because, firstly, it was located at exactly the same position as the strong signal in the lane without competitor and, secondly, it could not be out-competed by a specific unlabeled LTSM-positive probe.

### NonO and SFPQ regulate the transcriptional activity in dependence of the LTSM-TSS distance

To elucidate the functional role of NonO and SFPQ in RP gene expression we first tested whether an over expression of NonO or SFPQ would lead to an enhanced promoter activity in dual luciferase assays. We generated expression constructs of NonO and SFPQ, and a gene expression reporter construct of the promoter of RPL12, which is the only RP gene that contains a LTSM element on its 5'-UTR and the ATG start codon on its first exon (Additional file 11). The introduction of the reporter construct into HEK293 cells without NonO or SFPQ over expression verified that the RPL12 gene promoter is highly active (> 400% of psiCHECK positive control SV40 promoter). However, the single or combined co-transfection of NonO and SFPQ expression constructs together with the RPL12 reporter construct did not lead to a further increase of the reporter signal. By titrating the reporter construct we checked that the reporter signal itself was not in the saturation region (data not shown).

**Figure 5:**
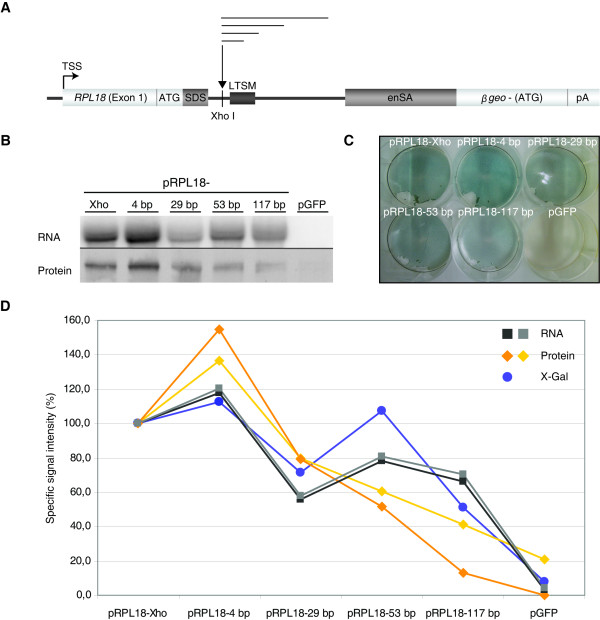
**Expression constructs with various LTSM - TSS distances**. **(A) **Schema of the vector construct pRPL18: the promoter region of RPL18 containing an internal start codon (ATG) was cloned into the gene trap vector pT1β-geo in frame with the β-geo gene lacking its start codon. An *Xho *I restriction site was added to facilitate the insertion of linker sequences of different lengths. **(B, C, D) **The cells were transfected with a GFP control construct (pGFP) and the five β-geo constructs, one with the endogenous RPL18 promoter including the *Xho *I site (pRPL18-*Xho*) and four with additional linkers of the lengths 4 bp, 29 bp, 53 bp and 117 bp. **(B) **Northern and Western blotting analyses of β-geo mRNA and protein using a β-geo specific DNA probe and an anti-β-Galactosidase antibody in the respective experiment. **(C) **One representative X-Gal staining of cells transfected with the different constructs. **(D) **Overlay of the results of three measures of expression of the construct: mRNA, protein, and enzymatic activity (see B, C). The images were scanned and the signals quantified using ImageQuant. Northern blotting signals were normalized to the rRNA fluorescence intensities of the agarose gels (not shown) and Western blotting signals by reprobing the membranes with an anti-β-actin antibody (not shown). For each experiment the signal of pRPL18-*Xho *was assigned 100% and the other signals were quantified relative to it.

Next, we tested the impact of the extension of the distance between TSS and LTSM. We selected the typical LTSM-positive gene RPL18. Exon 1 of RPL18 contains the 5'-TOP signal and the ATG start codon right before the splice site and intron 1 contains a LTSM at position +68 bp. The promoter region of RPL18 (-1000, +90 bp) was inserted into the gene trap vector pT1β-geo (Figure 5A). The introduction of an additional *Xho *I restriction site between the splice donor and the LTSM facilitated the insertion of an intronic linker sequence and thus an increase of the distance between TSS and LTSM. Linker sequences of length 29 and 117 bp led to reduced reporter signals (X-Gal staining), whereas the insertion of four bp and 53 bp produced similar or even stronger signals (Figure 5C, D). The patterns of the mRNA and protein expression of β-Galactosidase showed a slightly increased signal for the construct with the four bp fill-in and a diminished signal for the longer linker sequences. Please note that the location of the ATG start codon on the first exon ensured that the primary transcripts were correctly spliced and translated. In particular, we observed the same length for all mature mRNAs of the constructs with the various insertions (Figure 5B, Additional file 12). Furthermore, when we inhibited nonsense-mediated decay, the signals seen before in Northern blotting analyses persisted (data not shown). Thus, the gradual diminishing of the reporter signal could not be explained by nonsense-mediated decay. Finally, we also tested the inserted sequences for the presence of potential binding sites of transcriptional repressors but did not find any.

## Discussion

The comparison of human, mouse and zebrafish RP promoters affirmed the functional importance of the LTSM by the rationale of phylogenetical foot printing [10]. This analysis provided a refined definition of LTSM, consisting of ATC-flanks separated by about a full DNA turn. Studying LTSM elements in human and zebrafish promoters led us to the conclusion that the base distributions around the ATC tandem are governed by the specific genomic backgrounds of the promoter sequences. Furthermore, a detailed analysis of the base distribution in the spacer between the ATC-flanks did not yet reveal characteristic patterns that could explain the highly variable signal strength in our EMSAs experiments (data not shown). Such refined experimental permutation analyses are clearly a subject for follow-up studies that, in combination with a bioinformatic scoring, could lead to a precise prediction of the affinity of LTSM variants for NonO and SFPQ, and the respective contribution of a LTSM in regulating the level of co-ordinated expression of a LTSM-positive gene of the translational apparatus.

The genome-wide analysis did not support a global preference for location or orientation of the LTSM relative to the TSS. However, in the RP promoters of humans, mice and zebrafish LTSM elements were strictly oriented and located. And the insertion of intronic sequences between TSS and LTSM into the pT1β-geo reporter construct led to decreased expression levels. The most likely explanation for our results was that the LTSM binding complex functions as transcriptional activator or enhancer with varying strength depending on its positioning relative to the transcription initiation complex or the TSS. The distance between TSS and LTSM could be one way to fine-tune the transcription levels of RP genes. This concept would also fit our finding that the relatively small insert of four bp led to an enhanced activity of the RP promoter construct.

With regard to possible biological functions of the motif it is intriguing that although RP genes are highly and co-ordinately expressed and the sequence motif is evolutionarily conserved, less than half (35 out of 81) of the RP genes are LTSM-positive. Even a search for degenerate motifs in the RP promoters, allowing one of the six ATC-ATC base pairs to deviate, did not reveal instances that cover the entire RP gene set at the preferred position, but led to a high number of questionable matches with no preferred orientation or location instead (data not shown). In summary, gene annotation, scanning the literature, and sequence analysis provided no hint for what separates LTSM-positive and LTSM-negative RP genes [17]. For example, LTSM-positive RP genes encode parts of both the large and the small ribosomal subunits. Although there was some enrichment of proteins of the large subunit to be LTSM-positive (26 out of 47), it was not statistically significant (Fisher's exact test, p-value = 0.143).

The central discovery during this work was the identification of the related nucleotide binding proteins NonO and SFPQ as specific LTSM element binders. Although the EMSA experiments showed specific signals of widely varying strength and even no specific signal for three LTSM-positive probes, the pull-down of NonO and SFPQ with each of the tested probes establishes the specificity of our discovery. NonO is highly homologous with the C-terminal part of SFPQ, and NonO has been shown to bind RNA and single stranded DNA through its N-terminus, and double stranded DNA through its C-terminus [9]. The two proteins are parts of several complexes that are involved in a remarkable variety of nuclear processes, including regulation of transcription, splicing, nuclear retention of mRNAs, double strand break rejoining, and DNA relaxation; however, their exact function is still elusive in most cases [9,18-23]. NonO and SFPQ were reported to form homo- and heterodimers and the tandem repeat structure of the LTSM located on one side of the helix indicates that NonO and SFPQ bind DNA as dimeric complexes at LTSM elements [18]. Moreover, the discovery of the tripartite motif suggests that these proteins can even form oligomeric or at least trimeric complexes. The result of the intensity of EMSA signals being dose-dependently diminished in the presence of NonO and SFPQ antibodies could be explained by the fact that the recognized epitopes are located in the C-terminal parts of the proteins, which are considered to contain the DNA binding domain [9]. A second possible explanation could be that the antibodies impaired protein-protein interactions that are important for the DNA-binding complex to form.

In terms of transcriptional regulation, NonO and SFPQ have been studied intensively as cofactors of hormone receptors, especially androgen receptor, progesterone receptor, and steroidogenic factor 1 [19,24-26]. There are conflicting reports about the functional role of NonO in androgen receptor-dependent transcriptional regulation, be it coactivating or repressing [19,25]. Originally, NonO was identified to bind to the octamer ATTTGCAT in immunoglobulin gene promoters [27]. In addition, NonO and SFPQ regulate transcription indirectly by influencing histone modification and topoisomerase activity [9]. In particular, the two proteins were shown to mediate the binding of histone deacetylases through mSin3A to nuclear receptors and inhibit transcription [19,28].

The results of our reporter assays favour an activating or enhancing function of NonO and SFPQ on RP gene transcription by binding LTSM elements. However, the co-transfection of NonO and SFPQ expressing constructs and the RPL12 reporter construct did not lead to an increased reporter signal. A likely explanation of this result is that the extraordinarily active RPL12 promoter and the transcription factor levels are already optimized such that it is hardly possible to achieve a further increase with manipulations of any nature. Another explanation could be that the LTSM helps to recruit and orient the RNA polymerase complex, but is not a rate-limiting step in transcription. This explanation is consistent with the strong position-dependent requirements of LTSM to the TSS (as shown in Figure 5) and may explain why no further signal increase was observed following over expression of NonO and SFPQ in the reporter gene assay (Additional file 11).

The genome-wide investigation of LTSM-positive genes extended the target scope of the proposed transcriptional mechanism. Apart from RP genes, the GO analysis revealed LTSM-positive genes that encode other components of the translational machinery such as EIF3D. Most puzzling was the incidence of LTSM elements in 5S rRNA promoters. Firstly, in contrast to the RP genes, LTSMs occurred closer to the TSS at approximately +31 bp and on both strands. Secondly, in contrast to all other LTSM-positive genes 5S rRNA is transcribed by Pol-III. Classical investigations had shown that the transcription of 5S rRNA genes in *Xenopus laevis *is primarily controlled by the internal control region at +50 to +90 bps, which is essential for the determination of the correct positioning of Pol-III [29]. The presence of LTSM elements in the core promoter of more than half of the 5S rRNA genes gave rise to the hypothesis that LTSM is involved in the coordination of transcription of parts of the translational apparatus. It should be noted that we found no more LTSM elements in promoters of other rRNA genes or other Pol-III targets in EnsEMBL55.

Additionally, our genome-wide analysis revealed a number of LTSM-positive genes that encode proteins involved in transcription, splicing, mRNA processing and apparently unrelated processes. Like U2 snRNP and other splicing factors NonO and SFPQ bind the C-terminal domain of RNA Pol-II and can interact directly with the 5'-splice site [21]. If confirmed, the transcriptional regulation of splicing factors, including U2 spliceosomal RNA, by NonO and SFPQ would add another level of regulation to the well-documented direct involvement of NonO and SFPQ in splicing reactions [9,21].

## Conclusions

Bioinformatic analyses of genomic sequences are a powerful tool in predicting sequence elements with potential biological importance. However, a mere data mining with bioinformatic methods is often considered as a theoretical exercise by experimental geneticists. The here exploited synergistic application of bioinformatic and experimental techniques adds another example for the validity of *in silico *methods for the identification of players involved in the regulation of biological processes. The previously described LTSM was refined and verified experimentally in this study, and the here found LTSM-binding proteins NonO and SFPQ were only discovered as regulators of the expression of a set of genes of the translational apparatus by the predictive power of a conspicuous sequence element. The finding that these two transcription factors play a role in a TSS-LTSM distance-dependent manner opens an entry for further investigative approaches to fully disclose the role of LTSM and its binders, NonO and SFPQ, in how the translational apparatus is co-ordinately expressed.

## Methods

### Sequence set preparation and analyses

We extracted the regions of -500 to +500 bp relative to the 5'-TSS of all annotated human genes in EnsEMBL version 55 (EnsEMBL55) [11]. Apart from the raw sequence set, we generated a repeat masked sequence set using the EnsEMBL repeats. Due to the widespread existence of alternative TSSs for a given gene and the expected preferred distance of LTSM relative to the TSS, we created a transcript-based sequence set from EnsEMBL55, spanning from the TSS to +200 bp downstream.

Apart from EnsEMBL we examined two other databases CAGE and DBTSS. The Fantom 3 CAGE dataset [12] contained 663,278 CAGE tag clusters in total, a fraction of which fell into the categories BR (broad, 1517 clusters), MU (multimodal, 1277 clusters), PB (broad with sharp peak, 1185 clusters), and SP (sharp peak, 1553 clusters). The median tag cluster size was 19 bp for the complete set and 112 bp for the tag clusters from the above mentioned categories. We decided to use the most 5'-sequence tag of each cluster to define the representative TSS for promoter sequence extraction. We mapped the CAGE coordinates to human genome build hg18 using UCSC LiftOver and generated two sequence set, one containing sequences relative to all CAGE tag clusters, the second one only relative to CAGE tag clusters falling into one of the four above mentioned categories. DBTSS 6.0 [13] contained TSSs derived from full-length cDNAs in combination with massively parallel sequencing. A number of the 101,436 detected TSSs co-located at the same genomic locus. Therefore, we generated a less redundant set of TSSs by removing arbitrarily one of the overlapping TSSs, leaving 100,677 unique TSSs. We extracted genomic sequence (-500 to +500 bp) relative to the 5'-end of the annotated TSSs.

Gene Ontology information and annotation created by the consortium at 1/9/2009 was downloaded from the Gene Ontology website [30]. We used the Ontologizer 2.0 software and the Parent-Child-Union method to compute enrichments of gene ontology terms in our gene set. Subsequently, all p-values where corrected for multiple testing using the Benjamini-Hochberg procedure and values smaller than 0.05 were considered significant (same references as in the text).

### Cell culture

HEK293, COS7, and SHP77 cells were cultivated as described previously [31,32].

### Preparation of nuclear extracts

Collected cells were washed once in cold PBS. The cell pellet was resuspended in 4 times the volume of the pellet in NE buffer A (10 mM HEPES pH 7.9; 10 mM KCl; 1.5 mM MgCl_2_) and incubated on ice for 1 h. The sample was transferred to ice cold douncer and homogenized with 25 strokes. The homogenized cells were centrifuged at 2000 rpm for 5 min at 4°C and the resulting pellet was washed once in 1 ml of NE buffer A. The pellet was resuspended in 2 times its volume of NE buffer B (20 mM HEPES pH 7.9; 10% (v/v) glycerol; 420 mM NaCl; 1.5 mM MgCl_2_; 0.2 mM EDTA) and incubated on ice for 30 min. Afterwards the sample was centrifuged at 13000 rpm for 20 min at 4°C. The supernatant was transferred to a fresh 1.7 ml Eppendorf tube and an equal volume of NE buffer C (20 mM HEPES pH 7.9; 30% (v/v) glycerol; 1.5 mM MgCl_2_; 0.2 mM EDTA) was added. Nuclear extracts were aliquoted into small volumes (100-200 μl), snap freezed in dry ice and stored at -80°C.

### Electrophoretic Mobility Shift Assay

EMSAs were made using [^32^P]-dCTP-labeled double stranded DNA oligonucleotides, basically as described previously [33]. Annealing was performed by adding equal volumes of single stranded oligonucleotides (50 μM) and 4.55 mM MgCl_2 _with subsequent heating to 95°C for 2 min and successive cooling to 70°C, 65°C, 60°C, and 55°C for 30 min at a time. The labelling took place with 0.15 U of exo^- ^Klenow Fragment (NEB), 2.75 pmol [^32^P]-dCTP and 2 μmol of each remaining dNTP per 100 ng DNA for 15 min at 25°C. The labelling reaction was stopped using STE buffer (100 mM NaCl; 10 mM Tris; 1 mM EDTA; pH 7.5) in an amount equivalent to half of the reaction volume. 8 ng of labelled dsDNA oligonucleotide were mixed with 4 × binding buffer (80 mM HEPES pH 7.5; 0.04% (v/v) NP40, 20% (v/v) glycerol, 10 mM MgCl_2_), 40 mM KCl, 5-10 μg of total protein of HEK293 nuclear extracts and, if applicable, 700 fold molar excess of cold competitor probe and 2.5-20 fold molar excess of specific antibody (for antibodies see fishing experiment) in relation to the labelled oligonucleotide were added. The binding reaction took place at 37°C for 60 min. The probes were loaded into a 4% native acrylamide gel (4% (v/v) AA/0.1% (v/v) BA; 5% (v/v) glycerol; 1 x TBE) using 6 x glycerol loading dye and run with 1 × TBE at 250 V. The gel was exposed to a phosphor imager plate (Molecular Dynamics) overnight. The sequences of all DNA probes that were deployed in the EMSA experiments are presented in Additional file 13.

### Pull-down assay

200 ng of double stranded, biotinylated DNA oligonucleotides were mixed with 4 x binding buffer (80 mM HEPES pH 7.5; 0.04% (v/v) NP40; 20% (v/v) glycerol; 10 mM MgCl_2_), 40 mM KCl, 100 μg of total protein of HEK293/SHP77 nuclear extracts and, if applicable, 1000 fold molar excess of non-labelled competitor probe in relation to the labelled oligonucleotide in a final volume of 1 ml. The binding reaction took place at 37°C for 60 min. After binding the probes were given to 25 μl of streptavidin beads (Dynabeads, M-280, Dynal Biotech) and inverted at 4°C overnight. The beads were washed three times with binding buffer, whereas a vial exchange took place at every washing step, mixed with Laemmli loading dye and incubated for 5 min at 95°C. The samples were loaded into a 10-14% SDS gel. Subsequently the gel was stained with Coomassie Blue (Fermentas), the bands of interest were cut out and analyzed through MALDI-TOF. Alternatively the proteins were blotted and analyzed using specific antibodies (NonO: α-p54nrb, mouse IgG1, BD Biosciences/SFPQ: α-PSF (H-80), sc-28730, Santa Cruz). The sequences of all DNA probes that were deployed in pull-down experiments are presented in Additional file 14. For sequences of the non-labelled competitor probes see Additional file 13.

### Luciferase assay

For the luciferase assay the dual-luciferase reporter psiCHECK-2 (Promega) was used. The SV 40 promotor was replaced with the promotor sequence of RPL12 including part of exon 1 that contained the LTSM element but not the ATG start codon (- 500 bp to +88 bp relative to the TSS, primers RPL12BglII-fw, RPL12StuI-rev in Additional file 15). HEK293 cells were and transfected using Dreamfect (OZ Biosciences), 10 ng of luciferase vector and a total of 200 ng of protein expression vector were used per 96 well. If no cotransfection of NonO/SFPQ was required, one or both of the mentioned vectors were replaced by a GFP expression construct (pGFP-C1). The medium was exchanged after 24 h, lysis of cells and assay took place 48 h after transfection. The lysis and assay was performed using the Dual-Luciferase Reporter Assay System (Promega) and appropriate 96-well plates (Corning). NonO and SFPQ expression vectors were generated performing a PCR reaction from human cDNA (Sequences of used primers are presented in Additional file 15). After PCR, A-tailing and ligation into pTargeT vector (Promega) was performed.

### Generation of TSS vector

To examine whether the LTSM determines the TSS of ribosomal protein genes, the gene trap vector pT1β-geo was used. The vector construct contained a translation start site (ATG) separated from the β-geo (β-galactosidase and neomycin) fusion gene, a splicing acceptor site (enSA) and a polyadenylation signal (pA). The promoter region of RPL18 was inserted, including the TSS, the ATG, the splicing donor site and the LTSM of the gene, in detail: - 1000 bp to +90 bp (primers RPL18_HD, RPL18_XH2 in Additional file 15). Additionally an *Xho *I restriction site between the SDS and LTSM was included, through which linker oligonucleotides of different lengths could be added. First, the vector pT1β-geo was corrected with an additional nucleotide to maintain the open reading frame of the β-geo gene (GEO1-GEO4 in Additional file 15). Three oligonucleotides linkers were generated with using the primer pairs Geo-link 25-fw/rev, Geo-link 50-fw/rev and Geo-link 75-fw/rev (Additional file 15) and a fill-in of the *Xho *I site took place (T4 DNA polymerase, NEB) resulting in a relocation of the LTSM to position +4, +29, +53 and +117 related to the original position of +68 of the RPL18 LTSM. The constructs were checked by sequencing (Additional file 16).

HEK293 cells were transfected with the different pT1β-geo constructs (pRPL18) and a GFP control (pGFP-C1). After 24 h medium was exchanged and harvesting took place after 48 h. The expression of β-galactosidase was assessed using three different readouts, namely X-Gal staining, Western and Northern blot analysis. The pT1β-geo constructs comprised one vector containing the unmodified RPL18 promoter just containing the *Xho *I restriction site and the four vectors with the relocated LTSM. The plasmids were purified using CsCl purification twice before transfection.

### X-Gal staining

The growth medium was removed; the cells were washed with cold PBS and fixed on ice for 5 min with fixing solution (2% (v/v) formaldehyde; 0.8% (v/v) gluteraldehyde in PBS; pH 7.3). Afterwards the cells were washed three times with PBS and staining solution (20 mM K_3_Fe(CN)_6_; 20 mM K_4_Fe(CN)_6_; 2 mM MgCl_2_; 1 mg/ml X-Gal in PBS) was added to the cells. The samples were incubated at 37°C until colour reaction occurred (1-4 hours).

### Western blotting analysis

Cells were harvested using 1 mM PMSF in cold PBS. Cell lysis took place using RIPA buffer (50 mM Tris/HCl, pH 8.0; 150 mM NaCl; 1% (v/v) NP-40; 0.5% (v/v) deoxycholic acid; 0.1% (w/v) SDS and 1 x protease inhibitor (Complete; Roche). Proteins were blotted for 75 min at 16 V and analyzed using a specific antibody against β-Galactosidase (Promega, Catalog # Z3781).

### Northern blotting analysis

Cells were harvested using cold PBS. RNA was isolated using TRIzol Reagent (Invitrogen). Northern blotting was carried out following steps 13 to 32 of the protocol of Streit S *et al*. [34]. The 490 bp DNA probe was made by cutting the pT1β-geo vector with the restriction enzymes *Aat *II and *Eco*R I. The DNA probe matched the 5'-end of the β-geo sequence. Labeling of the probe took place with the Prime-It RmT Random Primer Labeling Kit (STRATAGENE) with subsequent purification from unincorporated nucleotides using MicroSpin G-50 Columns (Amersham Biosciences). The membrane was exposed to a phosphor imager plate overnight and scanned using a phosphorimager storm 820 (molecular dynamics).

## List of abbreviations

5'-TOP, 5': terminal oligo-pyrimidine tract; EMSA: electromobility shift assay; GO: Gene Ontology; LTSM: localized tandem sequence motif; NonO: non-POU domain-containing, octamer-binding protein; SFPQ: splicing factor proline/glutamine-rich; TSS: transcription start site

## Competing interests

The authors declare that they have no competing interests.

## Authors' contributions

SR, SS, LT, SG, and DJW carried out the molecular genetic studies, participated in the sequence alignment and drafted the manuscript. HK and MHC participated in the sequence analyses. SR, MV, and DJW participated in the design of the study and performed the statistical analysis. SR, MV, and DJW conceived of the study, and participated in its design and coordination. All authors read and approved the final manuscript.

## Supplementary Material

Additional file 1 **Supplementary Figure S1. Proximal promoters of 51 zebrafish ribosomal protein genes **Annotations of the promoter sequences (range: -80 to +100 around the TSS) with sequence motifs: LTSM (cyan underlined), CT-stretch (red box), YY1 (blue box), and TATA (orange box). Exons are marked by a grey background and the ATG translation start codon by an orange background. All sequences are aligned to their predicted TSS. Pink, yellow and green boxes mark G/T-rich, A/G-rich and A/T-rich sequence motifs of low specificity, respectively (identified automatically by motif search). Click here for file

Additional file 2 **Supplementary Table S1. LTSM hits in human RP gene promoters **The sequence set was generated by extraction of the genomic sequence from -500 to +500 relative to the TSS of each RP gene. 1 in the strand column indicates that the sequence element has the same orientation as the transcription start, -1 is the reversecomplement. The positions are given relative to the scanned sequence. The TSS is annotated at position 500. Bona fide LTSMs are marked light blue. Click here for file

Additional file 3 **Supplementary Table S2. Comparison of TSS annotations in various databases **To exclude the possibility that inaccuracies in the TSS or the transcript annotations led to the blurring of the positional enrichment of the motif we utilized our well-characterized RP promoter set. We found that for DBTSS and CAGE databases only about half of the RP genes could be correctly assigned. The EnsEMBL database contains gene annotations and transcript annotations. The gene annotations defined the 5-end of a gene as the most 5'-TSS of all its annotated transcripts. For many genes this led to the annotation of a TSS far upstream from the TSS of the most abundant transcript. For the RP gene set this resulted in an average distance between the EnsEMBL annotated TSS and the experimentally verified TSS of 18 or 21 bp depending on the version of the database. EnsEMBL55 contained on average 2.34 transcripts per RP gene. Choosing the transcripts with the closest TSS from EnsEMBL for each RP gene resulted in an average distance of seven bp relative to the TSSs of our set. Click here for file

Additional file 4 Supplementary Table S3. LTSM-positive genes from a genome-wide search Click here for file

Additional file 5 Supplementary Table S4. Tripartite LTSM-positive genes from a genome-wide search Click here for file

Additional file 6 **Supplementary Table S5. Result of the Gene Ontology (GO) enrichment analysis **The table shows the results of the Gene Ontology (GO) enrichment analysis using Otologizer. The first two columns denote the GO ID and GO name. The third column denotes the absolute number of GO terms as annotated in GO. The fourth and fifth column denote the enrichment p-value, without and with adjustments for multiple testing, respectively. Click here for file

Additional file 7 **Supplementary Figure S2. EMSA experiment with LTSM-positive probes of three genes - complete picture **The first lane of each probe contains the labelled doublestranded DNA, in the second lane nuclear extract (NE) was added. Unlabelled specific competitors (SC) were added to block specific protein binding in the third lane. In the last three lanes unspecific competitor (UC) sequence of LTSM-negative RPS6 was added. Specific binding is indicated by black arrows. Click here for file

Additional file 8 **Supplementary Figure S3. Identification of LTSM-specific binding proteins **Pull-down experiment using biotinylated LTSM-positive probes of RPL36, nuclear extract (NE) of SHP77 cells and 1000-fold molar excess of unspecific competitor (UC) sequence of LTSM-negative RPS6. The three dominant bands detected by Coomassie staining (indicated by black arrows) were analyzed using mass spectrometry (n = 2). For identified proteins compare **Additional file **9. Click here for file

Additional file 9 **Supplementary Table S6. List of proteins in LTSM pull-down samples **Mass spectrometry (MALDI-TOF) analysis of proteins pulled by biotinylated LTSM-positive probes of RPL36 (compare **Additional file **8) and two different unspecific, LTSM-negative competitors (RPS6 and RPL13A). The unspecific competitors (UC) were of the same position as LTSM. H.s: Homo sapiens. Click here for file

Additional file 10 **Supplementary Figure S6. NonO and SFPQ bind to tripartite LTSMs **Pull-down assays with subsequent Western blotting analysis using biotinylated DNA oligonucleotides containing the tripartite LTSMs of the three RP genes RPS15A, RPL17 and RPL24. Unspecific competitor (UC) sequence of LTSM-negative derived from RPS6 was applied in 1000-fold molecular excess. Non-biotinylated LTSM-positive RPL36 probes were used as control. Specific protein signals are indicated by black arrowheads. Proteins were detected using both specific antibodies in conjunction. Click here for file

Additional file 11 **Supplementary Figure S4. Influence of NonO and SFPQ on RPL12 promotor activity using luciferase assays **(A) Schematic view of a part of the human RPL12 gene indicating the amplified region. (B,C) Dual Luciferase vector psiCHECK2 with the original SV40 promotor and vector psiRPL12 with the original promotor replaced by the proximal promoter region and part of exon 1 of RPL12. (D) Cotransfection of psiRPL12 and eukaryotic expression vectors containing the genes of NonO and SFPQ (pTarget-NonO and pTarget-SFPQ) did not result in a significantly enhanced promoter activity. GFP expression vector alone served as a control. Click here for file

Additional file 12 **Supplementary Figure S5. Distance of LTSM to TSS determines the efficiency of transcription **Complete picture of Northern blotting experiment detecting β- Galactosidase transcripts via β-Galactosidase specific DNA probe at 5'-end of the transcript (Refer to Figure 5B). Click here for file

Additional file 13 **Supplementary Table S7. Sequences of single stranded DNA oligonucleotides used in EMSA experiments **The LTSMs are highlighted in grey on the forward strand. Click here for file

Additional file 14 **Supplementary Table S8. Sequences of biotinylated DNA oligonucleotides used in pull down experiments **The LTSMs are highlighted in grey on the forward strand. Click here for file

Additional file 15 Supplementary Table S9. Sequences of employed PCR primers and DNA linker oligonucleotides Click here for file

Additional file 16 **Supplementary Figure S7. Sequence verification of inserted linkers **Sequences obtained by sequencing the described RPL18 region in pGEM-T Easy vector using primer M13 rev (-49). Please, refer to Figure 5 in the main text. Translation start (ATG) is shown in bold letters, inserted *Xho *I restriction site with insertion of linkers of different lengths (bold letters) is highlighted in dark grey and the LTSM is highlighted in light grey. Click here for file
