# Supplementary material for: A tandem sequence motif acts as a distance-dependent enhancer in a set of genes involved in translation by binding the proteins NonO and SFPQ
Source: BMC Genomics. 2011 Dec 20;12:624. doi: 10.1186/1471-2164-12-624 (PMC3262029; doi:10.1186/1471-2164-12-624)
Supplement: Additional file 2 — Supplementary Table S1. LTSM hits in human RP gene promoters The sequence set was generated by extraction of the genomic sequence from -500 to +500 relative to the TSS of each RP gene. 1 in the strand column indicates that the sequence element has the same orientation as the transcription start, -1 is the reversecomplement. The positions are given relative to the scanned sequence. The TSS is annotated at position 500. Bona fide LTSMs are marked light blue. [file 1471-2164-12-624-S2.PDF]

Additional file 2 – Supplementary Table 1. LTSM hits in human RP gene promoters

The sequence set was generated by extraction of the genomic sequence from -500 to +500 relative to the TSS of each RP gene. 1 in the strand column indicates that the sequence element has the same orientation as the transcription start, -1 is the reversecomplement. The positions are given relative to the scanned sequence. The TSS is annotated at position 500. Bona fide LTSMs are marked light blue.

Standard LTSM element: ATC- 7 bp - ATC

Gene	Strand	Sequence element	Position
RPLP1	1	ATCCTCCTACATC	68
RPS17	1	ATCCTCCACAATC	552
RPL12	1	ATCCGGGTTCATC	555
RPS4Y	1	ATCTGCCTCCATC	556
RPL37	1	ATCGCGTTCCATC	556
RPL36	1	ATCCGCCGCCATC	557
RPS14	1	ATCGGGGGGCATC	558
RPS15	1	ATCCGGCTCCATC	558
RPL10A	1	ATCCGCGTTCATC	558
RPL32	1	ATCCGTGGCAATC	558
RPS4X	1	ATCCGCCTCCATC	559
RPL29	1	ATCCGATGCCATC	559
RPL8	1	ATCCGCCGCCATC	560
RPL9	1	ATCTGAAACCATC	560
RPL23	1	ATCCTCCACCATC	560
RPL31	1	ATCCAGCCCCATC	560
RPL11	1	ATCCGTCGCCATC	561
RPL27	1	ATCCGCTGCCATC	563
RPS10	1	ATCGGCTCCCATC	564
RPL7	1	ATCCTTCTCCATC	564
RPL13	1	ATCCAGCGCCATC	564
RPL6	1	ATCCTTCGCCATC	565
RPL26	1	ATCAGCAGCCATC	565
RPL36A	1	ATCCAGCTTAATC	565
RPL35	1	ATCGGGCGCCATC	566
RPS2	1	ATCCGCGGACATC	567
RPL34	1	ATCGGCTGCCATC	567
RPL41	1	ATCCATAGACATC	567
RPS18	1	ATCCAGCGGCATC	569
RPL18	1	ATCCGCTGCCATC	569
RPL24	1	ATCCGCTTACATC	570
RPL7A	1	ATCCGCTGCCATC	571
RPL37A	1	ATCTGCCTGCATC	571
RPL38	1	ATCTGGGGCAATC	571
RPS8	1	ATCAGGCCCCATC	573
RPL39	1	ATCGGCCCCCATC	578
RPL34	1	ATCTTTGGAAATC	684
RPL26	-1	ATCAACGTTCATC	9
RPS12	-1	ATCAGTTAAAATC	119
RPL23	-1	ATCTCGCGGTATC	233
RPS6	-1	ATCGCCTGCCATC	272
RPL4	-1	ATCGCCGCACATC	282
RPS3	-1	ATCCACCGCCATC	289
RPL5	-1	ATCTAACGCCATC	310

RPS15	-1	ATCTTGCCGGATC	479
RPL35A	-1	ATCCTCGGAAATC	589
RPL5	-1	ATCTCACAGGATC	658
RPS13	-1	ATCCAGCCTCATC	664
RPS3	-1	ATCTGCTGAAATC	829
RPL27	-1	ATCAGAACTTATC	953

Standard LTSM element: ATC- 8 bp - ATC

Gene	Strand	Sequence element	Position
RPL19	1	ATCTTTTAGTGATC	0
RPS8	1	ATCAAGTGACAATC	72
RPL28	1	ATCATATGCCAATC	170
RPL22	1	ATCGCTCCCCTATC	369
**RPS15A**	1	ATCCGTCTGCCATC	556
**RPL15**	1	ATCCGCCTTTGATC	556
**RPL17**	1	ATCCTCCTGCCATC	556
**RPS24**	1	ATCTGCCGCGTATC	568
**RPL3**	1	ATCCGCGACGCATC	582
RPL36A	1	ATCTTGCCGGGATC	656
RPS30	1	ATCCCGTCGCGATC	713
RPL27	1	ATCGTGAAGGTATC	885
RPL35A	-1	ATCAGCCCAACATC	306
RPS3A	-1	ATCCGACTGGAATC	308
RPL5	-1	ATCCACTCACCATC	412
RPSA	-1	ATCCTGGTCGGATC	671
RPL34	-1	ATCTATTGGAAATC	713
RPL19	-1	ATCACGTGGTTATC	757

Standard LTSM element: ATC- 6 bp - ATC

Gene	Strand	Sequence element	Position
RPS8	1	ATCCGATTGATC	63
RPS15A	1	ATCAAGAACATC	64
RPS8	1	ATCTTAGGAATC	83
RPS13	1	ATCTTTGTGATC	220
RPS13	1	ATCGCCGCCATC	514
**RPS15A**	1	ATCGGCGCCATC	567
**RPL17**	1	ATCGCCGCCATC	567
**RPS10**	1	ATCCGGGCTATC	574
**RPS24**	1	ATCCGAGCCATC	579
RPL3	1	ATCGCCGCCATC	593
RPS30	1	ATCTCCTTTATC	753
RPLP2	1	ATCAAGAAGATC	834
RPS27	1	ATCTCCTTCATC	862
RPL8	1	ATCAAGGGCATC	881
RPS17	1	ATCGCCATTATC	928
RPS12	-1	ATCAGAACCATC	110
RPL35A	-1	ATCTCCTCCATC	317
RPS3A	-1	ATCCTCGCCATC	319
RPL38	-1	ATCGGCCCCATC	337
RPS7	-1	ATCGGCTGTATC	359
RPL6	-1	ATCTGGGGCATC	564
RPL36	-1	ATCGACGACATC	769
RPL6	-1	ATCACTTGAATC	818

RPS16	-1	ATCCCCCGCATC	889
RPS4X	-1	ATCGAGACCATC	911
RPL3	-1	ATCTCGGGCATC	932
