# Supplementary material for: A tandem sequence motif acts as a distance-dependent enhancer in a set of genes involved in translation by binding the proteins NonO and SFPQ
Source: BMC Genomics. 2011 Dec 20;12:624. doi: 10.1186/1471-2164-12-624 (PMC3262029; doi:10.1186/1471-2164-12-624)
Supplement: Additional file 3 — Supplementary Table S2. Comparison of TSS annotations in various databases To exclude the possibility that inaccuracies in the TSS or the transcript annotations led to the blurring of the positional enrichment of the motif we utilized our well-characterized RP promoter set. We found that for DBTSS and CAGE databases only about half of the RP genes could be correctly assigned. The EnsEMBL database contains gene annotations and transcript annotations. The gene annotations defined the 5-end of a gene as the most 5'-TSS of all its annotated transcripts. For many genes this led to the annotation of a TSS far upstream from the TSS of the most abundant transcript. For the RP gene set this resulted in an average distance between the EnsEMBL annotated TSS and the experimentally verified TSS of 18 or 21 bp depending on the version of the database. EnsEMBL55 contained on average 2.34 transcripts per RP gene. Choosing the transcripts with the closest TSS from EnsEMBL for each RP gene resulted in an average distance of seven bp relative to the TSSs of our set. [file 1471-2164-12-624-S3.PDF]

**Additional file 3 – Supplementary Table 2. Comparison of TSS annotations in various databases**

To exclude the possibility that inaccuracies in the TSS or the transcript annotations led to the blurring of the positional enrichment of the motif we utilized our wellcharacterized RP promoter set. We found that for DBTSS and CAGE databases only about half of the RP genes could be correctly assigned. The EnsEMBL database contains gene annotations and transcript annotations. The gene annotations defined the 5-end of a gene as the most 5'-TSS of all its annotated transcripts. For many genes this led to the annotation of a TSS far upstream from the TSS of the most abundant transcript. For the RP gene set this resulted in an average distance between the EnsEMBL annotated TSS and the experimentally verified TSS of 18 or 21 bp depending on the version of the database. EnsEMBL55 contained on average 2.34 transcripts per RP gene. Choosing the transcripts with the closest TSS from EnsEMBL for each RP gene resulted in an average distance of seven bp relative to the TSSs of our set.

| Database | No. genes or transcripts | No. TSS for the 80 RPs | No. missing (or \|dist\| >100) | Mean distance |
|---|---|---|---|---|
| DBTSS all | 101,214 | 68 | 36 | 2 |
| DBTSS Unique | 100,677 | 54 | 36 | 2 |
| CAGE all | 663,278 | 103 | 22 | -9 |
| CAGE clustered | 5,536 | 28 | 52 | -28 |
| ENS46 genes | 25,202 | 63 | 17 | -18 |
| ENS46 transcripts | 51,142 | 97 | 11 | -13 |
| ENS55 genes | 29,591 | 70 | 11 | -21 |
| ENS55 transcripts | 96,145 | 179 | 5 | -7 |
