# Supplementary material for: A tandem sequence motif acts as a distance-dependent enhancer in a set of genes involved in translation by binding the proteins NonO and SFPQ
Source: BMC Genomics. 2011 Dec 20;12:624. doi: 10.1186/1471-2164-12-624 (PMC3262029; doi:10.1186/1471-2164-12-624)
Supplement: Additional file 4 — Supplementary Table S3. LTSM-positive genes from a genome-wide search [file 1471-2164-12-624-S4.PDF]

Additional file 4 – Supplementary Table 3. LTSM-positive genes from a genome-wide search

ENSEMBL55 GeneID	Gene Symbol	ENSEMBL55 TranscriptID	Biotype	Gene start	Gene end	Strand	Motif start	TSS offset	Motif strand	Description
ENSG00000211592	IGKC	ENST00000390237	Immunoglobulin	89156997	89157196	-	89157168	28	-	Immunoglobulin Kappa light chain C gene segment [Source:IMGT/GENE-DB;Acc:IGKC]
ENSG00000211960	AB019438.1-6	ENST00000390620	Immunoglobulin	106926445	1,07E+08	-	1,07E+08	68	+	Immunoglobulin heavy chain V gene segment [Source:IMGT/GENE-DB;Acc:IGHV3-43]
ENSG00000211946	AB019440.1-8	ENST00000390606	Immunoglobulin	106667834	1,07E+08	-	1,07E+08	68	+	Immunoglobulin heavy chain V gene segment [Source:IMGT/GENE-DB;Acc:IGHV3-20]
ENSG00000211874	AE000662.1-36	ENST00000390522	Immunoglobulin	22998580	22998779	+	22998652	72	-	T-cell receptor alpha J gene segment [Source:IMGT/GENE-DB;Acc:TRAJ15]
ENSG00000211680	D87023.1-5	ENST00000390326	Immunoglobulin	23252740	23252939	+	23252767	27	-	Immunoglobulin Lambda light chain J gene segment [Source:IMGT/GENE-DB;Acc:IGLJ4]
ENSG00000211622	AC233264.2-15	ENST00000390267	Immunoglobulin	89986352	89986551	+	89986406	54	-	Immunoglobulin Kappa light chain V gene segment [Source:IMGT/GENE-DB;Acc:IGKV2D-29]
ENSG00000211829	AE000661.1-17	ENST00000390477	Immunoglobulin	22931924	22932123	+	22932023	99	+	T-cell receptor delta C gene segment [Source:IMGT/GENE-DB;Acc:TRDC]
ENSG00000239109	AL121897.32-2	ENST00000459151	miRNA	30875837	30876036	-	30876005	31	-	
ENSG00000221091	hsa-mir-1302-5	ENST00000408164	miRNA	49231123	49231322	-	49231248	74	-	hsa-mir-1302-5 [Source:miRBase;Acc:MI0006366]
ENSG00000221170	hsa-mir-1304	ENST00000408243	miRNA	93466731	93466930	-	93466877	53	-	hsa-mir-1304 [Source:miRBase;Acc:MI0006371]
ENSG00000211520	hsa-mir-216b	ENST00000390186	miRNA	56227731	56227930	-	56227837	93	-	hsa-mir-216b [Source:miRBase;Acc:MI0005569]
ENSG00000207548	hsa-mir-217	ENST00000384817	miRNA	56210012	56210211	-	56210137	74	-	hsa-mir-217 [Source:miRBase;Acc:MI0000293]
ENSG00000216001	hsa-mir-450b	ENST00000401182	miRNA	133674093	1,34E+08	-	1,34E+08	64	-	hsa-mir-450b [Source:miRBase;Acc:MI0005531]
ENSG00000207933	hsa-mir-9-1	ENST00000385198	miRNA	156390022	1,56E+08	-	1,56E+08	26	-	hsa-mir-9-1 [Source:miRBase;Acc:MI0000466]
ENSG00000207570	hsa-mir-9-2	ENST00000384838	miRNA	87962558	87962757	-	87962731	26	-	hsa-mir-9-2 [Source:miRBase;Acc:MI0000467]
ENSG00000221690	AC004875.2	ENST00000408763	miRNA	121214609	1,21E+08	-	1,21E+08	92	+	
ENSG00000221530	AC005037.2-1	ENST00000408603	miRNA	201707823	2,02E+08	-	2,02E+08	47	+	
ENSG00000221631	AP000897.6	ENST00000408704	miRNA	7738024	7738223	-	7738175	48	+	
ENSG00000207692	hsa-mir-592	ENST00000384959	miRNA	126698039	1,27E+08	-	1,27E+08	44	+	hsa-mir-592 [Source:miRBase;Acc:MI0003604]
ENSG00000211571	AC010525.6-2	ENST00000390145	miRNA	56196138	56196337	+	56196197	59	-	
ENSG00000222759	AC068135.5	ENST00000410827	miRNA	194855777	1,95E+08	+	1,95E+08	89	-	
ENSG00000220985	AC092966.6	ENST00000408058	miRNA	192881351	1,93E+08	+	1,93E+08	96	-	
ENSG00000207608	hsa-mir-127	ENST00000384876	miRNA	101349316	1,01E+08	+	1,01E+08	41	-	hsa-mir-127 [Source:miRBase;Acc:MI0000472]
ENSG00000221379	AC004460.1-3	ENST00000408452	miRNA	31093509	31093708	+	31093599	90	+	
ENSG00000221374	AC007242.3-1	ENST00000408447	miRNA	202705852	2,03E+08	+	2,03E+08	71	+	
ENSG00000221593	AC026150.7-5	ENST00000408666	miRNA	30854013	30854212	+	30854103	90	+	

ENSEMBL55 GeneID	Gene Symbol	ENSEMBL55 TranscriptID	Biotype	Gene start	Gene end	Strand	Motif start	TSS offset	Motif strand	Description
ENSG00000221358	AC026150.7-6	ENST00000408431	miRNA	30906074	30906273	+	30906164	90	+	
ENSG00000239124	AC091565.10-2	ENST00000459619	miRNA	23264866	23265065	+	23264956	90	+	
ENSG00000212095	AC096922.2	ENST00000390806	miRNA	68684067	68684266	+	68684131	64	+	
ENSG00000238737	AC100756.5-5	ENST00000459602	miRNA	23609591	23609790	+	23609681	90	+	
ENSG00000221250	AC120045.19-6	ENST00000408323	miRNA	30385012	30385211	+	30385102	90	+	
ENSG00000221785	AC120045.19-7	ENST00000408858	miRNA	30437080	30437279	+	30437170	90	+	
ENSG00000221405	AC123768.8-3	ENST00000408478	miRNA	32895488	32895687	+	32895578	90	+	
ENSG00000221473	AL159152.11	ENST00000408546	miRNA	93142401	93142600	+	93142483	82	+	
ENSG00000221017	hsa-mir-1323	ENST00000408090	miRNA	54175222	54175421	+	54175307	85	+	hsa-mir-1323 [Source:miRBase;Acc:MI0003786]
ENSG00000207761	hsa-mir-329-1	ENST00000385028	miRNA	101493122	1,01E+08	+	1,01E+08	77	+	hsa-mir-329-1 [Source:miRBase;Acc:MI0001725]
ENSG00000207762	hsa-mir-329-2	ENST00000385029	miRNA	101493437	1,01E+08	+	1,01E+08	79	+	hsa-mir-329-2 [Source:miRBase;Acc:MI0001726]
ENSG00000199143	hsa-mir-373	ENST00000362273	miRNA	54291959	54292158	+	54292042	83	+	hsa-mir-373 [Source:miRBase;Acc:MI0000781]
ENSG00000200127	Y_RNA	ENST00000363257	misc_RNA	41149825	41150024	-	41149989	35	-	Y RNA [Source:RFAM;Acc:RF00019]
ENSG00000200171	Y_RNA	ENST00000363301	misc_RNA	44618953	44619152	-	44619117	35	-	Y RNA [Source:RFAM;Acc:RF00019]
ENSG00000200344	Y_RNA	ENST00000363474	misc_RNA	8042844	8043043	-	8043009	34	-	Y RNA [Source:RFAM;Acc:RF00019]
ENSG00000201371	Y_RNA	ENST00000364501	misc_RNA	27733514	27733713	-	27733678	35	-	Y RNA [Source:RFAM;Acc:RF00019]
ENSG00000201724	Y_RNA	ENST00000364854	misc_RNA	100221883	1E+08	-	1E+08	35	-	Y RNA [Source:RFAM;Acc:RF00019]
ENSG00000207155	Y_RNA	ENST00000384426	misc_RNA	141621897	1,42E+08	-	1,42E+08	35	-	Y RNA [Source:RFAM;Acc:RF00019]
ENSG00000207213	Y_RNA	ENST00000384484	misc_RNA	11059803	11060002	-	11059967	35	-	Y RNA [Source:RFAM;Acc:RF00019]
ENSG00000207231	Y_RNA	ENST00000384502	misc_RNA	186337041	1,86E+08	-	1,86E+08	35	-	Y RNA [Source:RFAM;Acc:RF00019]
ENSG00000222395	Y_RNA	ENST00000410463	misc_RNA	29843002	29843201	-	29843160	41	-	Y RNA [Source:RFAM;Acc:RF00019]
ENSG00000199831	7SK	ENST00000362961	misc_RNA	55842326	55842525	-	55842470	55	+	7SK RNA [Source:RFAM;Acc:RF00100]
ENSG00000222685	7SK	ENST00000410753	misc_RNA	47586725	47586924	-	47586845	79	+	7SK RNA [Source:RFAM;Acc:RF00100]
ENSG00000212437	SCARNA4	ENST00000391135	misc_RNA	131687258	1,32E+08	-	1,32E+08	38	+	Small Cajal body specific RNA 4 [Source:RFAM;Acc:RF00423]
ENSG00000223072	SRP_euk_arch	ENST00000411140	misc_RNA	58981973	58982172	-	58982113	59	+	Eukaryotic type signal recognition particle RNA [Source:RFAM;Acc:RF00017]
ENSG00000200615	Y_RNA	ENST00000363745	misc_RNA	33025692	33025891	-	33025870	21	+	Y RNA [Source:RFAM;Acc:RF00019]
ENSG00000201549	Y_RNA	ENST00000364679	misc_RNA	7936908	7937107	-	7937066	41	+	Y RNA [Source:RFAM;Acc:RF00019]
ENSG00000202041	Y_RNA	ENST00000365171	misc_RNA	240317873	2,4E+08	-	2,4E+08	44	+	Y RNA [Source:RFAM;Acc:RF00019]
ENSG00000207368	Y_RNA	ENST00000384638	misc_RNA	195140076	1,95E+08	-	1,95E+08	34	+	Y RNA [Source:RFAM;Acc:RF00019]
ENSG00000222511	Y_RNA	ENST00000410579	misc_RNA	8856532	8856731	-	8856686	45	+	Y RNA [Source:RFAM;Acc:RF00019]
ENSG00000222613	Y_RNA	ENST00000410681	misc_RNA	141700096	1,42E+08	-	1,42E+08	44	+	Y RNA [Source:RFAM;Acc:RF00019]
ENSG00000201315	7SK	ENST00000364445	misc_RNA	36726531	36726730	+	36726594	63	-	7SK RNA [Source:RFAM;Acc:RF00100]

ENSEMBL55 GeneID	Gene Symbol	ENSEMBL55 TranscriptID	Biotype	Gene start	Gene end	Strand	Motif start	TSS offset	Motif strand	Description
ENSG00000201583	7SK	ENST00000364713	misc_RNA	107859037	1,08E+08	+	1,08E+08	40	-	7SK RNA [Source:RFAM;Acc:RF00100]
ENSG00000222594	7SK	ENST00000410662	misc_RNA	145956284	1,46E+08	+	1,46E+08	54	-	7SK RNA [Source:RFAM;Acc:RF00100]
ENSG00000223026	7SK	ENST00000411094	misc_RNA	81717474	81717673	+	81717525	51	-	7SK RNA [Source:RFAM;Acc:RF00100]
ENSG00000223269	7SK	ENST00000411337	misc_RNA	98990906	98991105	+	98990966	60	-	7SK RNA [Source:RFAM;Acc:RF00100]
ENSG00000212212	SCARNA1	ENST00000390910	misc_RNA	28160912	28161111	+	28160967	55	-	Small Cajal body specific RNA 1 [Source:RFAM;Acc:RF00553]
ENSG00000222671	SRP_euk_arch	ENST00000410739	misc_RNA	231029887	2,31E+08	+	2,31E+08	37	-	Eukaryotic type signal recognition particle RNA [Source:RFAM;Acc:RF00017]
ENSG00000222885	SRP_euk_arch	ENST00000410953	misc_RNA	76709067	76709266	+	76709114	47	-	Eukaryotic type signal recognition particle RNA [Source:RFAM;Acc:RF00017]
ENSG00000223127	SRP_euk_arch	ENST00000411195	misc_RNA	99324234	99324433	+	99324281	47	-	Eukaryotic type signal recognition particle RNA [Source:RFAM;Acc:RF00017]
ENSG00000201676	Y_RNA	ENST00000364806	misc_RNA	60698893	60699092	+	60698914	21	-	Y RNA [Source:RFAM;Acc:RF00019]
ENSG00000202522	Y_RNA	ENST00000365652	misc_RNA	72477049	72477248	+	72477071	22	-	Y RNA [Source:RFAM;Acc:RF00019]
ENSG00000200714	Y_RNA	ENST00000363844	misc_RNA	66504966	66505165	+	66505044	78	+	Y RNA [Source:RFAM;Acc:RF00019]
ENSG00000206806	Y_RNA	ENST00000384079	misc_RNA	40373114	40373313	+	40373137	23	+	Y RNA [Source:RFAM;Acc:RF00019]
ENSG00000223023	Y_RNA	ENST00000411091	misc_RNA	20604559	20604758	+	20604582	23	+	Y RNA [Source:RFAM;Acc:RF00019]
ENSG00000184586	KRTAP7-1	ENST00000452750	polymorphic_pseudogene	32201879	32202078	-	32202037	41	-	keratin associated protein 7-1 [Source:RefSeq peptide;Acc:NP_853637]
ENSG00000227038	AC005077.9	ENST00000434037	processed_transcript	75737898	75738097	-	75738021	76	-	
ENSG00000227038	AC005077.9	ENST00000443068	processed_transcript	75737882	75738081	-	75738021	60	-	
ENSG00000232006	AC005537.2	ENST00000456114	processed_transcript	43084005	43084204	-	43084152	52	-	
ENSG00000167117	AC005921.2	ENST00000450727	processed_transcript	48844678	48844877	-	48844843	34	-	chromosome 17 open reading frame 73 (C17orf73), non-coding RNA [Source:RefSeq DNA;Acc:NR_024626]
ENSG00000167117	AC005921.2	ENST00000300458	processed_transcript	48844680	48844879	-	48844843	36	-	chromosome 17 open reading frame 73 (C17orf73), non-coding RNA [Source:RefSeq DNA;Acc:NR_024626]
ENSG00000167117	AC005921.2	ENST00000419688	processed_transcript	48844679	48844878	-	48844843	35	-	chromosome 17 open reading frame 73 (C17orf73), non-coding RNA [Source:RefSeq DNA;Acc:NR_024626]
ENSG00000228246	AC006028.9	ENST00000412266	processed_transcript	2762423	2762622	-	2762554	68	-	
ENSG00000232931	AC008268.3	ENST00000448494	processed_transcript	96481764	96481963	-	96481866	97	-	
ENSG00000236141	AC013727.2	ENST00000447761	processed_transcript	104485947	1,04E+08	-	1,04E+08	79	-	
ENSG00000237057	AC015922.6	ENST00000418821	processed_transcript	15720744	15720943	-	15720851	92	-	
ENSG00000229920	AC016734.3	ENST00000449995	processed_transcript	63870723	63870922	-	63870833	89	-	
ENSG00000147761	AC017019.3	ENST00000447655	processed_transcript	6898715	6898914	-	6898873	41	-	testis-specific transcript, Y-linked 7-like (LOC100101120), non-coding RNA [Source:RefSeq DNA;Acc:NR_003592]
ENSG00000224771	AC018502.1	ENST00000419591	processed_transcript	10668293	10668492	-	10668417	75	-	
ENSG00000236081	AC074389.9	ENST00000415399	processed_transcript	1781728	1781927	-	1781872	55	-	
ENSG00000236081	AC074389.9	ENST00000453348	processed_transcript	1781709	1781908	-	1781872	36	-	
ENSG00000234943	AC092839.4	ENST00000431130	processed_transcript	54773615	54773814	-	54773782	32	-	

ENSEMBL55 GeneID	Gene Symbol	ENSEMBL55 TranscriptID	Biotype	Gene start	Gene end	Strand	Motif start	TSS offset	Motif strand	Description
ENSG00000231062	AC103563.9	ENST00000442200	processed_transcript	95718722	95718921	-	95718867	54	-	
ENSG00000228740	AC136896.1	ENST00000439938	processed_transcript	27406223	27406422	-	27406352	70	-	
ENSG00000236532	AL035610.2	ENST00000453420	processed_transcript	29600787	29600986	-	29600944	42	-	
ENSG00000223608	AP001415.1	ENST00000417138	processed_transcript	39382721	39382920	-	39382833	87	-	
ENSG00000225278	BX571672.5	ENST00000412196	processed_transcript	143237516	1,43E+08	-	1,43E+08	27	-	
ENSG00000225278	BX571672.5	ENST00000449527	processed_transcript	143237516	1,43E+08	-	1,43E+08	27	-	
ENSG00000225278	BX571672.5	ENST00000456568	processed_transcript	143237516	1,43E+08	-	1,43E+08	27	-	
ENSG00000236858	CTA-992D9.6	ENST00000453934	processed_transcript	27456281	27456480	-	27456412	68	-	
ENSG00000227802	DNAJB3	ENST00000446806	processed_transcript	234652363	2,35E+08	-	2,35E+08	56	-	DnaJ (Hsp40) homolog, subfamily B, member 3 (DNAJB3), mRNA [Source:RefSeq DNA;Acc:NM_001001394]
ENSG00000232316	RP1-124C6.1	ENST00000421737	processed_transcript	113754424	1,14E+08	-	1,14E+08	39	-	
ENSG00000223492	RP11-15M15.1	ENST00000432706	processed_transcript	51795890	51796089	-	51796000	89	-	
ENSG00000233610	RP11-165D7.4	ENST00000425350	processed_transcript	49152025	49152224	-	49152155	69	-	
ENSG00000229454	RP11-202I11.2	ENST00000447148	processed_transcript	88379828	88380027	-	88379952	75	-	
ENSG00000234506	RP11-274B18.2	ENST00000446290	processed_transcript	71161306	71161505	-	71161438	67	-	
ENSG00000234222	RP11-315I20.1	ENST00000447686	processed_transcript	145508063	1,46E+08	-	1,46E+08	36	-	
ENSG00000227165	RP11-323P17.1	ENST00000456120	processed_transcript	122536197	1,23E+08	-	1,23E+08	93	-	
ENSG00000230575	RP11-369F10.2	ENST00000412137	processed_transcript	78198334	78198533	-	78198444	89	-	
ENSG00000232704	RP11-369F10.3	ENST00000423541	processed_transcript	78044643	78044842	-	78044782	60	-	
ENSG00000233672	RP11-40A8.3	ENST00000454605	processed_transcript	51484649	51484848	-	51484792	56	-	
ENSG00000231252	RP11-436K8.1	ENST00000423403	processed_transcript	61291057	61291256	-	61291165	91	-	
ENSG00000237895	RP11-460I13.5	ENST00000450821	processed_transcript	28357224	28357423	-	28357371	52	-	
ENSG00000231612	RP11-522M21.3	ENST00000414565	processed_transcript	245839581	2,46E+08	-	2,46E+08	41	-	
ENSG00000231612	RP11-522M21.3	ENST00000414565	processed_transcript	245839581	2,46E+08	-	2,46E+08	31	-	
ENSG00000227185	RP11-544D21.1	ENST00000442410	processed_transcript	239461038	2,39E+08	-	2,39E+08	61	-	
ENSG00000233735	RP11-567G24.3	ENST00000412311	processed_transcript	240342745	2,4E+08	-	2,4E+08	33	-	
ENSG00000233735	RP11-567G24.3	ENST00000444308	processed_transcript	240342745	2,4E+08	-	2,4E+08	33	-	
ENSG00000229578	RP11-71L7.1	ENST00000432697	processed_transcript	62603482	62603681	-	62603638	43	-	
ENSG00000226409	RP11-735G4.1	ENST00000422227	processed_transcript	125695271	1,26E+08	-	1,26E+08	61	-	
ENSG00000232286	RP11-80K6.2	ENST00000426963	processed_transcript	51479195	51479394	-	51479324	70	-	

ENSEMBL55 GeneID	Gene Symbol	ENSEMBL55 TranscriptID	Biotype	Gene start	Gene end	Strand	Motif start	TSS offset	Motif strand	Description
ENSG00000236494	RP11-89N17.4	ENST00000440034	processed_transcript	33842569	33842768	-	33842670	98	-	
ENSG00000236494	RP11-89N17.4	ENST00000420185	processed_transcript	33842543	33842742	-	33842670	72	-	
ENSG00000224729	RP13-530H6.2	ENST00000446022	processed_transcript	100201630	1E+08	-	1E+08	62	-	
ENSG00000236997	RP5-934G17.2	ENST00000442242	processed_transcript	11883138	11883337	-	11883290	47	-	
ENSG00000232907	RP5-977B1.7	ENST00000425233	processed_transcript	35201188	35201387	-	35201328	59	-	
ENSG00000226367	ST7OT2	ENST00000446784	processed_transcript	116714579	1,17E+08	-	1,17E+08	57	-	ST7 overlapping transcript 2 (non-protein coding) (ST7OT2), non-coding RNA [Source:RefSeq DNA;Acc:NR_002331]
ENSG00000223969	AC002456.2	ENST00000415965	processed_transcript	90224863	90225062	-	90225016	46	+	
ENSG00000229192	AC004870.6	ENST00000453267	processed_transcript	47065969	47066168	-	47066069	99	+	
ENSG00000228747	AC005075.5	ENST00000447058	processed_transcript	87860890	87861089	-	87861020	69	+	
ENSG00000228509	AC006460.2	ENST00000428032	processed_transcript	191566073	1,92E+08	-	1,92E+08	69	+	
ENSG00000226797	AC015923.1	ENST00000431604	processed_transcript	61491474	61491673	-	61491580	93	+	
ENSG00000232451	AC016768.1	ENST00000421581	processed_transcript	23313509	23313708	-	23313627	81	+	
ENSG00000223950	AC021937.1	ENST00000419834	processed_transcript	119111445	1,19E+08	-	1,19E+08	55	+	
ENSG00000206567	AC022007.5	ENST00000428375	processed_transcript	10050148	10050347	-	10050313	34	+	hypothetical LOC401052 (LOC401052), mRNA [Source:RefSeq DNA;Acc:NM_001008737]
ENSG00000172965	AC068491.1	ENST00000451884	processed_transcript	112123092	1,12E+08	-	1,12E+08	64	+	hypothetical LOC541471 (LOC541471), transcript variant 2, non-coding RNA [Source:RefSeq DNA;Acc:NR_024373]
ENSG00000236780	AC078941.1	ENST00000447453	processed_transcript	67404925	67405124	-	67405049	75	+	
ENSG00000230552	AC092162.1	ENST00000438143	processed_transcript	177630640	1,78E+08	-	1,78E+08	89	+	
ENSG00000234877	AC092660.1	ENST00000420648	processed_transcript	78826333	78826532	-	78826440	92	+	
ENSG00000226383	AC093375.1	ENST00000448255	processed_transcript	156880926	1,57E+08	-	1,57E+08	50	+	
ENSG00000238160	AC116366.5	ENST00000454380	processed_transcript	131762207	1,32E+08	-	1,32E+08	44	+	
ENSG00000236532	AL035610.2	ENST00000433303	processed_transcript	29542263	29542462	-	29542377	85	+	
ENSG00000204623	C6orf12	ENST00000452229	processed_transcript	30028557	30028756	-	30028709	47	+	non-protein coding RNA 171 (NCRNA00171), non-coding RNA [Source:RefSeq DNA;Acc:NR_026751]
ENSG00000229012	GS1-542M4.4	ENST00000440430	processed_transcript	8835443	8835642	-	8835567	75	+	
ENSG00000197549	PRAMEL	ENST00000419303	processed_transcript	22398057	22398256	-	22398232	24	+	
ENSG00000220504	RP1-214M20.2	ENST00000406231	processed_transcript	52822120	52822319	-	52822248	71	+	
ENSG00000227610	RP1-3D11.2	ENST00000415252	processed_transcript	106788852	1,07E+08	-	1,07E+08	76	+	
ENSG00000231298	RP11-117P22.1	ENST00000430998	processed_transcript	4720147	4720346	-	4720248	98	+	hypothetical LOC100216001 (LOC100216001), non-coding RNA [Source:RefSeq DNA;Acc:NR_024475]
ENSG00000231298	RP11-117P22.1	ENST00000449712	processed_transcript	4720125	4720324	-	4720248	76	+	hypothetical LOC100216001 (LOC100216001), non-coding RNA [Source:RefSeq DNA;Acc:NR_024475]
ENSG00000226599	RP11-136K14.3	ENST00000455229	processed_transcript	150972962	1,51E+08	-	1,51E+08	46	+	
ENSG00000223729	RP11-145E17.2	ENST00000417135	processed_transcript	137187044	1,37E+08	-	1,37E+08	95	+	

ENSEMBL55 GeneID	Gene Symbol	ENSEMBL55 TranscriptID	Biotype	Gene start	Gene end	Strand	Motif start	TSS offset	Motif strand	Description
ENSG00000224798	RP11-195L15.2	ENST00000424106	processed_transcript	50354568	50354767	-	50354687	80	+	
ENSG00000228327	RP11-206L10.2	ENST00000417659	processed_transcript	700106	700305	-	700256	49	+	
ENSG00000227527	RP11-223A3.1	ENST00000436207	processed_transcript	42803848	42804047	-	42803956	91	+	
ENSG00000237463	RP11-280O1.2	ENST00000452283	processed_transcript	165508650	1,66E+08	-	1,66E+08	24	+	hypothetical LOC400794 (LOC400794), non-coding RNA [Source:RefSeq DNA;Acc:NR_026744]
ENSG00000234076	RP11-297K7.2	ENST00000444488	processed_transcript	188665229	1,89E+08	-	1,89E+08	68	+	
ENSG00000227165	RP11-323P17.1	ENST00000451706	processed_transcript	122551238	1,23E+08	-	1,23E+08	91	+	
ENSG00000225065	RP11-346K17.4	ENST00000434040	processed_transcript	33302233	33302432	-	33302359	73	+	
ENSG00000215483	RP11-350A18.1	ENST00000400431	processed_transcript	41034347	41034546	-	41034467	79	+	
ENSG00000236758	RP11-351N4.3	ENST00000452602	processed_transcript	29824492	29824691	-	29824638	53	+	
ENSG00000236758	RP11-351N4.3	ENST00000434779	processed_transcript	29824492	29824691	-	29824638	53	+	
ENSG00000236758	RP11-351N4.3	ENST00000417624	processed_transcript	29824492	29824691	-	29824638	53	+	
ENSG00000230554	RP11-394I17.1	ENST00000448320	processed_transcript	45228106	45228305	-	45228252	53	+	
ENSG00000230200	RP11-426A6.8	ENST00000412464	processed_transcript	138356527	1,38E+08	-	1,38E+08	63	+	
ENSG00000215866	RP11-426L16.8	ENST00000401018	processed_transcript	113393066	1,13E+08	-	1,13E+08	57	+	
ENSG00000224460	RP11-439L18.2	ENST00000421237	processed_transcript	143277636	1,43E+08	-	1,43E+08	56	+	
ENSG00000232586	RP11-46A10.4	ENST00000415647	processed_transcript	180923824	1,81E+08	-	1,81E+08	50	+	
ENSG00000238230	RP11-477B16.4	ENST00000433569	processed_transcript	95354917	95355116	-	95355074	42	+	
ENSG00000224876	RP11-49G10.3	ENST00000419613	processed_transcript	31804674	31804873	-	31804826	47	+	
ENSG00000227185	RP11-544D21.1	ENST00000442410	processed_transcript	239461038	2,39E+08	-	2,39E+08	60	+	
ENSG00000233735	RP11-567G24.3	ENST00000412311	processed_transcript	240342745	2,4E+08	-	2,4E+08	32	+	
ENSG00000233735	RP11-567G24.3	ENST00000444308	processed_transcript	240342745	2,4E+08	-	2,4E+08	32	+	
ENSG00000177553	RP11-56N19.5	ENST00000376620	processed_transcript	11839477	11839676	-	11839647	29	+	
ENSG00000232774	RP11-786A20.1	ENST00000450545	processed_transcript	113138554	1,13E+08	-	1,13E+08	98	+	
ENSG00000237011	RP11-98G13.1	ENST00000447056	processed_transcript	193657360	1,94E+08	-	1,94E+08	67	+	
ENSG00000234147	RP3-460G2.2	ENST00000455011	processed_transcript	141173862	1,41E+08	-	1,41E+08	23	+	
ENSG00000204018	RP4-683M8.1	ENST00000435576	processed_transcript	48460073	48460272	-	48460228	44	+	
ENSG00000231265	RP4-710H13.2	ENST00000431460	processed_transcript	48657842	48658041	-	48658010	31	+	
ENSG00000230124	RP5-	ENST00000415414	processed_transcript	180243617	1,8E+08	-	1,8E+08	56	+	

ENSEMBL55 GeneID	Gene Symbol	ENSEMBL55 TranscriptID	Biotype	Gene start	Gene end	Strand	Motif start	TSS offset	Motif strand	Description
	1180C10.2									
ENSG00000236997	RP5-934G17.2	ENST00000442242	processed_transcript	11883138	11883337	-	11883291	46	+	
ENSG00000215889	SKINTL	ENST00000453388	processed_transcript	48647901	48648100	-	48648022	78	+	Skint-like (pseudogene) (SKINTL), non-coding RNA [Source:RefSeq DNA;Acc:NR_026749]
ENSG00000164519	AC004080.12	ENST00000449765	processed_transcript	27161538	27161737	+	27161593	55	-	
ENSG00000226690	AC005281.1	ENST00000443874	processed_transcript	12544025	12544224	+	12544119	94	-	
ENSG00000226690	AC005281.1	ENST00000424453	processed_transcript	12544050	12544249	+	12544119	69	-	
ENSG00000237327	AC005539.4	ENST00000439185	processed_transcript	158984469	1,59E+08	+	1,59E+08	41	-	
ENSG00000226323	AC006150.1	ENST00000412014	processed_transcript	14853756	14853955	+	14853798	42	-	
ENSG00000231815	AC007179.1	ENST00000415159	processed_transcript	59662348	59662547	+	59662405	57	-	
ENSG00000238273	AC012360.6	ENST00000457290	processed_transcript	105992323	1,06E+08	+	1,06E+08	88	-	
ENSG00000229131	AC016710.1	ENST00000421326	processed_transcript	140227345	1,4E+08	+	1,4E+08	32	-	
ENSG00000235047	AC016751.3	ENST00000453206	processed_transcript	176762064	1,77E+08	+	1,77E+08	89	-	
ENSG00000226320	AC018359.1	ENST00000424786	processed_transcript	34200826	34201025	+	34200859	33	-	
ENSG00000142396	AC020915.4	ENST00000434052	processed_transcript	58816743	58816942	+	58816842	99	-	
ENSG00000231770	AC046143.5	ENST00000453671	processed_transcript	194304740	1,94E+08	+	1,94E+08	24	-	
ENSG00000225537	AC073325.1	ENST00000443714	processed_transcript	45500311	45500510	+	45500402	91	-	
ENSG00000220804	AC093642.5	ENST00000416103	processed_transcript	243037045	2,43E+08	+	2,43E+08	34	-	hypothetical LOC728323 (LOC728323), non-coding RNA [Source:RefSeq DNA;Acc:NR_024437]
ENSG00000224257	AC107218.2	ENST00000416430	processed_transcript	215374920	2,15E+08	+	2,15E+08	32	-	
ENSG00000215383	AC118278.2	ENST00000400172	processed_transcript	154872	155071	+	154956	84	-	
ENSG00000224652	AC139666.1	ENST00000457079	processed_transcript	195869507	1,96E+08	+	1,96E+08	30	-	
ENSG00000238261	BX004987.5	ENST00000416712	processed_transcript	143217496	1,43E+08	+	1,43E+08	92	-	
ENSG00000237517	DGCR5	ENST00000424407	processed_transcript	18985787	18985986	+	18985850	63	-	
ENSG00000229481	LOC147976	ENST00000443870	processed_transcript	38320191	38320390	+	38320236	45	-	
ENSG00000214548	MEG3	ENST00000424076	processed_transcript	101295410	1,01E+08	+	1,01E+08	33	-	maternally expressed 3 (non-protein coding) (MEG3), transcript variant 1, non-coding RNA [Source:RefSeq DNA;Acc:NR_002766]
ENSG00000174680	NCRNA00110	ENST00000423221	processed_transcript	31121266	31121465	+	31121354	88	-	non-protein coding RNA 110 (NCRNA00110), non-coding RNA [Source:RefSeq DNA;Acc:NR_027021]
ENSG00000174680	NCRNA00110	ENST00000309331	processed_transcript	31121257	31121456	+	31121354	97	-	non-protein coding RNA 110 (NCRNA00110), non-coding RNA [Source:RefSeq DNA;Acc:NR_027021]
ENSG00000236546	RP1-118J21.5	ENST00000418255	processed_transcript	40363417	40363616	+	40363514	97	-	
ENSG00000231944	RP1-172N19.2	ENST00000420998	processed_transcript	71908800	71908999	+	71908872	72	-	
ENSG00000236823	RP1-249F5.3	ENST00000434562	processed_transcript	160320218	1,6E+08	+	1,6E+08	34	-	
ENSG00000235939	RP11-123B3.2	ENST00000423283	processed_transcript	50627323	50627522	+	50627422	99	-	
ENSG00000226835	RP11-	ENST00000418242	processed_transcript	94798823	94799022	+	94798875	52	-	

ENSEMBL55 GeneID	Gene Symbol	ENSEMBL55 TranscriptID	Biotype	Gene start	Gene end	Strand	Motif start	TSS offset	Motif strand	Description
	148B18.3									
ENSG00000229918	RP11-155N3.3	ENST00000439367	processed_transcript	99484338	99484537	+	99484404	66	-	
ENSG00000179766	RP11-156G14.1	ENST00000417941	processed_transcript	35478236	35478435	+	35478317	81	-	ATPase, Class I, type 8B family pseudogene (LOC158381), transcript variant 2, non-coding RNA [Source:RefSeq DNA;Acc:NR_003582]
ENSG00000226009	RP11-190J1.10	ENST00000412353	processed_transcript	103578835	1,04E+08	+	1,04E+08	81	-	
ENSG00000229015	RP11-265D19.6	ENST00000439434	processed_transcript	134530354	1,35E+08	+	1,35E+08	45	-	
ENSG00000230156	RP11-297I6.4	ENST00000421601	processed_transcript	107306264	1,07E+08	+	1,07E+08	97	-	
ENSG00000224500	RP11-328K22.1	ENST00000413564	processed_transcript	79073699	79073898	+	79073798	99	-	
ENSG00000235819	RP11-359J6.1	ENST00000438582	processed_transcript	89029197	89029396	+	89029254	57	-	
ENSG00000237321	RP11-374A22.1	ENST00000441521	processed_transcript	124144385	1,24E+08	+	1,24E+08	85	-	
ENSG00000236924	RP11-390F4.6	ENST00000413145	processed_transcript	6645956	6646155	+	6646042	86	-	
ENSG00000230945	RP11-394O9.1	ENST00000417801	processed_transcript	82645494	82645693	+	82645575	81	-	
ENSG00000238156	RP11-415D17.4	ENST00000455530	processed_transcript	76164660	76164859	+	76164686	26	-	
ENSG00000231633	RP11-484I6.2	ENST00000430111	processed_transcript	103395340	1,03E+08	+	1,03E+08	70	-	
ENSG00000230815	RP11-49O14.3	ENST00000437846	processed_transcript	97586554	97586753	+	97586594	40	-	
ENSG00000234860	RP11-547C13.1	ENST00000413271	processed_transcript	102293978	1,02E+08	+	1,02E+08	38	-	
ENSG00000231724	RP11-573D15.3	ENST00000434957	processed_transcript	186525481	1,87E+08	+	1,87E+08	46	-	
ENSG00000232532	RP11-63K6.7	ENST00000458201	processed_transcript	90629915	90630114	+	90630000	85	-	
ENSG00000237914	RP11-77C3.3	ENST00000456177	processed_transcript	1614154	1614353	+	1614251	97	-	
ENSG00000233403	RP11-86A5.1	ENST00000423914	processed_transcript	25896456	25896655	+	25896530	74	-	
ENSG00000238210	RP13-210D15.4	ENST00000438052	processed_transcript	134386009	1,34E+08	+	1,34E+08	50	-	
ENSG00000230597	RP3-331H24.4	ENST00000456795	processed_transcript	72038645	72038844	+	72038730	85	-	
ENSG00000237282	RP4-568F9.7	ENST00000418896	processed_transcript	18361416	18361615	+	18361450	34	-	
ENSG00000197670	RP4-724E16.2	ENST00000371487	processed_transcript	52169309	52169508	+	52169407	98	-	
ENSG00000197670	RP4-724E16.2	ENST00000424252	processed_transcript	52169309	52169508	+	52169407	98	-	
ENSG00000231816	RP4-782L23.1	ENST00000424725	processed_transcript	60238467	60238666	+	60238492	25	-	
ENSG00000230615	RP5-1198O20.4	ENST00000431800	processed_transcript	44514049	44514248	+	44514123	74	-	
ENSG00000230615	RP5-1198O20.4	ENST00000437643	processed_transcript	44514064	44514263	+	44514123	59	-	
ENSG00000228436	RP5-864K19.4	ENST00000456813	processed_transcript	39325755	39325954	+	39325793	38	-	

ENSEMBL55 GeneID	Gene Symbol	ENSEMBL55 TranscriptID	Biotype	Gene start	Gene end	Strand	Motif start	TSS offset	Motif strand	Description
ENSG00000234859	AC003958.2	ENST00000432258	processed_transcript	39558675	39558874	+	39558745	70	+	
ENSG00000234859	AC003958.2	ENST00000430006	processed_transcript	39558668	39558867	+	39558745	77	+	
ENSG00000170858	AC006293.1	ENST00000413439	processed_transcript	55219700	55219899	+	55219725	25	+	
ENSG00000227028	AC007254.3	ENST00000417875	processed_transcript	40478563	40478762	+	40478659	96	+	
ENSG00000237298	AC009948.3	ENST00000431259	processed_transcript	179388226	1,79E+08	+	1,79E+08	96	+	
ENSG00000229618	AC011288.2	ENST00000411542	processed_transcript	13141097	13141296	+	13141180	83	+	
ENSG00000227279	AC015933.2	ENST00000423367	processed_transcript	25534483	25534682	+	25534511	28	+	
ENSG00000228363	AC015971.2	ENST00000424788	processed_transcript	86831413	86831612	+	86831503	90	+	
ENSG00000237856	AC062020.2	ENST00000435441	processed_transcript	123822897	1,24E+08	+	1,24E+08	77	+	
ENSG00000189229	AC069277.2	ENST00000432743	processed_transcript	6736410	6736609	+	6736491	81	+	
ENSG00000225444	AC087073.1	ENST00000435357	processed_transcript	52600256	52600455	+	52600320	64	+	
ENSG00000236856	AC105393.1	ENST00000431911	processed_transcript	388412	388611	+	388497	85	+	
ENSG00000237077	AC105399.1	ENST00000447303	processed_transcript	78019947	78020146	+	78020009	62	+	
ENSG00000227256	AP000266.5	ENST00000453549	processed_transcript	33650174	33650373	+	33650272	98	+	
ENSG00000171671	C11orf76	ENST00000393738	processed_transcript	70709388	70709587	+	70709471	83	+	
ENSG00000226471	CTA-292E10.6	ENST00000422972	processed_transcript	29214319	29214518	+	29214384	65	+	
ENSG00000188693	CTB-161K23.1	ENST00000453068	processed_transcript	91763918	91764117	+	91763995	77	+	
ENSG00000230366	DSCR9	ENST00000454482	processed_transcript	38580804	38581003	+	38580827	23	+	Down syndrome critical region gene 9 (non-protein coding) (DSCR9), non-coding RNA [Source:RefSeq DNA;Acc:NR_026719]
ENSG00000223345	HIST2H2BB	ENST00000430394	processed_transcript	120906028	1,21E+08	+	1,21E+08	22	+	histone cluster 2, H2ba (HIST2H2BA), non-coding RNA [Source:RefSeq DNA;Acc:NR_027337]
ENSG00000180537	RNF182	ENST00000422158	processed_transcript	13977351	13977550	+	13977414	63	+	ring finger protein 182 (RNF182), mRNA [Source:RefSeq DNA;Acc:NM_152737]
ENSG00000237564	RP1-71H19.2	ENST00000421261	processed_transcript	57358736	57358935	+	57358814	78	+	
ENSG00000228127	RP11-12L8.1	ENST00000438885	processed_transcript	116461997	1,16E+08	+	1,16E+08	44	+	
ENSG00000235140	RP11-135D11.2	ENST00000450677	processed_transcript	61348908	61349107	+	61348937	29	+	
ENSG00000234464	RP11-136B18.2	ENST00000422844	processed_transcript	238439337	2,38E+08	+	2,38E+08	40	+	
ENSG00000235903	RP11-139H14.3	ENST00000415033	processed_transcript	46669028	46669227	+	46669077	49	+	
ENSG00000179766	RP11-156G14.1	ENST00000417941	processed_transcript	35478236	35478435	+	35478318	82	+	ATPase, Class I, type 8B family pseudogene (LOC158381), transcript variant 2, non-coding RNA [Source:RefSeq DNA;Acc:NR_003582]
ENSG00000225497	RP11-180I22.2	ENST00000428936	processed_transcript	78907410	78907609	+	78907445	35	+	
ENSG00000229015	RP11-265D19.6	ENST00000439434	processed_transcript	134530354	1,35E+08	+	1,35E+08	56	+	
ENSG00000228886	RP11-290D2.3	ENST00000420693	processed_transcript	45924458	45924657	+	45924483	25	+	

ENSEMBL55 GeneID	Gene Symbol	ENSEMBL55 TranscriptID	Biotype	Gene start	Gene end	Strand	Motif start	TSS offset	Motif strand	Description
ENSG00000224843	RP11-373D17.1	ENST00000424968	processed_transcript	26988232	26988431	+	26988268	36	+	
ENSG00000224843	RP11-373D17.1	ENST00000420081	processed_transcript	26988415	26988614	+	26988483	68	+	
ENSG00000224843	RP11-373D17.1	ENST00000420081	processed_transcript	26988415	26988614	+	26988447	32	+	
ENSG00000234535	RP11-37L2.1	ENST00000442716	processed_transcript	34185104	34185303	+	34185181	77	+	
ENSG00000233334	RP11-464O2.2	ENST00000432699	processed_transcript	126392753	1,26E+08	+	1,26E+08	90	+	
ENSG00000229984	RP11-479O17.5	ENST00000421889	processed_transcript	81691467	81691666	+	81691536	69	+	
ENSG00000234996	RP11-480I12.9	ENST00000438944	processed_transcript	202842422	2,03E+08	+	2,03E+08	97	+	actin pseudogene (LOC148709), non-coding RNA [Source:RefSeq DNA;Acc:NR_002929]
ENSG00000237786	RP11-501I19.3	ENST00000446001	processed_transcript	13486526	13486725	+	13486558	32	+	
ENSG00000185495	RP11-504P24.4	ENST00000332749	processed_transcript	224196429	2,24E+08	+	2,24E+08	86	+	
ENSG00000231082	RP11-514F8.2	ENST00000436500	processed_transcript	82409553	82409752	+	82409577	24	+	
ENSG00000214652	RP11-561N12.2	ENST00000430271	processed_transcript	63539167	63539366	+	63539191	24	+	
ENSG00000231345	RP11-564C4.7	ENST00000449695	processed_transcript	52415727	52415926	+	52415796	69	+	
ENSG00000231345	RP11-564C4.7	ENST00000449695	processed_transcript	52415727	52415926	+	52415786	59	+	
ENSG00000231616	RP11-575L7.4	ENST00000421734	processed_transcript	86473883	86474082	+	86473947	64	+	
ENSG00000228372	RP11-85L21.4	ENST00000453235	processed_transcript	134255567	1,34E+08	+	1,34E+08	75	+	
ENSG00000238210	RP13-210D15.4	ENST00000438052	processed_transcript	134386009	1,34E+08	+	1,34E+08	61	+	
ENSG00000235703	RP13-507I23.1	ENST00000450989	processed_transcript	149113851	1,49E+08	+	1,49E+08	27	+	hypothetical LOC100272228 (LOC100272228), non-coding RNA [Source:RefSeq DNA;Acc:NR_027456]
ENSG00000229313	RP3-425P12.5	ENST00000428903	processed_transcript	25042067	25042266	+	25042126	59	+	
ENSG00000233969	RP5-1006K12.1	ENST00000421825	processed_transcript	119808399	1,2E+08	+	1,2E+08	55	+	
ENSG00000226445	XXyac-YX65C7_A.2	ENST00000444188	processed_transcript	169613349	1,7E+08	+	1,7E+08	28	+	
ENSG00000228383	AC006158.7	ENST00000433321	protein_coding	6563571	6563770	-	6563671	99	-	chromosome Y open reading frame 16 (CYorf16), non-coding RNA [Source:RefSeq DNA;Acc:NR_001553]
ENSG00000182774	AC010724.6-6	ENST00000330244	protein_coding	83209009	83209208	-	83209139	69	-	40S ribosomal protein S17 [Source:UniProtKB/Swiss-Prot;Acc:P08708]
ENSG00000182774	AC010724.6-6	ENST00000330244	protein_coding	83209009	83209208	-	83209129	79	-	40S ribosomal protein S17 [Source:UniProtKB/Swiss-Prot;Acc:P08708]
ENSG00000233539	AC011294.3	ENST00000451905	protein_coding	46736521	46736720	-	46736650	70	-	
ENSG00000227774	AC016866.12	ENST00000427633	protein_coding	46545886	46546085	-	46545998	87	-	CDNA FLJ26679 fis, clone MPG04418 [Source:UniProtKB/TrEMBL;Acc:Q6ZP24]
ENSG00000213809	AC022075.29	ENST00000396451	protein_coding	10542441	10542640	-	10542564	76	-	NKG2-D type II integral membrane protein (NKG2-D-activating NK receptor)(NK cell receptor D)(Killer cell lectin-like receptor subfamily K member 1)(CD314 antigen)

ENSEMBL55 GeneID	Gene Symbol	ENSEMBL55 TranscriptID	Biotype	Gene start	Gene end	Strand	Motif start	TSS offset	Motif strand	Description
										[Source:UniProtKB/Swiss-Prot;Acc:P26718]
ENSG00000213809	AC022075.29	ENST00000240618	protein_coding	10542441	10542640	-	10542564	76	-	NKG2-D type II integral membrane protein (NKG2-D-activating NK receptor)(NK cell receptor D)(Killer cell lectin-like receptor subfamily K member 1)(CD314 antigen) [Source:UniProtKB/Swiss-Prot;Acc:P26718]
ENSG00000188014	AC023886.2	ENST00000313239	protein_coding	113508608	1,14E+08	-	1,14E+08	52	-	Uncharacterized protein FLJ44066 [Source:UniProtKB/Swiss-Prot;Acc:Q6ZU11]
ENSG00000224768	AC051642.5-2	ENST00000436094	protein_coding	23415470	23415669	-	23415608	61	-	
ENSG00000114126	AC112504.6-2	ENST00000317104	protein_coding	141868119	1,42E+08	-	1,42E+08	21	-	Transcription factor Dp-2 (E2F dimerization partner 2) [Source:UniProtKB/Swiss-Prot;Acc:Q14188]
ENSG00000114126	AC112504.6-2	ENST00000397991	protein_coding	141868119	1,42E+08	-	1,42E+08	21	-	Transcription factor Dp-2 (E2F dimerization partner 2) [Source:UniProtKB/Swiss-Prot;Acc:Q14188]
ENSG00000215769	AC132812.9	ENST00000400873	protein_coding	62758632	62758831	-	62758744	87	-	Importin subunit alpha-2-like protein [Source:UniProtKB/Swiss-Prot;Acc:A8MYJ9]
ENSG00000224734	AC135592.2-1	ENST00000442320	protein_coding	55942412	55942611	-	55942550	61	-	cDNA FLJ37640 fis, clone BRHIP1000174 [Source:UniProtKB/TrEMBL;Acc:Q8N9E6]
ENSG00000167769	ACER1	ENST00000301452	protein_coding	6333441	6333640	-	6333541	99	-	Alkaline ceramidase 1 (Alkaline CDase 1)(AlkCDase 1)(EC 3.5.1.23)(N-acylsphingosine amidohydrolase 3)(Acylsphingosine deacylase 3) [Source:UniProtKB/Swiss-Prot;Acc:Q8TDN7]
ENSG00000115170	ACVR1	ENST00000409283	protein_coding	158663310	1,59E+08	-	1,59E+08	81	-	Activin receptor type-1 Precursor (EC 2.7.11.30)(Activin receptor type I)(ACTR-I)(Serine/threonine-protein kinase receptor R1)(SKR1)(Activin receptor-like kinase 2)(ALK-2)(TGF-B superfamily receptor type I)(TSR-I) [Source:UniProtKB/Swiss-Prot;Acc:Q04771]
ENSG00000229049	AE000662.1-5	ENST00000415290	protein_coding	23015111	23015310	-	23015278	32	-	
ENSG00000135049	AGTPBP1	ENST00000357081	protein_coding	88356602	88356801	-	88356751	50	-	Cytosolic carboxypeptidase 1 (EC 3.4.17.-)(ATP/GTP-binding protein 1)(Nervous system nuclear protein induced by axotomy) [Source:UniProtKB/Swiss-Prot;Acc:Q9UPW5]
ENSG00000234760	AL035419.12	ENST00000446409	protein_coding	37200419	37200618	-	37200592	26	-	
ENSG00000228320	AL117190.6-1	ENST00000439283	protein_coding	101295338	1,01E+08	-	1,01E+08	94	-	PRO2160 [Source:UniProtKB/TrEMBL;Acc:Q9P183]
ENSG00000203949	AL590282.6	ENST00000370774	protein_coding	134384648	1,34E+08	-	1,34E+08	87	-	Putative uncharacterized protein ENSP00000359810 Fragment [Source:UniProtKB/TrEMBL;Acc:A8MSX2]
ENSG00000227957	AL596452.2	ENST00000419066	protein_coding	161499681	1,61E+08	-	1,61E+08	64	-	PRO2660 [Source:UniProtKB/TrEMBL;Acc:Q9H383]
ENSG00000160593	AMICA1	ENST00000292067	protein_coding	118083875	1,18E+08	-	1,18E+08	47	-	Junctional adhesion molecule-like Precursor (Dendritic cell-specific protein CREA7-1)(Adhesion molecule interacting with CXADR antigen 1) [Source:UniProtKB/Swiss-Prot;Acc:Q86YT9]
ENSG00000100325	ASCC2	ENST00000458594	protein_coding	30230340	30230539	-	30230493	46	-	Activating signal cointegrator 1 complex subunit 2 (ASC-1 complex subunit p100)(Trip4 complex subunit p100) [Source:UniProtKB/Swiss-Prot;Acc:Q9H1I8]
ENSG00000100325	ASCC2	ENST00000431535	protein_coding	30230340	30230539	-	30230493	46	-	Activating signal cointegrator 1 complex subunit 2 (ASC-1 complex subunit p100)(Trip4 complex subunit p100) [Source:UniProtKB/Swiss-Prot;Acc:Q9H1I8]
ENSG00000070669	ASNS	ENST00000454046	protein_coding	97498926	97499125	-	97499098	27	-	Asparagine synthetase [glutamine-hydrolyzing] (EC

ENSEMBL55 GeneID	Gene Symbol	ENSEMBL55 TranscriptID	Biotype	Gene start	Gene end	Strand	Motif start	TSS offset	Motif strand	Description
										6.3.5.4)(Glutamine-dependent asparagine synthetase)(Cell cycle control protein TS11) [Source:UniProtKB/Swiss-Prot;Acc:P08243]
ENSG00000119787	ATL2	ENST00000402054	protein_coding	38603167	38603366	-	38603338	28	-	Atlastin-2 (ADP-ribosylation factor-like protein 6-interacting protein 2)(ARL-6-interacting protein 2)(Aip-2) [Source:UniProtKB/Swiss-Prot;Acc:Q8NHH9]
ENSG00000198563	BAT1	ENST00000419020	protein_coding	31509581	31509780	-	31509692	88	-	Spliceosome RNA helicase BAT1 (EC 3.6.1.-)(DEAD box protein UAP56)(56 kDa U2AF65-associated protein)(ATP-dependent RNA helicase p47)(HLA-B-associated transcript-1) [Source:UniProtKB/Swiss-Prot;Acc:Q13838]
ENSG00000198563	BAT1	ENST00000456662	protein_coding	31509515	31509714	-	31509692	22	-	Spliceosome RNA helicase BAT1 (EC 3.6.1.-)(DEAD box protein UAP56)(56 kDa U2AF65-associated protein)(ATP-dependent RNA helicase p47)(HLA-B-associated transcript-1) [Source:UniProtKB/Swiss-Prot;Acc:Q13838]
ENSG00000198563	BAT1	ENST00000456976	protein_coding	31509564	31509763	-	31509692	71	-	Spliceosome RNA helicase BAT1 (EC 3.6.1.-)(DEAD box protein UAP56)(56 kDa U2AF65-associated protein)(ATP-dependent RNA helicase p47)(HLA-B-associated transcript-1) [Source:UniProtKB/Swiss-Prot;Acc:Q13838]
ENSG00000198563	BAT1	ENST00000428450	protein_coding	31509567	31509766	-	31509692	74	-	Spliceosome RNA helicase BAT1 (EC 3.6.1.-)(DEAD box protein UAP56)(56 kDa U2AF65-associated protein)(ATP-dependent RNA helicase p47)(HLA-B-associated transcript-1) [Source:UniProtKB/Swiss-Prot;Acc:Q13838]
ENSG00000198563	BAT1	ENST00000431908	protein_coding	31509559	31509758	-	31509692	66	-	Spliceosome RNA helicase BAT1 (EC 3.6.1.-)(DEAD box protein UAP56)(56 kDa U2AF65-associated protein)(ATP-dependent RNA helicase p47)(HLA-B-associated transcript-1) [Source:UniProtKB/Swiss-Prot;Acc:Q13838]
ENSG00000198563	BAT1	ENST00000418897	protein_coding	31509569	31509768	-	31509692	76	-	Spliceosome RNA helicase BAT1 (EC 3.6.1.-)(DEAD box protein UAP56)(56 kDa U2AF65-associated protein)(ATP-dependent RNA helicase p47)(HLA-B-associated transcript-1) [Source:UniProtKB/Swiss-Prot;Acc:Q13838]
ENSG00000123636	BAZ2B	ENST00000441143	protein_coding	160289262	1,6E+08	-	1,6E+08	50	-	Bromodomain adjacent to zinc finger domain protein 2B (hWALp4) [Source:UniProtKB/Swiss-Prot;Acc:Q9UIF8]
ENSG00000183337	BCOR	ENST00000378463	protein_coding	39927174	39927373	-	39927286	87	-	BCL-6 corepressor (BCoR) [Source:UniProtKB/Swiss-Prot;Acc:Q6W2J9]
ENSG00000010671	BTK	ENST00000443591	protein_coding	100640980	1,01E+08	-	1,01E+08	97	-	Tyrosine-protein kinase BTK (EC 2.7.10.2)(Bruton tyrosine kinase)(Agammaglobulinaemia tyrosine kinase)(ATK)(B-cell progenitor kinase)(BPK) [Source:UniProtKB/Swiss-Prot;Acc:Q06187]
ENSG00000010671	BTK	ENST00000372880	protein_coding	100640968	1,01E+08	-	1,01E+08	85	-	Tyrosine-protein kinase BTK (EC 2.7.10.2)(Bruton tyrosine kinase)(Agammaglobulinaemia tyrosine kinase)(ATK)(B-cell progenitor kinase)(BPK) [Source:UniProtKB/Swiss-Prot;Acc:Q06187]
ENSG00000001460	C1orf201	ENST00000435187	protein_coding	24717970	24718169	-	24718142	27	-	UPF0490 protein C1orf201 [Source:UniProtKB/Swiss-Prot;Acc:Q5TH74]
ENSG00000125462	C1orf61	ENST00000368243	protein_coding	156398985	1,56E+08	-	1,56E+08	97	-	Protein CROC-4 (Contingent replication of cDNA 4) [Source:UniProtKB/Swiss-Prot;Acc:Q13536]

ENSEMBL55 GeneID	Gene Symbol	ENSEMBL55 TranscriptID	Biotype	Gene start	Gene end	Strand	Motif start	TSS offset	Motif strand	Description
ENSG00000149636	C20orf172	ENST00000447406	protein_coding	35402003	35402202	-	35402136	66	-	Kinetochore-associated protein DSN1 homolog [Source:UniProtKB/Swiss-Prot;Acc:Q9H410]
ENSG00000149636	C20orf172	ENST00000448110	protein_coding	35402031	35402230	-	35402136	94	-	Kinetochore-associated protein DSN1 homolog [Source:UniProtKB/Swiss-Prot;Acc:Q9H410]
ENSG00000149636	C20orf172	ENST00000373734	protein_coding	35402031	35402230	-	35402136	94	-	Kinetochore-associated protein DSN1 homolog [Source:UniProtKB/Swiss-Prot;Acc:Q9H410]
ENSG00000149636	C20orf172	ENST00000449595	protein_coding	35401985	35402184	-	35402136	48	-	Kinetochore-associated protein DSN1 homolog [Source:UniProtKB/Swiss-Prot;Acc:Q9H410]
ENSG00000149636	C20orf172	ENST00000438549	protein_coding	35401974	35402173	-	35402136	37	-	Kinetochore-associated protein DSN1 homolog [Source:UniProtKB/Swiss-Prot;Acc:Q9H410]
ENSG00000149636	C20orf172	ENST00000373750	protein_coding	35402031	35402230	-	35402136	94	-	Kinetochore-associated protein DSN1 homolog [Source:UniProtKB/Swiss-Prot;Acc:Q9H410]
ENSG00000149636	C20orf172	ENST00000373740	protein_coding	35402031	35402230	-	35402136	94	-	Kinetochore-associated protein DSN1 homolog [Source:UniProtKB/Swiss-Prot;Acc:Q9H410]
ENSG00000125975	C20orf173	ENST00000432650	protein_coding	34110888	34111087	-	34111007	80	-	Uncharacterized protein C20orf173 [Source:UniProtKB/Swiss-Prot;Acc:Q96LM9]
ENSG00000154479	C2orf77	ENST00000284676	protein_coding	170550732	1,71E+08	-	1,71E+08	93	-	Uncharacterized protein C2orf77 [Source:UniProtKB/Swiss-Prot;Acc:Q0VFZ6]
ENSG00000154479	C2orf77	ENST00000421028	protein_coding	170550661	1,71E+08	-	1,71E+08	22	-	Uncharacterized protein C2orf77 [Source:UniProtKB/Swiss-Prot;Acc:Q0VFZ6]
ENSG00000178811	C3orf53	ENST00000317800	protein_coding	28617619	28617818	-	28617774	44	-	Putative uncharacterized protein C3orf53 [Source:UniProtKB/Swiss-Prot;Acc:Q8N290]
ENSG00000111863	C6orf105	ENST00000229583	protein_coding	11779081	11779280	-	11779209	71	-	Uncharacterized protein C6orf105 [Source:UniProtKB/Swiss-Prot;Acc:Q96IZ2]
ENSG00000111863	C6orf105	ENST00000379413	protein_coding	11779081	11779280	-	11779209	71	-	Uncharacterized protein C6orf105 [Source:UniProtKB/Swiss-Prot;Acc:Q96IZ2]
ENSG00000111863	C6orf105	ENST00000414691	protein_coding	11779081	11779280	-	11779209	71	-	Uncharacterized protein C6orf105 [Source:UniProtKB/Swiss-Prot;Acc:Q96IZ2]
ENSG00000153956	CACNA2D1	ENST00000284088	protein_coding	82072607	82072806	-	82072719	87	-	Voltage-dependent calcium channel subunit alpha-2/delta-1 Precursor (Voltage-gated calcium channel subunit alpha-2/delta-1) [Contains Voltage-dependent calcium channel subunit alpha-2-1;Voltage-dependent calcium channel subunit delta-1] [Source:UniProtKB/Swiss-Prot;Acc:P54289]
ENSG00000198286	CARD11	ENST00000355508	protein_coding	2969495	2969694	-	2969647	47	-	Caspase recruitment domain-containing protein 11 (CARD-containing MAGUK protein 3)(Carma 1) [Source:UniProtKB/Swiss-Prot;Acc:Q9BXL7]
ENSG00000079112	CDH17	ENST00000450165	protein_coding	95229332	95229531	-	95229486	45	-	Cadherin-17 Precursor (Liver-intestine cadherin)(LI-cadherin)(Intestinal peptide-associated transporter HPT-1) [Source:UniProtKB/Swiss-Prot;Acc:Q12864]
ENSG00000136861	CDK5RAP2	ENST00000416449	protein_coding	123239433	1,23E+08	-	1,23E+08	97	-	CDK5 regulatory subunit-associated protein 2 (CDK5 activator-binding protein C48)(Centrosome-associated protein 215) [Source:UniProtKB/Swiss-Prot;Acc:Q96SN8]
ENSG00000093072	CECR1	ENST00000262607	protein_coding	17690580	17690779	-	17690709	70	-	Cat eye syndrome critical region protein 1 Precursor [Source:UniProtKB/Swiss-Prot;Acc:Q9NZK5]
ENSG00000159261	CLDN14	ENST00000399135	protein_coding	37852189	37852388	-	37852291	97	-	Claudin-14 [Source:UniProtKB/Swiss-Prot;Acc:O95500]
ENSG00000159261	CLDN14	ENST00000399137	protein_coding	37852189	37852388	-	37852291	97	-	Claudin-14 [Source:UniProtKB/Swiss-Prot;Acc:O95500]

ENSEMBL55 GeneID	Gene Symbol	ENSEMBL55 TranscriptID	Biotype	Gene start	Gene end	Strand	Motif start	TSS offset	Motif strand	Description
ENSG00000141551	CSNK1D	ENST00000403276	protein_coding	80231374	80231573	-	80231508	65	-	Casein kinase I isoform delta (CKI-delta)(CKId)(EC 2.7.11.1) [Source:UniProtKB/Swiss-Prot;Acc:P48730]
ENSG00000141551	CSNK1D	ENST00000392334	protein_coding	80231374	80231573	-	80231508	65	-	Casein kinase I isoform delta (CKI-delta)(CKId)(EC 2.7.11.1) [Source:UniProtKB/Swiss-Prot;Acc:P48730]
ENSG00000141551	CSNK1D	ENST00000314028	protein_coding	80231374	80231573	-	80231508	65	-	Casein kinase I isoform delta (CKI-delta)(CKId)(EC 2.7.11.1) [Source:UniProtKB/Swiss-Prot;Acc:P48730]
ENSG00000141551	CSNK1D	ENST00000269361	protein_coding	80231374	80231573	-	80231508	65	-	Casein kinase I isoform delta (CKI-delta)(CKId)(EC 2.7.11.1) [Source:UniProtKB/Swiss-Prot;Acc:P48730]
ENSG00000170959	DCDC5	ENST00000339794	protein_coding	30953252	30953451	-	30953353	98	-	Doublecortin domain-containing protein 5 [Source:UniProtKB/Swiss-Prot;Acc:Q6ZRR9]
ENSG00000170959	DCDC5	ENST00000406071	protein_coding	30953252	30953451	-	30953353	98	-	Doublecortin domain-containing protein 5 [Source:UniProtKB/Swiss-Prot;Acc:Q6ZRR9]
ENSG00000152457	DCLRE1C	ENST00000449101	protein_coding	14981691	14981890	-	14981854	36	-	Protein artemis (EC 3.1.-.-)(DNA cross-link repair 1C protein)(hSNM1C)(SNM1-like protein)(A-SCID protein) [Source:UniProtKB/Swiss-Prot;Acc:Q96SD1]
ENSG00000007968	E2F2	ENST00000361729	protein_coding	23857514	23857713	-	23857647	66	-	Transcription factor E2F2 (E2F-2) [Source:UniProtKB/Swiss-Prot;Acc:Q14209]
ENSG00000149016	EEF1G	ENST00000424909	protein_coding	62341136	62341335	-	62341255	80	-	Elongation factor 1-gamma (EF-1-gamma)(eEF-1B gamma) [Source:UniProtKB/Swiss-Prot;Acc:P26641]
ENSG00000149016	EEF1G	ENST00000422402	protein_coding	62341136	62341335	-	62341255	80	-	Elongation factor 1-gamma (EF-1-gamma)(eEF-1B gamma) [Source:UniProtKB/Swiss-Prot;Acc:P26641]
ENSG00000167658	EEF2	ENST00000445244	protein_coding	3985262	3985461	-	3985386	75	-	Elongation factor 2 (EF-2) [Source:UniProtKB/Swiss-Prot;Acc:P13639]
ENSG00000167658	EEF2	ENST00000309311	protein_coding	3985262	3985461	-	3985386	75	-	Elongation factor 2 (EF-2) [Source:UniProtKB/Swiss-Prot;Acc:P13639]
ENSG00000184349	EFNA5	ENST00000333274	protein_coding	107006397	1,07E+08	-	1,07E+08	78	-	Ephrin-A5 Precursor (EPH-related receptor tyrosine kinase ligand 7)(LERK-7)(AL-1) [Source:UniProtKB/Swiss-Prot;Acc:P52803]
ENSG00000100353	EIF3D	ENST00000412520	protein_coding	36924984	36925183	-	36925146	37	-	Eukaryotic translation initiation factor 3 subunit D (eIF3d)(Eukaryotic translation initiation factor 3 subunit 7)(eIF-3-zeta)(eIF3 p66) [Source:UniProtKB/Swiss-Prot;Acc:O15371]
ENSG00000100353	EIF3D	ENST00000455547	protein_coding	36925014	36925213	-	36925146	67	-	Eukaryotic translation initiation factor 3 subunit D (eIF3d)(Eukaryotic translation initiation factor 3 subunit 7)(eIF-3-zeta)(eIF3 p66) [Source:UniProtKB/Swiss-Prot;Acc:O15371]
ENSG00000100353	EIF3D	ENST00000432675	protein_coding	36925032	36925231	-	36925146	85	-	Eukaryotic translation initiation factor 3 subunit D (eIF3d)(Eukaryotic translation initiation factor 3 subunit 7)(eIF-3-zeta)(eIF3 p66) [Source:UniProtKB/Swiss-Prot;Acc:O15371]
ENSG00000100353	EIF3D	ENST00000397177	protein_coding	36924984	36925183	-	36925146	37	-	Eukaryotic translation initiation factor 3 subunit D (eIF3d)(Eukaryotic translation initiation factor 3 subunit 7)(eIF-3-zeta)(eIF3 p66) [Source:UniProtKB/Swiss-Prot;Acc:O15371]
ENSG00000075151	EIF4G3	ENST00000411888	protein_coding	21503151	21503350	-	21503308	42	-	Eukaryotic translation initiation factor 4 gamma 3 (eIF-4-gamma 3)(eIF-4G 3)(eIF4G 3)(eIF-4-gamma II)(eIF4GII) [Source:UniProtKB/Swiss-Prot;Acc:O43432]
ENSG00000075151	EIF4G3	ENST00000374927	protein_coding	21503141	21503340	-	21503308	32	-	Eukaryotic translation initiation factor 4 gamma 3 (eIF-4-gamma 3)(eIF-4G 3)(eIF4G 3)(eIF-4-gamma II)(eIF4GII) [Source:UniProtKB/Swiss-Prot;Acc:O43432]

ENSEMBL55 GeneID	Gene Symbol	ENSEMBL55 TranscriptID	Biotype	Gene start	Gene end	Strand	Motif start	TSS offset	Motif strand	Description
ENSG00000075151	EIF4G3	ENST00000400422	protein_coding	21503141	21503340	-	21503308	32	-	Eukaryotic translation initiation factor 4 gamma 3 (eIF-4-gamma 3)(eIF-4G 3)(eIF4G 3)(eIF-4-gamma II)(eIF4GII) [Source:UniProtKB/Swiss-Prot;Acc:O43432]
ENSG00000075151	EIF4G3	ENST00000374937	protein_coding	21503141	21503340	-	21503308	32	-	Eukaryotic translation initiation factor 4 gamma 3 (eIF-4-gamma 3)(eIF-4G 3)(eIF4G 3)(eIF-4-gamma II)(eIF4GII) [Source:UniProtKB/Swiss-Prot;Acc:O43432]
ENSG00000075151	EIF4G3	ENST00000438975	protein_coding	21503178	21503377	-	21503308	69	-	Eukaryotic translation initiation factor 4 gamma 3 (eIF-4-gamma 3)(eIF-4G 3)(eIF4G 3)(eIF-4-gamma II)(eIF4GII) [Source:UniProtKB/Swiss-Prot;Acc:O43432]
ENSG00000185104	FAF1	ENST00000371778	protein_coding	51425601	51425800	-	51425743	57	-	FAS-associated factor 1 (hFAF1)(UBX domain-containing protein 3A)(UBX domain-containing protein 12) [Source:UniProtKB/Swiss-Prot;Acc:Q9UNN5]
ENSG00000184083	FAM120C	ENST00000375180	protein_coding	54209515	54209714	-	54209673	41	-	Constitutive coactivator of PPAR-gamma-like protein 2 (Protein FAM120C)(Tumor antigen BJ-HCC-21) [Source:UniProtKB/Swiss-Prot;Acc:Q9NX05]
ENSG00000184083	FAM120C	ENST00000328235	protein_coding	54209515	54209714	-	54209673	41	-	Constitutive coactivator of PPAR-gamma-like protein 2 (Protein FAM120C)(Tumor antigen BJ-HCC-21) [Source:UniProtKB/Swiss-Prot;Acc:Q9NX05]
ENSG00000078579	FGF20	ENST00000180166	protein_coding	16859475	16859674	-	16859599	75	-	Fibroblast growth factor 20 (FGF-20) [Source:UniProtKB/Swiss-Prot;Acc:Q9NP95]
ENSG00000143520	FLG2	ENST00000388718	protein_coding	152332283	1,52E+08	-	1,52E+08	26	-	Filaggrin-2 (FLG-2)(Intermediate filament-associated and psoriasis-susceptibility protein)(Ifapsoriasin) [Source:UniProtKB/Swiss-Prot;Acc:Q5D862]
ENSG00000164946	FREM1	ENST00000380875	protein_coding	14910794	14910993	-	14910923	70	-	FRAS1-related extracellular matrix protein 1 Precursor (Protein QBRICK) [Source:UniProtKB/Swiss-Prot;Acc:Q5H8C1]
ENSG00000172159	FRMD3	ENST00000376434	protein_coding	85946677	85946876	-	85946817	59	-	FERM domain-containing protein 3 (Band 4.1-like protein 4O)(Ovary type protein 4.1)(4.1O) [Source:UniProtKB/Swiss-Prot;Acc:A2A2Y4]
ENSG00000165694	FRMD7	ENST00000370879	protein_coding	131228092	1,31E+08	-	1,31E+08	53	-	FERM domain-containing protein 7 [Source:UniProtKB/Swiss-Prot;Acc:Q6ZUT3]
ENSG00000128242	GAL3ST1	ENST00000447224	protein_coding	30956547	30956746	-	30956648	98	-	Galactosylceramide sulfotransferase (GalCer sulfotransferase)(EC 2.8.2.11)(Cerebroside sulfotransferase)(3'-phosphoadenylylsulfate:galactosylceramide 3'-sulfotransferase)(3'-phosphoadenosine-5'-phosphosulfate:GalCer sulfotransferase) [Source:UniProtKB/Swiss-Prot;Acc:Q99999]
ENSG00000128242	GAL3ST1	ENST00000437282	protein_coding	30956547	30956746	-	30956648	98	-	Galactosylceramide sulfotransferase (GalCer sulfotransferase)(EC 2.8.2.11)(Cerebroside sulfotransferase)(3'-phosphoadenylylsulfate:galactosylceramide 3'-sulfotransferase)(3'-phosphoadenosine-5'-phosphosulfate:GalCer sulfotransferase) [Source:UniProtKB/Swiss-Prot;Acc:Q99999]
ENSG00000118990	GLRXP3	ENST00000309178	protein_coding	161178642	1,61E+08	-	1,61E+08	63	-	Putative glutaredoxin-like protein [Source:UniProtKB/Swiss-Prot;Acc:A6NLA2]
ENSG00000198756	GLT25D2	ENST00000367520	protein_coding	183932998	1,84E+08	-	1,84E+08	47	-	Glycosyltransferase 25 family member 2 Precursor (EC 2.-.-.-) [Source:UniProtKB/Swiss-Prot;Acc:Q8IYK4]

ENSEMBL55 GeneID	Gene Symbol	ENSEMBL55 TranscriptID	Biotype	Gene start	Gene end	Strand	Motif start	TSS offset	Motif strand	Description
ENSG00000204628	GNB2L1	ENST00000441597	protein_coding	180670707	1,81E+08	-	1,81E+08	69	-	Guanine nucleotide-binding protein subunit beta-2-like 1 (Guanine nucleotide-binding protein subunit beta-like protein 12.3)(Receptor of activated protein kinase C 1)(RACK1)(Receptor for activated C kinase)(Cell proliferation-inducing gene 21 protein)(Human lung cancer oncogene 7 protein)(HLC-7) [Source:UniProtKB/Swiss-Prot;Acc:P63244]
ENSG00000204628	GNB2L1	ENST00000389599	protein_coding	180670707	1,81E+08	-	1,81E+08	69	-	Guanine nucleotide-binding protein subunit beta-2-like 1 (Guanine nucleotide-binding protein subunit beta-like protein 12.3)(Receptor of activated protein kinase C 1)(RACK1)(Receptor for activated C kinase)(Cell proliferation-inducing gene 21 protein)(Human lung cancer oncogene 7 protein)(HLC-7) [Source:UniProtKB/Swiss-Prot;Acc:P63244]
ENSG00000204628	GNB2L1	ENST00000376817	protein_coding	180670707	1,81E+08	-	1,81E+08	69	-	Guanine nucleotide-binding protein subunit beta-2-like 1 (Guanine nucleotide-binding protein subunit beta-like protein 12.3)(Receptor of activated protein kinase C 1)(RACK1)(Receptor for activated C kinase)(Cell proliferation-inducing gene 21 protein)(Human lung cancer oncogene 7 protein)(HLC-7) [Source:UniProtKB/Swiss-Prot;Acc:P63244]
ENSG00000204628	GNB2L1	ENST00000456394	protein_coding	180670707	1,81E+08	-	1,81E+08	69	-	Guanine nucleotide-binding protein subunit beta-2-like 1 (Guanine nucleotide-binding protein subunit beta-like protein 12.3)(Receptor of activated protein kinase C 1)(RACK1)(Receptor for activated C kinase)(Cell proliferation-inducing gene 21 protein)(Human lung cancer oncogene 7 protein)(HLC-7) [Source:UniProtKB/Swiss-Prot;Acc:P63244]
ENSG00000111305	GSG1	ENST00000324458	protein_coding	13256420	13256619	-	13256584	35	-	Germ cell-specific gene 1 protein [Source:UniProtKB/Swiss-Prot;Acc:Q2KHT4]
ENSG00000111305	GSG1	ENST00000457134	protein_coding	13256420	13256619	-	13256584	35	-	Germ cell-specific gene 1 protein [Source:UniProtKB/Swiss-Prot;Acc:Q2KHT4]
ENSG00000111305	GSG1	ENST00000351606	protein_coding	13256420	13256619	-	13256584	35	-	Germ cell-specific gene 1 protein [Source:UniProtKB/Swiss-Prot;Acc:Q2KHT4]
ENSG00000165259	HDX	ENST00000297977	protein_coding	83757262	83757461	-	83757416	45	-	Highly divergent homeobox [Source:UniProtKB/Swiss-Prot;Acc:Q7Z353]
ENSG00000165259	HDX	ENST00000373177	protein_coding	83757262	83757461	-	83757416	45	-	Highly divergent homeobox [Source:UniProtKB/Swiss-Prot;Acc:Q7Z353]
ENSG00000165259	HDX	ENST00000449553	protein_coding	83757259	83757458	-	83757416	42	-	Highly divergent homeobox [Source:UniProtKB/Swiss-Prot;Acc:Q7Z353]
ENSG00000203814	HIST2H2BF	ENST00000427880	protein_coding	149783689	1,5E+08	-	1,5E+08	29	-	Histone H2B type 2-F [Source:UniProtKB/Swiss-Prot;Acc:Q5QNW6]
ENSG00000203814	HIST2H2BF	ENST00000369167	protein_coding	149783715	1,5E+08	-	1,5E+08	55	-	Histone H2B type 2-F [Source:UniProtKB/Swiss-Prot;Acc:Q5QNW6]
ENSG00000205581	HMGN1	ENST00000431390	protein_coding	40720867	40721066	-	40721012	54	-	Non-histone chromosomal protein HMG-14 (High-mobility group nucleosome-binding domain-containing protein 1) [Source:UniProtKB/Swiss-Prot;Acc:P05114]
ENSG00000205581	HMGN1	ENST00000380749	protein_coding	40720848	40721047	-	40721012	35	-	Non-histone chromosomal protein HMG-14 (High-mobility group nucleosome-binding domain-containing protein 1) [Source:UniProtKB/Swiss-Prot;Acc:P05114]

ENSEMBL55 GeneID	Gene Symbol	ENSEMBL55 TranscriptID	Biotype	Gene start	Gene end	Strand	Motif start	TSS offset	Motif strand	Description
ENSG00000140749	IGSF6	ENST00000268389	protein_coding	21663730	21663929	-	21663883	46	-	Immunoglobulin superfamily member 6 Precursor (Protein DORA) [Source:UniProtKB/Swiss-Prot;Acc:O95976]
ENSG00000140749	IGSF6	ENST00000445158	protein_coding	21663730	21663929	-	21663883	46	-	Immunoglobulin superfamily member 6 Precursor (Protein DORA) [Source:UniProtKB/Swiss-Prot;Acc:O95976]
ENSG00000030419	IKZF2	ENST00000442445	protein_coding	214014766	2,14E+08	-	2,14E+08	80	-	Zinc finger protein Helios (Ikaros family zinc finger protein 2) [Source:UniProtKB/Swiss-Prot;Acc:Q9UKS7]
ENSG00000112706	IMPG1	ENST00000369950	protein_coding	76782136	76782335	-	76782301	34	-	Interphotoreceptor matrix proteoglycan 1 Precursor (Interphotoreceptor matrix proteoglycan of 150 kDa)(IPM-150)(Sialoprotein associated with cones and rods) [Source:UniProtKB/Swiss-Prot;Acc:Q17R60]
ENSG00000112706	IMPG1	ENST00000369963	protein_coding	76782136	76782335	-	76782308	27	-	Interphotoreceptor matrix proteoglycan 1 Precursor (Interphotoreceptor matrix proteoglycan of 150 kDa)(IPM-150)(Sialoprotein associated with cones and rods) [Source:UniProtKB/Swiss-Prot;Acc:Q17R60]
ENSG00000112706	IMPG1	ENST00000369963	protein_coding	76782136	76782335	-	76782301	34	-	Interphotoreceptor matrix proteoglycan 1 Precursor (Interphotoreceptor matrix proteoglycan of 150 kDa)(IPM-150)(Sialoprotein associated with cones and rods) [Source:UniProtKB/Swiss-Prot;Acc:Q17R60]
ENSG00000112706	IMPG1	ENST00000369950	protein_coding	76782136	76782335	-	76782308	27	-	Interphotoreceptor matrix proteoglycan 1 Precursor (Interphotoreceptor matrix proteoglycan of 150 kDa)(IPM-150)(Sialoprotein associated with cones and rods) [Source:UniProtKB/Swiss-Prot;Acc:Q17R60]
ENSG00000197429	IPP	ENST00000396474	protein_coding	46211934	46212133	-	46212084	49	-	Actin-binding protein IPP (MIPP protein)(Kelch-like protein 27) [Source:UniProtKB/Swiss-Prot;Acc:Q9Y573]
ENSG00000123243	ITIH5	ENST00000305368	protein_coding	7620130	7620329	-	7620278	51	-	Inter-alpha-trypsin inhibitor heavy chain H5 Precursor (Inter-alpha-inhibitor heavy chain 5)(ITI heavy chain H5) [Source:UniProtKB/Swiss-Prot;Acc:Q86UX2]
ENSG00000121361	KCNJ8	ENST00000240662	protein_coding	21927548	21927747	-	21927687	60	-	ATP-sensitive inward rectifier potassium channel 8 (Potassium channel, inwardly rectifying subfamily J member 8)(Inwardly rectifier K(+) channel Kir6.1)(uKATP-1) [Source:UniProtKB/Swiss-Prot;Acc:Q15842]
ENSG00000198920	KIAA0753	ENST00000361413	protein_coding	6544048	6544247	-	6544160	87	-	Uncharacterized protein KIAA0753 [Source:UniProtKB/Swiss-Prot;Acc:Q2KHM9]
ENSG00000163807	KIAA1143	ENST00000296121	protein_coding	44802974	44803173	-	44803134	39	-	Uncharacterized protein KIAA1143 [Source:UniProtKB/Swiss-Prot;Acc:Q96AT1]
ENSG00000124743	KLHL31	ENST00000370905	protein_coding	53530307	53530506	-	53530468	38	-	Kelch-like protein 31 (Kelch repeat and BTB domain-containing protein 1)(Kelch-like protein KLHL)(BTB and kelch domain-containing protein 6) [Source:UniProtKB/Swiss-Prot;Acc:Q9H511]
ENSG00000169035	KLK7	ENST00000304045	protein_coding	51486951	51487150	-	51487115	35	-	Kallikrein-7 Precursor (hK7)(EC 3.4.21.117)(Stratum corneum chymotryptic enzyme)(hSCCE)(Serine protease 6) [Source:UniProtKB/Swiss-Prot;Acc:P49862]
ENSG00000186393	KRT26	ENST00000335552	protein_coding	38928212	38928411	-	38928330	81	-	Keratin, type I cytoskeletal 26 (Cytokeratin-26)(CK-26)(Keratin-26)(K26)(Type I inner root sheath-specific keratin-K25irs2)(Keratin 25B) [Source:UniProtKB/Swiss-Prot;Acc:Q7Z3Y9]

ENSEMBL55 GeneID	Gene Symbol	ENSEMBL55 TranscriptID	Biotype	Gene start	Gene end	Strand	Motif start	TSS offset	Motif strand	Description
ENSG00000205420	KRT6A	ENST00000452121	protein_coding	52886773	52886972	-	52886949	23	-	Keratin, type II cytoskeletal 6A (Cytokeratin-6A)(CK 6A)(K6a keratin)(Cytokeratin-6D)(CK 6D)(Allergen Hom s 5) [Source:UniProtKB/Swiss-Prot;Acc:P02538]
ENSG00000185479	KRT6B	ENST00000252252	protein_coding	52845711	52845910	-	52845839	71	-	Keratin, type II cytoskeletal 6B (Cytokeratin-6B)(CK 6B)(K6b keratin) [Source:UniProtKB/Swiss-Prot;Acc:P04259]
ENSG00000170465	KRT6C	ENST00000411979	protein_coding	52867322	52867521	-	52867498	23	-	Keratin, type II cytoskeletal 6C (Cytokeratin-6C)(CK 6C)(K6c keratin)(Cytokeratin-6E)(CK 6E)(Keratin K6h) [Source:UniProtKB/Swiss-Prot;Acc:P48668]
ENSG00000170465	KRT6C	ENST00000252250	protein_coding	52867370	52867569	-	52867498	71	-	Keratin, type II cytoskeletal 6C (Cytokeratin-6C)(CK 6C)(K6c keratin)(Cytokeratin-6E)(CK 6E)(Keratin K6h) [Source:UniProtKB/Swiss-Prot;Acc:P48668]
ENSG00000170523	KRT83	ENST00000293670	protein_coding	52714983	52715182	-	52715154	28	-	Keratin, type II cuticular Hb3 (Type II hair keratin Hb3)(Keratin-83)(K83)(K2.10) [Source:UniProtKB/Swiss-Prot;Acc:P78385]
ENSG00000204571	KRTAP5-11	ENST00000398530	protein_coding	71293722	71293921	-	71293884	37	-	Keratin-associated protein 5-11 (Keratin-associated protein 5.11)(Ultrahigh sulfur keratin-associated protein 5.11) [Source:UniProtKB/Swiss-Prot;Acc:Q6L8G4]
ENSG00000115318	LOXL3	ENST00000409549	protein_coding	74780012	74780211	-	74780174	37	-	Lysyl oxidase homolog 3 Precursor (EC 1.4.3.-)(Lysyl oxidase-like protein 3) [Source:UniProtKB/Swiss-Prot;Acc:P58215]
ENSG00000168702	LRP1B	ENST00000437977	protein_coding	141115438	1,41E+08	-	1,41E+08	29	-	Low-density lipoprotein receptor-related protein 1B Precursor (Low-density lipoprotein receptor-related protein-deleted in tumor)(LRP-DIT) [Source:UniProtKB/Swiss-Prot;Acc:Q9NZR2]
ENSG00000166897	LRRC62	ENST00000451509	protein_coding	37822843	37823042	-	37822973	69	-	Leucine-rich repeat and fibronectin type-III domain-containing protein 6 Precursor (Extracellular leucine-rich repeat and fibronectin type III domain-containing protein 2)(Leucine-rich repeat-containing protein 62) [Source:UniProtKB/Swiss-Prot;Acc:Q5R3F8]
ENSG00000162620	LRRIQ3	ENST00000444984	protein_coding	74648996	74649195	-	74649134	61	-	Leucine-rich repeat and IQ domain-containing protein 3 (Leucine-rich repeat-containing protein 44) [Source:UniProtKB/Swiss-Prot;Acc:A6PVS8]
ENSG00000163155	LYSMD1	ENST00000368908	protein_coding	151138171	1,51E+08	-	1,51E+08	48	-	LysM and putative peptidoglycan-binding domain-containing protein 1 [Source:UniProtKB/Swiss-Prot;Acc:Q96S90]
ENSG00000163818	LZTFL1	ENST00000440576	protein_coding	45877080	45877279	-	45877183	96	-	Leucine zipper transcription factor-like protein 1 [Source:UniProtKB/Swiss-Prot;Acc:Q9NQ48]
ENSG00000178573	MAF	ENST00000326043	protein_coding	79634423	79634622	-	79634599	23	-	Transcription factor Maf (Proto-oncogene c-maf)(C-Maf)(V-maf musculoaponeurotic fibrosarcoma oncogene homolog) [Source:UniProtKB/Swiss-Prot;Acc:O75444]
ENSG00000153721	MAGI1	ENST00000433165	protein_coding	154755008	1,55E+08	-	1,55E+08	68	-	Connector enhancer of kinase suppressor of ras 3 (CNKSR family member 3)(Membrane-associated guanylate kinase-interacting protein-like 1)(Maguin-like protein) [Source:UniProtKB/Swiss-Prot;Acc:Q6P9H4]
ENSG00000153721	MAGI1	ENST00000454664	protein_coding	154755008	1,55E+08	-	1,55E+08	68	-	Connector enhancer of kinase suppressor of ras 3 (CNKSR family member 3)(Membrane-associated guanylate kinase-interacting protein-like 1)(Maguin-like protein) [Source:UniProtKB/Swiss-Prot;Acc:Q6P9H4]
ENSG00000109323	MANBA	ENST00000226578	protein_coding	103681952	1,04E+08	-	1,04E+08	78	-	Beta-mannosidase Precursor (EC 3.2.1.25)(Lysosomal beta A mannosidase)(Mannanase)(Mannase) [Source:UniProtKB/Swiss-Prot;Acc:O00462]

ENSEMBL55 GeneID	Gene Symbol	ENSEMBL55 TranscriptID	Biotype	Gene start	Gene end	Strand	Motif start	TSS offset	Motif strand	Description
ENSG00000197971	MBP	ENST00000447114	protein_coding	74721879	74722078	-	74722054	24	-	Myelin basic protein (MBP)(Myelin A1 protein)(Myelin membrane encephalitogenic protein) [Source:UniProtKB/Swiss-Prot;Acc:P02686]
ENSG00000185231	MC2R	ENST00000399821	protein_coding	13885448	13885647	-	13885580	67	-	Adrenocorticotropic hormone receptor (ACTH receptor)(ACTH-R)(Adrenocorticotropin receptor)(Melanocortin receptor 2)(MC2-R) [Source:UniProtKB/Swiss-Prot;Acc:Q01718]
ENSG00000139915	MDGA2	ENST00000357362	protein_coding	47812122	47812321	-	47812263	58	-	MAM domain-containing glycosylphosphatidylinositol anchor protein 2 Precursor (MAM domain-containing protein 1) [Source:UniProtKB/Swiss-Prot;Acc:Q7Z553]
ENSG00000123562	MORF4L2	ENST00000340782	protein_coding	102933382	1,03E+08	-	1,03E+08	95	-	Mortality factor 4-like protein 2 (MORF-related gene X protein)(Transcription factor-like protein MRGX)(MSL3-2 protein) [Source:UniProtKB/Swiss-Prot;Acc:Q15014]
ENSG00000110077	MS4A6A	ENST00000420732	protein_coding	59950475	59950674	-	59950600	74	-	Membrane-spanning 4-domains subfamily A member 6A (Four-span transmembrane protein 3)(CD20 antigen-like 3) [Source:UniProtKB/Swiss-Prot;Acc:Q9H2W1]
ENSG00000110077	MS4A6A	ENST00000422075	protein_coding	59950475	59950674	-	59950600	74	-	Membrane-spanning 4-domains subfamily A member 6A (Four-span transmembrane protein 3)(CD20 antigen-like 3) [Source:UniProtKB/Swiss-Prot;Acc:Q9H2W1]
ENSG00000110077	MS4A6A	ENST00000412309	protein_coding	59950475	59950674	-	59950600	74	-	Membrane-spanning 4-domains subfamily A member 6A (Four-span transmembrane protein 3)(CD20 antigen-like 3) [Source:UniProtKB/Swiss-Prot;Acc:Q9H2W1]
ENSG00000110077	MS4A6A	ENST00000426738	protein_coding	59950475	59950674	-	59950600	74	-	Membrane-spanning 4-domains subfamily A member 6A (Four-span transmembrane protein 3)(CD20 antigen-like 3) [Source:UniProtKB/Swiss-Prot;Acc:Q9H2W1]
ENSG00000110077	MS4A6A	ENST00000323961	protein_coding	59950475	59950674	-	59950600	74	-	Membrane-spanning 4-domains subfamily A member 6A (Four-span transmembrane protein 3)(CD20 antigen-like 3) [Source:UniProtKB/Swiss-Prot;Acc:Q9H2W1]
ENSG00000196531	NACA	ENST00000435567	protein_coding	57118867	57119066	-	57119006	60	-	Nascent polypeptide-associated complex subunit alpha (NAC-alpha)(Alpha-NAC)(Allergen Hom s 2) [Source:UniProtKB/Swiss-Prot;Acc:Q13765]
ENSG00000196531	NACA	ENST00000356769	protein_coding	57118884	57119083	-	57119006	77	-	Nascent polypeptide-associated complex subunit alpha (NAC-alpha)(Alpha-NAC)(Allergen Hom s 2) [Source:UniProtKB/Swiss-Prot;Acc:Q13765]
ENSG00000196531	NACA	ENST00000393891	protein_coding	57118867	57119066	-	57119006	60	-	Nascent polypeptide-associated complex subunit alpha (NAC-alpha)(Alpha-NAC)(Allergen Hom s 2) [Source:UniProtKB/Swiss-Prot;Acc:Q13765]
ENSG00000196531	NACA	ENST00000450688	protein_coding	57118884	57119083	-	57119006	77	-	Nascent polypeptide-associated complex subunit alpha (NAC-alpha)(Alpha-NAC)(Allergen Hom s 2) [Source:UniProtKB/Swiss-Prot;Acc:Q13765]
ENSG00000122497	NBPF14	ENST00000448574	protein_coding	148025664	1,48E+08	-	1,48E+08	30	-	Neuroblastoma breakpoint family member 14 [Source:UniProtKB/Swiss-Prot;Acc:Q5TI25]
ENSG00000122497	NBPF14	ENST00000392972	protein_coding	148025664	1,48E+08	-	1,48E+08	30	-	Neuroblastoma breakpoint family member 14 [Source:UniProtKB/Swiss-Prot;Acc:Q5TI25]
ENSG00000122497	NBPF14	ENST00000436356	protein_coding	148025664	1,48E+08	-	1,48E+08	30	-	Neuroblastoma breakpoint family member 14 [Source:UniProtKB/Swiss-Prot;Acc:Q5TI25]
ENSG00000122497	NBPF14	ENST00000415551	protein_coding	148025662	1,48E+08	-	1,48E+08	28	-	Neuroblastoma breakpoint family member 14 [Source:UniProtKB/Swiss-Prot;Acc:Q5TI25]

ENSEMBL55 GeneID	Gene Symbol	ENSEMBL55 TranscriptID	Biotype	Gene start	Gene end	Strand	Motif start	TSS offset	Motif strand	Description
ENSG00000122497	NBPF14	ENST00000426874	protein_coding	148025664	1,48E+08	-	1,48E+08	30	-	Neuroblastoma breakpoint family member 14 [Source:UniProtKB/Swiss-Prot;Acc:Q5TI25]
ENSG00000122497	NBPF14	ENST00000431121	protein_coding	148025664	1,48E+08	-	1,48E+08	30	-	Neuroblastoma breakpoint family member 14 [Source:UniProtKB/Swiss-Prot;Acc:Q5TI25]
ENSG00000122497	NBPF14	ENST00000444640	protein_coding	148025664	1,48E+08	-	1,48E+08	30	-	Neuroblastoma breakpoint family member 14 [Source:UniProtKB/Swiss-Prot;Acc:Q5TI25]
ENSG00000122497	NBPF14	ENST00000458135	protein_coding	148025664	1,48E+08	-	1,48E+08	30	-	Neuroblastoma breakpoint family member 14 [Source:UniProtKB/Swiss-Prot;Acc:Q5TI25]
ENSG00000146938	NLGN4X	ENST00000381095	protein_coding	6146507	6146706	-	6146664	42	-	Neuroligin-4, X-linked Precursor (Neuroligin X)(HNLX) [Source:UniProtKB/Swiss-Prot;Acc:Q8N0W4]
ENSG00000105245	NUMBL	ENST00000252891	protein_coding	41196357	41196556	-	41196508	48	-	Numb-like protein (Numb-R) [Source:UniProtKB/Swiss-Prot;Acc:Q9Y6R0]
ENSG00000197023	OR51A6P	ENST00000417991	protein_coding	4911627	4911826	-	4911728	98	-	
ENSG00000197023	OR51A6P	ENST00000418680	protein_coding	4911627	4911826	-	4911728	98	-	
ENSG00000197023	OR51A6P	ENST00000355607	protein_coding	4911627	4911826	-	4911728	98	-	
ENSG00000197023	OR51A6P	ENST00000418847	protein_coding	4911627	4911826	-	4911728	98	-	
ENSG00000112462	OR5V1	ENST00000377154	protein_coding	29399545	29399744	-	29399689	55	-	Olfactory receptor 5V1 (Olfactory receptor OR6-26)(Hs6M1-21) [Source:UniProtKB/Swiss-Prot;Acc:Q9UGF6]
ENSG00000171540	OTP	ENST00000306422	protein_coding	76934323	76934522	-	76934468	54	-	Homeobox protein orthopedia [Source:UniProtKB/Swiss-Prot;Acc:Q5XKR4]
ENSG00000070756	PABPCP5	ENST00000347137	protein_coding	101734116	1,02E+08	-	1,02E+08	99	-	Polyadenylate-binding protein 1 (Poly(A)-binding protein 1)(PABP 1) [Source:UniProtKB/Swiss-Prot;Acc:P11940]
ENSG00000070756	PABPCP5	ENST00000318607	protein_coding	101734116	1,02E+08	-	1,02E+08	99	-	Polyadenylate-binding protein 1 (Poly(A)-binding protein 1)(PABP 1) [Source:UniProtKB/Swiss-Prot;Acc:P11940]
ENSG00000102096	PIM2	ENST00000442430	protein_coding	48772813	48773012	-	48772979	33	-	Serine/threonine-protein kinase Pim-2 (EC 2.7.11.1)(Pim-2h) [Source:UniProtKB/Swiss-Prot;Acc:Q9P1W9]
ENSG00000186088	PION	ENST00000449779	protein_coding	76984748	76984947	-	76984884	63	-	Protein pigeon homolog [Source:UniProtKB/Swiss-Prot;Acc:A4D1B5]
ENSG00000183530	PISD	ENST00000451635	protein_coding	32034289	32034488	-	32034424	64	-	Phosphatidylserine decarboxylase proenzyme (EC 4.1.1.65) [Contains Phosphatidylserine decarboxylase alpha chain;Phosphatidylserine decarboxylase beta chain] [Source:UniProtKB/Swiss-Prot;Acc:Q9UG56]
ENSG00000143850	PLEKHA6	ENST00000272203	protein_coding	204328845	2,04E+08	-	2,04E+08	88	-	Pleckstrin homology domain-containing family A member 6 (Phosphoinositol 3-phosphate-binding protein 3)(PEPP-3) [Source:UniProtKB/Swiss-Prot;Acc:Q9Y2H5]
ENSG00000143850	PLEKHA6	ENST00000414478	protein_coding	204328845	2,04E+08	-	2,04E+08	88	-	Pleckstrin homology domain-containing family A member 6 (Phosphoinositol 3-phosphate-binding protein 3)(PEPP-3) [Source:UniProtKB/Swiss-Prot;Acc:Q9Y2H5]
ENSG00000106397	PLOD3	ENST00000456079	protein_coding	100861502	1,01E+08	-	1,01E+08	61	-	Procollagen-lysine,2-oxoglutarate 5-dioxygenase 3 Precursor (EC 1.14.11.4)(Lysyl hydroxylase 3)(LH3) [Source:UniProtKB/Swiss-Prot;Acc:O60568]
ENSG00000104881	PPP1R13L	ENST00000416875	protein_coding	45908113	45908312	-	45908258	54	-	RelA-associated inhibitor (Inhibitor of ASPP protein)(Protein iASPP)(PPP1R13B-like protein)(NFkB-interacting protein 1) [Source:UniProtKB/Swiss-Prot;Acc:Q8WUF5]
ENSG00000104881	PPP1R13L	ENST00000360957	protein_coding	45908113	45908312	-	45908258	54	-	RelA-associated inhibitor (Inhibitor of ASPP protein)(Protein

ENSEMBL55 GeneID	Gene Symbol	ENSEMBL55 TranscriptID	Biotype	Gene start	Gene end	Strand	Motif start	TSS offset	Motif strand	Description
										iASPP)(PPP1R13B-like protein)(NFkB-interacting protein 1) [Source:UniProtKB/Swiss-Prot;Acc:Q8WUF5]
ENSG00000074211	PPP2R2C	ENST00000382597	protein_coding	6384944	6385143	-	6385057	86	-	Serine/threonine-protein phosphatase 2A 55 kDa regulatory subunit B gamma isoform (PP2A, subunit B, B-gamma isoform)(PP2A, subunit B, B55-gamma isoform)(PP2A, subunit B, PR55-gamma isoform)(PP2A, subunit B, R2-gamma isoform)(IMYPNO1) [Source:UniProtKB/Swiss-Prot;Acc:Q9Y2T4]
ENSG00000196998	PRAF2	ENST00000433252	protein_coding	48933202	48933401	-	48933355	46	-	PRA1 family protein 2 [Source:UniProtKB/Swiss-Prot;Acc:O60831]
ENSG00000115825	PRKD3	ENST00000379066	protein_coding	37544838	37545037	-	37544987	50	-	Serine/threonine-protein kinase D3 (EC 2.7.11.13)(Protein kinase C nu type)(nPKC-nu)(Protein kinase EPK2) [Source:UniProtKB/Swiss-Prot;Acc:O94806]
ENSG00000106772	PRUNE2	ENST00000441554	protein_coding	79320198	79320397	-	79320369	28	-	Protein prune homolog 2 [Source:UniProtKB/Swiss-Prot;Acc:Q8WUY3]
ENSG00000106772	PRUNE2	ENST00000223609	protein_coding	79320198	79320397	-	79320369	28	-	Protein prune homolog 2 [Source:UniProtKB/Swiss-Prot;Acc:Q8WUY3]
ENSG00000115165	PSCDBP	ENST00000435117	protein_coding	158345142	1,58E+08	-	1,58E+08	88	-	Cytohesin-interacting protein (Cytohesin-binding protein HE)(Cytohesin binder and regulator)(CYBR)(Cytohesin-associated scaffolding protein)(CASP)(Pleckstrin homology Sec7 and coiled-coil domains-binding protein) [Source:UniProtKB/Swiss-Prot;Acc:O60759]
ENSG00000068878	PSME4	ENST00000421748	protein_coding	54137008	54137207	-	54137185	22	-	Proteasome activator complex subunit 4 (Proteasome activator PA200) [Source:UniProtKB/Swiss-Prot;Acc:Q14997]
ENSG00000134644	PUM1	ENST00000373747	protein_coding	31538365	31538564	-	31538477	87	-	Pumilio homolog 1 (Pumilio-1)(HsPUM) [Source:UniProtKB/Swiss-Prot;Acc:Q14671]
ENSG00000134644	PUM1	ENST00000440538	protein_coding	31538365	31538564	-	31538477	87	-	Pumilio homolog 1 (Pumilio-1)(HsPUM) [Source:UniProtKB/Swiss-Prot;Acc:Q14671]
ENSG00000134644	PUM1	ENST00000426105	protein_coding	31538365	31538564	-	31538477	87	-	Pumilio homolog 1 (Pumilio-1)(HsPUM) [Source:UniProtKB/Swiss-Prot;Acc:Q14671]
ENSG00000134644	PUM1	ENST00000423018	protein_coding	31538365	31538564	-	31538477	87	-	Pumilio homolog 1 (Pumilio-1)(HsPUM) [Source:UniProtKB/Swiss-Prot;Acc:Q14671]
ENSG00000134644	PUM1	ENST00000257075	protein_coding	31538365	31538564	-	31538477	87	-	Pumilio homolog 1 (Pumilio-1)(HsPUM) [Source:UniProtKB/Swiss-Prot;Acc:Q14671]
ENSG00000134644	PUM1	ENST00000373742	protein_coding	31538343	31538542	-	31538477	65	-	Pumilio homolog 1 (Pumilio-1)(HsPUM) [Source:UniProtKB/Swiss-Prot;Acc:Q14671]
ENSG00000185379	RAD51L3	ENST00000345766	protein_coding	33446689	33446888	-	33446819	69	-	DNA repair protein RAD51 homolog 4 (R51H3)(RAD51-like protein 3)(TRAD) [Source:UniProtKB/Swiss-Prot;Acc:O75771]
ENSG00000185379	RAD51L3	ENST00000339934	protein_coding	33446689	33446888	-	33446819	69	-	DNA repair protein RAD51 homolog 4 (R51H3)(RAD51-like protein 3)(TRAD) [Source:UniProtKB/Swiss-Prot;Acc:O75771]
ENSG00000185379	RAD51L3	ENST00000360276	protein_coding	33446689	33446888	-	33446819	69	-	DNA repair protein RAD51 homolog 4 (R51H3)(RAD51-like protein 3)(TRAD) [Source:UniProtKB/Swiss-Prot;Acc:O75771]
ENSG00000185379	RAD51L3	ENST00000335858	protein_coding	33446689	33446888	-	33446819	69	-	DNA repair protein RAD51 homolog 4 (R51H3)(RAD51-like protein 3)(TRAD) [Source:UniProtKB/Swiss-Prot;Acc:O75771]
ENSG00000185379	RAD51L3	ENST00000394589	protein_coding	33446689	33446888	-	33446819	69	-	DNA repair protein RAD51 homolog 4 (R51H3)(RAD51-like protein 3)(TRAD) [Source:UniProtKB/Swiss-Prot;Acc:O75771]

ENSEMBL55 GeneID	Gene Symbol	ENSEMBL55 TranscriptID	Biotype	Gene start	Gene end	Strand	Motif start	TSS offset	Motif strand	Description
ENSG00000185379	RAD51L3	ENST00000345365	protein_coding	33446689	33446888	-	33446819	69	-	DNA repair protein RAD51 homolog 4 (R51H3)(RAD51-like protein 3)(TRAD) [Source:UniProtKB/Swiss-Prot;Acc:O75771]
ENSG00000185379	RAD51L3	ENST00000357906	protein_coding	33446689	33446888	-	33446819	69	-	DNA repair protein RAD51 homolog 4 (R51H3)(RAD51-like protein 3)(TRAD) [Source:UniProtKB/Swiss-Prot;Acc:O75771]
ENSG00000169800	RBMY1F	ENST00000455210	protein_coding	21679376	21679575	-	21679487	88	-	RNA-binding motif protein, Y chromosome, family 1 member F/J (Y chromosome RNA recognition motif 2) [Source:UniProtKB/Swiss-Prot;Acc:Q15415]
ENSG00000169800	RBMY1F	ENST00000454945	protein_coding	21679376	21679575	-	21679487	88	-	RNA-binding motif protein, Y chromosome, family 1 member F/J (Y chromosome RNA recognition motif 2) [Source:UniProtKB/Swiss-Prot;Acc:Q15415]
ENSG00000169800	RBMY1F	ENST00000303766	protein_coding	21679376	21679575	-	21679487	88	-	RNA-binding motif protein, Y chromosome, family 1 member F/J (Y chromosome RNA recognition motif 2) [Source:UniProtKB/Swiss-Prot;Acc:Q15415]
ENSG00000169800	RBMY1F	ENST00000454978	protein_coding	21679376	21679575	-	21679487	88	-	RNA-binding motif protein, Y chromosome, family 1 member F/J (Y chromosome RNA recognition motif 2) [Source:UniProtKB/Swiss-Prot;Acc:Q15415]
ENSG00000172348	RCAN2	ENST00000405162	protein_coding	46424514	46424713	-	46424690	23	-	Calcipressin-2 (Regulator of calcineurin 2)(Down syndrome candidate region 1-like 1)(Thyroid hormone-responsive protein ZAKI-4)(Myocyte-enriched calcineurin-interacting protein 2)(MCIP2) [Source:UniProtKB/Swiss-Prot;Acc:Q14206]
ENSG00000009413	REV3L	ENST00000229465	protein_coding	111636641	1,12E+08	-	1,12E+08	28	-	DNA polymerase zeta catalytic subunit (hREV3)(EC 2.7.7.7) [Source:UniProtKB/Swiss-Prot;Acc:O60673]
ENSG00000183054	RGPD6	ENST00000330331	protein_coding	111335409	1,11E+08	-	1,11E+08	31	-	RANBP2-like and GRIP domain-containing protein 5 (Ran-binding protein 2-like 1)(RanBP2L1)(Sperm membrane protein BS-63) [Source:UniProtKB/Swiss-Prot;Acc:Q99666]
ENSG00000183054	RGPD6	ENST00000329516	protein_coding	111335409	1,11E+08	-	1,11E+08	31	-	RANBP2-like and GRIP domain-containing protein 5 (Ran-binding protein 2-like 1)(RanBP2L1)(Sperm membrane protein BS-63) [Source:UniProtKB/Swiss-Prot;Acc:Q99666]
ENSG00000205937	RNPS1	ENST00000320225	protein_coding	2317915	2318114	-	2318028	86	-	RNA-binding protein with serine-rich domain 1 (SR-related protein LDC2) [Source:UniProtKB/Swiss-Prot;Acc:Q15287]
ENSG00000134318	ROCK2	ENST00000401753	protein_coding	11374597	11374796	-	11374767	29	-	Rho-associated protein kinase 2 (EC 2.7.11.1)(Rho-associated, coiled-coil-containing protein kinase 2)(p164 ROCK-2)(Rho kinase 2) [Source:UniProtKB/Swiss-Prot;Acc:O75116]
ENSG00000069667	RORA	ENST00000335670	protein_coding	61521319	61521518	-	61521436	82	-	Nuclear receptor ROR-alpha (Retinoid-related orphan receptor-alpha)(Nuclear receptor RZR-alpha)(Nuclear receptor subfamily 1 group F member 1) [Source:UniProtKB/Swiss-Prot;Acc:P35398]
ENSG00000196756	RP4-564F22.2	ENST00000436764	protein_coding	37063777	37063976	-	37063898	78	-	hypothetical LOC388796 (LOC388796), transcript variant 2, non-coding RNA [Source:RefSeq DNA;Acc:NR_027241]
ENSG00000196756	RP4-564F22.2	ENST00000424235	protein_coding	37063778	37063977	-	37063898	79	-	hypothetical LOC388796 (LOC388796), transcript variant 2, non-coding RNA [Source:RefSeq DNA;Acc:NR_027241]
ENSG00000196756	RP4-564F22.2	ENST00000424235	protein_coding	37063778	37063977	-	37063908	69	-	hypothetical LOC388796 (LOC388796), transcript variant 2, non-coding RNA [Source:RefSeq DNA;Acc:NR_027241]
ENSG00000196756	RP4-564F22.2	ENST00000456953	protein_coding	37063777	37063976	-	37063908	68	-	hypothetical LOC388796 (LOC388796), transcript variant 2, non-coding RNA [Source:RefSeq DNA;Acc:NR_027241]
ENSG00000196756	RP4-564F22.2	ENST00000456953	protein_coding	37063777	37063976	-	37063898	78	-	hypothetical LOC388796 (LOC388796), transcript variant 2, non-coding RNA [Source:RefSeq DNA;Acc:NR_027241]

ENSEMBL55 GeneID	Gene Symbol	ENSEMBL55 TranscriptID	Biotype	Gene start	Gene end	Strand	Motif start	TSS offset	Motif strand	Description
ENSG00000196756	RP4-564F22.2	ENST00000417578	protein_coding	37063721	37063920	-	37063898	22	-	hypothetical LOC388796 (LOC388796), transcript variant 2, non-coding RNA [Source:RefSeq DNA;Acc:NR_027241]
ENSG00000196756	RP4-564F22.2	ENST00000413755	protein_coding	37063797	37063996	-	37063898	98	-	hypothetical LOC388796 (LOC388796), transcript variant 2, non-coding RNA [Source:RefSeq DNA;Acc:NR_027241]
ENSG00000196756	RP4-564F22.2	ENST00000413755	protein_coding	37063797	37063996	-	37063908	88	-	hypothetical LOC388796 (LOC388796), transcript variant 2, non-coding RNA [Source:RefSeq DNA;Acc:NR_027241]
ENSG00000196756	RP4-564F22.2	ENST00000440508	protein_coding	37063785	37063984	-	37063898	86	-	hypothetical LOC388796 (LOC388796), transcript variant 2, non-coding RNA [Source:RefSeq DNA;Acc:NR_027241]
ENSG00000196756	RP4-564F22.2	ENST00000436764	protein_coding	37063777	37063976	-	37063908	68	-	hypothetical LOC388796 (LOC388796), transcript variant 2, non-coding RNA [Source:RefSeq DNA;Acc:NR_027241]
ENSG00000196756	RP4-564F22.2	ENST00000440508	protein_coding	37063785	37063984	-	37063908	76	-	hypothetical LOC388796 (LOC388796), transcript variant 2, non-coding RNA [Source:RefSeq DNA;Acc:NR_027241]
ENSG00000197958	RPL12	ENST00000361436	protein_coding	130213485	1,3E+08	-	1,3E+08	67	-	60S ribosomal protein L12 [Source:UniProtKB/Swiss-Prot;Acc:P30050]
ENSG00000063177	RPL18P13	ENST00000084795	protein_coding	49122234	49122433	-	49122352	81	-	60S ribosomal protein L18 [Source:UniProtKB/Swiss-Prot;Acc:Q07020]
ENSG00000063177	RPL18P13	ENST00000450952	protein_coding	49122234	49122433	-	49122352	81	-	60S ribosomal protein L18 [Source:UniProtKB/Swiss-Prot;Acc:Q07020]
ENSG00000125691	RPL23	ENST00000394333	protein_coding	37009789	37009988	-	37009917	71	-	60S ribosomal protein L23 (Ribosomal protein L17) [Source:UniProtKB/Swiss-Prot;Acc:P62829]
ENSG00000125691	RPL23	ENST00000394332	protein_coding	37009776	37009975	-	37009917	58	-	60S ribosomal protein L23 (Ribosomal protein L17) [Source:UniProtKB/Swiss-Prot;Acc:P62829]
ENSG00000114391	RPL24	ENST00000394077	protein_coding	101405364	1,01E+08	-	1,01E+08	83	-	60S ribosomal protein L24 (Ribosomal protein L30) [Source:UniProtKB/Swiss-Prot;Acc:P83731]
ENSG00000161970	RPL26	ENST00000334048	protein_coding	8286312	8286511	-	8286434	77	-	60S ribosomal protein L26 [Source:UniProtKB/Swiss-Prot;Acc:P61254]
ENSG00000144713	RPL32	ENST00000273223	protein_coding	12882846	12883045	-	12882970	75	-	60S ribosomal protein L32 [Source:UniProtKB/Swiss-Prot;Acc:P62910]
ENSG00000144713	RPL32	ENST00000452606	protein_coding	12882843	12883042	-	12882970	72	-	60S ribosomal protein L32 [Source:UniProtKB/Swiss-Prot;Acc:P62910]
ENSG00000144713	RPL32	ENST00000457131	protein_coding	12882844	12883043	-	12882970	73	-	60S ribosomal protein L32 [Source:UniProtKB/Swiss-Prot;Acc:P62910]
ENSG00000144713	RPL32	ENST00000435983	protein_coding	12882845	12883044	-	12882970	74	-	60S ribosomal protein L32 [Source:UniProtKB/Swiss-Prot;Acc:P62910]
ENSG00000136942	RPL35	ENST00000348462	protein_coding	127624041	1,28E+08	-	1,28E+08	78	-	60S ribosomal protein L35 [Source:UniProtKB/Swiss-Prot;Acc:P42766]
ENSG00000145592	RPL37	ENST00000315577	protein_coding	40835113	40835312	-	40835246	66	-	60S ribosomal protein L37 (G1.16) [Source:UniProtKB/Swiss-Prot;Acc:P61927]
ENSG00000198918	RPL39	ENST00000361575	protein_coding	118925407	1,19E+08	-	1,19E+08	90	-	60S ribosomal protein L39 [Source:UniProtKB/Swiss-Prot;Acc:P62891]
ENSG00000147604	RPL7	ENST00000396467	protein_coding	74205669	74205868	-	74205795	73	-	60S ribosomal protein L7 [Source:UniProtKB/Swiss-Prot;Acc:P18124]
ENSG00000147604	RPL7	ENST00000352983	protein_coding	74205670	74205869	-	74205795	74	-	60S ribosomal protein L7 [Source:UniProtKB/Swiss-Prot;Acc:P18124]
ENSG00000161016	RPL8	ENST00000444860	protein_coding	146017576	1,46E+08	-	1,46E+08	72	-	60S ribosomal protein L8 [Source:UniProtKB/Swiss-Prot;Acc:P62917]

ENSEMBL55 GeneID	Gene Symbol	ENSEMBL55 TranscriptID	Biotype	Gene start	Gene end	Strand	Motif start	TSS offset	Motif strand	Description
ENSG00000161016	RPL8	ENST00000394920	protein_coding	146017530	1,46E+08	-	1,46E+08	26	-	60S ribosomal protein L8 [Source:UniProtKB/Swiss-Prot;Acc:P62917]
ENSG00000163682	RPL9	ENST00000437992	protein_coding	39460345	39460544	-	39460463	81	-	60S ribosomal protein L9 [Source:UniProtKB/Swiss-Prot;Acc:P32969]
ENSG00000164587	RPS14	ENST00000401695	protein_coding	149829120	1,5E+08	-	1,5E+08	87	-	40S ribosomal protein S14 [Source:UniProtKB/Swiss-Prot;Acc:P62263]
ENSG00000164587	RPS14	ENST00000312037	protein_coding	149829120	1,5E+08	-	1,5E+08	87	-	40S ribosomal protein S14 [Source:UniProtKB/Swiss-Prot;Acc:P62263]
ENSG00000164587	RPS14	ENST00000407193	protein_coding	149829120	1,5E+08	-	1,5E+08	87	-	40S ribosomal protein S14 [Source:UniProtKB/Swiss-Prot;Acc:P62263]
ENSG00000184779	RPS17	ENST00000330339	protein_coding	82824666	82824865	-	82824786	79	-	40S ribosomal protein S17 [Source:UniProtKB/Swiss-Prot;Acc:P08708]
ENSG00000184779	RPS17	ENST00000330339	protein_coding	82824666	82824865	-	82824796	69	-	40S ribosomal protein S17 [Source:UniProtKB/Swiss-Prot;Acc:P08708]
ENSG00000140988	RPS2	ENST00000343262	protein_coding	2014628	2014827	-	2014748	79	-	40S ribosomal protein S2 (S4)(LLRep3 protein) [Source:UniProtKB/Swiss-Prot;Acc:P15880]
ENSG00000198034	RPS4X	ENST00000373626	protein_coding	71496869	71497068	-	71496997	71	-	40S ribosomal protein S4, X isoform (Single copy abundant mRNA protein)(SCR10) [Source:UniProtKB/Swiss-Prot;Acc:P62701]
ENSG00000114650	SCAP	ENST00000360832	protein_coding	47484284	47484483	-	47484403	80	-	Sterol regulatory element-binding protein cleavage-activating protein (SREBP cleavage-activating protein)(SCAP) [Source:UniProtKB/Swiss-Prot;Acc:Q12770]
ENSG00000168356	SCN11A	ENST00000455351	protein_coding	38991853	38992052	-	38992015	37	-	Sodium channel protein type 11 subunit alpha (Sodium channel protein type XI subunit alpha)(Voltage-gated sodium channel subunit alpha Nav1.9)(Sensory neuron sodium channel 2)(Peripheral nerve sodium channel 5)(hNaN) [Source:UniProtKB/Swiss-Prot;Acc:Q9UI33]
ENSG00000168356	SCN11A	ENST00000383755	protein_coding	38991853	38992052	-	38992015	37	-	Sodium channel protein type 11 subunit alpha (Sodium channel protein type XI subunit alpha)(Voltage-gated sodium channel subunit alpha Nav1.9)(Sensory neuron sodium channel 2)(Peripheral nerve sodium channel 5)(hNaN) [Source:UniProtKB/Swiss-Prot;Acc:Q9UI33]
ENSG00000168356	SCN11A	ENST00000302328	protein_coding	38991853	38992052	-	38992015	37	-	Sodium channel protein type 11 subunit alpha (Sodium channel protein type XI subunit alpha)(Voltage-gated sodium channel subunit alpha Nav1.9)(Sensory neuron sodium channel 2)(Peripheral nerve sodium channel 5)(hNaN) [Source:UniProtKB/Swiss-Prot;Acc:Q9UI33]
ENSG00000168356	SCN11A	ENST00000450244	protein_coding	38991853	38992052	-	38992015	37	-	Sodium channel protein type 11 subunit alpha (Sodium channel protein type XI subunit alpha)(Voltage-gated sodium channel subunit alpha Nav1.9)(Sensory neuron sodium channel 2)(Peripheral nerve sodium channel 5)(hNaN) [Source:UniProtKB/Swiss-Prot;Acc:Q9UI33]
ENSG00000168356	SCN11A	ENST00000444237	protein_coding	38991853	38992052	-	38992015	37	-	Sodium channel protein type 11 subunit alpha (Sodium channel protein type XI subunit alpha)(Voltage-gated sodium channel subunit alpha Nav1.9)(Sensory neuron sodium channel 2)(Peripheral nerve sodium channel 5)(hNaN) [Source:UniProtKB/Swiss-Prot;Acc:Q9UI33]

ENSEMBL55 GeneID	Gene Symbol	ENSEMBL55 TranscriptID	Biotype	Gene start	Gene end	Strand	Motif start	TSS offset	Motif strand	Description
ENSG00000138674	SEC31A	ENST00000355196	protein_coding	83821492	83821691	-	83821616	75	-	Protein transport protein Sec31A (SEC31-related protein A)(SEC31-like 1)(ABP125)(ABP130)(Web1-like protein) [Source:UniProtKB/Swiss-Prot;Acc:O94979]
ENSG00000075213	SEMA3A	ENST00000420047	protein_coding	83824278	83824477	-	83824413	64	-	Semaphorin-3A Precursor (Semaphorin III)(Sema III) [Source:UniProtKB/Swiss-Prot;Acc:Q14563]
ENSG00000156304	SFRS15	ENST00000435228	protein_coding	33083588	33083787	-	33083737	50	-	Splicing factor, arginine/serine-rich 15 (CTD-binding SR-like protein RA4) [Source:UniProtKB/Swiss-Prot;Acc:O95104]
ENSG00000156304	SFRS15	ENST00000419806	protein_coding	33083588	33083787	-	33083737	50	-	Splicing factor, arginine/serine-rich 15 (CTD-binding SR-like protein RA4) [Source:UniProtKB/Swiss-Prot;Acc:O95104]
ENSG00000156304	SFRS15	ENST00000434667	protein_coding	33083588	33083787	-	33083737	50	-	Splicing factor, arginine/serine-rich 15 (CTD-binding SR-like protein RA4) [Source:UniProtKB/Swiss-Prot;Acc:O95104]
ENSG00000132424	SFRS18	ENST00000438806	protein_coding	99873008	99873207	-	99873182	25	-	Splicing factor, arginine/serine-rich 18 (Splicing factor, arginine/serine-rich 130)(Serine-arginine-rich-splicing regulatory protein 130)(SRrp130)(SR-rich protein)(SR-related protein) [Source:UniProtKB/Swiss-Prot;Acc:Q8TF01]
ENSG00000132424	SFRS18	ENST00000369239	protein_coding	99873008	99873207	-	99873182	25	-	Splicing factor, arginine/serine-rich 18 (Splicing factor, arginine/serine-rich 130)(Serine-arginine-rich-splicing regulatory protein 130)(SRrp130)(SR-rich protein)(SR-related protein) [Source:UniProtKB/Swiss-Prot;Acc:Q8TF01]
ENSG00000004809	SLC22A16	ENST00000434949	protein_coding	110797444	1,11E+08	-	1,11E+08	24	-	Solute carrier family 22 member 16 (Carnitine transporter 2)(CT2)(Organic cation/carnitine transporter 6)(Organic cation transporter OKB1)(Fly-like putative transporter 2)(Flipt 2)(FLIPT2) [Source:UniProtKB/Swiss-Prot;Acc:Q86VW1]
ENSG00000112499	SLC22A2	ENST00000366952	protein_coding	160698471	1,61E+08	-	1,61E+08	61	-	Solute carrier family 22 member 2 (Organic cation transporter 2)(hOCT2) [Source:UniProtKB/Swiss-Prot;Acc:O15244]
ENSG00000170615	SLC26A5	ENST00000393723	protein_coding	103061770	1,03E+08	-	1,03E+08	83	-	Prestin (Solute carrier family 26 member 5) [Source:UniProtKB/Swiss-Prot;Acc:P58743]
ENSG00000170615	SLC26A5	ENST00000393727	protein_coding	103061770	1,03E+08	-	1,03E+08	83	-	Prestin (Solute carrier family 26 member 5) [Source:UniProtKB/Swiss-Prot;Acc:P58743]
ENSG00000059804	SLC2A3	ENST00000396584	protein_coding	8082320	8082519	-	8082424	95	-	Solute carrier family 2, facilitated glucose transporter member 3 (Glucose transporter type 3, brain)(GLUT-3) [Source:UniProtKB/Swiss-Prot;Acc:P11169]
ENSG00000127526	SLC35E1	ENST00000421082	protein_coding	16667747	16667946	-	16667862	84	-	Solute carrier family 35 member E1 [Source:UniProtKB/Swiss-Prot;Acc:Q96K37]
ENSG00000148942	SLC5A12	ENST00000340797	protein_coding	26744774	26744973	-	26744924	49	-	Sodium-coupled monocarboxylate transporter 2 (Electroneutral sodium monocarboxylate cotransporter)(Low-affinity sodium-lactate cotransporter)(Solute carrier family 5 member 12) [Source:UniProtKB/Swiss-Prot;Acc:Q1EHB4]
ENSG00000138074	SLC5A6	ENST00000432106	protein_coding	27435619	27435818	-	27435784	34	-	Sodium-dependent multivitamin transporter (Na(+)-dependent multivitamin transporter)(Solute carrier family 5 member 6) [Source:UniProtKB/Swiss-Prot;Acc:Q9Y289]
ENSG00000138074	SLC5A6	ENST00000442731	protein_coding	27435627	27435826	-	27435784	42	-	Sodium-dependent multivitamin transporter (Na(+)-dependent multivitamin transporter)(Solute carrier family 5 member 6) [Source:UniProtKB/Swiss-Prot;Acc:Q9Y289]
ENSG00000164893	SLC7A13	ENST00000297524	protein_coding	87242405	87242604	-	87242578	26	-	Solute carrier family 7 member 13 (Sodium-independent aspartate/glutamate transporter 1)(X-amino acid transporter 2) [Source:UniProtKB/Swiss-Prot;Acc:Q8TCU3]

ENSEMBL55 GeneID	Gene Symbol	ENSEMBL55 TranscriptID	Biotype	Gene start	Gene end	Strand	Motif start	TSS offset	Motif strand	Description
ENSG00000134278	SPIRE1	ENST00000453447	protein_coding	12656532	12656731	-	12656669	62	-	Protein spire homolog 1 (Spir-1) [Source:UniProtKB/Swiss-Prot;Acc:Q08AE8]
ENSG00000136158	SPRY2	ENST00000377102	protein_coding	80913595	80913794	-	80913712	82	-	Protein sprouty homolog 2 (Spry-2) [Source:UniProtKB/Swiss-Prot;Acc:O43597]
ENSG00000174448	STARD6	ENST00000307844	protein_coding	51880744	51880943	-	51880879	64	-	StAR-related lipid transfer protein 6 (START domain-containing protein 6)(StARD6) [Source:UniProtKB/Swiss-Prot;Acc:P59095]
ENSG00000146378	TAAR2	ENST00000275191	protein_coding	132939010	1,33E+08	-	1,33E+08	32	-	Trace amine-associated receptor 2 (G-protein coupled receptor 58) [Source:UniProtKB/Swiss-Prot;Acc:Q9P1P5]
ENSG00000106290	TAF6	ENST00000417771	protein_coding	99711633	99711832	-	99711749	83	-	Transcription initiation factor TFIID subunit 6 (Transcription initiation factor TFIID 70 kDa subunit)(TAF(II)70)(TAFII-70)(TAFII-80)(TAFII80)(RNA polymerase II TBP-associated factor subunit E) [Source:UniProtKB/Swiss-Prot;Acc:P49848]
ENSG00000131374	TBC1D5	ENST00000450163	protein_coding	17549901	17550100	-	17550046	54	-	TBC1 domain family member 5 [Source:UniProtKB/Swiss-Prot;Acc:Q92609]
ENSG00000125247	TMTC4	ENST00000344945	protein_coding	101322417	1,01E+08	-	1,01E+08	36	-	Transmembrane and TPR repeat-containing protein 4 [Source:UniProtKB/Swiss-Prot;Acc:Q5T4D3]
ENSG00000119121	TRPM6	ENST00000448641	protein_coding	77502811	77503010	-	77502967	43	-	Transient receptor potential cation channel subfamily M member 6 (EC 2.7.11.1)(Channel kinase 2)(Melastatin-related TRP cation channel 6) [Source:UniProtKB/Swiss-Prot;Acc:Q9BX84]
ENSG00000119121	TRPM6	ENST00000376864	protein_coding	77502811	77503010	-	77502967	43	-	Transient receptor potential cation channel subfamily M member 6 (EC 2.7.11.1)(Channel kinase 2)(Melastatin-related TRP cation channel 6) [Source:UniProtKB/Swiss-Prot;Acc:Q9BX84]
ENSG00000119121	TRPM6	ENST00000360774	protein_coding	77502811	77503010	-	77502967	43	-	Transient receptor potential cation channel subfamily M member 6 (EC 2.7.11.1)(Channel kinase 2)(Melastatin-related TRP cation channel 6) [Source:UniProtKB/Swiss-Prot;Acc:Q9BX84]
ENSG00000119121	TRPM6	ENST00000376871	protein_coding	77502811	77503010	-	77502967	43	-	Transient receptor potential cation channel subfamily M member 6 (EC 2.7.11.1)(Channel kinase 2)(Melastatin-related TRP cation channel 6) [Source:UniProtKB/Swiss-Prot;Acc:Q9BX84]
ENSG00000119121	TRPM6	ENST00000376872	protein_coding	77502811	77503010	-	77502967	43	-	Transient receptor potential cation channel subfamily M member 6 (EC 2.7.11.1)(Channel kinase 2)(Melastatin-related TRP cation channel 6) [Source:UniProtKB/Swiss-Prot;Acc:Q9BX84]
ENSG00000119121	TRPM6	ENST00000359047	protein_coding	77502811	77503010	-	77502967	43	-	Transient receptor potential cation channel subfamily M member 6 (EC 2.7.11.1)(Channel kinase 2)(Melastatin-related TRP cation channel 6) [Source:UniProtKB/Swiss-Prot;Acc:Q9BX84]
ENSG00000104447	TRPS1	ENST00000395713	protein_coding	116673706	1,17E+08	-	1,17E+08	40	-	Zinc finger transcription factor Trps1 (Tricho-rhino-phalangeal syndrome type I protein)(Zinc finger protein GC79) [Source:UniProtKB/Swiss-Prot;Acc:Q9UHF7]
ENSG00000136810	TXN	ENST00000374517	protein_coding	113018622	1,13E+08	-	1,13E+08	68	-	Thioredoxin (Trx)(ATL-derived factor)(ADF)(Surface-associated sulphydryl protein)(SASP) [Source:UniProtKB/Swiss-Prot;Acc:P10599]

ENSEMBL55 GeneID	Gene Symbol	ENSEMBL55 TranscriptID	Biotype	Gene start	Gene end	Strand	Motif start	TSS offset	Motif strand	Description
ENSG00000136810	TXN	ENST00000374515	protein_coding	113018622	1,13E+08	-	1,13E+08	68	-	Thioredoxin (Trx)(ATL-derived factor)(ADF)(Surface-associated sulphydryl protein)(SASP) [Source:UniProtKB/Swiss-Prot;Acc:P10599]
ENSG00000173610	UGT2A1	ENST00000286604	protein_coding	70513219	70513418	-	70513382	36	-	UDP-glucuronosyltransferase 2A1 Precursor (UDPGT 2A1)(EC 2.4.1.17) [Source:UniProtKB/Swiss-Prot;Acc:Q9Y4X1]
ENSG00000173610	UGT2A1	ENST00000457664	protein_coding	70513219	70513418	-	70513382	36	-	UDP-glucuronosyltransferase 2A1 Precursor (UDPGT 2A1)(EC 2.4.1.17) [Source:UniProtKB/Swiss-Prot;Acc:Q9Y4X1]
ENSG00000173610	UGT2A1	ENST00000286604	protein_coding	70513219	70513418	-	70513363	55	-	UDP-glucuronosyltransferase 2A1 Precursor (UDPGT 2A1)(EC 2.4.1.17) [Source:UniProtKB/Swiss-Prot;Acc:Q9Y4X1]
ENSG00000173610	UGT2A1	ENST00000457664	protein_coding	70513219	70513418	-	70513363	55	-	UDP-glucuronosyltransferase 2A1 Precursor (UDPGT 2A1)(EC 2.4.1.17) [Source:UniProtKB/Swiss-Prot;Acc:Q9Y4X1]
ENSG00000130477	UNC13A	ENST00000252773	protein_coding	17786779	17786978	-	17786913	65	-	Protein unc-13 homolog A (Munc13-1) [Source:UniProtKB/Swiss-Prot;Acc:Q9UPW8]
ENSG00000049247	UTS2	ENST00000400910	protein_coding	7912905	7913104	-	7913005	99	-	Urotensin-2 Precursor (Urotensin-II)(U-II)(UII) [Source:UniProtKB/Swiss-Prot;Acc:O95399]
ENSG00000049247	UTS2	ENST00000361696	protein_coding	7912905	7913104	-	7913005	99	-	Urotensin-2 Precursor (Urotensin-II)(U-II)(UII) [Source:UniProtKB/Swiss-Prot;Acc:O95399]
ENSG00000182489	XKRX	ENST00000372956	protein_coding	100183094	1E+08	-	1E+08	52	-	XK-related protein 2 (X Kell blood group-related, X-linked)(Membrane protein XPLAC) [Source:UniProtKB/Swiss-Prot;Acc:Q6PP77]
ENSG00000176601	YSK4	ENST00000437365	protein_coding	135744412	1,36E+08	-	1,36E+08	30	-	SPS1/STE20-related protein kinase YSK4 (EC 2.7.11.1)(Regulated in COPD, protein kinase) [Source:UniProtKB/Swiss-Prot;Acc:Q56UN5]
ENSG00000108953	YWHAE	ENST00000414131	protein_coding	1303295	1303494	-	1303438	56	-	14-3-3 protein epsilon (14-3-3E) [Source:UniProtKB/Swiss-Prot;Acc:P62258]
ENSG00000108953	YWHAE	ENST00000440251	protein_coding	1303295	1303494	-	1303438	56	-	14-3-3 protein epsilon (14-3-3E) [Source:UniProtKB/Swiss-Prot;Acc:P62258]
ENSG00000197608	ZNF841	ENST00000359973	protein_coding	52570587	52570786	-	52570723	63	-	Zinc finger protein 841 [Source:UniProtKB/Swiss-Prot;Acc:Q6ZN19]
ENSG00000184389	A3GALT2	ENST00000330379	protein_coding	33786500	33786699	-	33786606	93	+	Alpha 1,3-galactosyltransferase 2 Precursor (A3galt2)(EC 2.4.1.87)(Isoglobotriaosylceramide synthase)(iGb3 synthase)(iGb3S) [Source:UniProtKB/Swiss-Prot;Acc:Q5T0B8]
ENSG00000085563	ABCB1	ENST00000265724	protein_coding	87342365	87342564	-	87342535	29	+	Multidrug resistance protein 1 (EC 3.6.3.44)(ATP-binding cassette sub-family B member 1)(P-glycoprotein 1)(CD243 antigen) [Source:UniProtKB/Swiss-Prot;Acc:P08183]
ENSG00000085563	ABCB1	ENST00000416177	protein_coding	87342411	87342610	-	87342535	75	+	Multidrug resistance protein 1 (EC 3.6.3.44)(ATP-binding cassette sub-family B member 1)(P-glycoprotein 1)(CD243 antigen) [Source:UniProtKB/Swiss-Prot;Acc:P08183]
ENSG00000085563	ABCB1	ENST00000394661	protein_coding	87342371	87342570	-	87342535	35	+	Multidrug resistance protein 1 (EC 3.6.3.44)(ATP-binding cassette sub-family B member 1)(P-glycoprotein 1)(CD243 antigen) [Source:UniProtKB/Swiss-Prot;Acc:P08183]
ENSG00000150967	ABCB9	ENST00000442028	protein_coding	123428939	1,23E+08	-	1,23E+08	29	+	ATP-binding cassette sub-family B member 9 Precursor (ATP-binding cassette transporter 9)(ABC transporter 9 protein)(hABCB9)(TAP-like protein)(TAPL) [Source:UniProtKB/Swiss-Prot;Acc:Q9NP78]
ENSG00000121270	ABCC11	ENST00000394747	protein_coding	48265983	48266182	-	48266114	68	+	ATP-binding cassette transporter sub-family C member 11 (Multidrug resistance-associated protein 8)

ENSEMBL55 GeneID	Gene Symbol	ENSEMBL55 TranscriptID	Biotype	Gene start	Gene end	Strand	Motif start	TSS offset	Motif strand	Description
										[Source:UniProtKB/Swiss-Prot;Acc:Q96J66]
ENSG00000225481	AC006222.1	ENST00000423047	protein_coding	58266646	58266845	-	58266761	84	+	
ENSG00000205047	AC010536.8-2	ENST00000446344	protein_coding	87739091	87739290	-	87739213	77	+	hypothetical LOC100129637 (LOC100129637), non-coding RNA [Source:RefSeq DNA;Acc:NR_024488]
ENSG00000205047	AC010536.8-2	ENST00000378443	protein_coding	87739091	87739290	-	87739213	77	+	hypothetical LOC100129637 (LOC100129637), non-coding RNA [Source:RefSeq DNA;Acc:NR_024488]
ENSG00000205718	AC010606.7-3	ENST00000381394	protein_coding	7039956	7040155	-	7040088	67	+	Putative methyl-CpG-binding domain protein 3-like 4 (MBD3-like 4) [Source:UniProtKB/Swiss-Prot;Acc:A6NDZ8]
ENSG00000198787	AC011744.8	ENST00000361653	protein_coding	4176791	4176990	-	4176945	45	+	
ENSG00000224163	AC025594.5-1	ENST00000433622	protein_coding	128562925	1,29E+08	-	1,29E+08	59	+	Similar to Filamin C (Gamma-filamin) (Filamin 2) (Protein FLNc) (Actin-binding like protein) (ABP-L) (ABP-280-like protein) [Source:UniProtKB/TrEMBL;Acc:A4D1J9]
ENSG00000236147	AC068733.12-2	ENST00000424838	protein_coding	6412704	6412903	-	6412817	86	+	SMPD1 protein [Source:UniProtKB/TrEMBL;Acc:Q15495]
ENSG00000204733	AC087650.12-2	ENST00000377237	protein_coding	41466370	41466569	-	41466494	75	+	Putative uncharacterized protein ENSP00000366445 [Source:UniProtKB/TrEMBL;Acc:B7WPQ4]
ENSG00000232776	AC090691.16-1	ENST00000426631	protein_coding	578853	579052	-	578993	59	+	FLJ00290 protein Fragment [Source:UniProtKB/TrEMBL;Acc:Q8NF75]
ENSG00000214803	AC090921.7-1	ENST00000399016	protein_coding	125259440	1,25E+08	-	1,25E+08	91	+	Putative uncharacterized protein Fragment [Source:UniProtKB/TrEMBL;Acc:Q96BV5]
ENSG00000218761	AC093227.3	ENST00000447434	protein_coding	38184266	38184465	-	38184398	67	+	
ENSG00000215749	AC126339.6-2	ENST00000418912	protein_coding	82938862	82939061	-	82938963	98	+	cDNA FLJ61456, highly similar to Homo sapiens golgi autoantigen, golgin subfamily a-like, mRNA [Source:UniProtKB/TrEMBL;Acc:B4DXR5]
ENSG00000233050	AC130366.6-1	ENST00000437818	protein_coding	11929057	11929256	-	11929182	74	+	Beta-defensin 130 Precursor (Defensin, beta 130)(Beta-defensin 30)(DEFB-30) [Source:UniProtKB/Swiss-Prot;Acc:Q30KQ2]
ENSG00000226787	AC135048.2-3	ENST00000420533	protein_coding	31002326	31002525	-	31002439	86	+	FP17469 [Source:UniProtKB/TrEMBL;Acc:Q6XYE6]
ENSG00000205281	AC139425.3-2	ENST00000430944	protein_coding	82641371	82641570	-	82641472	98	+	Putative golgin subfamily A member 6-like protein 10 [Source:UniProtKB/Swiss-Prot;Acc:A6NI86]
ENSG00000212865	AC145060.2	ENST00000391554	protein_coding	12531	12730	-	12692	38	+	PRO2007 [Source:UniProtKB/TrEMBL;Acc:Q9P1F5]
ENSG00000114331	ACAP2	ENST00000439666	protein_coding	195163385	1,95E+08	-	1,95E+08	25	+	ARFGAP with coiled-coil, ANK repeat and PH domain-containing protein 2 (Centaurin-beta-2)(Cnt-b2) [Source:UniProtKB/Swiss-Prot;Acc:Q15057]
ENSG00000114331	ACAP2	ENST00000447662	protein_coding	195163435	1,95E+08	-	1,95E+08	75	+	ARFGAP with coiled-coil, ANK repeat and PH domain-containing protein 2 (Centaurin-beta-2)(Cnt-b2) [Source:UniProtKB/Swiss-Prot;Acc:Q15057]
ENSG00000135049	AGTPBP1	ENST00000357081	protein_coding	88356602	88356801	-	88356752	49	+	Cytosolic carboxypeptidase 1 (EC 3.4.17.-)(ATP/GTP-binding protein 1)(Nervous system nuclear protein induced by axotomy) [Source:UniProtKB/Swiss-Prot;Acc:Q9UPW5]
ENSG00000196326	AKR1CL1	ENST00000334314	protein_coding	5226945	5227144	-	5227056	88	+	Aldo-keto reductase family 1 member C-like protein 1 (EC 1.1.1.-) [Source:UniProtKB/Swiss-Prot;Acc:Q5T2L2]
ENSG00000196326	AKR1CL1	ENST00000380510	protein_coding	5226945	5227144	-	5227056	88	+	Aldo-keto reductase family 1 member C-like protein 1 (EC 1.1.1.-) [Source:UniProtKB/Swiss-Prot;Acc:Q5T2L2]

ENSEMBL55 GeneID	Gene Symbol	ENSEMBL55 TranscriptID	Biotype	Gene start	Gene end	Strand	Motif start	TSS offset	Motif strand	Description
ENSG00000225983	AL031274.1	ENST00000424590	protein_coding	171235621	1,71E+08	-	1,71E+08	61	+	PRO1257 [Source:UniProtKB/TrEMBL;Acc:Q9P1K0]
ENSG00000204879	AL603926.6-1	ENST00000440550	protein_coding	22783	22982	-	22889	93	+	
ENSG00000148218	ALAD	ENST00000277315	protein_coding	116163343	1,16E+08	-	1,16E+08	77	+	Delta-aminolevulinic acid dehydratase (ALADH)(EC 4.2.1.24)(Porphobilinogen synthase) [Source:UniProtKB/Swiss-Prot;Acc:P13716]
ENSG00000148218	ALAD	ENST00000374173	protein_coding	116163343	1,16E+08	-	1,16E+08	77	+	Delta-aminolevulinic acid dehydratase (ALADH)(EC 4.2.1.24)(Porphobilinogen synthase) [Source:UniProtKB/Swiss-Prot;Acc:P13716]
ENSG00000179148	ALOXE3	ENST00000448843	protein_coding	8021661	8021860	-	8021825	35	+	Epidermis-type lipoxygenase 3 (e-LOX-3)(EC 1.13.11.-) [Source:UniProtKB/Swiss-Prot;Acc:Q9BYJ1]
ENSG00000128805	ARHGAP22	ENST00000249601	protein_coding	49812977	49813176	-	49813099	77	+	Rho GTPase-activating protein 22 (Rho-type GTPase-activating protein 22) [Source:UniProtKB/Swiss-Prot;Acc:Q7Z5H3]
ENSG00000128805	ARHGAP22	ENST00000435790	protein_coding	49812977	49813176	-	49813099	77	+	Rho GTPase-activating protein 22 (Rho-type GTPase-activating protein 22) [Source:UniProtKB/Swiss-Prot;Acc:Q7Z5H3]
ENSG00000128805	ARHGAP22	ENST00000417912	protein_coding	49812977	49813176	-	49813099	77	+	Rho GTPase-activating protein 22 (Rho-type GTPase-activating protein 22) [Source:UniProtKB/Swiss-Prot;Acc:Q7Z5H3]
ENSG00000047648	ARHGAP6	ENST00000380717	protein_coding	11308399	11308598	-	11308545	53	+	Rho GTPase-activating protein 6 (Rho-type GTPase-activating protein 6)(Rho-type GTPase-activating protein RhoGAPX-1) [Source:UniProtKB/Swiss-Prot;Acc:O43182]
ENSG00000100325	ASCC2	ENST00000431535	protein_coding	30230340	30230539	-	30230494	45	+	Activating signal cointegrator 1 complex subunit 2 (ASC-1 complex subunit p100)(Trip4 complex subunit p100) [Source:UniProtKB/Swiss-Prot;Acc:Q9H1I8]
ENSG00000100325	ASCC2	ENST00000458594	protein_coding	30230340	30230539	-	30230494	45	+	Activating signal cointegrator 1 complex subunit 2 (ASC-1 complex subunit p100)(Trip4 complex subunit p100) [Source:UniProtKB/Swiss-Prot;Acc:Q9H1I8]
ENSG00000142867	BCL10	ENST00000271015	protein_coding	85743572	85743771	-	85743719	52	+	B-cell lymphoma/leukemia 10 (B-cell CLL/lymphoma 10)(Bcl-10)(CED-3/ICH-1 prodomain homologous E10-like regulator)(CIPER)(CARD-containing molecule enhancing NF-kappa-B)(Cellular homolog of vCARMEN)(cCARMEN)(Mammalian CARD-containing adapter molecule E10)(mE10)(Cellular-E10)(c-E10)(CARD-like apoptotic protein)(hCLAP) [Source:UniProtKB/Swiss-Prot;Acc:O95999]
ENSG00000183826	BTBD9	ENST00000373384	protein_coding	38563565	38563764	-	38563681	83	+	BTB/POZ domain-containing protein 9 [Source:UniProtKB/Swiss-Prot;Acc:Q96Q07]
ENSG00000183826	BTBD9	ENST00000328403	protein_coding	38563565	38563764	-	38563681	83	+	BTB/POZ domain-containing protein 9 [Source:UniProtKB/Swiss-Prot;Acc:Q96Q07]
ENSG00000120685	C13orf23	ENST00000379609	protein_coding	39605617	39605816	-	39605750	66	+	Uncharacterized protein KIAA2032 [Source:UniProtKB/Swiss-Prot;Acc:Q86XN7]
ENSG00000120685	C13orf23	ENST00000436678	protein_coding	39605647	39605846	-	39605750	96	+	Uncharacterized protein KIAA2032 [Source:UniProtKB/Swiss-Prot;Acc:Q86XN7]
ENSG00000108666	C17orf75	ENST00000225805	protein_coding	30668990	30669189	-	30669123	66	+	Protein Njmu-R1 [Source:UniProtKB/Swiss-Prot;Acc:Q9HAS0]
ENSG00000119559	C19orf25	ENST00000427685	protein_coding	1478703	1478902	-	1478825	77	+	UPF0449 protein C19orf25 [Source:UniProtKB/Swiss-Prot;Acc:Q9UFG5]
ENSG00000178965	C1orf173	ENST00000342217	protein_coding	75132329	75132528	-	75132475	53	+	Uncharacterized protein C1orf173 [Source:UniProtKB/Swiss-Prot;Acc:Q5RHP9]

ENSEMBL55 GeneID	Gene Symbol	ENSEMBL55 TranscriptID	Biotype	Gene start	Gene end	Strand	Motif start	TSS offset	Motif strand	Description
ENSG00000101353	C20orf132	ENST00000437321	protein_coding	35788338	35788537	-	35788495	42	+	Uncharacterized protein C20orf132 [Source:UniProtKB/Swiss-Prot;Acc:Q9H579]
ENSG00000101220	C20orf27	ENST00000399683	protein_coding	3739126	3739325	-	3739269	56	+	UPF0687 protein C20orf27 [Source:UniProtKB/Swiss-Prot;Acc:Q9GZN8]
ENSG00000198221	C6orf124	ENST00000417244	protein_coding	168225941	1,68E+08	-	1,68E+08	41	+	Uncharacterized protein C6orf124 (Protein HGC6.4) [Source:UniProtKB/Swiss-Prot;Acc:Q9Y6Z5]
ENSG00000146350	C6orf170	ENST00000422369	protein_coding	121655692	1,22E+08	-	1,22E+08	24	+	Uncharacterized protein C6orf170 [Source:UniProtKB/Swiss-Prot;Acc:Q96NH3]
ENSG00000235865	C9orf31	ENST00000437135	protein_coding	124044809	1,24E+08	-	1,24E+08	86	+	Putative uncharacterized protein C9orf31 (Protein MOST-2) [Source:UniProtKB/Swiss-Prot;Acc:Q9NRJ2]
ENSG00000130559	CAMSAP1	ENST00000312405	protein_coding	138774544	1,39E+08	-	1,39E+08	33	+	Calmodulin-regulated spectrin-associated protein 1 [Source:UniProtKB/Swiss-Prot;Acc:Q5T5Y3]
ENSG00000079112	CDH17	ENST00000450165	protein_coding	95229332	95229531	-	95229487	44	+	Cadherin-17 Precursor (Liver-intestine cadherin)(LI-cadherin)(Intestinal peptide-associated transporter HPT-1) [Source:UniProtKB/Swiss-Prot;Acc:Q12864]
ENSG00000093072	CECR1	ENST00000449907	protein_coding	17670980	17671179	-	17671114	65	+	Cat eye syndrome critical region protein 1 Precursor [Source:UniProtKB/Swiss-Prot;Acc:Q9NZK5]
ENSG00000182022	CHST15	ENST00000435907	protein_coding	125806042	1,26E+08	-	1,26E+08	88	+	N-acetylgalactosamine 4-sulfate 6-O-sulfotransferase (EC 2.8.2.33)(GalNAc4S-6ST)(B-cell RAG-associated gene protein)(hBRAG) [Source:UniProtKB/Swiss-Prot;Acc:Q7LFX5]
ENSG00000095321	CRAT	ENST00000415948	protein_coding	131871356	1,32E+08	-	1,32E+08	89	+	Carnitine O-acetyltransferase (Carnitine acetylase)(EC 2.3.1.7)(Carnitine acetyltransferase)(CrAT)(CAT) [Source:UniProtKB/Swiss-Prot;Acc:P43155]
ENSG00000173406	DAB1	ENST00000371231	protein_coding	57756538	57756737	-	57756701	36	+	Disabled homolog 1 [Source:UniProtKB/Swiss-Prot;Acc:O75553]
ENSG00000173406	DAB1	ENST00000332102	protein_coding	57756538	57756737	-	57756701	36	+	Disabled homolog 1 [Source:UniProtKB/Swiss-Prot;Acc:O75553]
ENSG00000173406	DAB1	ENST00000371232	protein_coding	57756538	57756737	-	57756701	36	+	Disabled homolog 1 [Source:UniProtKB/Swiss-Prot;Acc:O75553]
ENSG00000188120	DAZ1	ENST00000306922	protein_coding	22637194	22637393	-	22637322	71	+	Deleted in azoospermia protein 1 [Source:UniProtKB/Swiss-Prot;Acc:Q9NQZ3]
ENSG00000232948	DEFB130	ENST00000400079	protein_coding	12175626	12175825	-	12175751	74	+	Beta-defensin 130 Precursor (Defensin, beta 130)(Beta-defensin 30)(DEFB-30) [Source:UniProtKB/Swiss-Prot;Acc:Q30KQ2]
ENSG00000159123	DMRTC1B	ENST00000373530	protein_coding	72097499	72097698	-	72097602	96	+	Doublesex- and mab-3-related transcription factor C1 [Source:UniProtKB/Swiss-Prot;Acc:Q5HYR2]
ENSG00000185010	F8	ENST00000453950	protein_coding	154254944	1,54E+08	-	1,54E+08	62	+	Coagulation factor VIII Precursor (Procoagulant component)(Antihemophilic factor)(AHF) [Contains Factor VIIIa heavy chain, 200 kDa isoform;Factor VIIIa heavy chain, 92 kDa isoform;Factor VIII B chain;Factor VIIIa light chain] [Source:UniProtKB/Swiss-Prot;Acc:P00451]
ENSG00000146410	FAM54A	ENST00000367784	protein_coding	136570038	1,37E+08	-	1,37E+08	90	+	Protein FAM54A (DUF729 domain-containing protein 1) [Source:UniProtKB/Swiss-Prot;Acc:Q6P444]
ENSG00000215784	FAM72D	ENST00000445255	protein_coding	143914136	1,44E+08	-	1,44E+08	71	+	Gastric cancer up-regulated protein 2 [Source:UniProtKB/Swiss-Prot;Acc:Q6L9T8]
ENSG00000129682	FGF13	ENST00000370603	protein_coding	138067047	1,38E+08	-	1,38E+08	96	+	Fibroblast growth factor 13 (FGF-13)(Fibroblast growth factor homologous factor 2)(FHF-2) [Source:UniProtKB/Swiss-

ENSEMBL55 GeneID	Gene Symbol	ENSEMBL55 TranscriptID	Biotype	Gene start	Gene end	Strand	Motif start	TSS offset	Motif strand	Description
										Prot;Acc:Q92913]
ENSG00000137440	FGFBP1	ENST00000259988	protein_coding	15939772	15939971	-	15939892	79	+	Fibroblast growth factor-binding protein 1 Precursor (FGF-binding protein 1)(FGF-BP1)(FGFBP-1)(FGF-BP)(17 kDa heparin-binding growth factor-binding protein)(17 kDa HBGF-binding protein)(HBp17) [Source:UniProtKB/Swiss-Prot;Acc:Q14512]
ENSG00000171557	FGG	ENST00000443553	protein_coding	155533920	1,56E+08	-	1,56E+08	92	+	Fibrinogen gamma chain Precursor [Source:UniProtKB/Swiss-Prot;Acc:P02679]
ENSG00000159131	GART	ENST00000414353	protein_coding	34883465	34883664	-	34883617	47	+	Trifunctional purine biosynthetic protein adenosine-3 [Includes Phosphoribosylamine--glycine ligase(EC 6.3.4.13)(Glycinamide ribonucleotide synthetase)(GARS)(Phosphoribosylglycinamide synthetase);Phosphoribosylformylglycinamidine cyclo-ligase(EC 6.3.3.1)(Phosphoribosyl-aminoimidazole synthetase)(AIR synthase)(AIRS);Phosphoribosylglycinamide formyltransferase(EC 2.1.2.2)(5'-phosphoribosylglycinamide transformylase)(GAR transformylase)(GART)] [Source:UniProtKB/Swiss-Prot;Acc:P22102]
ENSG00000131233	GJA9	ENST00000357771	protein_coding	39347090	39347289	-	39347244	45	+	Gap junction alpha-9 protein (Gap junction alpha-10 protein)(Connexin-59)(Cx59)(Connexin-58)(Cx58) [Source:UniProtKB/Swiss-Prot;Acc:P57773]
ENSG00000159248	GJD2	ENST00000290374	protein_coding	35046583	35046782	-	35046688	94	+	Gap junction delta-2 protein (Gap junction alpha-9 protein)(Connexin-36)(Cx36) [Source:UniProtKB/Swiss-Prot;Acc:Q9UKL4]
ENSG00000073605	GSDMB	ENST00000309481	protein_coding	38074704	38074903	-	38074832	71	+	Gasdermin-B (Gasdermin-like protein) [Source:UniProtKB/Swiss-Prot;Acc:Q8TAX9]
ENSG00000073605	GSDMB	ENST00000354894	protein_coding	38074704	38074903	-	38074832	71	+	Gasdermin-B (Gasdermin-like protein) [Source:UniProtKB/Swiss-Prot;Acc:Q8TAX9]
ENSG00000073605	GSDMB	ENST00000394179	protein_coding	38074704	38074903	-	38074832	71	+	Gasdermin-B (Gasdermin-like protein) [Source:UniProtKB/Swiss-Prot;Acc:Q8TAX9]
ENSG00000073605	GSDMB	ENST00000433643	protein_coding	38074704	38074903	-	38074832	71	+	Gasdermin-B (Gasdermin-like protein) [Source:UniProtKB/Swiss-Prot;Acc:Q8TAX9]
ENSG00000073605	GSDMB	ENST00000418519	protein_coding	38074704	38074903	-	38074832	71	+	Gasdermin-B (Gasdermin-like protein) [Source:UniProtKB/Swiss-Prot;Acc:Q8TAX9]
ENSG00000073605	GSDMB	ENST00000437352	protein_coding	38074704	38074903	-	38074832	71	+	Gasdermin-B (Gasdermin-like protein) [Source:UniProtKB/Swiss-Prot;Acc:Q8TAX9]
ENSG00000126107	HECTD3	ENST00000372168	protein_coding	45473862	45474061	-	45473982	79	+	Probable E3 ubiquitin-protein ligase HECTD3 (EC 6.3.2.-)(HECT domain-containing protein 3) [Source:UniProtKB/Swiss-Prot;Acc:Q5T447]
ENSG00000138411	HECW2	ENST00000409111	protein_coding	197226747	1,97E+08	-	1,97E+08	77	+	E3 ubiquitin-protein ligase HECW2 (EC 6.3.2.-)(HECT, C2 and WW domain-containing protein 2)(NEDD4-like E3 ubiquitin-protein ligase 2) [Source:UniProtKB/Swiss-Prot;Acc:Q9P2P5]
ENSG00000164508	HIST1H2AA	ENST00000297012	protein_coding	25726591	25726790	-	25726754	36	+	Histone H2A type 1-A (H2A/r) [Source:UniProtKB/Swiss-Prot;Acc:Q96QV6]
ENSG00000120210	INSL6	ENST00000428422	protein_coding	5164052	5164251	-	5164191	60	+	Insulin-like peptide INSL6 Precursor (Insulin-like peptide 6)(Relaxin/insulin-like factor 1) [Contains Insulin-like peptide INSL6 B chain;Insulin-like peptide INSL6 A chain]

ENSEMBL55 GeneID	Gene Symbol	ENSEMBL55 TranscriptID	Biotype	Gene start	Gene end	Strand	Motif start	TSS offset	Motif strand	Description
										[Source:UniProtKB/Swiss-Prot;Acc:Q9Y581]
ENSG00000173389	IQCF1	ENST00000310914	protein_coding	51937152	51937351	-	51937322	29	+	IQ domain-containing protein F1 [Source:UniProtKB/Swiss-Prot;Acc:Q8N6M8]
ENSG00000173389	IQCF1	ENST00000314534	protein_coding	51937152	51937351	-	51937322	29	+	IQ domain-containing protein F1 [Source:UniProtKB/Swiss-Prot;Acc:Q8N6M8]
ENSG00000115474	KCNJ13	ENST00000413607	protein_coding	233635873	2,34E+08	-	2,34E+08	58	+	Inward rectifier potassium channel 13 (Potassium channel, inwardly rectifying subfamily J member 13)(Inward rectifier K(+) channel Kir7.1) [Source:UniProtKB/Swiss-Prot;Acc:O60928]
ENSG00000164659	KIAA1324L	ENST00000425689	protein_coding	86595092	86595291	-	86595222	69	+	UPF0577 protein KIAA1324-like Precursor (Estrogen-induced gene 121-like protein)(hEIG121L) [Source:UniProtKB/Swiss-Prot;Acc:A8MWY0]
ENSG00000170903	KIAA1826	ENST00000313794	protein_coding	105881111	1,06E+08	-	1,06E+08	74	+	Coiled-coil domain-containing protein KIAA1826 [Source:UniProtKB/Swiss-Prot;Acc:Q8NCY6]
ENSG00000169035	KLK7	ENST00000304045	protein_coding	51486951	51487150	-	51487116	34	+	Kallikrein-7 Precursor (hK7)(EC 3.4.21.117)(Stratum corneum chymotryptic enzyme)(hSCCE)(Serine protease 6) [Source:UniProtKB/Swiss-Prot;Acc:P49862]
ENSG00000186393	KRT26	ENST00000335552	protein_coding	38928212	38928411	-	38928331	80	+	Keratin, type I cytoskeletal 26 (Cytokeratin-26)(CK-26)(Keratin-26)(K26)(Type I inner root sheath-specific keratin-K25irs2)(Keratin 25B) [Source:UniProtKB/Swiss-Prot;Acc:Q7Z3Y9]
ENSG00000154655	L3MBTL4	ENST00000400105	protein_coding	6367483	6367682	-	6367583	99	+	Lethal(3)malignant brain tumor-like 4 protein (L(3)mbt-like 4 protein)(H-l(3)mbt-like protein) [Source:UniProtKB/Swiss-Prot;Acc:Q8NA19]
ENSG00000166477	LEO1	ENST00000299601	protein_coding	52263780	52263979	-	52263934	45	+	RNA polymerase-associated protein LEO1 (Replicative senescence down-regulated leo1-like protein) [Source:UniProtKB/Swiss-Prot;Acc:Q8WVC0]
ENSG00000166477	LEO1	ENST00000315141	protein_coding	52263780	52263979	-	52263934	45	+	RNA polymerase-associated protein LEO1 (Replicative senescence down-regulated leo1-like protein) [Source:UniProtKB/Swiss-Prot;Acc:Q8WVC0]
ENSG00000184574	LPAR5	ENST00000329858	protein_coding	6745098	6745297	-	6745198	99	+	Lysophosphatidic acid receptor 5 (LPA receptor 5)(LPA-5)(G-protein coupled receptor 92)(G-protein coupled receptor 93) [Source:UniProtKB/Swiss-Prot;Acc:Q9H1C0]
ENSG00000168702	LRP1B	ENST00000437977	protein_coding	141115438	1,41E+08	-	1,41E+08	80	+	Low-density lipoprotein receptor-related protein 1B Precursor (Low-density lipoprotein receptor-related protein-deleted in tumor)(LRP-DIT) [Source:UniProtKB/Swiss-Prot;Acc:Q9NZR2]
ENSG00000157625	MAP3K7IP3	ENST00000452127	protein_coding	30870758	30870957	-	30870876	81	+	Mitogen-activated protein kinase kinase kinase 7-interacting protein 3 (TAK1-binding protein 3)(NF-kappa-B-activating protein 1) [Source:UniProtKB/Swiss-Prot;Acc:Q8N5C8]
ENSG00000145416	MARCH1	ENST00000274056	protein_coding	164775084	1,65E+08	-	1,65E+08	80	+	E3 ubiquitin-protein ligase MARCH1 (EC 6.3.2.-)(Membrane-associated RING finger protein 1)(Membrane-associated RING-CH protein I)(MARCH-I)(RING finger protein 171) [Source:UniProtKB/Swiss-Prot;Acc:Q8TCQ1]
ENSG00000074416	MGLL	ENST00000398101	protein_coding	127454502	1,27E+08	-	1,27E+08	22	+	Monoglyceride lipase (MGL)(EC 3.1.1.23)(Lysophospholipase homolog)(Lysophospholipase-like)(HU-K5) [Source:UniProtKB/Swiss-Prot;Acc:Q99685]
ENSG00000072952	MRVI1	ENST00000308763	protein_coding	10673649	10673848	-	10673758	90	+	Protein MRVI1 (Inositol 1,4,5-triphosphate receptor-associated

ENSEMBL55 GeneID	Gene Symbol	ENSEMBL55 TranscriptID	Biotype	Gene start	Gene end	Strand	Motif start	TSS offset	Motif strand	Description
										cGMP kinase substrate)(JAW1-related protein MRVI1) [Source:UniProtKB/Swiss-Prot;Acc:Q9Y6F6]
ENSG00000072952	MRVI1	ENST00000421747	protein_coding	10673649	10673848	-	10673758	90	+	Protein MRVI1 (Inositol 1,4,5-triphosphate receptor-associated cGMP kinase substrate)(JAW1-related protein MRVI1) [Source:UniProtKB/Swiss-Prot;Acc:Q9Y6F6]
ENSG00000072952	MRVI1	ENST00000436272	protein_coding	10673649	10673848	-	10673758	90	+	Protein MRVI1 (Inositol 1,4,5-triphosphate receptor-associated cGMP kinase substrate)(JAW1-related protein MRVI1) [Source:UniProtKB/Swiss-Prot;Acc:Q9Y6F6]
ENSG00000166866	MYO1A	ENST00000442789	protein_coding	57444350	57444549	-	57444452	97	+	Myosin-Ia (Brush border myosin I)(BBM-I)(BBMI)(Myosin I heavy chain)(MIHC) [Source:UniProtKB/Swiss-Prot;Acc:Q9UBC5]
ENSG00000164600	NEUROD6	ENST00000297142	protein_coding	31380339	31380538	-	31380448	90	+	Neurogenic differentiation factor 6 (NeuroD6)(Protein atonal homolog 2) [Source:UniProtKB/Swiss-Prot;Acc:Q96NK8]
ENSG00000135540	NHSL1	ENST00000343505	protein_coding	138820380	1,39E+08	-	1,39E+08	31	+	NHS-like protein 1 [Source:UniProtKB/Swiss-Prot;Acc:Q5SYE7]
ENSG00000173572	NLRP13	ENST00000342929	protein_coding	56443503	56443702	-	56443603	99	+	NACHT, LRR and PYD domains-containing protein 13 (Nucleotide-binding oligomerization domain protein 14) [Source:UniProtKB/Swiss-Prot;Acc:Q86W25]
ENSG00000167634	NLRP7	ENST00000448121	protein_coding	55477412	55477611	-	55477538	73	+	NACHT, LRR and PYD domains-containing protein 7 (PYRIN-containing APAF1-like protein 3)(Nucleotide-binding oligomerization domain protein 12) [Source:UniProtKB/Swiss-Prot;Acc:Q8WX94]
ENSG00000167634	NLRP7	ENST00000446217	protein_coding	55477412	55477611	-	55477538	73	+	NACHT, LRR and PYD domains-containing protein 7 (PYRIN-containing APAF1-like protein 3)(Nucleotide-binding oligomerization domain protein 12) [Source:UniProtKB/Swiss-Prot;Acc:Q8WX94]
ENSG00000167634	NLRP7	ENST00000399724	protein_coding	55477412	55477611	-	55477538	73	+	NACHT, LRR and PYD domains-containing protein 7 (PYRIN-containing APAF1-like protein 3)(Nucleotide-binding oligomerization domain protein 12) [Source:UniProtKB/Swiss-Prot;Acc:Q8WX94]
ENSG00000167634	NLRP7	ENST00000427260	protein_coding	55477412	55477611	-	55477538	73	+	NACHT, LRR and PYD domains-containing protein 7 (PYRIN-containing APAF1-like protein 3)(Nucleotide-binding oligomerization domain protein 12) [Source:UniProtKB/Swiss-Prot;Acc:Q8WX94]
ENSG00000198157	NSBP1	ENST00000358130	protein_coding	80457242	80457441	-	80457400	41	+	Nucleosome-binding protein 1 [Source:UniProtKB/Swiss-Prot;Acc:P82970]
ENSG00000076685	NT5C2	ENST00000458345	protein_coding	104866208	1,05E+08	-	1,05E+08	86	+	Cytosolic purine 5'-nucleotidase (EC 3.1.3.5)(5'-nucleotidase cytosolic II) [Source:UniProtKB/Swiss-Prot;Acc:P49902]
ENSG00000113569	NUP155	ENST00000381843	protein_coding	37370688	37370887	-	37370820	67	+	Nuclear pore complex protein Nup155 (Nucleoporin Nup155)(155 kDa nucleoporin) [Source:UniProtKB/Swiss-Prot;Acc:O75694]
ENSG00000075188	NUP37	ENST00000251074	protein_coding	102512162	1,03E+08	-	1,03E+08	76	+	Nucleoporin Nup37 (p37) [Source:UniProtKB/Swiss-Prot;Acc:Q8NFH4]
ENSG00000181355	OFCC1	ENST00000327391	protein_coding	9933470	9933669	-	9933589	80	+	Orofacial cleft 1 candidate gene 1 protein (Orofacial clefting chromosomal breakpoint region candidate 1 protein) [Source:UniProtKB/Swiss-Prot;Acc:Q8IZS5]

ENSEMBL55 GeneID	Gene Symbol	ENSEMBL55 TranscriptID	Biotype	Gene start	Gene end	Strand	Motif start	TSS offset	Motif strand	Description
ENSG00000181355	OFCC1	ENST00000328225	protein_coding	9933470	9933669	-	9933589	80	+	Orofacial cleft 1 candidate gene 1 protein (Orofacial clefting chromosomal breakpoint region candidate 1 protein) [Source:UniProtKB/Swiss-Prot;Acc:Q8IZS5]
ENSG00000204246	OR13C3	ENST00000374780	protein_coding	107298805	1,07E+08	-	1,07E+08	67	+	Olfactory receptor 13C3 (Olfactory receptor OR9-8) [Source:UniProtKB/Swiss-Prot;Acc:Q8NGS6]
ENSG00000072682	P4HA2	ENST00000395164	protein_coding	131557585	1,32E+08	-	1,32E+08	43	+	Prolyl 4-hydroxylase subunit alpha-2 Precursor (EC 1.14.11.2)(4-PH alpha-2)(Procollagen-proline,2-oxoglutarate-4-dioxygenase subunit alpha-2) [Source:UniProtKB/Swiss-Prot;Acc:O15460]
ENSG00000196696	PDXDC2	ENST00000325845	protein_coding	70078144	70078343	-	70078322	21	+	Pyridoxal-dependent decarboxylase domain-containing protein 2 (EC 4.1.1.-) [Source:UniProtKB/Swiss-Prot;Acc:Q6P474]
ENSG00000179094	PER1	ENST00000354903	protein_coding	8059479	8059678	-	8059588	90	+	Period circadian protein homolog 1 (Circadian clock protein PERIOD 1)(Circadian pacemaker protein Rigui)(hPER1) [Source:UniProtKB/Swiss-Prot;Acc:O15534]
ENSG00000096088	PGC	ENST00000415707	protein_coding	41721648	41721847	-	41721759	88	+	Gastricsin Precursor (EC 3.4.23.3)(Pepsinogen C) [Source:UniProtKB/Swiss-Prot;Acc:P20142]
ENSG00000173889	PHC3	ENST00000454294	protein_coding	169899317	1,7E+08	-	1,7E+08	50	+	Polyhomeotic-like protein 3 (Homolog of polyhomeotic 3)(hPH3)(Early development regulatory protein 3) [Source:UniProtKB/Swiss-Prot;Acc:Q8NDX5]
ENSG00000173889	PHC3	ENST00000436251	protein_coding	169899317	1,7E+08	-	1,7E+08	50	+	Polyhomeotic-like protein 3 (Homolog of polyhomeotic 3)(hPH3)(Early development regulatory protein 3) [Source:UniProtKB/Swiss-Prot;Acc:Q8NDX5]
ENSG00000173889	PHC3	ENST00000308547	protein_coding	169899338	1,7E+08	-	1,7E+08	71	+	Polyhomeotic-like protein 3 (Homolog of polyhomeotic 3)(hPH3)(Early development regulatory protein 3) [Source:UniProtKB/Swiss-Prot;Acc:Q8NDX5]
ENSG00000173889	PHC3	ENST00000413540	protein_coding	169899317	1,7E+08	-	1,7E+08	50	+	Polyhomeotic-like protein 3 (Homolog of polyhomeotic 3)(hPH3)(Early development regulatory protein 3) [Source:UniProtKB/Swiss-Prot;Acc:Q8NDX5]
ENSG00000173889	PHC3	ENST00000447576	protein_coding	169899317	1,7E+08	-	1,7E+08	50	+	Polyhomeotic-like protein 3 (Homolog of polyhomeotic 3)(hPH3)(Early development regulatory protein 3) [Source:UniProtKB/Swiss-Prot;Acc:Q8NDX5]
ENSG00000116793	PHTF1	ENST00000412670	protein_coding	114255751	1,14E+08	-	1,14E+08	66	+	Putative homeodomain transcription factor 1 [Source:UniProtKB/Swiss-Prot;Acc:Q9UMS5]
ENSG00000100038	PI4KA	ENST00000411672	protein_coding	22328576	22328775	-	22328736	39	+	Phosphatidylinositol 4-kinase alpha (PI4-kinase alpha)(PtdIns-4-kinase alpha)(PI4K-alpha)(EC 2.7.1.67) [Source:UniProtKB/Swiss-Prot;Acc:P42356]
ENSG00000051382	PIK3CB	ENST00000289153	protein_coding	138477986	1,38E+08	-	1,38E+08	80	+	Phosphatidylinositol-4,5-bisphosphate 3-kinase catalytic subunit beta isoform (EC 2.7.1.153)(PI3-kinase p110 subunit beta)(PtdIns-3-kinase p110)(PI3Kbeta)(PI3K) [Source:UniProtKB/Swiss-Prot;Acc:P42338]
ENSG00000141506	PIK3R5	ENST00000444598	protein_coding	8814635	8814834	-	8814810	24	+	Phosphoinositide 3-kinase regulatory subunit 5 (PI3-kinase regulatory subunit 5)(PI3-kinase p101 subunit)(PtdIns-3-kinase p101)(p101-PI3K)(Phosphatidylinositol-4,5-bisphosphate 3-kinase regulatory subunit)(PtdIns-3-kinase regulatory subunit)(Protein FOAP-2) [Source:UniProtKB/Swiss-Prot;Acc:Q8WYR1]

ENSEMBL55 GeneID	Gene Symbol	ENSEMBL55 TranscriptID	Biotype	Gene start	Gene end	Strand	Motif start	TSS offset	Motif strand	Description
ENSG00000107959	PITRM1	ENST00000430362	protein_coding	3200951	3201150	-	3201083	67	+	Presequence protease, mitochondrial Precursor (hPreP)(EC 3.4.24.-)(Pitrilysin metalloproteinase 1)(Metalloprotease 1)(hMP1) [Source:UniProtKB/Swiss-Prot;Acc:Q5JRX3]
ENSG00000143850	PLEKHA6	ENST00000272203	protein_coding	204328845	2,04E+08	-	2,04E+08	87	+	Pleckstrin homology domain-containing family A member 6 (Phosphoinositol 3-phosphate-binding protein 3)(PEPP-3) [Source:UniProtKB/Swiss-Prot;Acc:Q9Y2H5]
ENSG00000143850	PLEKHA6	ENST00000414478	protein_coding	204328845	2,04E+08	-	2,04E+08	82	+	Pleckstrin homology domain-containing family A member 6 (Phosphoinositol 3-phosphate-binding protein 3)(PEPP-3) [Source:UniProtKB/Swiss-Prot;Acc:Q9Y2H5]
ENSG00000143850	PLEKHA6	ENST00000414478	protein_coding	204328845	2,04E+08	-	2,04E+08	87	+	Pleckstrin homology domain-containing family A member 6 (Phosphoinositol 3-phosphate-binding protein 3)(PEPP-3) [Source:UniProtKB/Swiss-Prot;Acc:Q9Y2H5]
ENSG00000143850	PLEKHA6	ENST00000272203	protein_coding	204328845	2,04E+08	-	2,04E+08	82	+	Pleckstrin homology domain-containing family A member 6 (Phosphoinositol 3-phosphate-binding protein 3)(PEPP-3) [Source:UniProtKB/Swiss-Prot;Acc:Q9Y2H5]
ENSG00000221866	PLXNA4	ENST00000251675	protein_coding	131833216	1,32E+08	-	1,32E+08	50	+	Plexin-A4 Precursor [Source:UniProtKB/Swiss-Prot;Acc:Q9HCM2]
ENSG00000221866	PLXNA4	ENST00000251675	protein_coding	131833216	1,32E+08	-	1,32E+08	80	+	Plexin-A4 Precursor [Source:UniProtKB/Swiss-Prot;Acc:Q9HCM2]
ENSG00000184271	POU6F1	ENST00000389243	protein_coding	51591751	51591950	-	51591868	82	+	POU domain, class 6, transcription factor 1 (mPOU homeobox protein)(Brain-specific homeobox/POU domain protein 5)(Brain-5)(Brn-5 protein) [Source:UniProtKB/Swiss-Prot;Acc:Q14863]
ENSG00000204569	PPP1R10	ENST00000376511	protein_coding	30584821	30585020	-	30584922	98	+	Serine/threonine-protein phosphatase 1 regulatory subunit 10 (Phosphatase 1 nuclear targeting subunit)(MHC class I region proline-rich protein CAT53)(FB19 protein)(PP1-binding protein of 114 kDa)(p99) [Source:UniProtKB/Swiss-Prot;Acc:Q96QC0]
ENSG00000104881	PPP1R13L	ENST00000416875	protein_coding	45908113	45908312	-	45908259	53	+	RelA-associated inhibitor (Inhibitor of ASPP protein)(Protein iASPP)(PPP1R13B-like protein)(NFkB-interacting protein 1) [Source:UniProtKB/Swiss-Prot;Acc:Q8WUF5]
ENSG00000104881	PPP1R13L	ENST00000360957	protein_coding	45908113	45908312	-	45908259	53	+	RelA-associated inhibitor (Inhibitor of ASPP protein)(Protein iASPP)(PPP1R13B-like protein)(NFkB-interacting protein 1) [Source:UniProtKB/Swiss-Prot;Acc:Q8WUF5]
ENSG00000154415	PPP1R3A	ENST00000284601	protein_coding	113558883	1,14E+08	-	1,14E+08	69	+	Protein phosphatase 1 regulatory subunit 3A (Protein phosphatase 1 glycogen-associated regulatory subunit)(Protein phosphatase type-1 glycogen targeting subunit) [Source:UniProtKB/Swiss-Prot;Acc:Q16821]
ENSG00000154415	PPP1R3A	ENST00000284602	protein_coding	113558852	1,14E+08	-	1,14E+08	38	+	Protein phosphatase 1 regulatory subunit 3A (Protein phosphatase 1 glycogen-associated regulatory subunit)(Protein phosphatase type-1 glycogen targeting subunit) [Source:UniProtKB/Swiss-Prot;Acc:Q16821]
ENSG00000185686	PRAME	ENST00000403441	protein_coding	22899975	22900174	-	22900137	37	+	Melanoma antigen preferentially expressed in tumors (Preferentially expressed antigen of melanoma)(OPA-interacting protein 4)(OIP4) [Source:UniProtKB/Swiss-Prot;Acc:P78395]
ENSG00000204491	PRAMEF18	ENST00000376126	protein_coding	13477370	13477569	-	13477521	48	+	PRAME family member 18 [Source:UniProtKB/Swiss-Prot;Acc:Q5VWM3]
ENSG00000204480	PRAMEF19	ENST00000332467	protein_coding	13698206	13698405	-	13698357	48	+	PRAME family member 19 [Source:UniProtKB/Swiss-Prot;Acc:Q5SWL8]

ENSEMBL55 GeneID	Gene Symbol	ENSEMBL55 TranscriptID	Biotype	Gene start	Gene end	Strand	Motif start	TSS offset	Motif strand	Description
ENSG00000204480	PRAMEF19	ENST00000376101	protein_coding	13698206	13698405	-	13698357	48	+	PRAME family member 19 [Source:UniProtKB/Swiss-Prot;Acc:Q5SWL8]
ENSG00000204503	PRAMEF3	ENST00000376173	protein_coding	13331516	13331715	-	13331664	51	+	PRAME family member 3 [Source:UniProtKB/Swiss-Prot;Acc:Q5TYW8]
ENSG00000204503	PRAMEF3	ENST00000353410	protein_coding	13331493	13331692	-	13331664	28	+	PRAME family member 3 [Source:UniProtKB/Swiss-Prot;Acc:Q5TYW8]
ENSG00000183463	PRHOXNB	ENST00000332715	protein_coding	28562575	28562774	-	28562692	82	+	Putative 2-oxo-4-hydroxy-4-carboxy-5-ureidoimidazoline decarboxylase (OHCU decarboxylase)(EC 4.1.1.n1)(Parahox neighbor) [Source:UniProtKB/Swiss-Prot;Acc:A6NGE7]
ENSG00000114302	PRKAR2A	ENST00000437821	protein_coding	48827862	48828061	-	48828030	31	+	cAMP-dependent protein kinase type II-alpha regulatory subunit [Source:UniProtKB/Swiss-Prot;Acc:P13861]
ENSG00000115825	PRKD3	ENST00000379066	protein_coding	37544838	37545037	-	37544988	49	+	Serine/threonine-protein kinase D3 (EC 2.7.11.13)(Protein kinase C nu type)(nPKC-nu)(Protein kinase EPK2) [Source:UniProtKB/Swiss-Prot;Acc:O94806]
ENSG00000167525	PROCA1	ENST00000415329	protein_coding	27038479	27038678	-	27038594	84	+	Protein PROCA1 [Source:UniProtKB/Swiss-Prot;Acc:Q8NCQ7]
ENSG00000213005	PTTG3	ENST00000391687	protein_coding	67680041	67680240	-	67680217	23	+	Securin-3 (Pituitary tumor transforming gene 3 protein)(hPTTG3)(rcPTTG1) [Source:UniProtKB/Swiss-Prot;Acc:Q9NZH4]
ENSG00000100362	PVALB	ENST00000216200	protein_coding	37215318	37215517	-	37215428	89	+	Parvalbumin alpha [Source:UniProtKB/Swiss-Prot;Acc:P20472]
ENSG00000100362	PVALB	ENST00000442775	protein_coding	37215318	37215517	-	37215428	89	+	Parvalbumin alpha [Source:UniProtKB/Swiss-Prot;Acc:P20472]
ENSG00000128340	RAC2	ENST00000417990	protein_coding	37639989	37640188	-	37640102	86	+	Ras-related C3 botulinum toxin substrate 2 Precursor (p21-Rac2)(Small G protein)(GX) [Source:UniProtKB/Swiss-Prot;Acc:P15153]
ENSG00000128340	RAC2	ENST00000458421	protein_coding	37639995	37640194	-	37640102	92	+	Ras-related C3 botulinum toxin substrate 2 Precursor (p21-Rac2)(Small G protein)(GX) [Source:UniProtKB/Swiss-Prot;Acc:P15153]
ENSG00000155918	RAET1L	ENST00000286380	protein_coding	150346469	1,5E+08	-	1,5E+08	27	+	Retinoic acid early transcript 1L protein Precursor [Source:UniProtKB/Swiss-Prot;Acc:Q5VY80]
ENSG00000100320	RBM9	ENST00000408983	protein_coding	36357515	36357714	-	36357641	73	+	RNA-binding protein 9 (RNA-binding motif protein 9)(Fox-1 homolog B)(Hexaribonucleotide-binding protein 2)(Repressor of tamoxifen transcriptional activity) [Source:UniProtKB/Swiss-Prot;Acc:O43251]
ENSG00000143839	REN	ENST00000272190	protein_coding	204135266	2,04E+08	-	2,04E+08	63	+	Renin Precursor (EC 3.4.23.15)(Angiotensinogenase) [Source:UniProtKB/Swiss-Prot;Acc:P00797]
ENSG00000143839	REN	ENST00000367195	protein_coding	204135266	2,04E+08	-	2,04E+08	63	+	Renin Precursor (EC 3.4.23.15)(Angiotensinogenase) [Source:UniProtKB/Swiss-Prot;Acc:P00797]
ENSG00000135597	REPS1	ENST00000415951	protein_coding	139264840	1,39E+08	-	1,39E+08	40	+	RalBP1-associated Eps domain-containing protein 1 (RalBP1-interacting protein 1) [Source:UniProtKB/Swiss-Prot;Acc:Q96D71]
ENSG00000135597	REPS1	ENST00000414243	protein_coding	139264864	1,39E+08	-	1,39E+08	64	+	RalBP1-associated Eps domain-containing protein 1 (RalBP1-interacting protein 1) [Source:UniProtKB/Swiss-Prot;Acc:Q96D71]
ENSG00000131378	RFTN1	ENST00000432519	protein_coding	16524173	16524372	-	16524278	94	+	Raftlin (Raft-linking protein)(Cell migration-inducing gene 2 protein) [Source:UniProtKB/Swiss-Prot;Acc:Q14699]
ENSG00000120555	RP11-291L22.2	ENST00000328105	protein_coding	38681841	38682040	-	38681968	72	+	Novel protein [Source:UniProtKB/TrEMBL;Acc:Q5W161]

ENSEMBL55 GeneID	Gene Symbol	ENSEMBL55 TranscriptID	Biotype	Gene start	Gene end	Strand	Motif start	TSS offset	Motif strand	Description
ENSG00000165837	RP11-351K3.2	ENST00000298738	protein_coding	46189675	46189874	-	46189843	31	+	Putative uncharacterized protein FLJ32682 [Source:UniProtKB/Swiss-Prot;Acc:Q5W0A0]
ENSG00000183291	RP4-604K5.1	ENST00000331835	protein_coding	87379849	87380048	-	87379949	99	+	15 kDa selenoprotein Precursor [Source:UniProtKB/Swiss-Prot;Acc:O60613]
ENSG00000103932	RPAP1	ENST00000304330	protein_coding	41836265	41836464	-	41836380	84	+	RNA polymerase II-associated protein 1 [Source:UniProtKB/Swiss-Prot;Acc:Q9BWH6]
ENSG00000103494	RPGRIP1L	ENST00000379925	protein_coding	53737572	53737771	-	53737687	84	+	Protein fantom (RPGR-interacting protein 1-like protein)(RPGRIP1-like protein) [Source:UniProtKB/Swiss-Prot;Acc:Q68CZ1]
ENSG00000103494	RPGRIP1L	ENST00000262135	protein_coding	53737572	53737771	-	53737687	84	+	Protein fantom (RPGR-interacting protein 1-like protein)(RPGRIP1-like protein) [Source:UniProtKB/Swiss-Prot;Acc:Q68CZ1]
ENSG00000144713	RPL32	ENST00000434963	protein_coding	12882182	12882381	-	12882294	87	+	60S ribosomal protein L32 [Source:UniProtKB/Swiss-Prot;Acc:P62910]
ENSG00000020633	RUNX3	ENST00000308873	protein_coding	25256571	25256770	-	25256748	22	+	Runt-related transcription factor 3 (Core-binding factor subunit alpha-3)(CBF-alpha-3)(Acute myeloid leukemia 2 protein)(Oncogene AML-2)(Polyomavirus enhancer-binding protein 2 alpha C subunit)(PEBP2-alpha C)(PEA2-alpha C)(SL3-3 enhancer factor 1 alpha C subunit)(SL3/AKV core-binding factor alpha C subunit) [Source:UniProtKB/Swiss-Prot;Acc:Q13761]
ENSG00000196420	S100A5	ENST00000368717	protein_coding	153512971	1,54E+08	-	1,54E+08	78	+	Protein S100-A5 (S100 calcium-binding protein A5)(Protein S-100D) [Source:UniProtKB/Swiss-Prot;Acc:P33763]
ENSG00000119042	SATB2	ENST00000457245	protein_coding	200335790	2E+08	-	2E+08	34	+	DNA-binding protein SATB2 (Special AT-rich sequence-binding protein 2) [Source:UniProtKB/Swiss-Prot;Acc:Q9UPW6]
ENSG00000140386	SCAPER	ENST00000324767	protein_coding	77175993	77176192	-	77176144	48	+	S phase cyclin A-associated protein in the endoplasmic reticulum (S phase cyclin A-associated protein in the ER)(Zinc finger protein 291) [Source:UniProtKB/Swiss-Prot;Acc:Q9BY12]
ENSG00000025796	SEC63	ENST00000437345	protein_coding	108224330	1,08E+08	-	1,08E+08	75	+	Translocation protein SEC63 homolog [Source:UniProtKB/Swiss-Prot;Acc:Q9UGP8]
ENSG00000123561	SERPINA7	ENST00000372563	protein_coding	105282530	1,05E+08	-	1,05E+08	25	+	Thyroxine-binding globulin Precursor (T4-binding globulin)(Serpin A7) [Source:UniProtKB/Swiss-Prot;Acc:P05543]
ENSG00000117601	SERPINC1	ENST00000351522	protein_coding	173886274	1,74E+08	-	1,74E+08	32	+	Antithrombin-III Precursor (ATIII) [Source:UniProtKB/Swiss-Prot;Acc:P01008]
ENSG00000117601	SERPINC1	ENST00000367698	protein_coding	173886274	1,74E+08	-	1,74E+08	32	+	Antithrombin-III Precursor (ATIII) [Source:UniProtKB/Swiss-Prot;Acc:P01008]
ENSG00000136527	SFRS10	ENST00000414862	protein_coding	185639696	1,86E+08	-	1,86E+08	34	+	Transformer-2 protein homolog beta (HTRA2-beta)(Transformer-2 protein homolog B)(Splicing factor, arginine/serine-rich 10) [Source:UniProtKB/Swiss-Prot;Acc:P62995]
ENSG00000135917	SLC19A3	ENST00000419059	protein_coding	228567494	2,29E+08	-	2,29E+08	42	+	Thiamine transporter 2 (ThTr-2)(ThTr2)(Solute carrier family 19 member 3) [Source:UniProtKB/Swiss-Prot;Acc:Q9BZV2]
ENSG00000164609	SLU7	ENST00000297151	protein_coding	159845969	1,6E+08	-	1,6E+08	99	+	Pre-mRNA-splicing factor SLU7 (hSlu7) [Source:UniProtKB/Swiss-Prot;Acc:O95391]
ENSG00000012817	SMCY	ENST00000447300	protein_coding	19256878	19257077	-	19257027	50	+	Histone demethylase JARID1D (EC 1.14.11.-)(Jumonji/ARID domain-containing protein 1D)(Protein SmcY)(Histocompatibility

ENSEMBL55 GeneID	Gene Symbol	ENSEMBL55 TranscriptID	Biotype	Gene start	Gene end	Strand	Motif start	TSS offset	Motif strand	Description
										Y antigen)(H-Y) [Source:UniProtKB/Swiss-Prot;Acc:Q9BY66]
ENSG00000204363	SPANXN5	ENST00000375511	protein_coding	52826189	52826388	-	52826307	81	+	Sperm protein associated with the nucleus on the X chromosome N5 (SPANX-N5)(SPANX family member N5) [Source:UniProtKB/Swiss-Prot;Acc:Q5MJ07]
ENSG00000160828	STAG3L2	ENST00000456236	protein_coding	74301017	74301216	-	74301130	86	+	STAG3-like protein (Stromal antigen 3-like) [Source:UniProtKB/Swiss-Prot;Acc:Q6NXR2]
ENSG00000100109	TFIP11	ENST00000458588	protein_coding	26906474	26906673	-	26906601	72	+	Tuftelin-interacting protein 11 (Septin and tuftelin-interacting protein 1)(STIP-1) [Source:UniProtKB/Swiss-Prot;Acc:Q9UBB9]
ENSG00000100109	TFIP11	ENST00000412662	protein_coding	26906474	26906673	-	26906601	72	+	Tuftelin-interacting protein 11 (Septin and tuftelin-interacting protein 1)(STIP-1) [Source:UniProtKB/Swiss-Prot;Acc:Q9UBB9]
ENSG00000100109	TFIP11	ENST00000263115	protein_coding	26906474	26906673	-	26906601	72	+	Tuftelin-interacting protein 11 (Septin and tuftelin-interacting protein 1)(STIP-1) [Source:UniProtKB/Swiss-Prot;Acc:Q9UBB9]
ENSG00000100109	TFIP11	ENST00000448938	protein_coding	26906469	26906668	-	26906601	67	+	Tuftelin-interacting protein 11 (Septin and tuftelin-interacting protein 1)(STIP-1) [Source:UniProtKB/Swiss-Prot;Acc:Q9UBB9]
ENSG00000100296	THOC5	ENST00000435403	protein_coding	29944940	29945139	-	29945086	53	+	THO complex subunit 5 homolog (NF2/meningioma region protein pK1.3)(Placental protein 39.2) [Source:UniProtKB/Swiss-Prot;Acc:Q13769]
ENSG00000151090	THRB	ENST00000416811	protein_coding	24270298	24270497	-	24270460	37	+	Thyroid hormone receptor beta (Nuclear receptor subfamily 1 group A member 2) [Source:UniProtKB/Swiss-Prot;Acc:P10828]
ENSG00000151353	TMEM18	ENST00000405941	protein_coding	676440	676639	-	676550	89	+	Transmembrane protein 18 [Source:UniProtKB/Swiss-Prot;Acc:Q96B42]
ENSG00000151353	TMEM18	ENST00000418447	protein_coding	676443	676642	-	676550	92	+	Transmembrane protein 18 [Source:UniProtKB/Swiss-Prot;Acc:Q96B42]
ENSG00000142188	TMEM50B	ENST00000435619	protein_coding	34853300	34853499	-	34853413	86	+	Transmembrane protein 50B (HCV p7-trans-regulated protein 3) [Source:UniProtKB/Swiss-Prot;Acc:P56557]
ENSG00000184090	TMEM75	ENST00000328323	protein_coding	128960081	1,29E+08	-	1,29E+08	27	+	Transmembrane protein 75 [Source:UniProtKB/Swiss-Prot;Acc:Q8N9X5]
ENSG00000137747	TMPRSS13	ENST00000278940	protein_coding	117790349	1,18E+08	-	1,18E+08	48	+	Transmembrane protease, serine 13 (EC 3.4.21.-)(Membrane-type mosaic serine protease)(Mosaic serine protease) [Source:UniProtKB/Swiss-Prot;Acc:Q9BYE2]
ENSG00000137747	TMPRSS13	ENST00000445164	protein_coding	117790349	1,18E+08	-	1,18E+08	48	+	Transmembrane protease, serine 13 (EC 3.4.21.-)(Membrane-type mosaic serine protease)(Mosaic serine protease) [Source:UniProtKB/Swiss-Prot;Acc:Q9BYE2]
ENSG00000137747	TMPRSS13	ENST00000413475	protein_coding	117790349	1,18E+08	-	1,18E+08	48	+	Transmembrane protease, serine 13 (EC 3.4.21.-)(Membrane-type mosaic serine protease)(Mosaic serine protease) [Source:UniProtKB/Swiss-Prot;Acc:Q9BYE2]
ENSG00000137747	TMPRSS13	ENST00000430170	protein_coding	117790349	1,18E+08	-	1,18E+08	48	+	Transmembrane protease, serine 13 (EC 3.4.21.-)(Membrane-type mosaic serine protease)(Mosaic serine protease) [Source:UniProtKB/Swiss-Prot;Acc:Q9BYE2]
ENSG00000128438	TNFRSF13B	ENST00000261651	protein_coding	16837954	16838153	-	16838120	33	+	Tumor necrosis factor receptor superfamily member 13B (Transmembrane activator and CAML interactor)(CD267 antigen) [Source:UniProtKB/Swiss-Prot;Acc:O14836]
ENSG00000061938	TNK2	ENST00000427576	protein_coding	195623455	1,96E+08	-	1,96E+08	70	+	Activated CDC42 kinase 1 (ACK-1)(EC 2.7.10.2)(Tyrosine kinase non-receptor protein 2) [Source:UniProtKB/Swiss-Prot;Acc:Q07912]
ENSG00000159173	TNNI1	ENST00000361379	protein_coding	201390659	2,01E+08	-	2,01E+08	96	+	Troponin I, slow skeletal muscle (Troponin I, slow-twitch

ENSEMBL55 GeneID	Gene Symbol	ENSEMBL55 TranscriptID	Biotype	Gene start	Gene end	Strand	Motif start	TSS offset	Motif strand	Description
										isoform) [Source:UniProtKB/Swiss-Prot;Acc:P19237]
ENSG00000159173	TNNI1	ENST00000367312	protein_coding	201390647	2,01E+08	-	2,01E+08	84	+	Troponin I, slow skeletal muscle (Troponin I, slow-twitch isoform) [Source:UniProtKB/Swiss-Prot;Acc:P19237]
ENSG00000159409	TNRC4	ENST00000415718	protein_coding	151687968	1,52E+08	-	1,52E+08	92	+	CUG-BP- and ETR-3-like factor 3 (CELF-3)(Bruno-like protein 1)(RNA-binding protein BRUNOL-1)(ELAV-type RNA-binding protein 1)(ETR-1)(Trinucleotide repeat-containing gene 4 protein)(Expanded repeat domain protein CAG/CTG 4)(CAG repeat protein 4) [Source:UniProtKB/Swiss-Prot;Acc:Q5SZQ8]
ENSG00000077097	TOP2B	ENST00000413971	protein_coding	25660141	25660340	-	25660319	21	+	DNA topoisomerase 2-beta (EC 5.99.1.3)(DNA topoisomerase II, beta isozyme) [Source:UniProtKB/Swiss-Prot;Acc:Q02880]
ENSG00000166157	TPTE	ENST00000359693	protein_coding	10971523	10971722	-	10971689	33	+	Putative tyrosine-protein phosphatase TPTE (EC 3.1.3.48)(Transmembrane phosphatase with tensin homology)(Tumor antigen BJ-HCC-5)(Cancer/testis antigen 44)(CT44) [Source:UniProtKB/Swiss-Prot;Acc:P56180]
ENSG00000124641	TRFP	ENST00000423815	protein_coding	41884605	41884804	-	41884754	50	+	Mediator of RNA polymerase II transcription subunit 20 (Mediator complex subunit 20)(TRF-proximal protein homolog)(hTRFP) [Source:UniProtKB/Swiss-Prot;Acc:Q9H944]
ENSG00000134160	TRPM1	ENST00000256552	protein_coding	31362496	31362695	-	31362605	90	+	Transient receptor potential cation channel subfamily M member 1 (Long transient receptor potential channel 1)(LTrpC1)(Melastatin-1) [Source:UniProtKB/Swiss-Prot;Acc:Q7Z4N2]
ENSG00000134160	TRPM1	ENST00000397793	protein_coding	31362496	31362695	-	31362605	90	+	Transient receptor potential cation channel subfamily M member 1 (Long transient receptor potential channel 1)(LTrpC1)(Melastatin-1) [Source:UniProtKB/Swiss-Prot;Acc:Q7Z4N2]
ENSG00000119121	TRPM6	ENST00000449912	protein_coding	77473802	77474001	-	77473958	43	+	Transient receptor potential cation channel subfamily M member 6 (EC 2.7.11.1)(Channel kinase 2)(Melastatin-related TRP cation channel 6) [Source:UniProtKB/Swiss-Prot;Acc:Q9BX84]
ENSG00000104447	TRPS1	ENST00000395713	protein_coding	116673706	1,17E+08	-	1,17E+08	66	+	Zinc finger transcription factor Trps1 (Tricho-rhino-phalangeal syndrome type I protein)(Zinc finger protein GC79) [Source:UniProtKB/Swiss-Prot;Acc:Q9UHF7]
ENSG00000136319	TTC5	ENST00000383029	protein_coding	20773937	20774136	-	20774085	51	+	Tetratricopeptide repeat protein 5 (TPR repeat protein 5) [Source:UniProtKB/Swiss-Prot;Acc:Q8N0Z6]
ENSG00000136319	TTC5	ENST00000258821	protein_coding	20773937	20774136	-	20774085	51	+	Tetratricopeptide repeat protein 5 (TPR repeat protein 5) [Source:UniProtKB/Swiss-Prot;Acc:Q8N0Z6]
ENSG00000155657	TTN	ENST00000446966	protein_coding	179516044	1,8E+08	-	1,8E+08	99	+	titin isoform novex-3 [Source:RefSeq peptide;Acc:NP_596870]
ENSG00000155657	TTN	ENST00000429997	protein_coding	179516044	1,8E+08	-	1,8E+08	99	+	titin isoform novex-3 [Source:RefSeq peptide;Acc:NP_596870]
ENSG00000092929	UNC13D	ENST00000412096	protein_coding	73840599	73840798	-	73840758	40	+	Protein unc-13 homolog D (Munc13-4) [Source:UniProtKB/Swiss-Prot;Acc:Q70J99]
ENSG00000092929	UNC13D	ENST00000207549	protein_coding	73840599	73840798	-	73840758	40	+	Protein unc-13 homolog D (Munc13-4) [Source:UniProtKB/Swiss-Prot;Acc:Q70J99]
ENSG00000092929	UNC13D	ENST00000448606	protein_coding	73840599	73840798	-	73840758	40	+	Protein unc-13 homolog D (Munc13-4) [Source:UniProtKB/Swiss-Prot;Acc:Q70J99]
ENSG00000136014	USP44	ENST00000258499	protein_coding	95945064	95945263	-	95945197	66	+	Ubiquitin carboxyl-terminal hydrolase 44 (EC 3.1.2.15)(Ubiquitin thioesterase 44)(Ubiquitin-specific-processing protease

ENSEMBL55 GeneID	Gene Symbol	ENSEMBL55 TranscriptID	Biotype	Gene start	Gene end	Strand	Motif start	TSS offset	Motif strand	Description
										44)(Deubiquitinating enzyme 44) [Source:UniProtKB/Swiss-Prot;Acc:Q9H0E7]
ENSG00000144560	VGLL4	ENST00000437722	protein_coding	11651779	11651978	-	11651924	54	+	Transcription cofactor vestigial-like protein 4 (Vgl-4) [Source:UniProtKB/Swiss-Prot;Acc:Q14135]
ENSG00000140006	WDR89	ENST00000400511	protein_coding	64107926	64108125	-	64108067	58	+	WD repeat-containing protein 89 [Source:UniProtKB/Swiss-Prot;Acc:Q96FK6]
ENSG00000140006	WDR89	ENST00000267522	protein_coding	64107926	64108125	-	64108067	58	+	WD repeat-containing protein 89 [Source:UniProtKB/Swiss-Prot;Acc:Q96FK6]
ENSG00000169554	ZEB2	ENST00000435831	protein_coding	145275265	1,45E+08	-	1,45E+08	80	+	Zinc finger E-box-binding homeobox 2 (Zinc finger homeobox protein 1b)(Smad-interacting protein 1)(SMADIP1) [Source:UniProtKB/Swiss-Prot;Acc:O60315]
ENSG00000166432	ZMAT1	ENST00000435980	protein_coding	101144994	1,01E+08	-	1,01E+08	78	+	Zinc finger matrin-type protein 1 [Source:UniProtKB/Swiss-Prot;Acc:Q5H9K5]
ENSG00000163867	ZMYM6	ENST00000311990	protein_coding	35444108	35444307	-	35444262	45	+	Zinc finger MYM-type protein 6 (Zinc finger protein 258) [Source:UniProtKB/Swiss-Prot;Acc:O95789]
ENSG00000173276	ZNF295	ENST00000425521	protein_coding	43427967	43428166	-	43428095	71	+	Zinc finger protein 295 (Zinc finger and BTB domain-containing protein 21) [Source:UniProtKB/Swiss-Prot;Acc:Q9ULJ3]
ENSG00000140987	ZNF434	ENST00000422427	protein_coding	3447428	3447627	-	3447605	22	+	Zinc finger protein 434 (Cervical cancer suppressor gene 5 protein)(HCCS-5) [Source:UniProtKB/Swiss-Prot;Acc:Q9NX65]
ENSG00000185219	ZNF445	ENST00000430301	protein_coding	44496993	44497192	-	44497149	43	+	Zinc finger protein 445 (Zinc finger protein 168)(Zinc finger protein with KRAB and SCAN domains 15) [Source:UniProtKB/Swiss-Prot;Acc:P59923]
ENSG00000185219	ZNF445	ENST00000296111	protein_coding	44496993	44497192	-	44497149	43	+	Zinc finger protein 445 (Zinc finger protein 168)(Zinc finger protein with KRAB and SCAN domains 15) [Source:UniProtKB/Swiss-Prot;Acc:P59923]
ENSG00000197124	ZNF682	ENST00000397165	protein_coding	20150078	20150277	-	20150193	84	+	Zinc finger protein 682 [Source:UniProtKB/Swiss-Prot;Acc:O95780]
ENSG00000197124	ZNF682	ENST00000358523	protein_coding	20150078	20150277	-	20150193	84	+	Zinc finger protein 682 [Source:UniProtKB/Swiss-Prot;Acc:O95780]
ENSG00000197782	ZNF780A	ENST00000455521	protein_coding	40596646	40596845	-	40596758	87	+	Zinc finger protein 780A [Source:UniProtKB/Swiss-Prot;Acc:O75290]
ENSG00000197782	ZNF780A	ENST00000392041	protein_coding	40596646	40596845	-	40596758	87	+	Zinc finger protein 780A [Source:UniProtKB/Swiss-Prot;Acc:O75290]
ENSG00000197782	ZNF780A	ENST00000340963	protein_coding	40596646	40596845	-	40596758	87	+	Zinc finger protein 780A [Source:UniProtKB/Swiss-Prot;Acc:O75290]
ENSG00000197782	ZNF780A	ENST00000414720	protein_coding	40596646	40596845	-	40596758	87	+	Zinc finger protein 780A [Source:UniProtKB/Swiss-Prot;Acc:O75290]
ENSG00000197782	ZNF780A	ENST00000443072	protein_coding	40596646	40596845	-	40596758	87	+	Zinc finger protein 780A [Source:UniProtKB/Swiss-Prot;Acc:O75290]
ENSG00000197782	ZNF780A	ENST00000450241	protein_coding	40596611	40596810	-	40596758	52	+	Zinc finger protein 780A [Source:UniProtKB/Swiss-Prot;Acc:O75290]
ENSG00000179869	ABCA13	ENST00000453246	protein_coding	48416039	48416238	+	48416108	69	-	ATP-binding cassette sub-family A member 13 [Source:UniProtKB/Swiss-Prot;Acc:Q86UQ4]
ENSG00000205396	AC004790.1-1	ENST00000379899	protein_coding	16126444	16126643	+	16126542	98	-	hypothetical LOC126536 (LOC126536), non-coding RNA [Source:RefSeq DNA;Acc:NR_026828]
ENSG00000226333	AC006112.4	ENST00000415768	protein_coding	6427904	6428103	+	6427930	26	-	Hypothetical LOC440337cDNA FLJ37013 fis, clone BRACE2010171 ; [Source:UniProtKB/TrEMBL;Acc:Q8N9J9]

ENSEMBL55 GeneID	Gene Symbol	ENSEMBL55 TranscriptID	Biotype	Gene start	Gene end	Strand	Motif start	TSS offset	Motif strand	Description
ENSG00000236914	AC006344.2	ENST00000434223	protein_coding	140471964	1,4E+08	+	1,4E+08	87	-	
ENSG00000225729	AC007421.13-1	ENST00000444551	protein_coding	7512280	7512479	+	7512368	88	-	
ENSG00000229833	AC008763.9	ENST00000456958	protein_coding	7694693	7694892	+	7694786	93	-	
ENSG00000225702	AC010326.7-2	ENST00000416025	protein_coding	58379253	58379452	+	58379279	26	-	Ubiquitin UBF-fl [Source:UniProtKB/TrEMBL;Acc:Q96JB9]
ENSG00000237247	AC010606.7-2	ENST00000329753	protein_coding	7030588	7030787	+	7030678	90	-	Putative methyl-CpG-binding domain protein 3-like 5 (MBD3-like 5) [Source:UniProtKB/Swiss-Prot;Acc:A6NJ08]
ENSG00000237247	AC010606.7-2	ENST00000450263	protein_coding	7030588	7030787	+	7030678	90	-	Putative methyl-CpG-binding domain protein 3-like 5 (MBD3-like 5) [Source:UniProtKB/Swiss-Prot;Acc:A6NJ08]
ENSG00000214694	AC019171.3	ENST00000409978	protein_coding	39117021	39117220	+	39117081	60	-	DH and coiled-coil domain-containing protein ENSP00000381780 [Source:UniProtKB/Swiss-Prot;Acc:A8MVX0]
ENSG00000235365	AC091948.3	ENST00000428129	protein_coding	147698550	1,48E+08	+	1,48E+08	73	-	Vitellogenin-like 1 Fragment [Source:UniProtKB/TrEMBL;Acc:Q6IE27]
ENSG00000230824	AC097469.2	ENST00000419935	protein_coding	67892294	67892493	+	67892335	41	-	
ENSG00000232773	AC116655.7-12	ENST00000426866	protein_coding	9424036	9424235	+	9424098	62	-	
ENSG00000232653	AC123768.8-1	ENST00000426622	protein_coding	32885755	32885954	+	32885843	88	-	Putative golgin subfamily A member 6-like protein 11 [Source:UniProtKB/Swiss-Prot;Acc:A6NCC3]
ENSG00000206193	AC124312.5-2	ENST00000383025	protein_coding	25322013	25322212	+	25322061	48	-	Putative uncharacterized protein DKFZp686M12165 Fragment [Source:UniProtKB/TrEMBL;Acc:Q5HYA3]
ENSG00000215302	AC127502.6	ENST00000399971	protein_coding	30771193	30771392	+	30771250	57	-	
ENSG00000224712	AC136443.3-1	ENST00000413283	protein_coding	14805442	14805641	+	14805470	28	-	Putative uncharacterized protein ENSP00000165086 [Source:UniProtKB/TrEMBL;Acc:A6NCT9]
ENSG00000147174	ACRC	ENST00000373695	protein_coding	70800135	70800334	+	70800234	99	-	Acidic repeat-containing protein [Source:UniProtKB/Swiss-Prot;Acc:Q96QF7]
ENSG00000163017	ACTG2	ENST00000438902	protein_coding	74119390	74119589	+	74119483	93	-	Actin, gamma-enteric smooth muscle (Smooth muscle gamma-actin)(Gamma-2-actin)(Alpha-actin-3) [Source:UniProtKB/Swiss-Prot;Acc:P63267]
ENSG00000078549	ADCYAP1R1	ENST00000436116	protein_coding	31126585	31126784	+	31126658	73	-	Pituitary adenylate cyclase-activating polypeptide type I receptor Precursor (PACAP type I receptor)(PACAP-R-1) [Source:UniProtKB/Swiss-Prot;Acc:P41586]
ENSG00000223846	AL355493.14-1	ENST00000443201	protein_coding	27602673	27602872	+	27602701	28	-	
ENSG00000204038	AL359195.24	ENST00000356374	protein_coding	82010981	82011180	+	82011078	97	-	cDNA FLJ46261 fis, clone TESTI4025062 [Source:UniProtKB/TrEMBL;Acc:Q6ZRL6]
ENSG00000228580	AL513523.33-1	ENST00000433612	protein_coding	153747489	1,54E+08	+	1,54E+08	44	-	Putative uncharacterized protein [Source:UniProtKB/TrEMBL;Acc:Q5K4L7]
ENSG00000226518	AL929601.4-1	ENST00000422907	protein_coding	20085105	20085304	+	20085203	98	-	
ENSG00000011426	ANLN	ENST00000457743	protein_coding	36462332	36462531	+	36462429	97	-	Actin-binding protein anillin [Source:UniProtKB/Swiss-Prot;Acc:Q9NQW6]
ENSG00000155008	APOOL	ENST00000373169	protein_coding	84301307	84301506	+	84301363	56	-	Apolipoprotein O-like Precursor (Protein FAM121A) [Source:UniProtKB/Swiss-Prot;Acc:Q6UXV4]
ENSG00000088756	ARHGAP28	ENST00000418986	protein_coding	6837192	6837391	+	6837245	53	-	Rho GTPase-activating protein 28 (Rho-type GTPase-activating

ENSEMBL55 GeneID	Gene Symbol	ENSEMBL55 TranscriptID	Biotype	Gene start	Gene end	Strand	Motif start	TSS offset	Motif strand	Description
										protein 28) [Source:UniProtKB/Swiss-Prot;Acc:Q9P2N2]
ENSG00000088756	ARHGAP28	ENST00000314319	protein_coding	6837192	6837391	+	6837245	53	-	Rho GTPase-activating protein 28 (Rho-type GTPase-activating protein 28) [Source:UniProtKB/Swiss-Prot;Acc:Q9P2N2]
ENSG00000049618	ARID1B	ENST00000445852	protein_coding	157522173	1,58E+08	+	1,58E+08	52	-	AT-rich interactive domain-containing protein 1B (ARID domain-containing protein 1B)(Osa homolog 2)(hOsa2)(p250R)(BRG1-binding protein hELD/OSA1)(BRG1-associated factor 250b)(BAF250B) [Source:UniProtKB/Swiss-Prot;Acc:Q8NFD5]
ENSG00000162772	ATF3	ENST00000366981	protein_coding	212738676	2,13E+08	+	2,13E+08	52	-	Cyclic AMP-dependent transcription factor ATF-3 (cAMP-dependent transcription factor ATF-3)(Activating transcription factor 3) [Source:UniProtKB/Swiss-Prot;Acc:P18847]
ENSG00000162772	ATF3	ENST00000366987	protein_coding	212738697	2,13E+08	+	2,13E+08	31	-	Cyclic AMP-dependent transcription factor ATF-3 (cAMP-dependent transcription factor ATF-3)(Activating transcription factor 3) [Source:UniProtKB/Swiss-Prot;Acc:P18847]
ENSG00000180389	ATP5EP2	ENST00000381026	protein_coding	28519343	28519542	+	28519437	94	-	ATP synthase subunit epsilon-like protein, mitochondrial [Source:UniProtKB/Swiss-Prot;Acc:Q5VTU8]
ENSG00000105778	AVL9	ENST00000329714	protein_coding	32582753	32582952	+	32582776	23	-	Late secretory pathway protein AVL9 homolog [Source:UniProtKB/Swiss-Prot;Acc:Q8NBF6]
ENSG00000162399	BSND	ENST00000371265	protein_coding	55464617	55464816	+	55464710	93	-	Barttin [Source:UniProtKB/Swiss-Prot;Acc:Q8WZ55]
ENSG00000132640	BTBD3	ENST00000422390	protein_coding	11873141	11873340	+	11873232	91	-	BTB/POZ domain-containing protein 3 [Source:UniProtKB/Swiss-Prot;Acc:Q9Y2F9]
ENSG00000107938	C10orf137	ENST00000433226	protein_coding	127424309	1,27E+08	+	1,27E+08	42	-	Erythroid differentiation-related factor 1 [Source:UniProtKB/Swiss-Prot;Acc:Q3B7T1]
ENSG00000148735	C10orf81	ENST00000369309	protein_coding	115531012	1,16E+08	+	1,16E+08	63	-	hypothetical protein LOC79949 [Source:RefSeq peptide;Acc:NP_079165]
ENSG00000215386	C21orf34	ENST00000441820	protein_coding	17909601	17909800	+	17909634	33	-	hypothetical protein LOC388815 isoform a [Source:RefSeq peptide;Acc:NP_001005732]
ENSG00000138085	C2orf28	ENST00000419744	protein_coding	27435734	27435933	+	27435784	50	-	Apoptosis-related protein 3 Precursor (APR-3)(p18) [Source:UniProtKB/Swiss-Prot;Acc:Q6UW56]
ENSG00000144649	C3orf41	ENST00000434206	protein_coding	43020805	43021004	+	43020870	65	-	Uncharacterized protein C3orf41 Precursor [Source:UniProtKB/Swiss-Prot;Acc:Q9UFP1]
ENSG00000144649	C3orf41	ENST00000434206	protein_coding	43020805	43021004	+	43020880	75	-	Uncharacterized protein C3orf41 Precursor [Source:UniProtKB/Swiss-Prot;Acc:Q9UFP1]
ENSG00000214381	C3orf66	ENST00000398239	protein_coding	108897012	1,09E+08	+	1,09E+08	73	-	Putative uncharacterized protein C3orf66 [Source:UniProtKB/Swiss-Prot;Acc:Q6ZWE1]
ENSG00000123838	C4BPA	ENST00000391922	protein_coding	207277607	2,07E+08	+	2,07E+08	54	-	C4b-binding protein alpha chain Precursor (C4bp)(Proline-rich protein)(PRP) [Source:UniProtKB/Swiss-Prot;Acc:P04003]
ENSG00000123838	C4BPA	ENST00000367070	protein_coding	207277607	2,07E+08	+	2,07E+08	54	-	C4b-binding protein alpha chain Precursor (C4bp)(Proline-rich protein)(PRP) [Source:UniProtKB/Swiss-Prot;Acc:P04003]
ENSG00000186312	CA5BP	ENST00000380331	protein_coding	15694417	15694616	+	15694454	37	-	Putative carbonic anhydrase 5B-like protein (CA-VB-like protein) [Source:UniProtKB/Swiss-Prot;Acc:Q8WTZ4]
ENSG00000099991	CABIN1	ENST00000403176	protein_coding	24494047	24494246	+	24494100	53	-	Calcineurin-binding protein cabin-1 (Calcineurin inhibitor)(CAIN) [Source:UniProtKB/Swiss-Prot;Acc:Q9Y6J0]
ENSG00000165806	CASP7	ENST00000452490	protein_coding	115457184	1,15E+08	+	1,15E+08	68	-	Caspase-7 Precursor (CASP-7)(EC 3.4.22.60)(ICE-like apoptotic protease 3)(ICE-LAP3)(Apoptotic protease Mch-3)(CMH-1) [Contains Caspase-7 subunit p20;Caspase-7 subunit p11] [Source:UniProtKB/Swiss-Prot;Acc:P55210]

ENSEMBL55 GeneID	Gene Symbol	ENSEMBL55 TranscriptID	Biotype	Gene start	Gene end	Strand	Motif start	TSS offset	Motif strand	Description
ENSG00000064012	CASP8	ENST00000323492	protein_coding	202125223	2,02E+08	+	2,02E+08	64	-	Caspase-8 Precursor (CASP-8)(EC 3.4.22.61)(ICE-like apoptotic protease 5)(MORT1-associated CED-3 homolog)(MACH)(FADD-homologous ICE/CED-3-like protease)(FADD-like ICE)(FLICE)(Apoptotic cysteine protease)(Apoptotic protease Mch-5)(CAP4) [Contains Caspase-8 subunit p18;Caspase-8 subunit p10] [Source:UniProtKB/Swiss-Prot;Acc:Q14790]
ENSG00000103021	CCDC113	ENST00000443128	protein_coding	58283840	58284039	+	58283918	78	-	Coiled-coil domain-containing protein 113 [Source:UniProtKB/Swiss-Prot;Acc:Q9H0I3]
ENSG00000103021	CCDC113	ENST00000219299	protein_coding	58283840	58284039	+	58283918	78	-	Coiled-coil domain-containing protein 113 [Source:UniProtKB/Swiss-Prot;Acc:Q9H0I3]
ENSG00000139537	CCDC65	ENST00000266984	protein_coding	49297893	49298092	+	49297951	58	-	Coiled-coil domain-containing protein 65 (Testis development protein NYD-SP28) [Source:UniProtKB/Swiss-Prot;Acc:Q8IXS2]
ENSG00000139537	CCDC65	ENST00000320516	protein_coding	49297893	49298092	+	49297951	58	-	Coiled-coil domain-containing protein 65 (Testis development protein NYD-SP28) [Source:UniProtKB/Swiss-Prot;Acc:Q8IXS2]
ENSG00000151465	CDC123	ENST00000440613	protein_coding	12272947	12273146	+	12273016	69	-	Cell division cycle protein 123 homolog (Protein D123)(HT-1080)(PZ32) [Source:UniProtKB/Swiss-Prot;Acc:O75794]
ENSG00000188312	CENPP	ENST00000375576	protein_coding	95372826	95373025	+	95372889	63	-	Centromere protein P (CENP-P) [Source:UniProtKB/Swiss-Prot;Acc:Q6IPU0]
ENSG00000148019	CEP78	ENST00000424347	protein_coding	80879123	80879322	+	80879146	23	-	Centrosomal protein of 78 kDa (Cep78) [Source:UniProtKB/Swiss-Prot;Acc:Q5JTW2]
ENSG00000184984	CHRM5	ENST00000327937	protein_coding	34354843	34355042	+	34354918	75	-	Muscarinic acetylcholine receptor M5 [Source:UniProtKB/Swiss-Prot;Acc:P08912]
ENSG00000237289	CKMT1B	ENST00000411560	protein_coding	43891590	43891789	+	43891674	84	-	Creatine kinase, ubiquitous mitochondrial Precursor (EC 2.7.3.2)(U-MtCK)(Acidic-type mitochondrial creatine kinase)(Mia-CK) [Source:UniProtKB/Swiss-Prot;Acc:P12532]
ENSG00000073464	CLCN4	ENST00000380829	protein_coding	10126488	10126687	+	10126537	49	-	Chloride channel protein 4 (ClC-4) [Source:UniProtKB/Swiss-Prot;Acc:P51793]
ENSG00000148444	COMMD3	ENST00000376776	protein_coding	22605338	22605537	+	22605382	44	-	COMM domain-containing protein 3 (Protein Bup)(Protein PIL) [Source:UniProtKB/Swiss-Prot;Acc:Q9UBI1]
ENSG00000148444	COMMD3	ENST00000456711	protein_coding	22605346	22605545	+	22605382	36	-	COMM domain-containing protein 3 (Protein Bup)(Protein PIL) [Source:UniProtKB/Swiss-Prot;Acc:Q9UBI1]
ENSG00000148444	COMMD3	ENST00000444869	protein_coding	22605349	22605548	+	22605382	33	-	COMM domain-containing protein 3 (Protein Bup)(Protein PIL) [Source:UniProtKB/Swiss-Prot;Acc:Q9UBI1]
ENSG00000148444	COMMD3	ENST00000376836	protein_coding	22605299	22605498	+	22605382	83	-	COMM domain-containing protein 3 (Protein Bup)(Protein PIL) [Source:UniProtKB/Swiss-Prot;Acc:Q9UBI1]
ENSG00000148444	COMMD3	ENST00000376787	protein_coding	22605338	22605537	+	22605382	44	-	COMM domain-containing protein 3 (Protein Bup)(Protein PIL) [Source:UniProtKB/Swiss-Prot;Acc:Q9UBI1]
ENSG00000148444	COMMD3	ENST00000376786	protein_coding	22605338	22605537	+	22605382	44	-	COMM domain-containing protein 3 (Protein Bup)(Protein PIL) [Source:UniProtKB/Swiss-Prot;Acc:Q9UBI1]
ENSG00000148444	COMMD3	ENST00000421135	protein_coding	22605338	22605537	+	22605382	44	-	COMM domain-containing protein 3 (Protein Bup)(Protein PIL) [Source:UniProtKB/Swiss-Prot;Acc:Q9UBI1]
ENSG00000168090	COPS6	ENST00000303904	protein_coding	99686583	99686782	+	99686670	87	-	COP9 signalosome complex subunit 6 (Signalosome subunit 6)(SGN6)(JAB1-containing signalosome subunit 6)(Vpr-interacting protein)(hVIP)(MOV34 homolog) [Source:UniProtKB/Swiss-Prot;Acc:Q7L5N1]

ENSEMBL55 GeneID	Gene Symbol	ENSEMBL55 TranscriptID	Biotype	Gene start	Gene end	Strand	Motif start	TSS offset	Motif strand	Description
ENSG00000168090	COPS6	ENST00000418625	protein_coding	99686604	99686803	+	99686670	66	-	COP9 signalosome complex subunit 6 (Signalosome subunit 6)(SGN6)(JAB1-containing signalosome subunit 6)(Vpr-interacting protein)(hVIP)(MOV34 homolog) [Source:UniProtKB/Swiss-Prot;Acc:Q7L5N1]
ENSG00000168090	COPS6	ENST00000416531	protein_coding	99686609	99686808	+	99686670	61	-	COP9 signalosome complex subunit 6 (Signalosome subunit 6)(SGN6)(JAB1-containing signalosome subunit 6)(Vpr-interacting protein)(hVIP)(MOV34 homolog) [Source:UniProtKB/Swiss-Prot;Acc:Q7L5N1]
ENSG00000168090	COPS6	ENST00000419210	protein_coding	99686598	99686797	+	99686670	72	-	COP9 signalosome complex subunit 6 (Signalosome subunit 6)(SGN6)(JAB1-containing signalosome subunit 6)(Vpr-interacting protein)(hVIP)(MOV34 homolog) [Source:UniProtKB/Swiss-Prot;Acc:Q7L5N1]
ENSG00000132792	CTNNBL1	ENST00000373469	protein_coding	36405675	36405874	+	36405752	77	-	Beta-catenin-like protein 1 (Nuclear-associated protein)(NAP)(Testis development protein NYD-SP19) [Source:UniProtKB/Swiss-Prot;Acc:Q8WYA6]
ENSG00000205279	CTXN3	ENST00000395322	protein_coding	126988707	1,27E+08	+	1,27E+08	44	-	Cortexin-3 (Kidney and brain-expressed protein) [Source:UniProtKB/Swiss-Prot;Acc:Q4LDR2]
ENSG00000124875	CXCL6	ENST00000226317	protein_coding	74702273	74702472	+	74702354	81	-	C-X-C motif chemokine 6 Precursor (Small-inducible cytokine B6)(Granulocyte chemotactic protein 2)(GCP-2)(Chemokine alpha 3)(CKA-3) [Contains Small-inducible cytokine B6, N-processed variant 1;Small-inducible cytokine B6, N-processed variant 2;Small-inducible cytokine B6, N-processed variant 3] [Source:UniProtKB/Swiss-Prot;Acc:P80162]
ENSG00000023697	DERA	ENST00000025429	protein_coding	16109701	16109900	+	16109768	67	-	Putative deoxyribose-phosphate aldolase (EC 4.1.2.4)(Phosphodeoxyriboaldolase)(Deoxyriboaldolase)(DERA) [Source:UniProtKB/Swiss-Prot;Acc:Q9Y315]
ENSG00000184911	DMRTC1	ENST00000373533	protein_coding	72062802	72063001	+	72062884	82	-	Doublesex- and mab-3-related transcription factor C1 [Source:UniProtKB/Swiss-Prot;Acc:Q5HYR2]
ENSG00000124721	DNAH8	ENST00000359357	protein_coding	38690552	38690751	+	38690585	33	-	Dynein heavy chain 8, axonemal (Axonemal beta dynein heavy chain 8)(Ciliary dynein heavy chain 8) [Source:UniProtKB/Swiss-Prot;Acc:Q96JB1]
ENSG00000101457	DNTTIP1	ENST00000415790	protein_coding	44421175	44421374	+	44421199	24	-	Deoxynucleotidyltransferase terminal-interacting protein 1 (Terminal deoxynucleotidyltransferase-interacting factor 1)(TdT-interacting factor 1)(TdIF1) [Source:UniProtKB/Swiss-Prot;Acc:Q9H147]
ENSG00000172421	EFCAB3	ENST00000305286	protein_coding	60457914	60458113	+	60457986	72	-	EF-hand calcium-binding domain-containing protein 3 [Source:UniProtKB/Swiss-Prot;Acc:Q8N7B9]
ENSG00000084710	EFR3B	ENST00000405108	protein_coding	25338857	25339056	+	25338900	43	-	Protein EFR3 homolog B [Source:UniProtKB/Swiss-Prot;Acc:Q9Y2G0]
ENSG00000156976	EIF4A2	ENST00000443963	protein_coding	186501386	1,87E+08	+	1,87E+08	99	-	Eukaryotic initiation factor 4A-II (eIF-4A-II)(eIF4A-II)(EC 3.6.1.-)(ATP-dependent RNA helicase eIF4A-2) [Source:UniProtKB/Swiss-Prot;Acc:Q14240]
ENSG00000088926	F11	ENST00000452239	protein_coding	187197009	1,87E+08	+	1,87E+08	91	-	Coagulation factor XI Precursor (FXI)(EC 3.4.21.27)(Plasma thromboplastin antecedent)(PTA) [Contains Coagulation factor XIa heavy chain;Coagulation factor XIa light chain] [Source:UniProtKB/Swiss-Prot;Acc:P03951]

ENSEMBL55 GeneID	Gene Symbol	ENSEMBL55 TranscriptID	Biotype	Gene start	Gene end	Strand	Motif start	TSS offset	Motif strand	Description
ENSG00000156500	FAM122C	ENST00000370784	protein_coding	133941267	1,34E+08	+	1,34E+08	44	-	Protein FAM122C [Source:UniProtKB/Swiss-Prot;Acc:Q6P4D5]
ENSG00000156500	FAM122C	ENST00000445123	protein_coding	133941267	1,34E+08	+	1,34E+08	44	-	Protein FAM122C [Source:UniProtKB/Swiss-Prot;Acc:Q6P4D5]
ENSG00000156500	FAM122C	ENST00000366189	protein_coding	133941224	1,34E+08	+	1,34E+08	87	-	Protein FAM122C [Source:UniProtKB/Swiss-Prot;Acc:Q6P4D5]
ENSG00000156500	FAM122C	ENST00000458639	protein_coding	133941267	1,34E+08	+	1,34E+08	44	-	Protein FAM122C [Source:UniProtKB/Swiss-Prot;Acc:Q6P4D5]
ENSG00000156500	FAM122C	ENST00000370786	protein_coding	133941224	1,34E+08	+	1,34E+08	87	-	Protein FAM122C [Source:UniProtKB/Swiss-Prot;Acc:Q6P4D5]
ENSG00000156500	FAM122C	ENST00000370785	protein_coding	133941224	1,34E+08	+	1,34E+08	87	-	Protein FAM122C [Source:UniProtKB/Swiss-Prot;Acc:Q6P4D5]
ENSG00000203780	FANK1	ENST00000417114	protein_coding	127661942	1,28E+08	+	1,28E+08	95	-	Fibronectin type 3 and ankyrin repeat domains protein 1 [Source:UniProtKB/Swiss-Prot;Acc:Q8TC84]
ENSG00000147364	FBXO25	ENST00000382824	protein_coding	363072	363271	+	363114	42	-	F-box only protein 25 [Source:UniProtKB/Swiss-Prot;Acc:Q8TCJ0]
ENSG00000146192	FGD2	ENST00000274963	protein_coding	36973423	36973622	+	36973471	48	-	FYVE, RhoGEF and PH domain-containing protein 2 (Zinc finger FYVE domain-containing protein 4) [Source:UniProtKB/Swiss-Prot;Acc:Q7Z6J4]
ENSG00000146192	FGD2	ENST00000373535	protein_coding	36973422	36973621	+	36973471	49	-	FYVE, RhoGEF and PH domain-containing protein 2 (Zinc finger FYVE domain-containing protein 4) [Source:UniProtKB/Swiss-Prot;Acc:Q7Z6J4]
ENSG00000022267	FHL1	ENST00000370676	protein_coding	135279272	1,35E+08	+	1,35E+08	95	-	Four and a half LIM domains protein 1 (FHL-1)(Skeletal muscle LIM-protein 1)(SLIM 1)(SLIM) [Source:UniProtKB/Swiss-Prot;Acc:Q13642]
ENSG00000198225	FKBP1C	ENST00000356170	protein_coding	63921462	63921661	+	63921548	86	-	Novel protein similar to FK506 binding protein 1A, 12kDa FKBP1AHCG1998784 ; [Source:UniProtKB/TrEMBL;Acc:Q5VVH2]
ENSG00000010932	FMO1	ENST00000433267	protein_coding	171217638	1,71E+08	+	1,71E+08	49	-	Dimethylaniline monooxygenase [N-oxide-forming] 1 (EC 1.14.13.8)(Fetal hepatic flavin-containing monooxygenase 1)(FMO 1)(Dimethylaniline oxidase 1) [Source:UniProtKB/Swiss-Prot;Acc:Q01740]
ENSG00000010932	FMO1	ENST00000402921	protein_coding	171217647	1,71E+08	+	1,71E+08	40	-	Dimethylaniline monooxygenase [N-oxide-forming] 1 (EC 1.14.13.8)(Fetal hepatic flavin-containing monooxygenase 1)(FMO 1)(Dimethylaniline oxidase 1) [Source:UniProtKB/Swiss-Prot;Acc:Q01740]
ENSG00000010932	FMO1	ENST00000367750	protein_coding	171217663	1,71E+08	+	1,71E+08	24	-	Dimethylaniline monooxygenase [N-oxide-forming] 1 (EC 1.14.13.8)(Fetal hepatic flavin-containing monooxygenase 1)(FMO 1)(Dimethylaniline oxidase 1) [Source:UniProtKB/Swiss-Prot;Acc:Q01740]
ENSG00000073910	FRY	ENST00000400513	protein_coding	32635240	32635439	+	32635268	28	-	Protein furry homolog [Source:UniProtKB/Swiss-Prot;Acc:Q5TBA9]
ENSG00000144278	GALNT13	ENST00000431076	protein_coding	154996854	1,55E+08	+	1,55E+08	48	-	Polypeptide N-acetylgalactosaminyltransferase 13 (EC 2.4.1.41)(Polypeptide GalNAc transferase 13)(pp-GaNTase 13)(GalNAc-T13)(Protein-UDP acetylgalactosaminyltransferase 13)(UDP-GalNAc:polypeptide N-acetylgalactosaminyltransferase 13) [Source:UniProtKB/Swiss-Prot;Acc:Q8IUC8]
ENSG00000111846	GCNT2	ENST00000265012	protein_coding	10585993	10586192	+	10586069	76	-	N-acetyllactosaminide beta-1,6-N-acetylglucosaminyl-transferase (N-acetylglucosaminyltransferase)(EC 2.4.1.150)(I-

ENSEMBL55 GeneID	Gene Symbol	ENSEMBL55 TranscriptID	Biotype	Gene start	Gene end	Strand	Motif start	TSS offset	Motif strand	Description
										branching enzyme)(IGNT) [Source:UniProtKB/Swiss-Prot;Acc:Q06430]
ENSG00000119125	GDA	ENST00000436438	protein_coding	74842913	74843112	+	74842982	69	-	Guanine deaminase (Guanase)(Guanine aminase)(EC 3.5.4.3)(Guanine aminohydrolase)(GAH)(p51-nedasin) [Source:UniProtKB/Swiss-Prot;Acc:Q9Y2T3]
ENSG00000112414	GPR126	ENST00000367608	protein_coding	142723948	1,43E+08	+	1,43E+08	64	-	Probable G-protein coupled receptor 126 Precursor [Source:UniProtKB/Swiss-Prot;Acc:Q86SQ4]
ENSG00000164199	GPR98	ENST00000450321	protein_coding	89968371	89968570	+	89968452	81	-	G-protein coupled receptor 98 Precursor (Monogenic audiogenic seizure susceptibility protein 1 homolog)(Very large G-protein coupled receptor 1)(Usher syndrome type-2C protein) [Source:UniProtKB/Swiss-Prot;Acc:Q8WXG9]
ENSG00000075218	GTSE1	ENST00000440170	protein_coding	46693352	46693551	+	46693426	74	-	G2 and S phase-expressed protein 1 (B99 homolog) [Source:UniProtKB/Swiss-Prot;Acc:Q9NYZ3]
ENSG00000206149	HERC2P2	ENST00000382946	protein_coding	28878409	28878608	+	28878472	63	-	Putative uncharacterized protein HERC2P2 [Source:UniProtKB/TrEMBL;Acc:A6NLQ0]
ENSG00000204592	HLA-E	ENST00000376630	protein_coding	30457271	30457470	+	30457305	34	-	major histocompatibility complex, class I, E precursor [Source:RefSeq peptide;Acc:NP_005507]
ENSG00000204592	HLA-E	ENST00000447104	protein_coding	30457231	30457430	+	30457305	74	-	major histocompatibility complex, class I, E precursor [Source:RefSeq peptide;Acc:NP_005507]
ENSG00000204592	HLA-E	ENST00000427226	protein_coding	30457231	30457430	+	30457305	74	-	major histocompatibility complex, class I, E precursor [Source:RefSeq peptide;Acc:NP_005507]
ENSG00000170606	HSPA4	ENST00000304858	protein_coding	132387662	1,32E+08	+	1,32E+08	35	-	Heat shock 70 kDa protein 4 (Heat shock 70-related protein APG-2)(HSP70RY) [Source:UniProtKB/Swiss-Prot;Acc:P34932]
ENSG00000170606	HSPA4	ENST00000321956	protein_coding	132387662	1,32E+08	+	1,32E+08	35	-	Heat shock 70 kDa protein 4 (Heat shock 70-related protein APG-2)(HSP70RY) [Source:UniProtKB/Swiss-Prot;Acc:P34932]
ENSG00000159387	IRX6	ENST00000290552	protein_coding	55357672	55357871	+	55357724	52	-	Iroquois-class homeodomain protein IRX-6 (Iroquois homeobox protein 6)(Homeodomain protein IRXB3) [Source:UniProtKB/Swiss-Prot;Acc:P78412]
ENSG00000167930	ITFG3	ENST00000417499	protein_coding	290187	290386	+	290233	46	-	Protein ITFG3 [Source:UniProtKB/Swiss-Prot;Acc:Q9H0X4]
ENSG00000142945	KIF2C	ENST00000423289	protein_coding	45226152	45226351	+	45226204	52	-	Kinesin-like protein KIF2C (Mitotic centromere-associated kinesin)(MCAK)(Kinesin-like protein 6) [Source:UniProtKB/Swiss-Prot;Acc:Q99661]
ENSG00000150045	KLRF1	ENST00000279545	protein_coding	9980077	9980276	+	9980147	70	-	Killer cell lectin-like receptor subfamily F member 1 (Lectin-like receptor F1)(Activating coreceptor NKp80) [Source:UniProtKB/Swiss-Prot;Acc:Q9NZS2]
ENSG00000150045	KLRF1	ENST00000279544	protein_coding	9980077	9980276	+	9980147	70	-	Killer cell lectin-like receptor subfamily F member 1 (Lectin-like receptor F1)(Activating coreceptor NKp80) [Source:UniProtKB/Swiss-Prot;Acc:Q9NZS2]
ENSG00000150045	KLRF1	ENST00000354855	protein_coding	9980077	9980276	+	9980147	70	-	Killer cell lectin-like receptor subfamily F member 1 (Lectin-like receptor F1)(Activating coreceptor NKp80) [Source:UniProtKB/Swiss-Prot;Acc:Q9NZS2]
ENSG00000150045	KLRF1	ENST00000324214	protein_coding	9980077	9980276	+	9980147	70	-	Killer cell lectin-like receptor subfamily F member 1 (Lectin-like receptor F1)(Activating coreceptor NKp80) [Source:UniProtKB/Swiss-Prot;Acc:Q9NZS2]
ENSG00000203786	KPRP	ENST00000368773	protein_coding	152730506	1,53E+08	+	1,53E+08	54	-	Keratinocyte proline-rich protein (hKPRP) [Source:UniProtKB/Swiss-Prot;Acc:Q5T749]

ENSEMBL55 GeneID	Gene Symbol	ENSEMBL55 TranscriptID	Biotype	Gene start	Gene end	Strand	Motif start	TSS offset	Motif strand	Description
ENSG00000053747	LAMA3	ENST00000269217	protein_coding	21452984	21453183	+	21453046	62	-	Laminin subunit alpha-3 Precursor (Epiligrin 170 kDa subunit)(E170)(Nicein subunit alpha) [Source:UniProtKB/Swiss-Prot;Acc:Q16787]
ENSG00000186001	LRCH3	ENST00000452660	protein_coding	197574830	1,98E+08	+	1,98E+08	29	-	Leucine-rich repeat and calponin homology domain-containing protein 3 Precursor [Source:UniProtKB/Swiss-Prot;Acc:Q96II8]
ENSG00000171492	LRRC8D	ENST00000414841	protein_coding	90308607	90308806	+	90308679	72	-	Leucine-rich repeat-containing protein 8D [Source:UniProtKB/Swiss-Prot;Acc:Q7L1W4]
ENSG00000127603	MACF1	ENST00000422234	protein_coding	39934337	39934536	+	39934361	24	-	Microtubule-actin cross-linking factor 1, isoforms 1/2/3/5 (Actin cross-linking family protein 7)(Macrophin-1)(Trabeculin-alpha)(620 kDa actin-binding protein)(ABP620) [Source:UniProtKB/Swiss-Prot;Acc:Q9UPN3]
ENSG00000063601	MTMR1	ENST00000428594	protein_coding	149900721	1,5E+08	+	1,5E+08	67	-	Myotubularin-related protein 1 (EC 3.1.3.-) [Source:UniProtKB/Swiss-Prot;Acc:Q13613]
ENSG00000041515	MYO16	ENST00000357550	protein_coding	109318231	1,09E+08	+	1,09E+08	30	-	Myosin-XVI (Unconventional myosin-16) [Source:UniProtKB/Swiss-Prot;Acc:Q9Y6X6]
ENSG00000041515	MYO16	ENST00000251041	protein_coding	109318231	1,09E+08	+	1,09E+08	30	-	Myosin-XVI (Unconventional myosin-16) [Source:UniProtKB/Swiss-Prot;Acc:Q9Y6X6]
ENSG00000136504	MYST2	ENST00000454930	protein_coding	47866058	47866257	+	47866094	36	-	Histone acetyltransferase MYST2 (MYST protein 2)(EC 2.3.1.48)(MOZ, YBF2/SAS3, SAS2 and TIP60 protein 2)(Histone acetyltransferase binding to ORC1) [Source:UniProtKB/Swiss-Prot;Acc:O95251]
ENSG00000136504	MYST2	ENST00000259021	protein_coding	47866058	47866257	+	47866094	36	-	Histone acetyltransferase MYST2 (MYST protein 2)(EC 2.3.1.48)(MOZ, YBF2/SAS3, SAS2 and TIP60 protein 2)(Histone acetyltransferase binding to ORC1) [Source:UniProtKB/Swiss-Prot;Acc:O95251]
ENSG00000136504	MYST2	ENST00000435742	protein_coding	47866058	47866257	+	47866094	36	-	Histone acetyltransferase MYST2 (MYST protein 2)(EC 2.3.1.48)(MOZ, YBF2/SAS3, SAS2 and TIP60 protein 2)(Histone acetyltransferase binding to ORC1) [Source:UniProtKB/Swiss-Prot;Acc:O95251]
ENSG00000136504	MYST2	ENST00000424009	protein_coding	47866058	47866257	+	47866094	36	-	Histone acetyltransferase MYST2 (MYST protein 2)(EC 2.3.1.48)(MOZ, YBF2/SAS3, SAS2 and TIP60 protein 2)(Histone acetyltransferase binding to ORC1) [Source:UniProtKB/Swiss-Prot;Acc:O95251]
ENSG00000138386	NAB1	ENST00000434473	protein_coding	191524556	1,92E+08	+	1,92E+08	57	-	NGFI-A-binding protein 1 (EGR-1-binding protein 1)(Transcriptional regulatory protein p54) [Source:UniProtKB/Swiss-Prot;Acc:Q13506]
ENSG00000095380	NANS	ENST00000415280	protein_coding	100839111	1,01E+08	+	1,01E+08	78	-	Sialic acid synthase (N-acetylneuraminate synthase)(EC 2.5.1.56)(N-acetylneuraminic acid synthase)(N-acetylneuraminate-9-phosphate synthase)(EC 2.5.1.57)(N-acetylneuraminic acid phosphate synthase) [Source:UniProtKB/Swiss-Prot;Acc:Q9NR45]
ENSG00000171428	NAT1	ENST00000307719	protein_coding	18067615	18067814	+	18067636	21	-	Arylamine N-acetyltransferase 1 (EC 2.3.1.5)(Arylamide acetylase 1)(Monomorphic arylamine N-acetyltransferase)(MNAT)(N-acetyltransferase type 1)(NAT-1) [Source:UniProtKB/Swiss-Prot;Acc:P18440]
ENSG00000188505	NCCRP1	ENST00000339852	protein_coding	39687601	39687800	+	39687661	60	-	Non-specific cytotoxic cell receptor protein 1 homolog [Source:UniProtKB/Swiss-Prot;Acc:Q6ZVX7]

ENSEMBL55 GeneID	Gene Symbol	ENSEMBL55 TranscriptID	Biotype	Gene start	Gene end	Strand	Motif start	TSS offset	Motif strand	Description
ENSG00000162736	NCSTN	ENST00000392212	protein_coding	160314380	1,6E+08	+	1,6E+08	29	-	Nicastrin Precursor [Source:UniProtKB/Swiss-Prot;Acc:Q92542]
ENSG00000224435	NF1L4	ENST00000413428	protein_coding	16352989	16353188	+	16353087	98	-	Putative neurofibromin 1-like protein 4/6 Precursor [Source:UniProtKB/Swiss-Prot;Acc:P0C859]
ENSG00000224435	NF1L4	ENST00000446978	protein_coding	16352989	16353188	+	16353087	98	-	Putative neurofibromin 1-like protein 4/6 Precursor [Source:UniProtKB/Swiss-Prot;Acc:P0C859]
ENSG00000163531	NFASC	ENST00000367173	protein_coding	204919685	2,05E+08	+	2,05E+08	81	-	Neurofascin Precursor [Source:UniProtKB/Swiss-Prot;Acc:O94856]
ENSG00000162711	NLRP3	ENST00000336119	protein_coding	247581351	2,48E+08	+	2,48E+08	87	-	NACHT, LRR and PYD domains-containing protein 3 (Cold autoinflammatory syndrome 1 protein)(Cryopyrin)(PYRIN-containing APAF1-like protein 1)(Angiotensin/vasopressin receptor AII/AVP-like) [Source:UniProtKB/Swiss-Prot;Acc:Q96P20]
ENSG00000162711	NLRP3	ENST00000366496	protein_coding	247581351	2,48E+08	+	2,48E+08	87	-	NACHT, LRR and PYD domains-containing protein 3 (Cold autoinflammatory syndrome 1 protein)(Cryopyrin)(PYRIN-containing APAF1-like protein 1)(Angiotensin/vasopressin receptor AII/AVP-like) [Source:UniProtKB/Swiss-Prot;Acc:Q96P20]
ENSG00000162711	NLRP3	ENST00000348069	protein_coding	247581351	2,48E+08	+	2,48E+08	87	-	NACHT, LRR and PYD domains-containing protein 3 (Cold autoinflammatory syndrome 1 protein)(Cryopyrin)(PYRIN-containing APAF1-like protein 1)(Angiotensin/vasopressin receptor AII/AVP-like) [Source:UniProtKB/Swiss-Prot;Acc:Q96P20]
ENSG00000011052	NME1-NME2	ENST00000393183	protein_coding	49233016	49233215	+	49233045	29	-	Nucleoside diphosphate kinase A (NDP kinase A)(NDK A)(EC 2.7.4.6)(Tumor metastatic process-associated protein)(Metastasis inhibition factor nm23)(nm23-H1)(Granzyme A-activated DNase)(GAAD) [Source:UniProtKB/Swiss-Prot;Acc:P15531]
ENSG00000213240	NOTCH2NL	ENST00000454606	protein_coding	145224597	1,45E+08	+	1,45E+08	83	-	Notch homolog 2 N-terminal-like protein [Source:UniProtKB/Swiss-Prot;Acc:Q7Z3S9]
ENSG00000213240	NOTCH2NL	ENST00000369340	protein_coding	145224597	1,45E+08	+	1,45E+08	83	-	Notch homolog 2 N-terminal-like protein [Source:UniProtKB/Swiss-Prot;Acc:Q7Z3S9]
ENSG00000198805	NP	ENST00000361505	protein_coding	20937542	20937741	+	20937626	84	-	Purine nucleoside phosphorylase (PNP)(EC 2.4.2.1)(Inosine phosphorylase) [Source:UniProtKB/Swiss-Prot;Acc:P00491]
ENSG00000198805	NP	ENST00000361505	protein_coding	20937542	20937741	+	20937616	74	-	Purine nucleoside phosphorylase (PNP)(EC 2.4.2.1)(Inosine phosphorylase) [Source:UniProtKB/Swiss-Prot;Acc:P00491]
ENSG00000123358	NR4A1	ENST00000360284	protein_coding	52432494	52432693	+	52432549	55	-	Nuclear receptor subfamily 4 group A member 1 (Orphan nuclear receptor HMR)(Early response protein NAK1)(TR3 orphan receptor)(ST-59) [Source:UniProtKB/Swiss-Prot;Acc:P22736]
ENSG00000170502	NUDT9	ENST00000440591	protein_coding	88343735	88343934	+	88343833	98	-	ADP-ribose pyrophosphatase, mitochondrial Precursor (EC 3.6.1.13)(ADP-ribose diphosphatase)(Adenosine diphosphoribose pyrophosphatase)(ADPR-PPase)(ADP-ribose phosphohydrolase)(Nucleoside diphosphate-linked moiety X motif 9)(Nudix motif 9) [Source:UniProtKB/Swiss-Prot;Acc:Q9BW91]

ENSEMBL55 GeneID	Gene Symbol	ENSEMBL55 TranscriptID	Biotype	Gene start	Gene end	Strand	Motif start	TSS offset	Motif strand	Description
ENSG00000170502	NUDT9	ENST00000358033	protein_coding	88343738	88343937	+	88343833	95	-	ADP-ribose pyrophosphatase, mitochondrial Precursor (EC 3.6.1.13)(ADP-ribose diphosphatase)(Adenosine diphosphoribose pyrophosphatase)(ADPR-PPase)(ADP-ribose phosphohydrolase)(Nucleoside diphosphate-linked moiety X motif 9)(Nudix motif 9) [Source:UniProtKB/Swiss-Prot;Acc:Q9BW91]
ENSG00000170502	NUDT9	ENST00000302174	protein_coding	88343735	88343934	+	88343833	98	-	ADP-ribose pyrophosphatase, mitochondrial Precursor (EC 3.6.1.13)(ADP-ribose diphosphatase)(Adenosine diphosphoribose pyrophosphatase)(ADPR-PPase)(ADP-ribose phosphohydrolase)(Nucleoside diphosphate-linked moiety X motif 9)(Nudix motif 9) [Source:UniProtKB/Swiss-Prot;Acc:Q9BW91]
ENSG00000173679	OR1L1	ENST00000373686	protein_coding	125423845	1,25E+08	+	1,25E+08	86	-	Olfactory receptor 1L1 (Olfactory receptor OR9-27)(Olfactory receptor 9-C)(OR9-C) [Source:UniProtKB/Swiss-Prot;Acc:Q8NH94]
ENSG00000204700	OR2J2	ENST00000377167	protein_coding	29141311	29141510	+	29141408	97	-	olfactory receptor, family 2, subfamily J, member 2 [Source:RefSeq peptide;Acc:NP_112167]
ENSG00000181950	OR4A13P	ENST00000314689	protein_coding	55234532	55234731	+	55234591	59	-	Seven transmembrane helix receptor [Source:UniProtKB/TrEMBL;Acc:Q8NH17]
ENSG00000181698	OR5T1	ENST00000313033	protein_coding	56043029	56043228	+	56043050	21	-	Olfactory receptor 5T1 (Olfactory receptor OR11-179) [Source:UniProtKB/Swiss-Prot;Acc:Q8NG75]
ENSG00000112852	PCDHB2	ENST00000194155	protein_coding	140474237	1,4E+08	+	1,4E+08	67	-	Protocadherin beta-2 Precursor (PCDH-beta-2) [Source:UniProtKB/Swiss-Prot;Acc:Q9Y5E7]
ENSG00000081853	PCDHGA12	ENST00000253812	protein_coding	140723601	1,41E+08	+	1,41E+08	79	-	Protocadherin gamma-A2 Precursor (PCDH-gamma-A2) [Source:UniProtKB/Swiss-Prot;Acc:Q9Y5H1]
ENSG00000150593	PDCD4	ENST00000452838	protein_coding	112640806	1,13E+08	+	1,13E+08	31	-	Programmed cell death protein 4 (Nuclear antigen H731-like)(Neoplastic transformation inhibitor protein)(Protein 197/15a) [Source:UniProtKB/Swiss-Prot;Acc:Q53EL6]
ENSG00000184588	PDE4B	ENST00000371049	protein_coding	66378998	66379197	+	66379027	29	-	cAMP-specific 3',5'-cyclic phosphodiesterase 4B (EC 3.1.4.17)(DPDE4)(PDE32) [Source:UniProtKB/Swiss-Prot;Acc:Q07343]
ENSG00000184588	PDE4B	ENST00000423851	protein_coding	66378998	66379197	+	66379027	29	-	cAMP-specific 3',5'-cyclic phosphodiesterase 4B (EC 3.1.4.17)(DPDE4)(PDE32) [Source:UniProtKB/Swiss-Prot;Acc:Q07343]
ENSG00000112137	PHACTR1	ENST00000379350	protein_coding	12717893	12718092	+	12717949	56	-	Phosphatase and actin regulator 1 [Source:UniProtKB/Swiss-Prot;Acc:Q9C0D0]
ENSG00000131788	PIAS3	ENST00000369299	protein_coding	145575988	1,46E+08	+	1,46E+08	80	-	E3 SUMO-protein ligase PIAS3 (Protein inhibitor of activated STAT protein 3) [Source:UniProtKB/Swiss-Prot;Acc:Q9Y6X2]
ENSG00000131788	PIAS3	ENST00000369298	protein_coding	145576024	1,46E+08	+	1,46E+08	44	-	E3 SUMO-protein ligase PIAS3 (Protein inhibitor of activated STAT protein 3) [Source:UniProtKB/Swiss-Prot;Acc:Q9Y6X2]
ENSG00000131788	PIAS3	ENST00000393045	protein_coding	145575988	1,46E+08	+	1,46E+08	80	-	E3 SUMO-protein ligase PIAS3 (Protein inhibitor of activated STAT protein 3) [Source:UniProtKB/Swiss-Prot;Acc:Q9Y6X2]
ENSG00000065243	PKN2	ENST00000411919	protein_coding	89294785	89294984	+	89294849	64	-	Serine/threonine-protein kinase N2 (EC 2.7.11.13)(Protein kinase C-like 2)(Protein-kinase C-related kinase 2) [Source:UniProtKB/Swiss-Prot;Acc:Q16513]
ENSG00000125551	PLGLB2	ENST00000427160	protein_coding	88045921	88046120	+	88045981	60	-	Plasminogen-related protein B Precursor [Source:UniProtKB/Swiss-Prot;Acc:Q02325]

ENSEMBL55 GeneID	Gene Symbol	ENSEMBL55 TranscriptID	Biotype	Gene start	Gene end	Strand	Motif start	TSS offset	Motif strand	Description
ENSG00000127838	PNKD	ENST00000436005	protein_coding	219199426	2,19E+08	+	2,19E+08	60	-	Probable hydrolase PNKD (EC 3.-.-.-)(Paroxysmal nonkinesiogenic dyskinesia protein)(Myofibrillogenesis regulator 1)(MR-1)(Trans-activated by hepatitis C virus core protein 2) [Source:UniProtKB/Swiss-Prot;Acc:Q8N490]
ENSG00000115946	PNO1	ENST00000263657	protein_coding	68385005	68385204	+	68385056	51	-	RNA-binding protein PNO1 [Source:UniProtKB/Swiss-Prot;Acc:Q9NRX1]
ENSG00000170734	POLH	ENST00000453158	protein_coding	43543893	43544092	+	43543991	98	-	DNA polymerase eta (EC 2.7.7.7)(RAD30 homolog A)(Xeroderma pigmentosum variant type protein) [Source:UniProtKB/Swiss-Prot;Acc:Q9Y253]
ENSG00000186184	POLR1D	ENST00000399696	protein_coding	28196029	28196228	+	28196120	91	-	DNA-directed RNA polymerases I and III subunit RPAC2 (RNA polymerases I and III subunit AC2)(DNA-directed RNA polymerase I subunit D)(DNA-directed RNA polymerase I 16 kDa polypeptide)(RPA16)(RPC16)(hRPA19)(AC19) [Source:UniProtKB/Swiss-Prot;Acc:Q9Y2S0]
ENSG00000212993	POU5F1B	ENST00000391675	protein_coding	128427857	1,28E+08	+	1,28E+08	69	-	Putative POU domain, class 5, transcription factor 1-like protein 1 (Octamer-binding protein 3-like) [Source:UniProtKB/Swiss-Prot;Acc:Q06416]
ENSG00000116721	PRAMEF1	ENST00000400814	protein_coding	12854988	12855187	+	12855056	68	-	PRAME family member 1 [Source:UniProtKB/Swiss-Prot;Acc:O95521]
ENSG00000156858	PRR14	ENST00000287463	protein_coding	30662223	30662422	+	30662316	93	-	Proline-rich protein 14 [Source:UniProtKB/Swiss-Prot;Acc:Q9BWN1]
ENSG00000156858	PRR14	ENST00000300835	protein_coding	30662241	30662440	+	30662316	75	-	Proline-rich protein 14 [Source:UniProtKB/Swiss-Prot;Acc:Q9BWN1]
ENSG00000011454	RABGAP1	ENST00000403637	protein_coding	125748616	1,26E+08	+	1,26E+08	29	-	Rab GTPase-activating protein 1 (Rab6 GTPase-activating protein GAPCenA)(GAP and centrosome-associated protein) [Source:UniProtKB/Swiss-Prot;Acc:Q9Y3P9]
ENSG00000152689	RASGRP3	ENST00000407811	protein_coding	33738942	33739141	+	33738977	35	-	Ras guanyl-releasing protein 3 (Calcium and DAG-regulated guanine nucleotide exchange factor III)(Guanine nucleotide exchange factor for Rap1) [Source:UniProtKB/Swiss-Prot;Acc:Q8IV61]
ENSG00000100387	RBX1	ENST00000216225	protein_coding	41347385	41347584	+	41347414	29	-	RING-box protein 1 (Rbx1)(Regulator of cullins 1)(RING finger protein 75)(Protein ZYP) [Source:UniProtKB/Swiss-Prot;Acc:P62877]
ENSG00000180198	RCC1	ENST00000437681	protein_coding	28832863	28833062	+	28832925	62	-	Regulator of chromosome condensation (Chromosome condensation protein 1)(Cell cycle regulatory protein) [Source:UniProtKB/Swiss-Prot;Acc:P18754]
ENSG00000111445	RFC5	ENST00000420967	protein_coding	118454509	1,18E+08	+	1,18E+08	88	-	Replication factor C subunit 5 (Activator 1 subunit 5)(Replication factor C 36 kDa subunit)(RF-C 36 kDa subunit)(RFC36)(Activator 1 36 kDa subunit)(A1 36 kDa subunit) [Source:UniProtKB/Swiss-Prot;Acc:P40937]
ENSG00000111445	RFC5	ENST00000229043	protein_coding	118454508	1,18E+08	+	1,18E+08	89	-	Replication factor C subunit 5 (Activator 1 subunit 5)(Replication factor C 36 kDa subunit)(RF-C 36 kDa subunit)(RFC36)(Activator 1 36 kDa subunit)(A1 36 kDa subunit) [Source:UniProtKB/Swiss-Prot;Acc:P40937]
ENSG00000111445	RFC5	ENST00000458342	protein_coding	118454508	1,18E+08	+	1,18E+08	89	-	Replication factor C subunit 5 (Activator 1 subunit 5)(Replication factor C 36 kDa subunit)(RF-C 36 kDa subunit)(RFC36)(Activator 1 36 kDa subunit)(A1 36 kDa

ENSEMBL55 GeneID	Gene Symbol	ENSEMBL55 TranscriptID	Biotype	Gene start	Gene end	Strand	Motif start	TSS offset	Motif strand	Description
										subunit) [Source:UniProtKB/Swiss-Prot;Acc:P40937]
ENSG00000111445	RFC5	ENST00000392542	protein_coding	118454527	1,18E+08	+	1,18E+08	70	-	Replication factor C subunit 5 (Activator 1 subunit 5)(Replication factor C 36 kDa subunit)(RF-C 36 kDa subunit)(RFC36)(Activator 1 36 kDa subunit)(A1 36 kDa subunit) [Source:UniProtKB/Swiss-Prot;Acc:P40937]
ENSG00000111445	RFC5	ENST00000454402	protein_coding	118454508	1,18E+08	+	1,18E+08	89	-	Replication factor C subunit 5 (Activator 1 subunit 5)(Replication factor C 36 kDa subunit)(RF-C 36 kDa subunit)(RFC36)(Activator 1 36 kDa subunit)(A1 36 kDa subunit) [Source:UniProtKB/Swiss-Prot;Acc:P40937]
ENSG00000143344	RGL1	ENST00000367531	protein_coding	183605220	1,84E+08	+	1,84E+08	25	-	Ral guanine nucleotide dissociation stimulator-like 1 (RalGDS-like 1) [Source:UniProtKB/Swiss-Prot;Acc:Q9NZL6]
ENSG00000143344	RGL1	ENST00000304685	protein_coding	183605208	1,84E+08	+	1,84E+08	37	-	Ral guanine nucleotide dissociation stimulator-like 1 (RalGDS-like 1) [Source:UniProtKB/Swiss-Prot;Acc:Q9NZL6]
ENSG00000144468	RHBDD1	ENST00000392062	protein_coding	227729377	2,28E+08	+	2,28E+08	43	-	Rhomboid domain-containing protein 1 (EC 3.4.21.-) [Source:UniProtKB/Swiss-Prot;Acc:Q8TEB9]
ENSG00000204393	RP11-410N8.4	ENST00000375670	protein_coding	31175281	31175480	+	31175317	36	-	Putative uncharacterized protein MGC163334 [Source:UniProtKB/Swiss-Prot;Acc:Q5W150]
ENSG00000115268	RPS15P5	ENST00000233609	protein_coding	1438363	1438562	+	1438414	51	-	40S ribosomal protein S15 (RIG protein) [Source:UniProtKB/Swiss-Prot;Acc:P62841]
ENSG00000196933	RPS26P11	ENST00000356756	protein_coding	71264259	71264458	+	71264319	60	-	Putative 40S ribosomal protein S26-like 1 [Source:UniProtKB/Swiss-Prot;Acc:Q5JNZ5]
ENSG00000117676	RPS6KA1	ENST00000374162	protein_coding	26872343	26872542	+	26872391	48	-	Ribosomal protein S6 kinase alpha-1 (S6K-alpha 1)(EC 2.7.11.1)(90 kDa ribosomal protein S6 kinase 1)(p90-RSK 1)(pp90RSK1)(p90S6K)(Ribosomal S6 kinase 1)(RSK-1)(MAP kinase-activated protein kinase 1a)(MAPKAPK1A) [Source:UniProtKB/Swiss-Prot;Acc:Q15418]
ENSG00000117676	RPS6KA1	ENST00000374163	protein_coding	26872333	26872532	+	26872391	58	-	Ribosomal protein S6 kinase alpha-1 (S6K-alpha 1)(EC 2.7.11.1)(90 kDa ribosomal protein S6 kinase 1)(p90-RSK 1)(pp90RSK1)(p90S6K)(Ribosomal S6 kinase 1)(RSK-1)(MAP kinase-activated protein kinase 1a)(MAPKAPK1A) [Source:UniProtKB/Swiss-Prot;Acc:Q15418]
ENSG00000031698	SARS	ENST00000369923	protein_coding	109756554	1,1E+08	+	1,1E+08	60	-	Seryl-tRNA synthetase, cytoplasmic (EC 6.1.1.11)(Seryl-tRNA(Ser/Sec) synthetase)(Serine--tRNA ligase)(SerRS) [Source:UniProtKB/Swiss-Prot;Acc:P49591]
ENSG00000031698	SARS	ENST00000234677	protein_coding	109756540	1,1E+08	+	1,1E+08	74	-	Seryl-tRNA synthetase, cytoplasmic (EC 6.1.1.11)(Seryl-tRNA(Ser/Sec) synthetase)(Serine--tRNA ligase)(SerRS) [Source:UniProtKB/Swiss-Prot;Acc:P49591]
ENSG00000164265	SCGB3A2	ENST00000296694	protein_coding	147258274	1,47E+08	+	1,47E+08	36	-	Secretoglobin family 3A member 2 Precursor (Uteroglobin-related protein 1)(Pneumo secretory protein 1)(PnSP-1) [Source:UniProtKB/Swiss-Prot;Acc:Q96PL1]
ENSG00000166634	SERPINB12	ENST00000382768	protein_coding	61223393	61223592	+	61223446	53	-	Serpin B12 [Source:UniProtKB/Swiss-Prot;Acc:Q96P63]
ENSG00000166634	SERPINB12	ENST00000269491	protein_coding	61223393	61223592	+	61223446	53	-	Serpin B12 [Source:UniProtKB/Swiss-Prot;Acc:Q96P63]
ENSG00000100650	SFRS5	ENST00000344070	protein_coding	70234874	70235073	+	70234950	76	-	Splicing factor, arginine/serine-rich 5 (Pre-mRNA-splicing factor SRP40)(Delayed-early protein HRS) [Source:UniProtKB/Swiss-Prot;Acc:Q13243]

ENSEMBL55 GeneID	Gene Symbol	ENSEMBL55 TranscriptID	Biotype	Gene start	Gene end	Strand	Motif start	TSS offset	Motif strand	Description
ENSG00000136603	SKIL	ENST00000431185	protein_coding	170078120	1,7E+08	+	1,7E+08	59	-	Ski-like protein (Ski-related protein)(Ski-related oncogene) [Source:UniProtKB/Swiss-Prot;Acc:P12757]
ENSG00000139737	SLAIN1	ENST00000377236	protein_coding	78273017	78273216	+	78273048	31	-	SLAIN motif-containing protein 1 [Source:UniProtKB/Swiss-Prot;Acc:Q8ND83]
ENSG00000112473	SLC39A7	ENST00000444757	protein_coding	33168222	33168421	+	33168294	72	-	Zinc transporter SLC39A7 (Solute carrier family 39 member 7)(Histidine-rich membrane protein Ke4) [Source:UniProtKB/Swiss-Prot;Acc:Q92504]
ENSG00000164975	SNAPC3	ENST00000380799	protein_coding	15447110	15447309	+	15447159	49	-	snRNA-activating protein complex subunit 3 (SNAPc subunit 3)(Small nuclear RNA-activating complex polypeptide 3)(snRNA-activating protein complex 50 kDa subunit)(SNAPc 50 kDa subunit)(Proximal sequence element-binding transcription factor subunit beta)(PSE-binding factor subunit beta)(PTF subunit beta) [Source:UniProtKB/Swiss-Prot;Acc:Q92966]
ENSG00000170677	SOCS6	ENST00000302056	protein_coding	67991778	67991977	+	67991843	65	-	Suppressor of cytokine signaling 6 (SOCS-6)(Suppressor of cytokine signaling 4)(SOCS-4)(Cytokine-inducible SH2 protein 4)(CIS-4) [Source:UniProtKB/Swiss-Prot;Acc:O14544]
ENSG00000203923	SPANXN1	ENST00000370493	protein_coding	144329107	1,44E+08	+	1,44E+08	69	-	Sperm protein associated with the nucleus on the X chromosome N1 (SPANX-N1)(SPANX family member N1) [Source:UniProtKB/Swiss-Prot;Acc:Q5VSR9]
ENSG00000073849	ST6GAL1	ENST00000432152	protein_coding	186756467	1,87E+08	+	1,87E+08	62	-	Beta-galactoside alpha-2,6-sialyltransferase 1 (EC 2.4.99.1)(CMP-N-acetylneuraminate-beta-galactosamide-alpha-2,6-sialyltransferase 1)(Alpha 2,6-ST)(Sialyltransferase 1)(ST6Gal I)(B-cell antigen CD75) [Source:UniProtKB/Swiss-Prot;Acc:P15907]
ENSG00000101972	STAG2	ENST00000354548	protein_coding	123095589	1,23E+08	+	1,23E+08	76	-	Cohesin subunit SA-2 (Stromal antigen 2)(SCC3 homolog 2) [Source:UniProtKB/Swiss-Prot;Acc:Q8N3U4]
ENSG00000101972	STAG2	ENST00000458176	protein_coding	123095589	1,23E+08	+	1,23E+08	76	-	Cohesin subunit SA-2 (Stromal antigen 2)(SCC3 homolog 2) [Source:UniProtKB/Swiss-Prot;Acc:Q8N3U4]
ENSG00000101846	STS	ENST00000217961	protein_coding	7137472	7137671	+	7137557	85	-	Steryl-sulfatase Precursor (EC 3.1.6.2)(Steroid sulfatase)(Steryl-sulfate sulfohydrolase)(Arylsulfatase C)(ASC) [Source:UniProtKB/Swiss-Prot;Acc:P08842]
ENSG00000146383	TAAR6	ENST00000275198	protein_coding	132891461	1,33E+08	+	1,33E+08	88	-	Trace amine-associated receptor 6 (Trace amine receptor 4)(TaR-4) [Source:UniProtKB/Swiss-Prot;Acc:Q96RI8]
ENSG00000146385	TAAR8	ENST00000275200	protein_coding	132873832	1,33E+08	+	1,33E+08	45	-	Trace amine-associated receptor 8 (Trace amine receptor 5)(TaR-5)(G-protein coupled receptor 102) [Source:UniProtKB/Swiss-Prot;Acc:Q969N4]
ENSG00000121075	TBX4	ENST00000393853	protein_coding	59533807	59534006	+	59533851	44	-	T-box transcription factor TBX4 (T-box protein 4) [Source:UniProtKB/Swiss-Prot;Acc:P57082]
ENSG00000121075	TBX4	ENST00000240335	protein_coding	59533807	59534006	+	59533851	44	-	T-box transcription factor TBX4 (T-box protein 4) [Source:UniProtKB/Swiss-Prot;Acc:P57082]
ENSG00000178826	TMEM139	ENST00000409102	protein_coding	142977050	1,43E+08	+	1,43E+08	41	-	Transmembrane protein 139 Precursor [Source:UniProtKB/Swiss-Prot;Acc:Q8IV31]
ENSG00000158882	TOMM40L	ENST00000367988	protein_coding	161195833	1,61E+08	+	1,61E+08	80	-	Mitochondrial import receptor subunit TOM40B (Protein TOMM40-like) [Source:UniProtKB/Swiss-Prot;Acc:Q969M1]
ENSG00000158882	TOMM40L	ENST00000367988	protein_coding	161195833	1,61E+08	+	1,61E+08	90	-	Mitochondrial import receptor subunit TOM40B (Protein TOMM40-like) [Source:UniProtKB/Swiss-Prot;Acc:Q969M1]

ENSEMBL55 GeneID	Gene Symbol	ENSEMBL55 TranscriptID	Biotype	Gene start	Gene end	Strand	Motif start	TSS offset	Motif strand	Description
ENSG00000101255	TRIB3	ENST00000449710	protein_coding	361890	362089	+	361988	98	-	Tribbles homolog 3 (TRB-3)(Neuronal cell death-inducible putative kinase)(p65-interacting inhibitor of NF-kappa-B)(SINK) [Source:UniProtKB/Swiss-Prot;Acc:Q96RU7]
ENSG00000108448	TRIM16L	ENST00000449552	protein_coding	18602490	18602689	+	18602551	61	-	Tripartite motif-containing protein 16-like protein (Tripartite motif-containing protein 70) [Source:UniProtKB/Swiss-Prot;Acc:Q309B1]
ENSG00000175513	TSGA10IP	ENST00000312452	protein_coding	65713115	65713314	+	65713136	21	-	Testis-specific protein 10-interacting protein (Tsga10-interacting protein) [Source:UniProtKB/Swiss-Prot;Acc:Q3SY00]
ENSG00000118271	TTR	ENST00000237014	protein_coding	29171730	29171929	+	29171771	41	-	Transthyretin Precursor (Prealbumin)(TBPA)(TTR)(ATTR) [Source:UniProtKB/Swiss-Prot;Acc:P02766]
ENSG00000118271	TTR	ENST00000432547	protein_coding	29171730	29171929	+	29171771	41	-	Transthyretin Precursor (Prealbumin)(TBPA)(TTR)(ATTR) [Source:UniProtKB/Swiss-Prot;Acc:P02766]
ENSG00000130939	UBE4B	ENST00000414346	protein_coding	10131672	10131871	+	10131697	25	-	Ubiquitin conjugation factor E4 B (Ubiquitin fusion degradation protein 2)(Homozygously deleted in neuroblastoma 1) [Source:UniProtKB/Swiss-Prot;Acc:O95155]
ENSG00000186150	UBL4B	ENST00000334179	protein_coding	110655062	1,11E+08	+	1,11E+08	69	-	Ubiquitin-like protein 4B [Source:UniProtKB/Swiss-Prot;Acc:Q8N7F7]
ENSG00000169764	UGP2	ENST00000445915	protein_coding	64069526	64069725	+	64069612	86	-	UTP--glucose-1-phosphate uridylyltransferase (EC 2.7.7.9)(UDP-glucose pyrophosphorylase)(UDPGP)(UGPase) [Source:UniProtKB/Swiss-Prot;Acc:Q16851]
ENSG00000095787	WAC	ENST00000424883	protein_coding	28823189	28823388	+	28823258	69	-	WW domain-containing adapter protein with coiled-coil [Source:UniProtKB/Swiss-Prot;Acc:Q9BTA9]
ENSG00000173714	WFIKKN2	ENST00000426127	protein_coding	48912011	48912210	+	48912058	47	-	WAP, kazal, immunoglobulin, kunitz and NTR domain-containing protein 2 Precursor (WAP, follistatin, immunoglobulin, kunitz and NTR domain-containing-related protein)(WFIKKN-related protein)(Growth and differentiation factor-associated serum protein 1)(GASP-1)(hGASP-1) [Source:UniProtKB/Swiss-Prot;Acc:Q8TEU8]
ENSG00000230797	YY2	ENST00000429584	protein_coding	21874560	21874759	+	21874644	84	-	Transcription factor YY2 (Yin and yang 2)(YY-2)(Zinc finger protein 631) [Source:UniProtKB/Swiss-Prot;Acc:O15391]
ENSG00000148516	ZEB1	ENST00000437844	protein_coding	31608128	31608327	+	31608160	32	-	Zinc finger E-box-binding homeobox 1 (Transcription factor 8)(NIL-2-A zinc finger protein)(Negative regulator of IL2) [Source:UniProtKB/Swiss-Prot;Acc:P37275]
ENSG00000148516	ZEB1	ENST00000320985	protein_coding	31608101	31608300	+	31608160	59	-	Zinc finger E-box-binding homeobox 1 (Transcription factor 8)(NIL-2-A zinc finger protein)(Negative regulator of IL2) [Source:UniProtKB/Swiss-Prot;Acc:P37275]
ENSG00000158552	ZFAND2B	ENST00000436556	protein_coding	220059959	2,2E+08	+	2,2E+08	64	-	AN1-type zinc finger protein 2B [Source:UniProtKB/Swiss-Prot;Acc:Q8WV99]
ENSG00000136870	ZNF189	ENST00000339664	protein_coding	104161163	1,04E+08	+	1,04E+08	96	-	Zinc finger protein 189 [Source:UniProtKB/Swiss-Prot;Acc:O75820]
ENSG00000136870	ZNF189	ENST00000259395	protein_coding	104161163	1,04E+08	+	1,04E+08	96	-	Zinc finger protein 189 [Source:UniProtKB/Swiss-Prot;Acc:O75820]
ENSG00000136870	ZNF189	ENST00000374861	protein_coding	104161163	1,04E+08	+	1,04E+08	96	-	Zinc finger protein 189 [Source:UniProtKB/Swiss-Prot;Acc:O75820]
ENSG00000197013	ZNF429	ENST00000358491	protein_coding	21688437	21688636	+	21688523	86	-	Zinc finger protein 429 [Source:UniProtKB/Swiss-Prot;Acc:Q86V71]

ENSEMBL55 GeneID	Gene Symbol	ENSEMBL55 TranscriptID	Biotype	Gene start	Gene end	Strand	Motif start	TSS offset	Motif strand	Description
ENSG00000124444	ZNF576	ENST00000336564	protein_coding	44100757	44100956	+	44100856	99	-	Zinc finger protein 576 [Source:UniProtKB/Swiss-Prot;Acc:Q9H609]
ENSG00000196247	ZNF588	ENST00000360117	protein_coding	64126511	64126710	+	64126610	99	-	Zinc finger protein 107 (Zinc finger protein 588)(Zinc finger protein ZFD25) [Source:UniProtKB/Swiss-Prot;Acc:Q9UII5]
ENSG00000196247	ZNF588	ENST00000344930	protein_coding	64126511	64126710	+	64126610	99	-	Zinc finger protein 107 (Zinc finger protein 588)(Zinc finger protein ZFD25) [Source:UniProtKB/Swiss-Prot;Acc:Q9UII5]
ENSG00000172687	ZNF738	ENST00000311015	protein_coding	21541732	21541931	+	21541775	43	-	Protein ZNF738 [Source:UniProtKB/Swiss-Prot;Acc:Q8NE65]
ENSG00000172687	ZNF738	ENST00000380870	protein_coding	21541732	21541931	+	21541775	43	-	Protein ZNF738 [Source:UniProtKB/Swiss-Prot;Acc:Q8NE65]
ENSG00000176371	ZSCAN2	ENST00000379353	protein_coding	85147159	85147358	+	85147257	98	-	Zinc finger and SCAN domain-containing protein 2 (Zinc finger protein 29 homolog)(Zfp-29) [Source:UniProtKB/Swiss-Prot;Acc:Q7Z7L9]
ENSG00000176371	ZSCAN2	ENST00000448803	protein_coding	85147159	85147358	+	85147257	98	-	Zinc finger and SCAN domain-containing protein 2 (Zinc finger protein 29 homolog)(Zfp-29) [Source:UniProtKB/Swiss-Prot;Acc:Q7Z7L9]
ENSG00000004846	ABCB5	ENST00000406935	protein_coding	20686966	20687165	+	20686991	25	+	ATP-binding cassette sub-family B member 5 (P-glycoprotein ABCB5)(ABCB5 P-gp) [Source:UniProtKB/Swiss-Prot;Acc:Q2M3G0]
ENSG00000231256	AC003098.2	ENST00000449302	protein_coding	41857828	41858027	+	41857867	39	+	hypothetical protein LOC284067 [Source:RefSeq peptide;Acc:NP_001129955]
ENSG00000205396	AC004790.1-1	ENST00000379899	protein_coding	16126444	16126643	+	16126543	99	+	hypothetical LOC126536 (LOC126536), non-coding RNA [Source:RefSeq DNA;Acc:NR_026828]
ENSG00000229833	AC008763.9	ENST00000456958	protein_coding	7694693	7694892	+	7694787	94	+	
ENSG00000235183	AC009720.19-1	ENST00000447193	protein_coding	47872705	47872904	+	47872770	65	+	
ENSG00000235183	AC009720.19-1	ENST00000416881	protein_coding	47872705	47872904	+	47872770	65	+	
ENSG00000218819	AC010872.2	ENST00000450853	protein_coding	21360340	21360539	+	21360380	40	+	Putative uncharacterized protein ENSP00000384376 [Source:UniProtKB/TrEMBL;Acc:B5MCY1]
ENSG00000187600	AC016912.2	ENST00000416466	protein_coding	46656404	46656603	+	46656453	49	+	Transmembrane protein ENSP00000343375 [Source:UniProtKB/Swiss-Prot;Acc:A6NEH6]
ENSG00000215506	AC019099.1	ENST00000457266	protein_coding	25971523	25971722	+	25971553	30	+	
ENSG00000215506	AC019099.1	ENST00000419256	protein_coding	25971523	25971722	+	25971553	30	+	
ENSG00000179213	AC063977.6-1	ENST00000316401	protein_coding	51760964	51761163	+	51761042	78	+	Uncharacterized protein FLJ40235 [Source:UniProtKB/Swiss-Prot;Acc:Q8N7X8]
ENSG00000214487	AC078864.20	ENST00000439409	protein_coding	52473480	52473679	+	52473531	51	+	
ENSG00000230883	AC084035.24	ENST00000455379	protein_coding	127106115	1,27E+08	+	1,27E+08	77	+	
ENSG00000223762	AC093872.3-1	ENST00000358458	protein_coding	111338148	1,11E+08	+	1,11E+08	61	+	
ENSG00000229717	AC110615.3-1	ENST00000438151	protein_coding	76751904	76752103	+	76751938	34	+	Putative uncharacterized protein FP6679 [Source:UniProtKB/TrEMBL;Acc:Q71MF9]
ENSG00000236260	AC130366.6-5	ENST00000438689	protein_coding	12003178	12003377	+	12003270	92	+	
ENSG00000123983	ACSL3	ENST00000421680	protein_coding	223785672	2,24E+08	+	2,24E+08	68	+	Long-chain-fatty-acid--CoA ligase 3 (EC 6.2.1.3)(Long-chain acyl-CoA synthetase 3)(LACS 3) [Source:UniProtKB/Swiss-Prot;Acc:O95573]

ENSEMBL55 GeneID	Gene Symbol	ENSEMBL55 TranscriptID	Biotype	Gene start	Gene end	Strand	Motif start	TSS offset	Motif strand	Description
ENSG00000197381	ADARB1	ENST00000360697	protein_coding	46591557	46591756	+	46591615	58	+	Double-stranded RNA-specific editase 1 (EC 3.5.-.-)(dsRNA adenosine deaminase)(RNA-editing deaminase 1)(RNA-editing enzyme 1) [Source:UniProtKB/Swiss-Prot;Acc:P78563]
ENSG00000197381	ADARB1	ENST00000360697	protein_coding	46591557	46591756	+	46591605	48	+	Double-stranded RNA-specific editase 1 (EC 3.5.-.-)(dsRNA adenosine deaminase)(RNA-editing deaminase 1)(RNA-editing enzyme 1) [Source:UniProtKB/Swiss-Prot;Acc:P78563]
ENSG00000145192	AHSG	ENST00000411641	protein_coding	186330712	1,86E+08	+	1,86E+08	64	+	Alpha-2-HS-glycoprotein Precursor (Ba-alpha-2-glycoprotein)(Alpha-2-Z-globulin)(Fetuin-A) [Contains Alpha-2-HS-glycoprotein chain A;Alpha-2-HS-glycoprotein chain B] [Source:UniProtKB/Swiss-Prot;Acc:P02765]
ENSG00000215435	AL117372.35	ENST00000400332	protein_coding	59339247	59339446	+	59339341	94	+	Putative uncharacterized protein ENSP00000383186 Fragment [Source:UniProtKB/TrEMBL;Acc:A8MWJ3]
ENSG00000204122	AL162724.16-2	ENST00000373594	protein_coding	127032385	1,27E+08	+	1,27E+08	24	+	Putative uncharacterized protein ENSP00000362696 Fragment [Source:UniProtKB/TrEMBL;Acc:A6NEW8]
ENSG00000234278	AL353652.17-2	ENST00000445351	protein_coding	57741718	57741917	+	57741764	46	+	Proline-rich protein 20 [Source:UniProtKB/Swiss-Prot;Acc:Q8N7V5]
ENSG00000234755	AL359643.27	ENST00000450335	protein_coding	5044978	5045177	+	5045042	64	+	Putative uncharacterized protein UNQ5815/PRO19632 [Source:UniProtKB/Swiss-Prot;Acc:Q6UWF5]
ENSG00000223900	AL450342.16-2	ENST00000428327	protein_coding	51592283	51592482	+	51592373	90	+	PRO1197 [Source:UniProtKB/TrEMBL;Acc:Q9P1K1]
ENSG00000101745	ANKRD12	ENST00000359158	protein_coding	9239491	9239690	+	9239551	60	+	Ankyrin repeat domain-containing protein 12 (Ankyrin repeat-containing cofactor 2)(GAC-1 protein) [Source:UniProtKB/Swiss-Prot;Acc:Q6UB98]
ENSG00000011426	ANLN	ENST00000441696	protein_coding	36446012	36446211	+	36446034	22	+	Actin-binding protein anillin [Source:UniProtKB/Swiss-Prot;Acc:Q9NQW6]
ENSG00000011426	ANLN	ENST00000429082	protein_coding	36446011	36446210	+	36446034	23	+	Actin-binding protein anillin [Source:UniProtKB/Swiss-Prot;Acc:Q9NQW6]
ENSG00000135046	ANXA1	ENST00000257497	protein_coding	75766675	75766874	+	75766746	71	+	Annexin A1 (Annexin-1)(Annexin I)(Lipocortin I)(Calpactin II)(Chromobindin-9)(p35)(Phospholipase A2 inhibitory protein) [Source:UniProtKB/Swiss-Prot;Acc:P04083]
ENSG00000220703	AP000907.6-1	ENST00000403628	protein_coding	111779540	1,12E+08	+	1,12E+08	95	+	Putative uncharacterized protein ENSP00000384476 [Source:UniProtKB/TrEMBL;Acc:B5MCL6]
ENSG00000167279	AP001267.4-2	ENST00000300681	protein_coding	118308183	1,18E+08	+	1,18E+08	95	+	Putative uncharacterized protein ENSP00000300681 [Source:UniProtKB/TrEMBL;Acc:B7WPF1]
ENSG00000214264	AP002884.5-1	ENST00000397969	protein_coding	112052523	1,12E+08	+	1,12E+08	67	+	Putative uncharacterized protein ENSP00000381059 [Source:UniProtKB/TrEMBL;Acc:A8MZH4]
ENSG00000086159	AQP6	ENST00000394984	protein_coding	50366620	50366819	+	50366664	44	+	Aquaporin-6 (AQP-6)(Aquaporin-2-like)(Kidney-specific aquaporin)(hKID) [Source:UniProtKB/Swiss-Prot;Acc:Q13520]
ENSG00000086159	AQP6	ENST00000315520	protein_coding	50366620	50366819	+	50366664	44	+	Aquaporin-6 (AQP-6)(Aquaporin-2-like)(Kidney-specific aquaporin)(hKID) [Source:UniProtKB/Swiss-Prot;Acc:Q13520]
ENSG00000088756	ARHGAP28	ENST00000314319	protein_coding	6837192	6837391	+	6837236	44	+	Rho GTPase-activating protein 28 (Rho-type GTPase-activating protein 28) [Source:UniProtKB/Swiss-Prot;Acc:Q9P2N2]
ENSG00000088756	ARHGAP28	ENST00000418986	protein_coding	6837192	6837391	+	6837236	44	+	Rho GTPase-activating protein 28 (Rho-type GTPase-activating protein 28) [Source:UniProtKB/Swiss-Prot;Acc:Q9P2N2]
ENSG00000172995	ARPP-21	ENST00000421492	protein_coding	35681747	35681946	+	35681783	36	+	cAMP-regulated phosphoprotein 21 (ARPP-21)(Thymocyte cAMP-regulated phosphoprotein) [Source:UniProtKB/Swiss-Prot;Acc:Q9UBL0]

ENSEMBL55 GeneID	Gene Symbol	ENSEMBL55 TranscriptID	Biotype	Gene start	Gene end	Strand	Motif start	TSS offset	Motif strand	Description
ENSG00000111875	ASF1A	ENST00000368478	protein_coding	119150209	1,19E+08	+	1,19E+08	73	+	Histone chaperone ASF1A (Anti-silencing function protein 1 homolog A)(hAsf1)(hAsf1a)(CCG1-interacting factor A)(CIA)(hCIA) [Source:UniProtKB/Swiss-Prot;Acc:Q9Y294]
ENSG00000018625	ATP1A2	ENST00000435866	protein_coding	160098186	1,6E+08	+	1,6E+08	88	+	Sodium/potassium-transporting ATPase subunit alpha-2 Precursor (Sodium pump subunit alpha-2)(EC 3.6.3.9)(Na(+)/K(+) ATPase alpha-2 subunit) [Source:UniProtKB/Swiss-Prot;Acc:P50993]
ENSG00000167283	ATP5L	ENST00000300688	protein_coding	118272104	1,18E+08	+	1,18E+08	70	+	ATP synthase subunit g, mitochondrial (ATPase subunit g) [Source:UniProtKB/Swiss-Prot;Acc:O75964]
ENSG00000204469	BAT2	ENST00000376007	protein_coding	31588450	31588649	+	31588549	99	+	HLA-B associated transcript-2 [Source:RefSeq peptide;Acc:NP_542417]
ENSG00000204469	BAT2	ENST00000376033	protein_coding	31588502	31588701	+	31588549	47	+	HLA-B associated transcript-2 [Source:RefSeq peptide;Acc:NP_542417]
ENSG00000204469	BAT2	ENST00000414096	protein_coding	31588450	31588649	+	31588549	99	+	HLA-B associated transcript-2 [Source:RefSeq peptide;Acc:NP_542417]
ENSG00000130723	BAT2L	ENST00000405995	protein_coding	134269480	1,34E+08	+	1,34E+08	86	+	Protein BAT2-like (HLA-B-associated transcript 2-like) [Source:UniProtKB/Swiss-Prot;Acc:Q5JSZ5]
ENSG00000101425	BPI	ENST00000422597	protein_coding	36888577	36888776	+	36888664	87	+	Bactericidal permeability-increasing protein Precursor (BPI)(CAP 57) [Source:UniProtKB/Swiss-Prot;Acc:P17213]
ENSG00000101425	BPI	ENST00000451435	protein_coding	36888577	36888776	+	36888664	87	+	Bactericidal permeability-increasing protein Precursor (BPI)(CAP 57) [Source:UniProtKB/Swiss-Prot;Acc:P17213]
ENSG00000136261	BZW2	ENST00000446596	protein_coding	16700848	16701047	+	16700947	99	+	Basic leucine zipper and W2 domain-containing protein 2 [Source:UniProtKB/Swiss-Prot;Acc:Q9Y6E2]
ENSG00000136261	BZW2	ENST00000438834	protein_coding	16700897	16701096	+	16700947	50	+	Basic leucine zipper and W2 domain-containing protein 2 [Source:UniProtKB/Swiss-Prot;Acc:Q9Y6E2]
ENSG00000108021	C10orf18	ENST00000328090	protein_coding	5759622	5759821	+	5759670	48	+	Uncharacterized protein C10orf18 [Source:UniProtKB/Swiss-Prot;Acc:Q5VWN6]
ENSG00000150076	C10orf68	ENST00000375030	protein_coding	32856764	32856963	+	32856802	38	+	Uncharacterized protein C10orf68 [Source:UniProtKB/Swiss-Prot;Acc:Q9H943]
ENSG00000150076	C10orf68	ENST00000375028	protein_coding	32856764	32856963	+	32856802	38	+	Uncharacterized protein C10orf68 [Source:UniProtKB/Swiss-Prot;Acc:Q9H943]
ENSG00000150076	C10orf68	ENST00000426285	protein_coding	32856764	32856963	+	32856802	38	+	Uncharacterized protein C10orf68 [Source:UniProtKB/Swiss-Prot;Acc:Q9H943]
ENSG00000100744	C14orf129	ENST00000438650	protein_coding	96846022	96846221	+	96846107	85	+	GSK3-beta interaction protein (GSKIP) [Source:UniProtKB/Swiss-Prot;Acc:Q9P0R6]
ENSG00000159189	C1QC	ENST00000374640	protein_coding	22970118	22970317	+	22970163	45	+	Complement C1q subcomponent subunit C Precursor [Source:UniProtKB/Swiss-Prot;Acc:P02747]
ENSG00000159189	C1QC	ENST00000374639	protein_coding	22970118	22970317	+	22970163	45	+	Complement C1q subcomponent subunit C Precursor [Source:UniProtKB/Swiss-Prot;Acc:P02747]
ENSG00000160679	C1orf77	ENST00000368687	protein_coding	153610560	1,54E+08	+	1,54E+08	73	+	Uncharacterized protein C1orf77 [Source:UniProtKB/Swiss-Prot;Acc:Q9Y3Y2]
ENSG00000187833	C2orf78	ENST00000342345	protein_coding	74035932	74036131	+	74035961	29	+	Uncharacterized protein C2orf78 [Source:UniProtKB/Swiss-Prot;Acc:A6NCI8]
ENSG00000144649	C3orf41	ENST00000434206	protein_coding	43020805	43021004	+	43020881	76	+	Uncharacterized protein C3orf41 Precursor [Source:UniProtKB/Swiss-Prot;Acc:Q9UFP1]
ENSG00000144649	C3orf41	ENST00000434206	protein_coding	43020805	43021004	+	43020871	66	+	Uncharacterized protein C3orf41 Precursor [Source:UniProtKB/Swiss-Prot;Acc:Q9UFP1]

ENSEMBL55 GeneID	Gene Symbol	ENSEMBL55 TranscriptID	Biotype	Gene start	Gene end	Strand	Motif start	TSS offset	Motif strand	Description
ENSG00000148120	C9orf3	ENST00000433691	protein_coding	97766506	97766705	+	97766541	35	+	Aminopeptidase O (AP-O)(EC 3.4.11.-) [Source:UniProtKB/Swiss-Prot;Acc:Q8N6M6]
ENSG00000148120	C9orf3	ENST00000425634	protein_coding	97766506	97766705	+	97766541	35	+	Aminopeptidase O (AP-O)(EC 3.4.11.-) [Source:UniProtKB/Swiss-Prot;Acc:Q8N6M6]
ENSG00000150753	CCT5	ENST00000423695	protein_coding	10250416	10250615	+	10250504	88	+	T-complex protein 1 subunit epsilon (TCP-1-epsilon)(CCT-epsilon) [Source:UniProtKB/Swiss-Prot;Acc:P48643]
ENSG00000150753	CCT5	ENST00000440011	protein_coding	10250416	10250615	+	10250504	88	+	T-complex protein 1 subunit epsilon (TCP-1-epsilon)(CCT-epsilon) [Source:UniProtKB/Swiss-Prot;Acc:P48643]
ENSG00000135624	CCT7	ENST00000409924	protein_coding	73461437	73461636	+	73461531	94	+	T-complex protein 1 subunit eta (TCP-1-eta)(CCT-eta)(HIV-1 Nef-interacting protein) [Source:UniProtKB/Swiss-Prot;Acc:Q99832]
ENSG00000135624	CCT7	ENST00000409081	protein_coding	73461448	73461647	+	73461531	83	+	T-complex protein 1 subunit eta (TCP-1-eta)(CCT-eta)(HIV-1 Nef-interacting protein) [Source:UniProtKB/Swiss-Prot;Acc:Q99832]
ENSG00000135624	CCT7	ENST00000409081	protein_coding	73461448	73461647	+	73461541	93	+	T-complex protein 1 subunit eta (TCP-1-eta)(CCT-eta)(HIV-1 Nef-interacting protein) [Source:UniProtKB/Swiss-Prot;Acc:Q99832]
ENSG00000135624	CCT7	ENST00000399032	protein_coding	73461436	73461635	+	73461531	95	+	T-complex protein 1 subunit eta (TCP-1-eta)(CCT-eta)(HIV-1 Nef-interacting protein) [Source:UniProtKB/Swiss-Prot;Acc:Q99832]
ENSG00000158985	CDC42SE2	ENST00000446153	protein_coding	130651668	1,31E+08	+	1,31E+08	89	+	CDC42 small effector protein 2 (Small effector of CDC42 protein 2) [Source:UniProtKB/Swiss-Prot;Acc:Q9NRR3]
ENSG00000135837	CEP350	ENST00000437245	protein_coding	180049652	1,8E+08	+	1,8E+08	84	+	Centrosome-associated protein 350 (Cep350)(Centrosome-associated protein of 350 kDa) [Source:UniProtKB/Swiss-Prot;Acc:Q5VT06]
ENSG00000135837	CEP350	ENST00000367606	protein_coding	179965757	1,8E+08	+	1,8E+08	90	+	Centrosome-associated protein 350 (Cep350)(Centrosome-associated protein of 350 kDa) [Source:UniProtKB/Swiss-Prot;Acc:Q5VT06]
ENSG00000134216	CHIA	ENST00000451398	protein_coding	111857114	1,12E+08	+	1,12E+08	22	+	Acidic mammalian chitinase Precursor (AMCase)(EC 3.2.1.14)(TSA1902) [Source:UniProtKB/Swiss-Prot;Acc:Q9BZP6]
ENSG00000179583	CIITA	ENST00000381835	protein_coding	10971055	10971254	+	10971102	47	+	MHC class II transactivator (CIITA) [Source:UniProtKB/Swiss-Prot;Acc:P33076]
ENSG00000179583	CIITA	ENST00000388910	protein_coding	10971055	10971254	+	10971102	47	+	MHC class II transactivator (CIITA) [Source:UniProtKB/Swiss-Prot;Acc:P33076]
ENSG00000179583	CIITA	ENST00000324288	protein_coding	10971055	10971254	+	10971102	47	+	MHC class II transactivator (CIITA) [Source:UniProtKB/Swiss-Prot;Acc:P33076]
ENSG00000223572	CKMT1A	ENST00000415044	protein_coding	43986069	43986268	+	43986166	97	+	Creatine kinase, ubiquitous mitochondrial Precursor (EC 2.7.3.2)(U-MtCK)(Acidic-type mitochondrial creatine kinase)(Mia-CK) [Source:UniProtKB/Swiss-Prot;Acc:P12532]
ENSG00000157224	CLDN12	ENST00000394605	protein_coding	90032863	90033062	+	90032938	75	+	Claudin-12 [Source:UniProtKB/Swiss-Prot;Acc:P56749]
ENSG00000157224	CLDN12	ENST00000427904	protein_coding	90032881	90033080	+	90032938	57	+	Claudin-12 [Source:UniProtKB/Swiss-Prot;Acc:P56749]
ENSG00000007545	CRAMP1L	ENST00000454337	protein_coding	1682260	1682459	+	1682351	91	+	Protein cramped-like (Hematological and neurological expressed 1-like protein) [Source:UniProtKB/Swiss-Prot;Acc:Q96RY5]
ENSG00000131068	DEFB118	ENST00000253381	protein_coding	29956421	29956620	+	29956505	84	+	Beta-defensin 118 Precursor (Defensin, beta 118)(Beta-

ENSEMBL55 GeneID	Gene Symbol	ENSEMBL55 TranscriptID	Biotype	Gene start	Gene end	Strand	Motif start	TSS offset	Motif strand	Description
										defensin 18)(DEFB-18)(Epididymal secretory protein 13.6)(ESP13.6) [Source:UniProtKB/Swiss-Prot;Acc:Q96PH6]
ENSG00000108406	DHX40	ENST00000251241	protein_coding	57642886	57643085	+	57642985	99	+	Probable ATP-dependent RNA helicase DHX40 (EC 3.6.1.-)(DEAH box protein 40)(Protein PAD) [Source:UniProtKB/Swiss-Prot;Acc:Q8IX18]
ENSG00000185842	DNAH14	ENST00000432355	protein_coding	225339707	2,25E+08	+	2,25E+08	52	+	Dynein heavy chain 14, axonemal (Axonemal beta dynein heavy chain 14)(Ciliary dynein heavy chain 14) [Source:UniProtKB/Swiss-Prot;Acc:Q0VDD8]
ENSG00000091073	DTX2	ENST00000423250	protein_coding	76099654	76099853	+	76099740	86	+	Protein deltex-2 (Deltex2)(hDTX2)(RING finger protein 58) [Source:UniProtKB/Swiss-Prot;Acc:Q86UW9]
ENSG00000091073	DTX2	ENST00000435861	protein_coding	76107444	76107643	+	76107475	31	+	Protein deltex-2 (Deltex2)(hDTX2)(RING finger protein 58) [Source:UniProtKB/Swiss-Prot;Acc:Q86UW9]
ENSG00000203666	EFCAB2	ENST00000366521	protein_coding	245133658	2,45E+08	+	2,45E+08	24	+	EF-hand calcium-binding domain-containing protein 2 [Source:UniProtKB/Swiss-Prot;Acc:Q5VUJ9]
ENSG00000203666	EFCAB2	ENST00000447569	protein_coding	245133631	2,45E+08	+	2,45E+08	51	+	EF-hand calcium-binding domain-containing protein 2 [Source:UniProtKB/Swiss-Prot;Acc:Q5VUJ9]
ENSG00000114867	EIF4G1	ENST00000427141	protein_coding	184032462	1,84E+08	+	1,84E+08	53	+	Eukaryotic translation initiation factor 4 gamma 1 (eIF-4-gamma 1)(eIF-4G 1)(eIF-4G1)(p220) [Source:UniProtKB/Swiss-Prot;Acc:Q04637]
ENSG00000162374	ELAVL4	ENST00000396173	protein_coding	50610460	50610659	+	50610518	58	+	ELAV-like protein 4 (Paraneoplastic encephalomyelitis antigen HuD)(Hu-antigen D) [Source:UniProtKB/Swiss-Prot;Acc:P26378]
ENSG00000162374	ELAVL4	ENST00000396174	protein_coding	50610423	50610622	+	50610518	95	+	ELAV-like protein 4 (Paraneoplastic encephalomyelitis antigen HuD)(Hu-antigen D) [Source:UniProtKB/Swiss-Prot;Acc:P26378]
ENSG00000214595	EML6	ENST00000356458	protein_coding	54952149	54952348	+	54952196	47	+	Echinoderm microtubule-associated protein-like 6 (Echinoderm microtubule-associated protein-like 5-like) [Source:UniProtKB/Swiss-Prot;Acc:Q6ZMW3]
ENSG00000214595	EML6	ENST00000398629	protein_coding	54952149	54952348	+	54952196	47	+	Echinoderm microtubule-associated protein-like 6 (Echinoderm microtubule-associated protein-like 5-like) [Source:UniProtKB/Swiss-Prot;Acc:Q6ZMW3]
ENSG00000088367	EPB41L1	ENST00000454226	protein_coding	34785802	34786001	+	34785871	69	+	Band 4.1-like protein 1 (Neuronal protein 4.1)(4.1N) [Source:UniProtKB/Swiss-Prot;Acc:Q9H4G0]
ENSG00000138190	EXOC6	ENST00000458552	protein_coding	94757231	94757430	+	94757271	40	+	Exocyst complex component 6 (Exocyst complex component Sec15A)(SEC15-like protein 1) [Source:UniProtKB/Swiss-Prot;Acc:Q8TAG9]
ENSG00000203780	FANK1	ENST00000445510	protein_coding	127662068	1,28E+08	+	1,28E+08	34	+	Fibronectin type 3 and ankyrin repeat domains protein 1 [Source:UniProtKB/Swiss-Prot;Acc:Q8TC84]
ENSG00000073598	FNDC8	ENST00000158009	protein_coding	33448631	33448830	+	33448708	77	+	Fibronectin type III domain-containing protein 8 [Source:UniProtKB/Swiss-Prot;Acc:Q8TC99]
ENSG00000073910	FRY	ENST00000400513	protein_coding	32635240	32635439	+	32635269	29	+	Protein furry homolog [Source:UniProtKB/Swiss-Prot;Acc:Q5TBA9]
ENSG00000187210	GCNT1	ENST00000444201	protein_coding	79115549	79115748	+	79115633	84	+	Beta-1,3-galactosyl-O-glycosyl-glycoprotein beta-1,6-N-acetylglucosaminyltransferase (EC 2.4.1.102)(Core 2-branching enzyme)(Core2-GlcNAc-transferase)(Core 2 GNT)(C2GNT) [Source:UniProtKB/Swiss-Prot;Acc:Q02742]

ENSEMBL55 GeneID	Gene Symbol	ENSEMBL55 TranscriptID	Biotype	Gene start	Gene end	Strand	Motif start	TSS offset	Motif strand	Description
ENSG00000115419	GLS	ENST00000457316	protein_coding	191791742	1,92E+08	+	1,92E+08	96	+	Glutaminase kidney isoform, mitochondrial Precursor (GLS)(EC 3.5.1.2)(L-glutamine amidohydrolase)(K-glutaminase) [Source:UniProtKB/Swiss-Prot;Acc:O94925]
ENSG00000205336	GPR56	ENST00000379694	protein_coding	57662419	57662618	+	57662441	22	+	G-protein coupled receptor 56 Precursor (Protein TM7XN1) [Source:UniProtKB/Swiss-Prot;Acc:Q9Y653]
ENSG00000205336	GPR56	ENST00000456916	protein_coding	57662419	57662618	+	57662441	22	+	G-protein coupled receptor 56 Precursor (Protein TM7XN1) [Source:UniProtKB/Swiss-Prot;Acc:Q9Y653]
ENSG00000205336	GPR56	ENST00000388812	protein_coding	57662419	57662618	+	57662441	22	+	G-protein coupled receptor 56 Precursor (Protein TM7XN1) [Source:UniProtKB/Swiss-Prot;Acc:Q9Y653]
ENSG00000214511	HIGD1C	ENST00000398455	protein_coding	51347782	51347981	+	51347829	47	+	HIG1 domain family member 1C [Source:UniProtKB/Swiss-Prot;Acc:A8MV81]
ENSG00000156510	HKDC1	ENST00000395087	protein_coding	70980059	70980258	+	70980154	95	+	Putative hexokinase HKDC1 (EC 2.7.1.1)(Hexokinase domain-containing protein 1) [Source:UniProtKB/Swiss-Prot;Acc:Q2TB90]
ENSG00000156510	HKDC1	ENST00000354624	protein_coding	70980059	70980258	+	70980154	95	+	Putative hexokinase HKDC1 (EC 2.7.1.1)(Hexokinase domain-containing protein 1) [Source:UniProtKB/Swiss-Prot;Acc:Q2TB90]
ENSG00000156510	HKDC1	ENST00000395086	protein_coding	70980059	70980258	+	70980154	95	+	Putative hexokinase HKDC1 (EC 2.7.1.1)(Hexokinase domain-containing protein 1) [Source:UniProtKB/Swiss-Prot;Acc:Q2TB90]
ENSG00000113716	HMGXB3	ENST00000421549	protein_coding	149379609	1,49E+08	+	1,49E+08	91	+	HMG domain-containing protein 3 (HMG box-containing protein 3)(Protein SMF) [Source:UniProtKB/Swiss-Prot;Acc:Q12766]
ENSG00000100281	HMGXB4	ENST00000444518	protein_coding	35658380	35658579	+	35658453	73	+	HMG domain-containing protein 4 (HMG box-containing protein 4)(High mobility group protein 2-like 1)(Protein HMGBCG) [Source:UniProtKB/Swiss-Prot;Acc:Q9UGU5]
ENSG00000172789	HOXC5	ENST00000312492	protein_coding	54426832	54427031	+	54426857	25	+	Homeobox protein Hox-C5 (Hox-3D)(CP11) [Source:UniProtKB/Swiss-Prot;Acc:Q00444]
ENSG00000183199	HSP90AB3P	ENST00000327934	protein_coding	88812995	88813194	+	88813069	74	+	Putative heat shock protein HSP 90-beta-3 (Heat shock protein 90-beta c)(Heat shock protein 90Bc) [Source:UniProtKB/Swiss-Prot;Acc:Q58FF7]
ENSG00000170606	HSPA4	ENST00000304858	protein_coding	132387662	1,32E+08	+	1,32E+08	36	+	Heat shock 70 kDa protein 4 (Heat shock 70-related protein APG-2)(HSP70RY) [Source:UniProtKB/Swiss-Prot;Acc:P34932]
ENSG00000170606	HSPA4	ENST00000321956	protein_coding	132387662	1,32E+08	+	1,32E+08	36	+	Heat shock 70 kDa protein 4 (Heat shock 70-related protein APG-2)(HSP70RY) [Source:UniProtKB/Swiss-Prot;Acc:P34932]
ENSG00000204010	IFIT1L	ENST00000371809	protein_coding	91137813	91138012	+	91137869	56	+	Interferon-induced protein with tetratricopeptide repeats 1-like protein [Source:UniProtKB/Swiss-Prot;Acc:Q5T764]
ENSG00000006652	IFRD1	ENST00000456910	protein_coding	112090515	1,12E+08	+	1,12E+08	31	+	Interferon-related developmental regulator 1 (Nerve growth factor-inducible protein PC4) [Source:UniProtKB/Swiss-Prot;Acc:O00458]
ENSG00000006652	IFRD1	ENST00000403825	protein_coding	112090515	1,12E+08	+	1,12E+08	31	+	Interferon-related developmental regulator 1 (Nerve growth factor-inducible protein PC4) [Source:UniProtKB/Swiss-Prot;Acc:O00458]
ENSG00000006652	IFRD1	ENST00000429071	protein_coding	112090513	1,12E+08	+	1,12E+08	33	+	Interferon-related developmental regulator 1 (Nerve growth factor-inducible protein PC4) [Source:UniProtKB/Swiss-Prot;Acc:O00458]
ENSG00000231151	IGLV4-60	ENST00000435694	protein_coding	22516776	22516975	+	22516872	96	+	V5-4 protein Fragment [Source:UniProtKB/TrEMBL;Acc:Q5NV79]

ENSEMBL55 GeneID	Gene Symbol	ENSEMBL55 TranscriptID	Biotype	Gene start	Gene end	Strand	Motif start	TSS offset	Motif strand	Description
ENSG00000232262	IGLV4-69	ENST00000438781	protein_coding	22385572	22385771	+	22385668	96	+	V5-6 protein Fragment [Source:UniProtKB/TrEMBL;Acc:Q5NV92]
ENSG00000103599	IQCH	ENST00000358767	protein_coding	67547194	67547393	+	67547270	76	+	IQ domain-containing protein H (Testis development protein NYD-SP5) [Source:UniProtKB/Swiss-Prot;Acc:Q86VS3]
ENSG00000055957	ITIH1	ENST00000273283	protein_coding	52811608	52811807	+	52811682	74	+	Inter-alpha-trypsin inhibitor heavy chain H1 Precursor (Inter-alpha-inhibitor heavy chain 1)(ITI heavy chain H1)(Inter-alpha-trypsin inhibitor complex component III)(Serum-derived hyaluronan-associated protein)(SHAP) [Source:UniProtKB/Swiss-Prot;Acc:P19827]
ENSG00000214655	KIAA0913	ENST00000433366	protein_coding	75549943	75550142	+	75549972	29	+	Zinc finger SWIM domain-containing protein KIAA0913 [Source:UniProtKB/Swiss-Prot;Acc:A7E2V4]
ENSG00000198945	L3MBTL3	ENST00000361794	protein_coding	130339734	1,3E+08	+	1,3E+08	90	+	Lethal(3)malignant brain tumor-like 3 protein (L(3)mbt-like 3 protein)(H-l(3)mbt-like protein) [Source:UniProtKB/Swiss-Prot;Acc:Q96JM7]
ENSG00000198945	L3MBTL3	ENST00000368139	protein_coding	130339734	1,3E+08	+	1,3E+08	90	+	Lethal(3)malignant brain tumor-like 3 protein (L(3)mbt-like 3 protein)(H-l(3)mbt-like protein) [Source:UniProtKB/Swiss-Prot;Acc:Q96JM7]
ENSG00000147036	LANCL3	ENST00000378621	protein_coding	37430822	37431021	+	37430892	70	+	LanC-like protein 3 [Source:UniProtKB/Swiss-Prot;Acc:Q6ZV70]
ENSG00000147036	LANCL3	ENST00000378619	protein_coding	37430822	37431021	+	37430892	70	+	LanC-like protein 3 [Source:UniProtKB/Swiss-Prot;Acc:Q6ZV70]
ENSG00000187170	LCE4A	ENST00000335535	protein_coding	152681523	1,53E+08	+	1,53E+08	80	+	Late cornified envelope protein 4A (Late envelope protein 8)(Small proline-rich-like epidermal differentiation complex protein 4A) [Source:UniProtKB/Swiss-Prot;Acc:Q5TA78]
ENSG00000131981	LGALS3	ENST00000399106	protein_coding	55603699	55603898	+	55603779	80	+	Galectin-3 (Galactose-specific lectin 3)(Mac-2 antigen)(IgE-binding protein)(35 kDa lectin)(Carbohydrate-binding protein 35)(CBP 35)(Laminin-binding protein)(Lectin L-29)(L-31)(Galactoside-binding protein)(GALBP) [Source:UniProtKB/Swiss-Prot;Acc:P17931]
ENSG00000034152	MAP2K3	ENST00000316920	protein_coding	21201660	21201859	+	21201746	86	+	Dual specificity mitogen-activated protein kinase kinase 3 (MAP kinase kinase 3)(MAPKK 3)(EC 2.7.12.2)(MAPK/ERK kinase 3) [Source:UniProtKB/Swiss-Prot;Acc:P46734]
ENSG00000105976	MET	ENST00000422097	protein_coding	116398571	1,16E+08	+	1,16E+08	82	+	Hepatocyte growth factor receptor Precursor (HGF receptor)(EC 2.7.10.1)(Scatter factor receptor)(SF receptor)(HGF/SF receptor)(Met proto-oncogene tyrosine kinase)(c-Met) [Source:UniProtKB/Swiss-Prot;Acc:P08581]
ENSG00000076242	MLH1	ENST00000458009	protein_coding	37053573	37053772	+	37053632	59	+	DNA mismatch repair protein Mlh1 (MutL protein homolog 1) [Source:UniProtKB/Swiss-Prot;Acc:P40692]
ENSG00000076242	MLH1	ENST00000447829	protein_coding	37053533	37053732	+	37053632	99	+	DNA mismatch repair protein Mlh1 (MutL protein homolog 1) [Source:UniProtKB/Swiss-Prot;Acc:P40692]
ENSG00000188010	MORN2	ENST00000409077	protein_coding	39107733	39107932	+	39107760	27	+	MORN repeat-containing protein 2 (MORN motif protein in testis) [Source:UniProtKB/Swiss-Prot;Acc:Q502X0]
ENSG00000196199	MPHOSPH8	ENST00000449056	protein_coding	20242531	20242730	+	20242593	62	+	M-phase phosphoprotein 8 (Two hybrid-associated protein 3 with RanBPM)(Twa3) [Source:UniProtKB/Swiss-Prot;Acc:Q99549]
ENSG00000205364	MT1M	ENST00000379818	protein_coding	56666534	56666733	+	56666609	75	+	Metallothionein-1M (MT-1M)(Metallothionein-IM)(MT-IM) [Source:UniProtKB/Swiss-Prot;Acc:Q8N339]

ENSEMBL55 GeneID	Gene Symbol	ENSEMBL55 TranscriptID	Biotype	Gene start	Gene end	Strand	Motif start	TSS offset	Motif strand	Description
ENSG00000084676	NCOA1	ENST00000407230	protein_coding	24787179	24787378	+	24787223	44	+	Nuclear receptor coactivator 1 (NCoA-1)(EC 2.3.1.48)(Steroid receptor coactivator 1)(SRC-1)(RIP160)(Protein Hin-2)(Renal carcinoma antigen NY-REN-52) [Source:UniProtKB/Swiss-Prot;Acc:Q15788]
ENSG00000084676	NCOA1	ENST00000407230	protein_coding	24787179	24787378	+	24787213	34	+	Nuclear receptor coactivator 1 (NCoA-1)(EC 2.3.1.48)(Steroid receptor coactivator 1)(SRC-1)(RIP160)(Protein Hin-2)(Renal carcinoma antigen NY-REN-52) [Source:UniProtKB/Swiss-Prot;Acc:Q15788]
ENSG00000169760	NLGN1	ENST00000361589	protein_coding	173302345	1,73E+08	+	1,73E+08	26	+	Neuroligin-1 Precursor [Source:UniProtKB/Swiss-Prot;Acc:Q8N2Q7]
ENSG00000198805	NP	ENST00000361505	protein_coding	20937542	20937741	+	20937617	75	+	Purine nucleoside phosphorylase (PNP)(EC 2.4.2.1)(Inosine phosphorylase) [Source:UniProtKB/Swiss-Prot;Acc:P00491]
ENSG00000198805	NP	ENST00000361505	protein_coding	20937542	20937741	+	20937627	85	+	Purine nucleoside phosphorylase (PNP)(EC 2.4.2.1)(Inosine phosphorylase) [Source:UniProtKB/Swiss-Prot;Acc:P00491]
ENSG00000171936	OR10H3	ENST00000305892	protein_coding	15852203	15852402	+	15852229	26	+	Olfactory receptor 10H3 (Olfactory receptor OR19-24) [Source:UniProtKB/Swiss-Prot;Acc:O60404]
ENSG00000170929	OR1M1	ENST00000305465	protein_coding	9203855	9204054	+	9203947	92	+	Olfactory receptor 1M1 (Olfactory receptor OR19-5)(Olfactory receptor 19-6)(OR19-6) [Source:UniProtKB/Swiss-Prot;Acc:Q8NGA1]
ENSG00000170929	OR1M1	ENST00000429566	protein_coding	9203855	9204054	+	9203947	92	+	Olfactory receptor 1M1 (Olfactory receptor OR19-5)(Olfactory receptor 19-6)(OR19-6) [Source:UniProtKB/Swiss-Prot;Acc:Q8NGA1]
ENSG00000171501	OR1N2	ENST00000373688	protein_coding	125315391	1,25E+08	+	1,25E+08	71	+	Olfactory receptor 1N2 (Olfactory receptor OR9-23) [Source:UniProtKB/Swiss-Prot;Acc:Q8NGR9]
ENSG00000181023	OR56B1	ENST00000317121	protein_coding	5757681	5757880	+	5757764	83	+	Olfactory receptor 56B1 (Olfactory receptor OR11-65) [Source:UniProtKB/Swiss-Prot;Acc:Q8NGI3]
ENSG00000117859	OSBPL9	ENST00000371714	protein_coding	52042851	52043050	+	52042916	65	+	Oxysterol-binding protein-related protein 9 (OSBP-related protein 9)(ORP-9) [Source:UniProtKB/Swiss-Prot;Acc:Q96SU4]
ENSG00000189401	OTUD6A	ENST00000338352	protein_coding	69282341	69282540	+	69282372	31	+	OTU domain-containing protein 6A (DUBA-2) [Source:UniProtKB/Swiss-Prot;Acc:Q7L8S5]
ENSG00000100836	PABPN1	ENST00000216727	protein_coding	23789397	23789596	+	23789438	41	+	Polyadenylate-binding protein 2 (Poly(A)-binding protein 2)(Poly(A)-binding protein II)(PABII)(Polyadenylate-binding nuclear protein 1)(Nuclear poly(A)-binding protein 1) [Source:UniProtKB/Swiss-Prot;Acc:Q86U42]
ENSG00000197996	PADI6	ENST00000434762	protein_coding	17698765	17698964	+	17698837	72	+	Protein-arginine deiminase type-6 (EC 3.5.3.15)(Protein-arginine deiminase type VI)(Peptidylarginine deiminase VI) [Source:UniProtKB/Swiss-Prot;Acc:Q6TGC4]
ENSG00000197996	PADI6	ENST00000413477	protein_coding	17698741	17698940	+	17698837	96	+	Protein-arginine deiminase type-6 (EC 3.5.3.15)(Protein-arginine deiminase type VI)(Peptidylarginine deiminase VI) [Source:UniProtKB/Swiss-Prot;Acc:Q6TGC4]
ENSG00000197996	PADI6	ENST00000358481	protein_coding	17698741	17698940	+	17698837	96	+	Protein-arginine deiminase type-6 (EC 3.5.3.15)(Protein-arginine deiminase type VI)(Peptidylarginine deiminase VI) [Source:UniProtKB/Swiss-Prot;Acc:Q6TGC4]
ENSG00000075891	PAX2	ENST00000370285	protein_coding	102566300	1,03E+08	+	1,03E+08	38	+	Paired box protein Pax-2 [Source:UniProtKB/Swiss-Prot;Acc:Q02962]
ENSG00000081853	PCDHGA12	ENST00000253812	protein_coding	140723601	1,41E+08	+	1,41E+08	80	+	Protocadherin gamma-A2 Precursor (PCDH-gamma-A2) [Source:UniProtKB/Swiss-Prot;Acc:Q9Y5H1]

ENSEMBL55 GeneID	Gene Symbol	ENSEMBL55 TranscriptID	Biotype	Gene start	Gene end	Strand	Motif start	TSS offset	Motif strand	Description
ENSG00000034693	PEX3	ENST00000406025	protein_coding	143811630	1,44E+08	+	1,44E+08	47	+	Peroxisomal biogenesis factor 3 (Peroxin-3)(Peroxisomal assembly protein PEX3) [Source:UniProtKB/Swiss-Prot;Acc:P56589]
ENSG00000100151	PICK1	ENST00000445628	protein_coding	38452318	38452517	+	38452385	67	+	PRKCA-binding protein (Protein kinase C-alpha-binding protein)(Protein interacting with C kinase 1) [Source:UniProtKB/Swiss-Prot;Acc:Q9NRD5]
ENSG00000085514	PILRA	ENST00000432297	protein_coding	99965153	99965352	+	99965203	50	+	Paired immunoglobulin-like type 2 receptor alpha Precursor (Inhibitory receptor PILR-alpha)(Cell surface receptor FDF03) [Source:UniProtKB/Swiss-Prot;Acc:Q9UKJ1]
ENSG00000115556	PLCD4	ENST00000251959	protein_coding	219472622	2,19E+08	+	2,19E+08	90	+	1-phosphatidylinositol-4,5-bisphosphate phosphodiesterase delta-4 (EC 3.1.4.11)(Phosphoinositide phospholipase C delta-4)(Phospholipase C-delta-4)(PLC-delta-4)(hPLCD4) [Source:UniProtKB/Swiss-Prot;Acc:Q9BRC7]
ENSG00000115556	PLCD4	ENST00000444453	protein_coding	219472622	2,19E+08	+	2,19E+08	90	+	1-phosphatidylinositol-4,5-bisphosphate phosphodiesterase delta-4 (EC 3.1.4.11)(Phosphoinositide phospholipase C delta-4)(Phospholipase C-delta-4)(PLC-delta-4)(hPLCD4) [Source:UniProtKB/Swiss-Prot;Acc:Q9BRC7]
ENSG00000115556	PLCD4	ENST00000446503	protein_coding	219472622	2,19E+08	+	2,19E+08	90	+	1-phosphatidylinositol-4,5-bisphosphate phosphodiesterase delta-4 (EC 3.1.4.11)(Phosphoinositide phospholipase C delta-4)(Phospholipase C-delta-4)(PLC-delta-4)(hPLCD4) [Source:UniProtKB/Swiss-Prot;Acc:Q9BRC7]
ENSG00000115556	PLCD4	ENST00000450993	protein_coding	219472632	2,19E+08	+	2,19E+08	80	+	1-phosphatidylinositol-4,5-bisphosphate phosphodiesterase delta-4 (EC 3.1.4.11)(Phosphoinositide phospholipase C delta-4)(Phospholipase C-delta-4)(PLC-delta-4)(hPLCD4) [Source:UniProtKB/Swiss-Prot;Acc:Q9BRC7]
ENSG00000115556	PLCD4	ENST00000417849	protein_coding	219472642	2,19E+08	+	2,19E+08	70	+	1-phosphatidylinositol-4,5-bisphosphate phosphodiesterase delta-4 (EC 3.1.4.11)(Phosphoinositide phospholipase C delta-4)(Phospholipase C-delta-4)(PLC-delta-4)(hPLCD4) [Source:UniProtKB/Swiss-Prot;Acc:Q9BRC7]
ENSG00000115556	PLCD4	ENST00000423512	protein_coding	219472622	2,19E+08	+	2,19E+08	90	+	1-phosphatidylinositol-4,5-bisphosphate phosphodiesterase delta-4 (EC 3.1.4.11)(Phosphoinositide phospholipase C delta-4)(Phospholipase C-delta-4)(PLC-delta-4)(hPLCD4) [Source:UniProtKB/Swiss-Prot;Acc:Q9BRC7]
ENSG00000115556	PLCD4	ENST00000457426	protein_coding	219472622	2,19E+08	+	2,19E+08	90	+	1-phosphatidylinositol-4,5-bisphosphate phosphodiesterase delta-4 (EC 3.1.4.11)(Phosphoinositide phospholipase C delta-4)(Phospholipase C-delta-4)(PLC-delta-4)(hPLCD4) [Source:UniProtKB/Swiss-Prot;Acc:Q9BRC7]
ENSG00000115556	PLCD4	ENST00000396797	protein_coding	219472639	2,19E+08	+	2,19E+08	73	+	1-phosphatidylinositol-4,5-bisphosphate phosphodiesterase delta-4 (EC 3.1.4.11)(Phosphoinositide phospholipase C delta-4)(Phospholipase C-delta-4)(PLC-delta-4)(hPLCD4) [Source:UniProtKB/Swiss-Prot;Acc:Q9BRC7]
ENSG00000175535	PNLIP	ENST00000369221	protein_coding	118305428	1,18E+08	+	1,18E+08	76	+	Pancreatic triacylglycerol lipase Precursor (Pancreatic lipase)(PL)(EC 3.1.1.3) [Source:UniProtKB/Swiss-Prot;Acc:P16233]
ENSG00000170734	POLH	ENST00000453158	protein_coding	43543893	43544092	+	43543992	99	+	DNA polymerase eta (EC 2.7.7.7)(RAD30 homolog A)(Xeroderma pigmentosum variant type protein) [Source:UniProtKB/Swiss-Prot;Acc:Q9Y253]

ENSEMBL55 GeneID	Gene Symbol	ENSEMBL55 TranscriptID	Biotype	Gene start	Gene end	Strand	Motif start	TSS offset	Motif strand	Description
ENSG00000087074	PPP1R15A	ENST00000200453	protein_coding	49375649	49375848	+	49375684	35	+	Protein phosphatase 1 regulatory subunit 15A (Growth arrest and DNA damage-inducible protein GADD34)(Myeloid differentiation primary response protein MyD116 homolog) [Source:UniProtKB/Swiss-Prot;Acc:O75807]
ENSG00000116721	PRAMEF1	ENST00000400814	protein_coding	12854988	12855187	+	12855057	69	+	PRAME family member 1 [Source:UniProtKB/Swiss-Prot;Acc:O95521]
ENSG00000156858	PRR14	ENST00000300835	protein_coding	30662241	30662440	+	30662317	76	+	Proline-rich protein 14 [Source:UniProtKB/Swiss-Prot;Acc:Q9BWN1]
ENSG00000156858	PRR14	ENST00000287463	protein_coding	30662223	30662422	+	30662317	94	+	Proline-rich protein 14 [Source:UniProtKB/Swiss-Prot;Acc:Q9BWN1]
ENSG00000048991	R3HDM1	ENST00000456040	protein_coding	136362426	1,36E+08	+	1,36E+08	81	+	R3H domain-containing protein 1 [Source:UniProtKB/Swiss-Prot;Acc:Q15032]
ENSG00000111445	RFC5	ENST00000420967	protein_coding	118454509	1,18E+08	+	1,18E+08	89	+	Replication factor C subunit 5 (Activator 1 subunit 5)(Replication factor C 36 kDa subunit)(RF-C 36 kDa subunit)(RFC36)(Activator 1 36 kDa subunit)(A1 36 kDa subunit) [Source:UniProtKB/Swiss-Prot;Acc:P40937]
ENSG00000111445	RFC5	ENST00000229043	protein_coding	118454508	1,18E+08	+	1,18E+08	90	+	Replication factor C subunit 5 (Activator 1 subunit 5)(Replication factor C 36 kDa subunit)(RF-C 36 kDa subunit)(RFC36)(Activator 1 36 kDa subunit)(A1 36 kDa subunit) [Source:UniProtKB/Swiss-Prot;Acc:P40937]
ENSG00000111445	RFC5	ENST00000458342	protein_coding	118454508	1,18E+08	+	1,18E+08	90	+	Replication factor C subunit 5 (Activator 1 subunit 5)(Replication factor C 36 kDa subunit)(RF-C 36 kDa subunit)(RFC36)(Activator 1 36 kDa subunit)(A1 36 kDa subunit) [Source:UniProtKB/Swiss-Prot;Acc:P40937]
ENSG00000111445	RFC5	ENST00000392542	protein_coding	118454527	1,18E+08	+	1,18E+08	71	+	Replication factor C subunit 5 (Activator 1 subunit 5)(Replication factor C 36 kDa subunit)(RF-C 36 kDa subunit)(RFC36)(Activator 1 36 kDa subunit)(A1 36 kDa subunit) [Source:UniProtKB/Swiss-Prot;Acc:P40937]
ENSG00000111445	RFC5	ENST00000454402	protein_coding	118454508	1,18E+08	+	1,18E+08	90	+	Replication factor C subunit 5 (Activator 1 subunit 5)(Replication factor C 36 kDa subunit)(RF-C 36 kDa subunit)(RFC36)(Activator 1 36 kDa subunit)(A1 36 kDa subunit) [Source:UniProtKB/Swiss-Prot;Acc:P40937]
ENSG00000159788	RGS12	ENST00000306648	protein_coding	3344231	3344430	+	3344266	35	+	Regulator of G-protein signaling 12 (RGS12) [Source:UniProtKB/Swiss-Prot;Acc:O14924]
ENSG00000018189	RIPX	ENST00000226328	protein_coding	71587696	71587895	+	71587760	64	+	Protein RUFY3 (Rap2-interacting protein x)(RIPx)(Single axon-regulated protein)(Singar) [Source:UniProtKB/Swiss-Prot;Acc:Q7L099]
ENSG00000018189	RIPX	ENST00000226328	protein_coding	71587696	71587895	+	71587750	54	+	Protein RUFY3 (Rap2-interacting protein x)(RIPx)(Single axon-regulated protein)(Singar) [Source:UniProtKB/Swiss-Prot;Acc:Q7L099]
ENSG00000018189	RIPX	ENST00000381006	protein_coding	71587696	71587895	+	71587760	64	+	Protein RUFY3 (Rap2-interacting protein x)(RIPx)(Single axon-regulated protein)(Singar) [Source:UniProtKB/Swiss-Prot;Acc:Q7L099]
ENSG00000018189	RIPX	ENST00000381006	protein_coding	71587696	71587895	+	71587750	54	+	Protein RUFY3 (Rap2-interacting protein x)(RIPx)(Single axon-regulated protein)(Singar) [Source:UniProtKB/Swiss-Prot;Acc:Q7L099]

ENSEMBL55 GeneID	Gene Symbol	ENSEMBL55 TranscriptID	Biotype	Gene start	Gene end	Strand	Motif start	TSS offset	Motif strand	Description
ENSG00000187147	RNF220	ENST00000335497	protein_coding	45097636	45097835	+	45097706	70	+	RING finger protein 220 [Source:UniProtKB/Swiss-Prot;Acc:Q5VTB9]
ENSG00000187147	RNF220	ENST00000440132	protein_coding	45097634	45097833	+	45097706	72	+	RING finger protein 220 [Source:UniProtKB/Swiss-Prot;Acc:Q5VTB9]
ENSG00000198755	RPL10A	ENST00000322203	protein_coding	35436178	35436377	+	35436239	61	+	60S ribosomal protein L10a (CSA-19)(Neural precursor cell expressed developmentally down-regulated protein 6)(NEDD-6) [Source:UniProtKB/Swiss-Prot;Acc:P62906]
ENSG00000142676	RPL11	ENST00000374550	protein_coding	24018294	24018493	+	24018353	59	+	60S ribosomal protein L11 (CLL-associated antigen KW-12) [Source:UniProtKB/Swiss-Prot;Acc:P62913]
ENSG00000167526	RPL13	ENST00000311528	protein_coding	89627090	89627289	+	89627154	64	+	60S ribosomal protein L13 (Breast basic conserved protein 1) [Source:UniProtKB/Swiss-Prot;Acc:P26373]
ENSG00000167526	RPL13	ENST00000393099	protein_coding	89627131	89627330	+	89627154	23	+	60S ribosomal protein L13 (Breast basic conserved protein 1) [Source:UniProtKB/Swiss-Prot;Acc:P26373]
ENSG00000131469	RPL27	ENST00000253788	protein_coding	41150446	41150645	+	41150518	72	+	60S ribosomal protein L27 [Source:UniProtKB/Swiss-Prot;Acc:P61353]
ENSG00000071082	RPL31	ENST00000419276	protein_coding	101618773	1,02E+08	+	1,02E+08	38	+	60S ribosomal protein L31 [Source:UniProtKB/Swiss-Prot;Acc:P62899]
ENSG00000071082	RPL31	ENST00000409038	protein_coding	101618755	1,02E+08	+	1,02E+08	56	+	60S ribosomal protein L31 [Source:UniProtKB/Swiss-Prot;Acc:P62899]
ENSG00000071082	RPL31	ENST00000409650	protein_coding	101618755	1,02E+08	+	1,02E+08	56	+	60S ribosomal protein L31 [Source:UniProtKB/Swiss-Prot;Acc:P62899]
ENSG00000071082	RPL31	ENST00000409733	protein_coding	101618755	1,02E+08	+	1,02E+08	56	+	60S ribosomal protein L31 [Source:UniProtKB/Swiss-Prot;Acc:P62899]
ENSG00000109475	RPL34	ENST00000394667	protein_coding	109541733	1,1E+08	+	1,1E+08	67	+	60S ribosomal protein L34 [Source:UniProtKB/Swiss-Prot;Acc:P49207]
ENSG00000109475	RPL34	ENST00000422834	protein_coding	109541722	1,1E+08	+	1,1E+08	78	+	60S ribosomal protein L34 [Source:UniProtKB/Swiss-Prot;Acc:P49207]
ENSG00000109475	RPL34	ENST00000394668	protein_coding	109541749	1,1E+08	+	1,1E+08	51	+	60S ribosomal protein L34 [Source:UniProtKB/Swiss-Prot;Acc:P49207]
ENSG00000109475	RPL34	ENST00000394665	protein_coding	109541746	1,1E+08	+	1,1E+08	54	+	60S ribosomal protein L34 [Source:UniProtKB/Swiss-Prot;Acc:P49207]
ENSG00000126945	RPL36A	ENST00000372849	protein_coding	100646004	1,01E+08	+	1,01E+08	60	+	60S ribosomal protein L36a (60S ribosomal protein L44)(Cell migration-inducing gene 6 protein) [Source:UniProtKB/Swiss-Prot;Acc:P83881]
ENSG00000126945	RPL36A	ENST00000392994	protein_coding	100646003	1,01E+08	+	1,01E+08	61	+	60S ribosomal protein L36a (60S ribosomal protein L44)(Cell migration-inducing gene 6 protein) [Source:UniProtKB/Swiss-Prot;Acc:P83881]
ENSG00000126945	RPL36A	ENST00000409338	protein_coding	100645999	1,01E+08	+	1,01E+08	65	+	60S ribosomal protein L36a (60S ribosomal protein L44)(Cell migration-inducing gene 6 protein) [Source:UniProtKB/Swiss-Prot;Acc:P83881]
ENSG00000126945	RPL36A	ENST00000409170	protein_coding	100646003	1,01E+08	+	1,01E+08	61	+	60S ribosomal protein L36a (60S ribosomal protein L44)(Cell migration-inducing gene 6 protein) [Source:UniProtKB/Swiss-Prot;Acc:P83881]
ENSG00000130255	RPL36P14	ENST00000347512	protein_coding	5690272	5690471	+	5690364	92	+	60S ribosomal protein L36 [Source:UniProtKB/Swiss-Prot;Acc:Q9Y3U8]
ENSG00000197756	RPL37A	ENST00000420712	protein_coding	217363585	2,17E+08	+	2,17E+08	49	+	60S ribosomal protein L37a [Source:UniProtKB/Swiss-Prot;Acc:P61513]

ENSEMBL55 GeneID	Gene Symbol	ENSEMBL55 TranscriptID	Biotype	Gene start	Gene end	Strand	Motif start	TSS offset	Motif strand	Description
ENSG00000197756	RPL37A	ENST00000446558	protein_coding	217363578	2,17E+08	+	2,17E+08	56	+	60S ribosomal protein L37a [Source:UniProtKB/Swiss-Prot;Acc:P61513]
ENSG00000172809	RPL38	ENST00000439590	protein_coding	72199795	72199994	+	72199888	93	+	60S ribosomal protein L38 [Source:UniProtKB/Swiss-Prot;Acc:P63173]
ENSG00000172809	RPL38	ENST00000311111	protein_coding	72199795	72199994	+	72199888	93	+	60S ribosomal protein L38 [Source:UniProtKB/Swiss-Prot;Acc:P63173]
ENSG00000148303	RPL7A	ENST00000426651	protein_coding	136215107	1,36E+08	+	1,36E+08	41	+	60S ribosomal protein L7a (Surfeit locus protein 3)(PLA-X polypeptide) [Source:UniProtKB/Swiss-Prot;Acc:P62424]
ENSG00000148303	RPL7A	ENST00000323345	protein_coding	136215069	1,36E+08	+	1,36E+08	79	+	60S ribosomal protein L7a (Surfeit locus protein 3)(PLA-X polypeptide) [Source:UniProtKB/Swiss-Prot;Acc:P62424]
ENSG00000146223	RPL7L1	ENST00000397415	protein_coding	42847671	42847870	+	42847733	62	+	60S ribosomal protein L7-like 1 [Source:UniProtKB/Swiss-Prot;Acc:Q6DKI1]
ENSG00000142937	RPS8	ENST00000372209	protein_coding	45241246	45241445	+	45241319	73	+	40S ribosomal protein S8 [Source:UniProtKB/Swiss-Prot;Acc:P62241]
ENSG00000142937	RPS8	ENST00000396651	protein_coding	45241246	45241445	+	45241319	73	+	40S ribosomal protein S8 [Source:UniProtKB/Swiss-Prot;Acc:P62241]
ENSG00000140264	SERF2	ENST00000381359	protein_coding	44069298	44069497	+	44069333	35	+	Small EDRK-rich factor 2 (4F5rel)(h4F5rel)(Gastric cancer-related protein VRG107) [Source:UniProtKB/Swiss-Prot;Acc:P84101]
ENSG00000183918	SH2D1A	ENST00000371139	protein_coding	123480148	1,23E+08	+	1,23E+08	38	+	SH2 domain-containing protein 1A (Signaling lymphocytic activation molecule-associated protein)(SLAM-associated protein)(T-cell signal transduction molecule SAP)(Duncan disease SH2-protein) [Source:UniProtKB/Swiss-Prot;Acc:O60880]
ENSG00000183918	SH2D1A	ENST00000360027	protein_coding	123480148	1,23E+08	+	1,23E+08	38	+	SH2 domain-containing protein 1A (Signaling lymphocytic activation molecule-associated protein)(SLAM-associated protein)(T-cell signal transduction molecule SAP)(Duncan disease SH2-protein) [Source:UniProtKB/Swiss-Prot;Acc:O60880]
ENSG00000131171	SH3BGRL	ENST00000373212	protein_coding	80457303	80457502	+	80457400	97	+	SH3 domain-binding glutamic acid-rich-like protein [Source:UniProtKB/Swiss-Prot;Acc:O75368]
ENSG00000130821	SLC6A8	ENST00000413787	protein_coding	152958509	1,53E+08	+	1,53E+08	74	+	Sodium- and chloride-dependent creatine transporter 1 (Creatine transporter 1)(CT1)(Solute carrier family 6 member 8) [Source:UniProtKB/Swiss-Prot;Acc:P48029]
ENSG00000067066	SP100	ENST00000409897	protein_coding	231281471	2,31E+08	+	2,31E+08	82	+	Nuclear autoantigen Sp-100 (Nuclear dot-associated Sp100 protein)(Speckled 100 kDa)(Lysp100b) [Source:UniProtKB/Swiss-Prot;Acc:P23497]
ENSG00000079263	SP140	ENST00000420434	protein_coding	231090445	2,31E+08	+	2,31E+08	89	+	Nuclear body protein SP140 (Nuclear autoantigen Sp-140)(Speckled 140 kDa)(Lymphoid-restricted homolog of Sp100)(LYSp100 protein) [Source:UniProtKB/Swiss-Prot;Acc:Q13342]
ENSG00000079263	SP140	ENST00000350136	protein_coding	231090445	2,31E+08	+	2,31E+08	89	+	Nuclear body protein SP140 (Nuclear autoantigen Sp-140)(Speckled 140 kDa)(Lymphoid-restricted homolog of Sp100)(LYSp100 protein) [Source:UniProtKB/Swiss-Prot;Acc:Q13342]
ENSG00000079263	SP140	ENST00000343805	protein_coding	231090445	2,31E+08	+	2,31E+08	89	+	Nuclear body protein SP140 (Nuclear autoantigen Sp-140)(Speckled 140 kDa)(Lymphoid-restricted homolog of

ENSEMBL55 GeneID	Gene Symbol	ENSEMBL55 TranscriptID	Biotype	Gene start	Gene end	Strand	Motif start	TSS offset	Motif strand	Description
										Sp100)(LYSp100 protein) [Source:UniProtKB/Swiss-Prot;Acc:Q13342]
ENSG00000079263	SP140	ENST00000392045	protein_coding	231090445	2,31E+08	+	2,31E+08	89	+	Nuclear body protein SP140 (Nuclear autoantigen Sp-140)(Speckled 140 kDa)(Lymphoid-restricted homolog of Sp100)(LYSp100 protein) [Source:UniProtKB/Swiss-Prot;Acc:Q13342]
ENSG00000079263	SP140	ENST00000373645	protein_coding	231090445	2,31E+08	+	2,31E+08	89	+	Nuclear body protein SP140 (Nuclear autoantigen Sp-140)(Speckled 140 kDa)(Lymphoid-restricted homolog of Sp100)(LYSp100 protein) [Source:UniProtKB/Swiss-Prot;Acc:Q13342]
ENSG00000079263	SP140	ENST00000417495	protein_coding	231090457	2,31E+08	+	2,31E+08	77	+	Nuclear body protein SP140 (Nuclear autoantigen Sp-140)(Speckled 140 kDa)(Lymphoid-restricted homolog of Sp100)(LYSp100 protein) [Source:UniProtKB/Swiss-Prot;Acc:Q13342]
ENSG00000079263	SP140	ENST00000392044	protein_coding	231090445	2,31E+08	+	2,31E+08	89	+	Nuclear body protein SP140 (Nuclear autoantigen Sp-140)(Speckled 140 kDa)(Lymphoid-restricted homolog of Sp100)(LYSp100 protein) [Source:UniProtKB/Swiss-Prot;Acc:Q13342]
ENSG00000068781	STON1-GTF2A1L	ENST00000444932	protein_coding	48809435	48809634	+	48809497	62	+	Stonin-1 (Stoned B-like factor) [Source:UniProtKB/Swiss-Prot;Acc:Q9Y6Q2]
ENSG00000152455	SUV39H2	ENST00000358298	protein_coding	14923499	14923698	+	14923572	73	+	Histone-lysine N-methyltransferase SUV39H2 (EC 2.1.1.43)(Suppressor of variegation 3-9 homolog 2)(Su(var)3-9 homolog 2)(Histone H3-K9 methyltransferase 2)(H3-K9-HMTase 2)(Lysine N-methyltransferase 1B) [Source:UniProtKB/Swiss-Prot;Acc:Q9H5I1]
ENSG00000152455	SUV39H2	ENST00000452301	protein_coding	14923501	14923700	+	14923572	71	+	Histone-lysine N-methyltransferase SUV39H2 (EC 2.1.1.43)(Suppressor of variegation 3-9 homolog 2)(Su(var)3-9 homolog 2)(Histone H3-K9 methyltransferase 2)(H3-K9-HMTase 2)(Lysine N-methyltransferase 1B) [Source:UniProtKB/Swiss-Prot;Acc:Q9H5I1]
ENSG00000065882	TBC1D1	ENST00000443855	protein_coding	38046095	38046294	+	38046191	96	+	TBC1 domain family member 1 [Source:UniProtKB/Swiss-Prot;Acc:Q86TI0]
ENSG00000065882	TBC1D1	ENST00000421339	protein_coding	38047446	38047645	+	38047471	25	+	TBC1 domain family member 1 [Source:UniProtKB/Swiss-Prot;Acc:Q86TI0]
ENSG00000105254	TBCB	ENST00000221855	protein_coding	36605888	36606087	+	36605938	50	+	Tubulin folding cofactor B (Tubulin-specific chaperone B)(Cytoskeleton-associated protein 1)(Cytoskeleton-associated protein CKAPI) [Source:UniProtKB/Swiss-Prot;Acc:Q99426]
ENSG00000162782	TDRD5	ENST00000367613	protein_coding	179599442	1,8E+08	+	1,8E+08	72	+	Tudor domain-containing protein 5 [Source:UniProtKB/Swiss-Prot;Acc:Q8NAT2]
ENSG00000163513	TGFBR2	ENST00000439925	protein_coding	30713129	30713328	+	30713215	86	+	TGF-beta receptor type-2 Precursor (EC 2.7.11.30)(Transforming growth factor-beta receptor type II)(TGF-beta receptor type II)(TGF-beta type II receptor)(TbetaR-II)(TGFR-2) [Source:UniProtKB/Swiss-Prot;Acc:P37173]
ENSG00000137251	TINAG	ENST00000370865	protein_coding	54173349	54173548	+	54173373	24	+	Tubulointerstitial nephritis antigen (TIN-Ag) [Source:UniProtKB/Swiss-Prot;Acc:Q9UJW2]

ENSEMBL55 GeneID	Gene Symbol	ENSEMBL55 TranscriptID	Biotype	Gene start	Gene end	Strand	Motif start	TSS offset	Motif strand	Description
ENSG00000178826	TMEM139	ENST00000409102	protein_coding	142977050	1,43E+08	+	1,43E+08	42	+	Transmembrane protein 139 Precursor [Source:UniProtKB/Swiss-Prot;Acc:Q8IV31]
ENSG00000231312	TMEM178	ENST00000437068	protein_coding	39892122	39892321	+	39892173	51	+	
ENSG00000188001	TPRG1	ENST00000345063	protein_coding	188889763	1,89E+08	+	1,89E+08	63	+	Tumor protein p63-regulated gene 1 protein (Protein FAM79B) [Source:UniProtKB/Swiss-Prot;Acc:Q6ZUI0]
ENSG00000188056	TREML4	ENST00000448827	protein_coding	41196485	41196684	+	41196534	49	+	Trem-like transcript 4 protein Precursor (TLT-4)(Triggering receptor expressed on myeloid cells-like protein 4) [Source:UniProtKB/Swiss-Prot;Acc:Q6UXN2]
ENSG00000101255	TRIB3	ENST00000449710	protein_coding	361890	362089	+	361989	99	+	Tribbles homolog 3 (TRB-3)(Neuronal cell death-inducible putative kinase)(p65-interacting inhibitor of NF-kappa-B)(SINK) [Source:UniProtKB/Swiss-Prot;Acc:Q96RU7]
ENSG00000163462	TRIM46	ENST00000368380	protein_coding	155147391	1,55E+08	+	1,55E+08	38	+	Tripartite motif-containing protein 46 (Tripartite, fibronectin type-III and C-terminal SPRY motif protein)(Gene Y protein)(GeneY) [Source:UniProtKB/Swiss-Prot;Acc:Q7Z4K8]
ENSG00000130338	TULP4	ENST00000367097	protein_coding	158733692	1,59E+08	+	1,59E+08	92	+	Tubby-related protein 4 (Tubby-like protein 4)(Tubby superfamily protein) [Source:UniProtKB/Swiss-Prot;Acc:Q9NRJ4]
ENSG00000130338	TULP4	ENST00000367094	protein_coding	158733692	1,59E+08	+	1,59E+08	92	+	Tubby-related protein 4 (Tubby-like protein 4)(Tubby superfamily protein) [Source:UniProtKB/Swiss-Prot;Acc:Q9NRJ4]
ENSG00000167165	UGT1A8	ENST00000406651	protein_coding	234602338	2,35E+08	+	2,35E+08	42	+	UDP-glucuronosyltransferase 1-8 Precursor (EC 2.4.1.17)(UDP-glucuronosyltransferase 1A8)(UDPGT)(UGT1*8)(UGT1-08)(UGT1.8)(UGT-1H)(UGT1H) [Source:UniProtKB/Swiss-Prot;Acc:Q9HAW9]
ENSG00000168883	USP39	ENST00000450066	protein_coding	85839449	85839648	+	85839501	52	+	U4/U6.U5 tri-snRNP-associated protein 2 (U4/U6.U5 tri-snRNP-associated 65 kDa protein)(65K)(Inactive ubiquitin-specific peptidase 39)(SAD1 homolog) [Source:UniProtKB/Swiss-Prot;Acc:Q53GS9]
ENSG00000101443	WFDC2	ENST00000342873	protein_coding	44108485	44108684	+	44108531	46	+	WAP four-disulfide core domain protein 2 Precursor (Major epididymis-specific protein E4)(Epididymal secretory protein E4)(Putative protease inhibitor WAP5) [Source:UniProtKB/Swiss-Prot;Acc:Q14508]
ENSG00000158552	ZFAND2B	ENST00000436556	protein_coding	220059959	2,2E+08	+	2,2E+08	65	+	AN1-type zinc finger protein 2B [Source:UniProtKB/Swiss-Prot;Acc:Q8WV99]
ENSG00000162664	ZNF326	ENST00000361911	protein_coding	90460678	90460877	+	90460730	52	+	Zinc finger protein 326 [Source:UniProtKB/Swiss-Prot;Acc:Q5BKZ1]
ENSG00000162664	ZNF326	ENST00000394583	protein_coding	90460671	90460870	+	90460730	59	+	Zinc finger protein 326 [Source:UniProtKB/Swiss-Prot;Acc:Q5BKZ1]
ENSG00000162664	ZNF326	ENST00000340281	protein_coding	90460678	90460877	+	90460730	52	+	Zinc finger protein 326 [Source:UniProtKB/Swiss-Prot;Acc:Q5BKZ1]
ENSG00000162664	ZNF326	ENST00000370447	protein_coding	90460696	90460895	+	90460730	34	+	Zinc finger protein 326 [Source:UniProtKB/Swiss-Prot;Acc:Q5BKZ1]
ENSG00000148143	ZNF462	ENST00000457913	protein_coding	109625378	1,1E+08	+	1,1E+08	44	+	Zinc finger protein 462 [Source:UniProtKB/Swiss-Prot;Acc:Q96JM2]
ENSG00000148143	ZNF462	ENST00000277225	protein_coding	109625378	1,1E+08	+	1,1E+08	44	+	Zinc finger protein 462 [Source:UniProtKB/Swiss-Prot;Acc:Q96JM2]

ENSEMBL55 GeneID	Gene Symbol	ENSEMBL55 TranscriptID	Biotype	Gene start	Gene end	Strand	Motif start	TSS offset	Motif strand	Description
ENSG00000235579	AC007283.4	ENST00000424739	pseudogene	202027204	2,02E+08	-	2,02E+08	74	-	
ENSG00000223739	AC007389.2	ENST00000434630	pseudogene	65739011	65739210	-	65739113	97	-	
ENSG00000234131	AC008175.5	ENST00000439309	pseudogene	22014001	22014200	-	22014142	58	-	
ENSG00000226116	AC009976.3	ENST00000413550	pseudogene	17383791	17383990	-	17383910	80	-	
ENSG00000226116	AC009976.3	ENST00000413550	pseudogene	17383791	17383990	-	17383900	90	-	
ENSG00000236478	AC012513.4	ENST00000441511	pseudogene	217040556	2,17E+08	-	2,17E+08	97	-	
ENSG00000233829	AC017078.1	ENST00000416146	pseudogene	46231249	46231448	-	46231371	77	-	
ENSG00000232526	AC025750.6	ENST00000447826	pseudogene	42690938	42691137	-	42691100	37	-	
ENSG00000213228	AC037475.9-1	ENST00000359713	pseudogene	58511455	58511654	-	58511594	60	-	
ENSG00000226285	AC091813.2	ENST00000416748	pseudogene	79173970	79174169	-	79174092	77	-	
ENSG00000230118	AC092569.2	ENST00000455192	pseudogene	30969368	30969567	-	30969522	45	-	
ENSG00000230118	AC092569.2	ENST00000455192	pseudogene	30969368	30969567	-	30969495	72	-	
ENSG00000232766	AC098614.3	ENST00000438957	pseudogene	27744860	27745059	-	27744961	98	-	
ENSG00000228112	AC112220.2	ENST00000434628	pseudogene	33768897	33769096	-	33769038	58	-	
ENSG00000224252	AC113618.2	ENST00000416747	pseudogene	239643989	2,4E+08	-	2,4E+08	45	-	
ENSG00000214203	AC126474.6	ENST00000397774	pseudogene	100403417	1E+08	-	1E+08	94	-	
ENSG00000213450	AC131005.3	ENST00000394254	pseudogene	77366565	77366764	-	77366721	43	-	
ENSG00000224366	AC138472.4	ENST00000441814	pseudogene	45073611	45073810	-	45073768	42	-	
ENSG00000229735	AF241726.3	ENST00000446400	pseudogene	38344318	38344517	-	38344445	72	-	
ENSG00000237704	AP004289.2	ENST00000444413	pseudogene	100353790	1E+08	-	1E+08	54	-	
ENSG00000230071	CTA-229A8.5	ENST00000438116	pseudogene	40983808	40984007	-	40983979	28	-	
ENSG00000230870	FBXW11P1	ENST00000425623	pseudogene	33000714	33000913	-	33000884	29	-	
ENSG00000230870	FBXW11P1	ENST00000393804	pseudogene	33000714	33000913	-	33000884	29	-	
ENSG00000231234	GS1-124K5.5	ENST00000438081	pseudogene	65888681	65888880	-	65888800	80	-	
ENSG00000223472	IGHVII-62-1	ENST00000420094	pseudogene	107106590	1,07E+08	-	1,07E+08	90	-	
ENSG00000230643	LA16c-60G3.5	ENST00000447704	pseudogene	16389403	16389602	-	16389521	81	-	
ENSG00000223864	NPM1P19	ENST00000450070	pseudogene	37606866	37607065	-	37606978	87	-	
ENSG00000180636	OR4E1P	ENST00000303519	pseudogene	22138951	22139150	-	22139126	24	-	
ENSG00000180636	OR4E1P	ENST00000413808	pseudogene	22138951	22139150	-	22139126	24	-	
ENSG00000226616	OR52M2P	ENST00000414298	pseudogene	4536955	4537154	-	4537074	80	-	
ENSG00000237115	RP1-131F15.2	ENST00000455305	pseudogene	132149354	1,32E+08	-	1,32E+08	59	-	
ENSG00000216360	RP1-182O16.2	ENST00000403053	pseudogene	5818400	5818599	-	5818500	99	-	
ENSG00000184106	RP1-229K20.2	ENST00000457327	pseudogene	41185486	41185685	-	41185624	61	-	triggering receptor expressed on myeloid cells-like 3 (TREML3), non-coding RNA [Source:RefSeq DNA;Acc:NR_027256]

ENSEMBL55 GeneID	Gene Symbol	ENSEMBL55 TranscriptID	Biotype	Gene start	Gene end	Strand	Motif start	TSS offset	Motif strand	Description
ENSG00000213856	RP1-8N8.10	ENST00000423609	pseudogene	49397753	49397952	-	49397918	34	-	
ENSG00000213856	RP1-8N8.10	ENST00000396571	pseudogene	49397762	49397961	-	49397918	43	-	
ENSG00000228810	RP11-163F8.3	ENST00000428966	pseudogene	17591117	17591316	-	17591271	45	-	
ENSG00000219448	RP11-193H22.2	ENST00000401486	pseudogene	158003110	1,58E+08	-	1,58E+08	27	-	
ENSG00000232154	RP11-193H5.5	ENST00000433123	pseudogene	238112244	2,38E+08	-	2,38E+08	69	-	
ENSG00000232154	RP11-193H5.5	ENST00000433123	pseudogene	238112244	2,38E+08	-	2,38E+08	79	-	
ENSG00000215104	RP11-217H19.1	ENST00000451110	pseudogene	79546237	79546436	-	79546352	84	-	
ENSG00000227254	RP11-218I21.1	ENST00000451853	pseudogene	34657248	34657447	-	34657410	37	-	
ENSG00000214089	RP11-256K7.1	ENST00000397509	pseudogene	44910402	44910601	-	44910580	21	-	
ENSG00000214089	RP11-256K7.1	ENST00000392269	pseudogene	44910402	44910601	-	44910580	21	-	
ENSG00000232805	RP11-280G19.1	ENST00000433227	pseudogene	94866941	94867140	-	94867080	60	-	
ENSG00000218418	RP11-296E7.1	ENST00000401578	pseudogene	80777655	80777854	-	80777819	35	-	
ENSG00000237679	RP11-307E17.2	ENST00000458323	pseudogene	97050631	97050830	-	97050796	34	-	
ENSG00000238190	RP11-637B23.3	ENST00000453854	pseudogene	50674349	50674548	-	50674490	58	-	
ENSG00000224368	RP11-73B2.5	ENST00000440565	pseudogene	62865579	62865778	-	62865695	83	-	
ENSG00000231264	RP11-73B2.7	ENST00000422172	pseudogene	63398688	63398887	-	63398809	78	-	
ENSG00000215604	RP11-77P19.1	ENST00000342944	pseudogene	19059562	19059761	-	19059667	94	-	
ENSG00000238026	RP11-78E6.1	ENST00000419971	pseudogene	175832126	1,76E+08	-	1,76E+08	79	-	
ENSG00000230986	RP13-204A15.1	ENST00000446138	pseudogene	73352750	73352949	-	73352860	89	-	
ENSG00000232882	RP4-665J23.3	ENST00000420457	pseudogene	91358970	91359169	-	91359094	75	-	
ENSG00000226212	TRGV6	ENST00000417928	pseudogene	38380400	38380599	-	38380527	72	-	
ENSG00000226212	TRGV6	ENST00000417928	pseudogene	38380400	38380599	-	38380537	62	-	
ENSG00000236405	UBQLN1P	ENST00000441056	pseudogene	30331858	30332057	-	30331968	89	-	
ENSG00000236405	UBQLN1P	ENST00000441056	pseudogene	30331858	30332057	-	30331978	79	-	
ENSG00000227394	AC007386.3	ENST00000455212	pseudogene	65187569	65187768	-	65187739	29	+	
ENSG00000229129	AC007742.5	ENST00000415776	pseudogene	17220286	17220485	-	17220427	58	+	
ENSG00000230476	AC008175.9	ENST00000417699	pseudogene	22110509	22110708	-	22110652	56	+	
ENSG00000229000	AC008753.2	ENST00000413308	pseudogene	54276335	54276534	-	54276462	72	+	
ENSG00000226926	AC010423.1	ENST00000412514	pseudogene	102862122	1,03E+08	-	1,03E+08	47	+	
ENSG00000205215	AC015818.3	ENST00000379222	pseudogene	20431801	20432000	-	20431975	25	+	
ENSG00000232202	AC098824.6	ENST00000453565	pseudogene	43054558	43054757	-	43054695	62	+	

ENSEMBL55 GeneID	Gene Symbol	ENSEMBL55 TranscriptID	Biotype	Gene start	Gene end	Strand	Motif start	TSS offset	Motif strand	Description
ENSG00000231489	AF196972.10	ENST00000423117	pseudogene	48281654	48281853	-	48281776	77	+	
ENSG00000213018	AL590762.11	ENST00000391782	pseudogene	70640165	70640364	-	70640341	23	+	
ENSG00000183545	AL928768.1-1	ENST00000333301	pseudogene	106166981	1,06E+08	-	1,06E+08	74	+	
ENSG00000236493	EIF2S2P3	ENST00000439671	pseudogene	94429301	94429500	-	94429474	26	+	
ENSG00000236493	EIF2S2P3	ENST00000428356	pseudogene	94429301	94429500	-	94429474	26	+	
ENSG00000234468	IGHV3-62	ENST00000426630	pseudogene	107099389	1,07E+08	-	1,07E+08	68	+	
ENSG00000218074	IGKV2-29	ENST00000401932	pseudogene	89534194	89534393	-	89534326	67	+	
ENSG00000220110	RP1-6P5.2	ENST00000406055	pseudogene	128960599	1,29E+08	-	1,29E+08	35	+	
ENSG00000236960	RP11-265G8.3	ENST00000427935	pseudogene	19780029	19780228	-	19780187	41	+	
ENSG00000223746	RP11-272J7.1	ENST00000447108	pseudogene	44237599	44237798	-	44237703	95	+	
ENSG00000234115	RP11-288G3.4	ENST00000440581	pseudogene	7517874	7518073	-	7518026	47	+	
ENSG00000229704	RP11-29A1.2	ENST00000447161	pseudogene	184964030	1,85E+08	-	1,85E+08	26	+	
ENSG00000215105	RP11-324B6.1	ENST00000427129	pseudogene	74965347	74965546	-	74965487	59	+	
ENSG00000232601	RP11-393N4.1	ENST00000411665	pseudogene	174020878	1,74E+08	-	1,74E+08	57	+	
ENSG00000235318	RP11-3N2.11	ENST00000425022	pseudogene	63565764	63565963	-	63565884	79	+	
ENSG00000235318	RP11-3N2.11	ENST00000435146	pseudogene	63565755	63565954	-	63565884	70	+	
ENSG00000230495	RP11-462D18.2	ENST00000434165	pseudogene	17580253	17580452	-	17580420	32	+	
ENSG00000238079	RP11-72I2.1	ENST00000442911	pseudogene	108693932	1,09E+08	-	1,09E+08	68	+	
ENSG00000236241	RP11-744O11.2	ENST00000454746	pseudogene	175394390	1,75E+08	-	1,75E+08	42	+	
ENSG00000219532	RP3-323K23.3	ENST00000407837	pseudogene	132141032	1,32E+08	-	1,32E+08	67	+	
ENSG00000237381	RP3-455H14.1	ENST00000414840	pseudogene	92643786	92643985	-	92643922	63	+	
ENSG00000237622	RP5-1033K19.1	ENST00000434209	pseudogene	68841147	68841346	-	68841269	77	+	
ENSG00000231276	RP5-1043E3.1	ENST00000422443	pseudogene	39522520	39522719	-	39522625	94	+	
ENSG00000231293	RPL36AP6	ENST00000435770	pseudogene	110596911	1,11E+08	-	1,11E+08	92	+	
ENSG00000233467	U40455.1	ENST00000422702	pseudogene	147280199	1,47E+08	-	1,47E+08	89	+	
ENSG00000233467	U40455.1	ENST00000411473	pseudogene	147280199	1,47E+08	-	1,47E+08	89	+	
ENSG00000214185	XPOTP1	ENST00000443317	pseudogene	32803499	32803698	-	32803600	98	+	
ENSG00000214185	XPOTP1	ENST00000389162	pseudogene	32803499	32803698	-	32803600	98	+	
ENSG00000204789	ZNF204	ENST00000377448	pseudogene	27339105	27339304	-	27339210	94	+	zinc finger protein 204 pseudogene (ZNF204), non-coding RNA [Source:RefSeq DNA;Acc:NR_002722]
ENSG00000214607	AB020862.1	ENST00000398655	pseudogene	17327027	17327226	+	17327076	49	-	
ENSG00000233167	AC005039.3	ENST00000458319	pseudogene	14230420	14230619	+	14230513	93	-	
ENSG00000182965	AC005192.5	ENST00000330645	pseudogene	112160522	1,12E+08	+	1,12E+08	32	-	

ENSEMBL55 GeneID	Gene Symbol	ENSEMBL55 TranscriptID	Biotype	Gene start	Gene end	Strand	Motif start	TSS offset	Motif strand	Description
ENSG00000233448	AC007000.9	ENST00000443097	pseudogene	76668797	76668996	+	76668868	71	-	postmeiotic segregation increased 2-like 5-like (LOC100132832), mRNA [Source:RefSeq DNA;Acc:NM_001129851]
ENSG00000233130	AC007241.5	ENST00000413237	pseudogene	17899340	17899539	+	17899369	29	-	
ENSG00000213189	AC010093.1	ENST00000392798	pseudogene	159860487	1,6E+08	+	1,6E+08	47	-	
ENSG00000223406	AC010682.7	ENST00000449659	pseudogene	25249015	25249214	+	25249044	29	-	
ENSG00000224240	AC019099.2	ENST00000420810	pseudogene	26046052	26046251	+	26046128	76	-	
ENSG00000229234	AC025227.2	ENST00000423480	pseudogene	21705958	21706157	+	21706045	87	-	
ENSG00000224962	AC072046.11	ENST00000454471	pseudogene	166408976	1,66E+08	+	1,66E+08	47	-	
ENSG00000230192	AC073387.2	ENST00000453261	pseudogene	109300151	1,09E+08	+	1,09E+08	26	-	
ENSG00000227470	AC073415.2	ENST00000449240	pseudogene	109494764	1,09E+08	+	1,09E+08	83	-	
ENSG00000213871	AC092798.3	ENST00000396646	pseudogene	25796416	25796615	+	25796478	62	-	
ENSG00000227965	AC098592.6	ENST00000413158	pseudogene	16928245	16928444	+	16928330	85	-	
ENSG00000213128	AC109327.10	ENST00000392422	pseudogene	78516003	78516202	+	78516082	79	-	
ENSG00000226559	AC134980.3-2	ENST00000438898	pseudogene	22332586	22332785	+	22332651	65	-	
ENSG00000203430	AC140061.14-2	ENST00000366249	pseudogene	50688917	50689116	+	50688953	36	-	
ENSG00000229349	ACTGP9	ENST00000457622	pseudogene	46172466	46172665	+	46172514	48	-	
ENSG00000232109	AL022344.2	ENST00000435210	pseudogene	43156176	43156375	+	43156213	37	-	
ENSG00000234500	GS1-124K5.10	ENST00000448776	pseudogene	65976543	65976742	+	65976623	80	-	
ENSG00000237958	OR2Q1P	ENST00000449203	pseudogene	143677998	1,44E+08	+	1,44E+08	53	-	
ENSG00000224300	OR51P1P	ENST00000447547	pseudogene	5036368	5036567	+	5036438	70	-	
ENSG00000205578	POM121B	ENST00000411631	pseudogene	72705857	72706056	+	72705895	38	-	
ENSG00000175718	RBMXL3	ENST00000321377	pseudogene	114423963	1,14E+08	+	1,14E+08	44	-	RNA binding motif protein, X-linked-like 3 (RBMXL3), mRNA [Source:RefSeq DNA;Acc:NM_001145346]
ENSG00000230169	RP11-11A18.1	ENST00000419341	pseudogene	113551118	1,14E+08	+	1,14E+08	95	-	
ENSG00000234748	RP11-183K14.1	ENST00000421173	pseudogene	145619053	1,46E+08	+	1,46E+08	54	-	
ENSG00000235060	RP11-254N18.1	ENST00000450197	pseudogene	180403935	1,8E+08	+	1,8E+08	67	-	
ENSG00000237193	RP11-275O4.4	ENST00000420896	pseudogene	227669954	2,28E+08	+	2,28E+08	94	-	
ENSG00000220514	RP11-331O9.6	ENST00000402379	pseudogene	153989724	1,54E+08	+	1,54E+08	67	-	
ENSG00000214961	RP11-355K23B.1	ENST00000399325	pseudogene	133406317	1,33E+08	+	1,33E+08	30	-	
ENSG00000227882	RP11-380N8.3	ENST00000431978	pseudogene	26808350	26808549	+	26808429	79	-	
ENSG00000227499	RP11-419M24.4	ENST00000452998	pseudogene	55945605	55945804	+	55945635	30	-	
ENSG00000229347	RP11-	ENST00000427910	pseudogene	35077044	35077243	+	35077142	98	-	

ENSEMBL55 GeneID	Gene Symbol	ENSEMBL55 TranscriptID	Biotype	Gene start	Gene end	Strand	Motif start	TSS offset	Motif strand	Description
	504E21.1									
ENSG00000228464	RP11-617O8.1	ENST00000438690	pseudogene	26363353	26363552	+	26363413	60	-	
ENSG00000223559	RP11-700P18.1	ENST00000449426	pseudogene	56355923	56356122	+	56356009	86	-	
ENSG00000228158	RP3-323B6.2	ENST00000426165	pseudogene	64628131	64628330	+	64628213	82	-	
ENSG00000220370	RP3-399J4.2	ENST00000404458	pseudogene	95054455	95054654	+	95054486	31	-	
ENSG00000218893	RP3-451B15.3	ENST00000405924	pseudogene	12319685	12319884	+	12319712	27	-	
ENSG00000231005	RP3-481F12.1	ENST00000455840	pseudogene	55903602	55903801	+	55903680	78	-	
ENSG00000217330	RP3-509L4.1	ENST00000402595	pseudogene	118910233	1,19E+08	+	1,19E+08	78	-	
ENSG00000229919	TCEB1P3	ENST00000439303	pseudogene	10216193	10216392	+	10216255	62	-	
ENSG00000237347	AC004461.4	ENST00000412461	pseudogene	19043327	19043526	+	19043348	21	+	
ENSG00000228568	AC006461.2	ENST00000412637	pseudogene	72515914	72516113	+	72516012	98	+	
ENSG00000233448	AC007000.9	ENST00000443097	pseudogene	76668797	76668996	+	76668869	72	+	postmeiotic segregation increased 2-like 5-like (LOC100132832), mRNA [Source:RefSeq DNA;Acc:NM_001129851]
ENSG00000213092	AC008507.11-2	ENST00000392268	pseudogene	30528330	30528529	+	30528393	63	+	
ENSG00000213437	AC012183.9-1	ENST00000394188	pseudogene	58811118	58811317	+	58811154	36	+	
ENSG00000224291	AC012671.2	ENST00000451912	pseudogene	88727597	88727796	+	88727656	59	+	
ENSG00000213264	AC020633.3	ENST00000393181	pseudogene	132120097	1,32E+08	+	1,32E+08	57	+	
ENSG00000233546	AC022486.2	ENST00000451909	pseudogene	18041724	18041923	+	18041779	55	+	
ENSG00000229890	AC023672.1	ENST00000440793	pseudogene	108715566	1,09E+08	+	1,09E+08	68	+	
ENSG00000181735	AC068988.20	ENST00000314555	pseudogene	53157190	53157389	+	53157277	87	+	
ENSG00000235157	AC073539.3	ENST00000443755	pseudogene	22321358	22321557	+	22321438	80	+	
ENSG00000226034	AC092845.1	ENST00000448444	pseudogene	101276674	1,01E+08	+	1,01E+08	31	+	
ENSG00000231583	AC108938.3	ENST00000451204	pseudogene	111257677	1,11E+08	+	1,11E+08	77	+	
ENSG00000203430	AC140061.14-2	ENST00000366249	pseudogene	50688917	50689116	+	50688954	37	+	
ENSG00000234106	AP004242.2-1	ENST00000425126	pseudogene	93268635	93268834	+	93268700	65	+	
ENSG00000236931	BX664608.1	ENST00000429748	pseudogene	40812904	40813103	+	40812969	65	+	
ENSG00000230584	CCT5P2	ENST00000432974	pseudogene	79483054	79483253	+	79483107	53	+	
ENSG00000228238	GS1-304P7.2	ENST00000456921	pseudogene	186547411	1,87E+08	+	1,87E+08	26	+	
ENSG00000237127	LL22NC03-84E4.8	ENST00000458673	pseudogene	23177041	23177240	+	23177124	83	+	
ENSG00000229707	RP11-100J16.4	ENST00000429280	pseudogene	41630397	41630596	+	41630462	65	+	
ENSG00000236777	RP11-108B14.1	ENST00000426134	pseudogene	22452021	22452220	+	22452081	60	+	

ENSEMBL55 GeneID	Gene Symbol	ENSEMBL55 TranscriptID	Biotype	Gene start	Gene end	Strand	Motif start	TSS offset	Motif strand	Description
ENSG00000229622	RP11-10E18.4	ENST00000442927	pseudogene	96344792	96344991	+	96344861	69	+	
ENSG00000227850	RP11-123D12.1	ENST00000419651	pseudogene	106240661	1,06E+08	+	1,06E+08	93	+	
ENSG00000235076	RP11-149B7.1	ENST00000449535	pseudogene	21932512	21932711	+	21932598	86	+	
ENSG00000234145	RP11-214O11.1	ENST00000432820	pseudogene	48337595	48337794	+	48337688	93	+	
ENSG00000235060	RP11-254N18.1	ENST00000450197	pseudogene	180403935	1,8E+08	+	1,8E+08	25	+	
ENSG00000229629	RP11-302K17.4	ENST00000454105	pseudogene	103849725	1,04E+08	+	1,04E+08	68	+	
ENSG00000215034	RP11-331G2.4	ENST00000399472	pseudogene	39255690	39255889	+	39255758	68	+	
ENSG00000237210	RP11-339F13.2	ENST00000425993	pseudogene	55311940	55312139	+	55312032	92	+	
ENSG00000232822	RP11-381G8.1	ENST00000451428	pseudogene	39485376	39485575	+	39485445	69	+	
ENSG00000232822	RP11-381G8.1	ENST00000423634	pseudogene	39485380	39485579	+	39485445	65	+	
ENSG00000229849	RP11-393K10.1	ENST00000431627	pseudogene	158848536	1,59E+08	+	1,59E+08	48	+	
ENSG00000233162	RP11-456E1.2	ENST00000418771	pseudogene	72694399	72694598	+	72694435	36	+	
ENSG00000232525	RP11-51B10.3	ENST00000445923	pseudogene	35164138	35164337	+	35164219	81	+	
ENSG00000214020	RP11-560D2.1	ENST00000397259	pseudogene	30935483	30935682	+	30935519	36	+	
ENSG00000214020	RP11-560D2.1	ENST00000430740	pseudogene	30935483	30935682	+	30935519	36	+	
ENSG00000230788	RP11-560L11.1	ENST00000434586	pseudogene	105314472	1,05E+08	+	1,05E+08	32	+	
ENSG00000232203	RP11-572H4.2	ENST00000445887	pseudogene	31254043	31254242	+	31254079	36	+	
ENSG00000232185	RP11-710N8.2	ENST00000441051	pseudogene	116107222	1,16E+08	+	1,16E+08	87	+	
ENSG00000228024	RP11-71A24.3	ENST00000416662	pseudogene	75657661	75657860	+	75657691	30	+	
ENSG00000235926	RP13-650G11.1	ENST00000458329	pseudogene	100525990	1,01E+08	+	1,01E+08	66	+	
ENSG00000237977	RP3-430N8.8	ENST00000424380	pseudogene	31298206	31298405	+	31298266	60	+	
ENSG00000218586	RP4-791C19.1	ENST00000407916	pseudogene	55001392	55001591	+	55001424	32	+	
ENSG00000229344	RP5-857K21.7	ENST00000427426	pseudogene	568137	568336	+	568200	63	+	
ENSG00000227298	RP5-905H7.5	ENST00000454154	pseudogene	62803089	62803288	+	62803160	71	+	
ENSG00000213400	RPL12P18	ENST00000393866	pseudogene	85101654	85101853	+	85101699	45	+	
ENSG00000234187	SCYE1P	ENST00000418670	pseudogene	14108046	14108245	+	14108136	90	+	
ENSG00000214273	U85056.1-1	ENST00000397992	pseudogene	190963386	1,91E+08	+	1,91E+08	69	+	
ENSG00000214273	U85056.1-1	ENST00000397992	pseudogene	190963386	1,91E+08	+	1,91E+08	98	+	
ENSG00000073905	VDAC1LP	ENST00000395455	pseudogene	80184993	80185192	+	80185024	31	+	
ENSG00000073905	VDAC1LP	ENST00000439229	pseudogene	80184999	80185198	+	80185024	25	+	

ENSEMBL55 GeneID	Gene Symbol	ENSEMBL55 TranscriptID	Biotype	Gene start	Gene end	Strand	Motif start	TSS offset	Motif strand	Description
ENSG00000199270	5S_rRNA	ENST00000362400	rRNA	228770537	2,29E+08	-	2,29E+08	43	-	5S ribosomal RNA [Source:RFAM;Acc:RF00001]
ENSG00000199276	5S_rRNA	ENST00000362406	rRNA	38428909	38429108	-	38429065	43	-	5S ribosomal RNA [Source:RFAM;Acc:RF00001]
ENSG00000199318	5S_rRNA	ENST00000362448	rRNA	87918857	87919056	-	87919013	43	-	5S ribosomal RNA [Source:RFAM;Acc:RF00001]
ENSG00000199334	5S_rRNA	ENST00000362464	rRNA	228768297	2,29E+08	-	2,29E+08	43	-	5S ribosomal RNA [Source:RFAM;Acc:RF00001]
ENSG00000199337	5S_rRNA	ENST00000362467	rRNA	228750416	2,29E+08	-	2,29E+08	43	-	5S ribosomal RNA [Source:RFAM;Acc:RF00001]
ENSG00000199345	5S_rRNA	ENST00000362475	rRNA	161207417	1,61E+08	-	1,61E+08	43	-	5S ribosomal RNA [Source:RFAM;Acc:RF00001]
ENSG00000199350	5S_rRNA	ENST00000362480	rRNA	84073439	84073638	-	84073598	40	-	5S ribosomal RNA [Source:RFAM;Acc:RF00001]
ENSG00000199352	5S_rRNA	ENST00000362482	rRNA	228745934	2,29E+08	-	2,29E+08	43	-	5S ribosomal RNA [Source:RFAM;Acc:RF00001]
ENSG00000199374	5S_rRNA	ENST00000362504	rRNA	10789500	10789699	-	10789663	36	-	5S ribosomal RNA [Source:RFAM;Acc:RF00001]
ENSG00000199395	5S_rRNA	ENST00000362525	rRNA	56462266	56462465	-	56462422	43	-	5S ribosomal RNA [Source:RFAM;Acc:RF00001]
ENSG00000199396	5S_rRNA	ENST00000362526	rRNA	228754898	2,29E+08	-	2,29E+08	43	-	5S ribosomal RNA [Source:RFAM;Acc:RF00001]
ENSG00000199404	5S_rRNA	ENST00000362534	rRNA	152289977	1,52E+08	-	1,52E+08	43	-	5S ribosomal RNA [Source:RFAM;Acc:RF00001]
ENSG00000199450	5S_rRNA	ENST00000362580	rRNA	117034805	1,17E+08	-	1,17E+08	32	-	5S ribosomal RNA [Source:RFAM;Acc:RF00001]
ENSG00000199454	5S_rRNA	ENST00000362584	rRNA	78644065	78644264	-	78644221	43	-	5S ribosomal RNA [Source:RFAM;Acc:RF00001]
ENSG00000199528	5S_rRNA	ENST00000362658	rRNA	103783174	1,04E+08	-	1,04E+08	43	-	5S ribosomal RNA [Source:RFAM;Acc:RF00001]
ENSG00000199625	5S_rRNA	ENST00000362755	rRNA	110074259	1,1E+08	-	1,1E+08	43	-	5S ribosomal RNA [Source:RFAM;Acc:RF00001]
ENSG00000199771	5S_rRNA	ENST00000362901	rRNA	50903287	50903486	-	50903442	44	-	5S ribosomal RNA [Source:RFAM;Acc:RF00001]
ENSG00000199786	5S_rRNA	ENST00000362916	rRNA	101466646	1,01E+08	-	1,01E+08	43	-	5S ribosomal RNA [Source:RFAM;Acc:RF00001]
ENSG00000199837	5S_rRNA	ENST00000362967	rRNA	182913426	1,83E+08	-	1,83E+08	43	-	5S ribosomal RNA [Source:RFAM;Acc:RF00001]
ENSG00000199839	5S_rRNA	ENST00000362969	rRNA	181540579	1,82E+08	-	1,82E+08	43	-	5S ribosomal RNA [Source:RFAM;Acc:RF00001]
ENSG00000199873	5S_rRNA	ENST00000363003	rRNA	37372316	37372515	-	37372474	41	-	5S ribosomal RNA [Source:RFAM;Acc:RF00001]
ENSG00000199910	5S_rRNA	ENST00000363040	rRNA	228766056	2,29E+08	-	2,29E+08	43	-	5S ribosomal RNA [Source:RFAM;Acc:RF00001]
ENSG00000199953	5S_rRNA	ENST00000363083	rRNA	43404664	43404863	-	43404820	43	-	5S ribosomal RNA [Source:RFAM;Acc:RF00001]
ENSG00000200021	5S_rRNA	ENST00000363151	rRNA	72413644	72413843	-	72413800	43	-	5S ribosomal RNA [Source:RFAM;Acc:RF00001]
ENSG00000200114	5S_rRNA	ENST00000363244	rRNA	12552513	12552712	-	12552669	43	-	5S ribosomal RNA [Source:RFAM;Acc:RF00001]
ENSG00000200115	5S_rRNA	ENST00000363245	rRNA	36887974	36888173	-	36888130	43	-	5S ribosomal RNA [Source:RFAM;Acc:RF00001]
ENSG00000200159	5S_rRNA	ENST00000363289	rRNA	92270118	92270317	-	92270274	43	-	5S ribosomal RNA [Source:RFAM;Acc:RF00001]
ENSG00000200168	5S_rRNA	ENST00000363298	rRNA	110910733	1,11E+08	-	1,11E+08	43	-	5S ribosomal RNA [Source:RFAM;Acc:RF00001]
ENSG00000200293	5S_rRNA	ENST00000363423	rRNA	42144028	42144227	-	42144184	43	-	5S ribosomal RNA [Source:RFAM;Acc:RF00001]
ENSG00000200326	5S_rRNA	ENST00000363456	rRNA	27525384	27525583	-	27525540	43	-	5S ribosomal RNA [Source:RFAM;Acc:RF00001]
ENSG00000200327	5S_rRNA	ENST00000363457	rRNA	163438196	1,63E+08	-	1,63E+08	43	-	5S ribosomal RNA [Source:RFAM;Acc:RF00001]
ENSG00000200336	5S_rRNA	ENST00000363466	rRNA	18269854	18270053	-	18270010	43	-	5S ribosomal RNA [Source:RFAM;Acc:RF00001]
ENSG00000200343	5S_rRNA	ENST00000363473	rRNA	228761575	2,29E+08	-	2,29E+08	43	-	5S ribosomal RNA [Source:RFAM;Acc:RF00001]
ENSG00000200370	5S_rRNA	ENST00000363500	rRNA	228781706	2,29E+08	-	2,29E+08	43	-	5S ribosomal RNA [Source:RFAM;Acc:RF00001]

ENSEMBL55 GeneID	Gene Symbol	ENSEMBL55 TranscriptID	Biotype	Gene start	Gene end	Strand	Motif start	TSS offset	Motif strand	Description
ENSG00000200381	5S_rRNA	ENST00000363511	rRNA	228752657	2,29E+08	-	2,29E+08	43	-	5S ribosomal RNA [Source:RFAM;Acc:RF00001]
ENSG00000200411	5S_rRNA	ENST00000363541	rRNA	96207657	96207856	-	96207813	43	-	5S ribosomal RNA [Source:RFAM;Acc:RF00001]
ENSG00000200468	5S_rRNA	ENST00000363598	rRNA	9658155	9658354	-	9658311	43	-	5S ribosomal RNA [Source:RFAM;Acc:RF00001]
ENSG00000200624	5S_rRNA	ENST00000363754	rRNA	228757113	2,29E+08	-	2,29E+08	43	-	5S ribosomal RNA [Source:RFAM;Acc:RF00001]
ENSG00000200649	5S_rRNA	ENST00000363779	rRNA	73909699	73909898	-	73909856	42	-	5S ribosomal RNA [Source:RFAM;Acc:RF00001]
ENSG00000200650	5S_rRNA	ENST00000363780	rRNA	189141372	1,89E+08	-	1,89E+08	43	-	5S ribosomal RNA [Source:RFAM;Acc:RF00001]
ENSG00000200687	5S_rRNA	ENST00000363817	rRNA	19423330	19423529	-	19423485	44	-	5S ribosomal RNA [Source:RFAM;Acc:RF00001]
ENSG00000200709	5S_rRNA	ENST00000363839	rRNA	46125539	46125738	-	46125695	43	-	5S ribosomal RNA [Source:RFAM;Acc:RF00001]
ENSG00000200719	5S_rRNA	ENST00000363849	rRNA	28905922	28906121	-	28906078	43	-	5S ribosomal RNA [Source:RFAM;Acc:RF00001]
ENSG00000200820	5S_rRNA	ENST00000363950	rRNA	93179576	93179775	-	93179735	40	-	5S ribosomal RNA [Source:RFAM;Acc:RF00001]
ENSG00000200880	5S_rRNA	ENST00000364010	rRNA	76137989	76138188	-	76138145	43	-	5S ribosomal RNA [Source:RFAM;Acc:RF00001]
ENSG00000200890	5S_rRNA	ENST00000364020	rRNA	81723252	81723451	-	81723408	43	-	5S ribosomal RNA [Source:RFAM;Acc:RF00001]
ENSG00000200914	5S_rRNA	ENST00000364044	rRNA	6504172	6504371	-	6504328	43	-	5S ribosomal RNA [Source:RFAM;Acc:RF00001]
ENSG00000201014	5S_rRNA	ENST00000364144	rRNA	71378642	71378841	-	71378798	43	-	5S ribosomal RNA [Source:RFAM;Acc:RF00001]
ENSG00000201046	5S_rRNA	ENST00000364176	rRNA	63690928	63691127	-	63691084	43	-	5S ribosomal RNA [Source:RFAM;Acc:RF00001]
ENSG00000201149	5S_rRNA	ENST00000364279	rRNA	95257099	95257298	-	95257255	43	-	5S ribosomal RNA [Source:RFAM;Acc:RF00001]
ENSG00000201285	5S_rRNA	ENST00000364415	rRNA	147089536	1,47E+08	-	1,47E+08	43	-	5S ribosomal RNA [Source:RFAM;Acc:RF00001]
ENSG00000201312	5S_rRNA	ENST00000364442	rRNA	184984000	1,85E+08	-	1,85E+08	42	-	5S ribosomal RNA [Source:RFAM;Acc:RF00001]
ENSG00000201321	5S_rRNA	ENST00000364451	rRNA	228763814	2,29E+08	-	2,29E+08	43	-	5S ribosomal RNA [Source:RFAM;Acc:RF00001]
ENSG00000201325	5S_rRNA	ENST00000364455	rRNA	137236898	1,37E+08	-	1,37E+08	43	-	5S ribosomal RNA [Source:RFAM;Acc:RF00001]
ENSG00000201355	5S_rRNA	ENST00000364485	rRNA	228775003	2,29E+08	-	2,29E+08	43	-	5S ribosomal RNA [Source:RFAM;Acc:RF00001]
ENSG00000201361	5S_rRNA	ENST00000364491	rRNA	84296335	84296534	-	84296491	43	-	5S ribosomal RNA [Source:RFAM;Acc:RF00001]
ENSG00000201369	5S_rRNA	ENST00000364499	rRNA	54480656	54480855	-	54480812	43	-	5S ribosomal RNA [Source:RFAM;Acc:RF00001]
ENSG00000201394	5S_rRNA	ENST00000364524	rRNA	133428812	1,33E+08	-	1,33E+08	35	-	5S ribosomal RNA [Source:RFAM;Acc:RF00001]
ENSG00000201413	5S_rRNA	ENST00000364543	rRNA	134502198	1,35E+08	-	1,35E+08	43	-	5S ribosomal RNA [Source:RFAM;Acc:RF00001]
ENSG00000201415	5S_rRNA	ENST00000364545	rRNA	17721935	17722134	-	17722092	42	-	5S ribosomal RNA [Source:RFAM;Acc:RF00001]
ENSG00000201420	5S_rRNA	ENST00000364550	rRNA	110912977	1,11E+08	-	1,11E+08	43	-	5S ribosomal RNA [Source:RFAM;Acc:RF00001]
ENSG00000201440	5S_rRNA	ENST00000364570	rRNA	134562011	1,35E+08	-	1,35E+08	43	-	5S ribosomal RNA [Source:RFAM;Acc:RF00001]
ENSG00000201515	5S_rRNA	ENST00000364645	rRNA	66015482	66015681	-	66015638	43	-	5S ribosomal RNA [Source:RFAM;Acc:RF00001]
ENSG00000201518	5S_rRNA	ENST00000364648	rRNA	128570425	1,29E+08	-	1,29E+08	43	-	5S ribosomal RNA [Source:RFAM;Acc:RF00001]
ENSG00000201527	5S_rRNA	ENST00000364657	rRNA	23141409	23141608	-	23141565	43	-	5S ribosomal RNA [Source:RFAM;Acc:RF00001]
ENSG00000201588	5S_rRNA	ENST00000364718	rRNA	228748175	2,29E+08	-	2,29E+08	43	-	5S ribosomal RNA [Source:RFAM;Acc:RF00001]
ENSG00000201594	5S_rRNA	ENST00000364724	rRNA	145508891	1,46E+08	-	1,46E+08	43	-	5S ribosomal RNA [Source:RFAM;Acc:RF00001]
ENSG00000201595	5S_rRNA	ENST00000364725	rRNA	51728399	51728598	-	51728555	43	-	5S ribosomal RNA [Source:RFAM;Acc:RF00001]

ENSEMBL55 GeneID	Gene Symbol	ENSEMBL55 TranscriptID	Biotype	Gene start	Gene end	Strand	Motif start	TSS offset	Motif strand	Description
ENSG00000201620	5S_rRNA	ENST00000364750	rRNA	86349282	86349481	-	86349438	43	-	5S ribosomal RNA [Source:RFAM;Acc:RF00001]
ENSG00000201728	5S_rRNA	ENST00000364858	rRNA	23361450	23361649	-	23361606	43	-	5S ribosomal RNA [Source:RFAM;Acc:RF00001]
ENSG00000201766	5S_rRNA	ENST00000364896	rRNA	14705285	14705484	-	14705450	34	-	5S ribosomal RNA [Source:RFAM;Acc:RF00001]
ENSG00000201790	5S_rRNA	ENST00000364920	rRNA	105258610	1,05E+08	-	1,05E+08	37	-	5S ribosomal RNA [Source:RFAM;Acc:RF00001]
ENSG00000201822	5S_rRNA	ENST00000364952	rRNA	179879566	1,8E+08	-	1,8E+08	43	-	5S ribosomal RNA [Source:RFAM;Acc:RF00001]
ENSG00000201856	5S_rRNA	ENST00000364986	rRNA	13928938	13929137	-	13929094	43	-	5S ribosomal RNA [Source:RFAM;Acc:RF00001]
ENSG00000201861	5S_rRNA	ENST00000364991	rRNA	327862	328061	-	328022	39	-	5S ribosomal RNA [Source:RFAM;Acc:RF00001]
ENSG00000201876	5S_rRNA	ENST00000365006	rRNA	74195125	74195324	-	74195290	34	-	5S ribosomal RNA [Source:RFAM;Acc:RF00001]
ENSG00000201923	5S_rRNA	ENST00000365053	rRNA	44188316	44188515	-	44188472	43	-	5S ribosomal RNA [Source:RFAM;Acc:RF00001]
ENSG00000201925	5S_rRNA	ENST00000365055	rRNA	228777234	2,29E+08	-	2,29E+08	43	-	5S ribosomal RNA [Source:RFAM;Acc:RF00001]
ENSG00000201942	5S_rRNA	ENST00000365072	rRNA	80272000	80272199	-	80272159	40	-	5S ribosomal RNA [Source:RFAM;Acc:RF00001]
ENSG00000201962	5S_rRNA	ENST00000365092	rRNA	25393684	25393883	-	25393840	43	-	5S ribosomal RNA [Source:RFAM;Acc:RF00001]
ENSG00000201991	5S_rRNA	ENST00000365121	rRNA	61068438	61068637	-	61068594	43	-	5S ribosomal RNA [Source:RFAM;Acc:RF00001]
ENSG00000202110	5S_rRNA	ENST00000365240	rRNA	22069820	22070019	-	22069976	43	-	5S ribosomal RNA [Source:RFAM;Acc:RF00001]
ENSG00000202175	5S_rRNA	ENST00000365305	rRNA	33533391	33533590	-	33533547	43	-	5S ribosomal RNA [Source:RFAM;Acc:RF00001]
ENSG00000202225	5S_rRNA	ENST00000365355	rRNA	120621408	1,21E+08	-	1,21E+08	43	-	5S ribosomal RNA [Source:RFAM;Acc:RF00001]
ENSG00000202248	5S_rRNA	ENST00000365378	rRNA	11701706	11701905	-	11701862	43	-	5S ribosomal RNA [Source:RFAM;Acc:RF00001]
ENSG00000202257	5S_rRNA	ENST00000365387	rRNA	228779475	2,29E+08	-	2,29E+08	43	-	5S ribosomal RNA [Source:RFAM;Acc:RF00001]
ENSG00000202263	5S_rRNA	ENST00000365393	rRNA	78560403	78560602	-	78560566	36	-	5S ribosomal RNA [Source:RFAM;Acc:RF00001]
ENSG00000202290	5S_rRNA	ENST00000365420	rRNA	98015156	98015355	-	98015312	43	-	5S ribosomal RNA [Source:RFAM;Acc:RF00001]
ENSG00000202324	5S_rRNA	ENST00000365454	rRNA	99477484	99477683	-	99477640	43	-	5S ribosomal RNA [Source:RFAM;Acc:RF00001]
ENSG00000202334	5S_rRNA	ENST00000365464	rRNA	114253762	1,14E+08	-	1,14E+08	43	-	5S ribosomal RNA [Source:RFAM;Acc:RF00001]
ENSG00000202356	5S_rRNA	ENST00000365486	rRNA	120903270	1,21E+08	-	1,21E+08	43	-	5S ribosomal RNA [Source:RFAM;Acc:RF00001]
ENSG00000202502	5S_rRNA	ENST00000365632	rRNA	182976005	1,83E+08	-	1,83E+08	43	-	5S ribosomal RNA [Source:RFAM;Acc:RF00001]
ENSG00000202521	5S_rRNA	ENST00000365651	rRNA	228759333	2,29E+08	-	2,29E+08	43	-	5S ribosomal RNA [Source:RFAM;Acc:RF00001]
ENSG00000202526	5S_rRNA	ENST00000365656	rRNA	228772762	2,29E+08	-	2,29E+08	43	-	5S ribosomal RNA [Source:RFAM;Acc:RF00001]
ENSG00000206882	5S_rRNA	ENST00000384155	rRNA	22561676	22561875	-	22561832	43	-	5S ribosomal RNA [Source:RFAM;Acc:RF00001]
ENSG00000212171	5S_rRNA	ENST00000390869	rRNA	20601280	20601479	-	20601442	37	-	5S ribosomal RNA [Source:RFAM;Acc:RF00001]
ENSG00000212276	5S_rRNA	ENST00000390974	rRNA	109605948	1,1E+08	-	1,1E+08	43	-	5S ribosomal RNA [Source:RFAM;Acc:RF00001]
ENSG00000212290	5S_rRNA	ENST00000390988	rRNA	47484173	47484372	-	47484329	43	-	5S ribosomal RNA [Source:RFAM;Acc:RF00001]
ENSG00000212320	5S_rRNA	ENST00000391018	rRNA	129814435	1,3E+08	-	1,3E+08	43	-	5S ribosomal RNA [Source:RFAM;Acc:RF00001]
ENSG00000212331	5S_rRNA	ENST00000391029	rRNA	200684	200883	-	200840	43	-	5S ribosomal RNA [Source:RFAM;Acc:RF00001]
ENSG00000212333	5S_rRNA	ENST00000391031	rRNA	114541751	1,15E+08	-	1,15E+08	43	-	5S ribosomal RNA [Source:RFAM;Acc:RF00001]
ENSG00000212396	5S_rRNA	ENST00000391094	rRNA	95270238	95270437	-	95270394	43	-	5S ribosomal RNA [Source:RFAM;Acc:RF00001]

ENSEMBL55 GeneID	Gene Symbol	ENSEMBL55 TranscriptID	Biotype	Gene start	Gene end	Strand	Motif start	TSS offset	Motif strand	Description
ENSG00000212425	5S_rRNA	ENST00000391123	rRNA	138269581	1,38E+08	-	1,38E+08	39	-	5S ribosomal RNA [Source:RFAM;Acc:RF00001]
ENSG00000212439	5S_rRNA	ENST00000391137	rRNA	222869762	2,23E+08	-	2,23E+08	34	-	5S ribosomal RNA [Source:RFAM;Acc:RF00001]
ENSG00000212499	5S_rRNA	ENST00000391197	rRNA	12966583	12966782	-	12966744	38	-	5S ribosomal RNA [Source:RFAM;Acc:RF00001]
ENSG00000212549	5S_rRNA	ENST00000391247	rRNA	26526467	26526666	-	26526623	43	-	5S ribosomal RNA [Source:RFAM;Acc:RF00001]
ENSG00000212559	5S_rRNA	ENST00000391257	rRNA	94677649	94677848	-	94677809	39	-	5S ribosomal RNA [Source:RFAM;Acc:RF00001]
ENSG00000212595	5S_rRNA	ENST00000391293	rRNA	150194679	1,5E+08	-	1,5E+08	43	-	5S ribosomal RNA [Source:RFAM;Acc:RF00001]
ENSG00000212601	5S_rRNA	ENST00000391299	rRNA	93953810	93954009	-	93953965	44	-	5S ribosomal RNA [Source:RFAM;Acc:RF00001]
ENSG00000222063	5S_rRNA	ENST00000410131	rRNA	84564972	84565171	-	84565128	43	-	5S ribosomal RNA [Source:RFAM;Acc:RF00001]
ENSG00000222118	5S_rRNA	ENST00000410186	rRNA	54780030	54780229	-	54780185	44	-	5S ribosomal RNA [Source:RFAM;Acc:RF00001]
ENSG00000222199	5S_rRNA	ENST00000410267	rRNA	162291008	1,62E+08	-	1,62E+08	43	-	5S ribosomal RNA [Source:RFAM;Acc:RF00001]
ENSG00000222205	5S_rRNA	ENST00000410273	rRNA	58039122	58039321	-	58039278	43	-	5S ribosomal RNA [Source:RFAM;Acc:RF00001]
ENSG00000222236	5S_rRNA	ENST00000410304	rRNA	72625134	72625333	-	72625290	43	-	5S ribosomal RNA [Source:RFAM;Acc:RF00001]
ENSG00000222285	5S_rRNA	ENST00000410353	rRNA	136304796	1,36E+08	-	1,36E+08	43	-	5S ribosomal RNA [Source:RFAM;Acc:RF00001]
ENSG00000222308	5S_rRNA	ENST00000410376	rRNA	151255053	1,51E+08	-	1,51E+08	42	-	5S ribosomal RNA [Source:RFAM;Acc:RF00001]
ENSG00000222346	5S_rRNA	ENST00000410414	rRNA	111593621	1,12E+08	-	1,12E+08	43	-	5S ribosomal RNA [Source:RFAM;Acc:RF00001]
ENSG00000222383	5S_rRNA	ENST00000410451	rRNA	10752960	10753159	-	10753116	43	-	5S ribosomal RNA [Source:RFAM;Acc:RF00001]
ENSG00000222419	5S_rRNA	ENST00000410487	rRNA	103105642	1,03E+08	-	1,03E+08	38	-	5S ribosomal RNA [Source:RFAM;Acc:RF00001]
ENSG00000222419	5S_rRNA	ENST00000410487	rRNA	103105642	1,03E+08	-	1,03E+08	43	-	5S ribosomal RNA [Source:RFAM;Acc:RF00001]
ENSG00000222428	5S_rRNA	ENST00000410496	rRNA	68188835	68189034	-	68188992	42	-	5S ribosomal RNA [Source:RFAM;Acc:RF00001]
ENSG00000222455	5S_rRNA	ENST00000410523	rRNA	130603300	1,31E+08	-	1,31E+08	38	-	5S ribosomal RNA [Source:RFAM;Acc:RF00001]
ENSG00000222520	5S_rRNA	ENST00000410588	rRNA	19508856	19509055	-	19509012	43	-	5S ribosomal RNA [Source:RFAM;Acc:RF00001]
ENSG00000222682	5S_rRNA	ENST00000410750	rRNA	106807640	1,07E+08	-	1,07E+08	43	-	5S ribosomal RNA [Source:RFAM;Acc:RF00001]
ENSG00000222740	5S_rRNA	ENST00000410808	rRNA	127833952	1,28E+08	-	1,28E+08	43	-	5S ribosomal RNA [Source:RFAM;Acc:RF00001]
ENSG00000222778	5S_rRNA	ENST00000410846	rRNA	143905663	1,44E+08	-	1,44E+08	43	-	5S ribosomal RNA [Source:RFAM;Acc:RF00001]
ENSG00000222789	5S_rRNA	ENST00000410857	rRNA	36633176	36633375	-	36633332	43	-	5S ribosomal RNA [Source:RFAM;Acc:RF00001]
ENSG00000222838	5S_rRNA	ENST00000410906	rRNA	72740763	72740962	-	72740919	43	-	5S ribosomal RNA [Source:RFAM;Acc:RF00001]
ENSG00000222854	5S_rRNA	ENST00000410922	rRNA	148913183	1,49E+08	-	1,49E+08	42	-	5S ribosomal RNA [Source:RFAM;Acc:RF00001]
ENSG00000223016	5S_rRNA	ENST00000411084	rRNA	49159937	49160136	-	49160093	43	-	5S ribosomal RNA [Source:RFAM;Acc:RF00001]
ENSG00000223086	5S_rRNA	ENST00000411154	rRNA	10117309	10117508	-	10117465	43	-	5S ribosomal RNA [Source:RFAM;Acc:RF00001]
ENSG00000223169	5S_rRNA	ENST00000411237	rRNA	76574853	76575052	-	76575009	43	-	5S ribosomal RNA [Source:RFAM;Acc:RF00001]
ENSG00000223238	5S_rRNA	ENST00000411306	rRNA	114173823	1,14E+08	-	1,14E+08	43	-	5S ribosomal RNA [Source:RFAM;Acc:RF00001]
ENSG00000223259	5S_rRNA	ENST00000411327	rRNA	75875329	75875528	-	75875485	43	-	5S ribosomal RNA [Source:RFAM;Acc:RF00001]
ENSG00000223304	5S_rRNA	ENST00000411372	rRNA	24253510	24253709	-	24253675	34	-	5S ribosomal RNA [Source:RFAM;Acc:RF00001]
ENSG00000223318	5S_rRNA	ENST00000411386	rRNA	165752088	1,66E+08	-	1,66E+08	43	-	5S ribosomal RNA [Source:RFAM;Acc:RF00001]

ENSEMBL55 GeneID	Gene Symbol	ENSEMBL55 TranscriptID	Biotype	Gene start	Gene end	Strand	Motif start	TSS offset	Motif strand	Description
ENSG00000238379	5S_rRNA	ENST00000459503	rRNA	129202573	1,29E+08	-	1,29E+08	42	-	5S ribosomal RNA [Source:RFAM;Acc:RF00001]
ENSG00000238391	5S_rRNA	ENST00000459153	rRNA	73901671	73901870	-	73901827	43	-	5S ribosomal RNA [Source:RFAM;Acc:RF00001]
ENSG00000238967	5S_rRNA	ENST00000384062	rRNA	74011019	74011218	-	74011175	43	-	5S ribosomal RNA [Source:RFAM;Acc:RF00001]
ENSG00000199270	5S_rRNA	ENST00000362400	rRNA	228770537	2,29E+08	-	2,29E+08	42	+	5S ribosomal RNA [Source:RFAM;Acc:RF00001]
ENSG00000199334	5S_rRNA	ENST00000362464	rRNA	228768297	2,29E+08	-	2,29E+08	42	+	5S ribosomal RNA [Source:RFAM;Acc:RF00001]
ENSG00000199337	5S_rRNA	ENST00000362467	rRNA	228750416	2,29E+08	-	2,29E+08	42	+	5S ribosomal RNA [Source:RFAM;Acc:RF00001]
ENSG00000199345	5S_rRNA	ENST00000362475	rRNA	161207417	1,61E+08	-	1,61E+08	42	+	5S ribosomal RNA [Source:RFAM;Acc:RF00001]
ENSG00000199350	5S_rRNA	ENST00000362480	rRNA	84073439	84073638	-	84073599	39	+	5S ribosomal RNA [Source:RFAM;Acc:RF00001]
ENSG00000199352	5S_rRNA	ENST00000362482	rRNA	228745934	2,29E+08	-	2,29E+08	42	+	5S ribosomal RNA [Source:RFAM;Acc:RF00001]
ENSG00000199374	5S_rRNA	ENST00000362504	rRNA	10789500	10789699	-	10789664	35	+	5S ribosomal RNA [Source:RFAM;Acc:RF00001]
ENSG00000199395	5S_rRNA	ENST00000362525	rRNA	56462266	56462465	-	56462423	42	+	5S ribosomal RNA [Source:RFAM;Acc:RF00001]
ENSG00000199396	5S_rRNA	ENST00000362526	rRNA	228754898	2,29E+08	-	2,29E+08	42	+	5S ribosomal RNA [Source:RFAM;Acc:RF00001]
ENSG00000199404	5S_rRNA	ENST00000362534	rRNA	152289977	1,52E+08	-	1,52E+08	42	+	5S ribosomal RNA [Source:RFAM;Acc:RF00001]
ENSG00000199454	5S_rRNA	ENST00000362584	rRNA	78644065	78644264	-	78644222	42	+	5S ribosomal RNA [Source:RFAM;Acc:RF00001]
ENSG00000199625	5S_rRNA	ENST00000362755	rRNA	110074259	1,1E+08	-	1,1E+08	42	+	5S ribosomal RNA [Source:RFAM;Acc:RF00001]
ENSG00000199786	5S_rRNA	ENST00000362916	rRNA	101466646	1,01E+08	-	1,01E+08	42	+	5S ribosomal RNA [Source:RFAM;Acc:RF00001]
ENSG00000199839	5S_rRNA	ENST00000362969	rRNA	181540579	1,82E+08	-	1,82E+08	42	+	5S ribosomal RNA [Source:RFAM;Acc:RF00001]
ENSG00000199873	5S_rRNA	ENST00000363003	rRNA	37372316	37372515	-	37372475	40	+	5S ribosomal RNA [Source:RFAM;Acc:RF00001]
ENSG00000199910	5S_rRNA	ENST00000363040	rRNA	228766056	2,29E+08	-	2,29E+08	42	+	5S ribosomal RNA [Source:RFAM;Acc:RF00001]
ENSG00000199953	5S_rRNA	ENST00000363083	rRNA	43404664	43404863	-	43404821	42	+	5S ribosomal RNA [Source:RFAM;Acc:RF00001]
ENSG00000200114	5S_rRNA	ENST00000363244	rRNA	12552513	12552712	-	12552670	42	+	5S ribosomal RNA [Source:RFAM;Acc:RF00001]
ENSG00000200159	5S_rRNA	ENST00000363289	rRNA	92270118	92270317	-	92270275	42	+	5S ribosomal RNA [Source:RFAM;Acc:RF00001]
ENSG00000200168	5S_rRNA	ENST00000363298	rRNA	110910733	1,11E+08	-	1,11E+08	42	+	5S ribosomal RNA [Source:RFAM;Acc:RF00001]
ENSG00000200293	5S_rRNA	ENST00000363423	rRNA	42144028	42144227	-	42144185	42	+	5S ribosomal RNA [Source:RFAM;Acc:RF00001]
ENSG00000200327	5S_rRNA	ENST00000363457	rRNA	163438196	1,63E+08	-	1,63E+08	42	+	5S ribosomal RNA [Source:RFAM;Acc:RF00001]
ENSG00000200343	5S_rRNA	ENST00000363473	rRNA	228761575	2,29E+08	-	2,29E+08	42	+	5S ribosomal RNA [Source:RFAM;Acc:RF00001]
ENSG00000200370	5S_rRNA	ENST00000363500	rRNA	228781706	2,29E+08	-	2,29E+08	42	+	5S ribosomal RNA [Source:RFAM;Acc:RF00001]
ENSG00000200381	5S_rRNA	ENST00000363511	rRNA	228752657	2,29E+08	-	2,29E+08	42	+	5S ribosomal RNA [Source:RFAM;Acc:RF00001]
ENSG00000200411	5S_rRNA	ENST00000363541	rRNA	96207657	96207856	-	96207814	42	+	5S ribosomal RNA [Source:RFAM;Acc:RF00001]
ENSG00000200468	5S_rRNA	ENST00000363598	rRNA	9658155	9658354	-	9658312	42	+	5S ribosomal RNA [Source:RFAM;Acc:RF00001]
ENSG00000200601	5S_rRNA	ENST00000363731	rRNA	74215110	74215309	-	74215266	43	+	5S ribosomal RNA [Source:RFAM;Acc:RF00001]
ENSG00000200624	5S_rRNA	ENST00000363754	rRNA	228757113	2,29E+08	-	2,29E+08	42	+	5S ribosomal RNA [Source:RFAM;Acc:RF00001]
ENSG00000200649	5S_rRNA	ENST00000363779	rRNA	73909699	73909898	-	73909857	41	+	5S ribosomal RNA [Source:RFAM;Acc:RF00001]
ENSG00000200650	5S_rRNA	ENST00000363780	rRNA	189141372	1,89E+08	-	1,89E+08	42	+	5S ribosomal RNA [Source:RFAM;Acc:RF00001]

ENSEMBL55 GeneID	Gene Symbol	ENSEMBL55 TranscriptID	Biotype	Gene start	Gene end	Strand	Motif start	TSS offset	Motif strand	Description
ENSG00000200687	5S_rRNA	ENST00000363817	rRNA	19423330	19423529	-	19423486	43	+	5S ribosomal RNA [Source:RFAM;Acc:RF00001]
ENSG00000200719	5S_rRNA	ENST00000363849	rRNA	28905922	28906121	-	28906079	42	+	5S ribosomal RNA [Source:RFAM;Acc:RF00001]
ENSG00000200820	5S_rRNA	ENST00000363950	rRNA	93179576	93179775	-	93179736	39	+	5S ribosomal RNA [Source:RFAM;Acc:RF00001]
ENSG00000200854	5S_rRNA	ENST00000363984	rRNA	77870138	77870337	-	77870295	42	+	5S ribosomal RNA [Source:RFAM;Acc:RF00001]
ENSG00000200914	5S_rRNA	ENST00000364044	rRNA	6504172	6504371	-	6504329	42	+	5S ribosomal RNA [Source:RFAM;Acc:RF00001]
ENSG00000201014	5S_rRNA	ENST00000364144	rRNA	71378642	71378841	-	71378799	42	+	5S ribosomal RNA [Source:RFAM;Acc:RF00001]
ENSG00000201046	5S_rRNA	ENST00000364176	rRNA	63690928	63691127	-	63691085	42	+	5S ribosomal RNA [Source:RFAM;Acc:RF00001]
ENSG00000201149	5S_rRNA	ENST00000364279	rRNA	95257099	95257298	-	95257256	42	+	5S ribosomal RNA [Source:RFAM;Acc:RF00001]
ENSG00000201321	5S_rRNA	ENST00000364451	rRNA	228763814	2,29E+08	-	2,29E+08	42	+	5S ribosomal RNA [Source:RFAM;Acc:RF00001]
ENSG00000201325	5S_rRNA	ENST00000364455	rRNA	137236898	1,37E+08	-	1,37E+08	42	+	5S ribosomal RNA [Source:RFAM;Acc:RF00001]
ENSG00000201355	5S_rRNA	ENST00000364485	rRNA	228775003	2,29E+08	-	2,29E+08	42	+	5S ribosomal RNA [Source:RFAM;Acc:RF00001]
ENSG00000201394	5S_rRNA	ENST00000364524	rRNA	133428812	1,33E+08	-	1,33E+08	34	+	5S ribosomal RNA [Source:RFAM;Acc:RF00001]
ENSG00000201413	5S_rRNA	ENST00000364543	rRNA	134502198	1,35E+08	-	1,35E+08	42	+	5S ribosomal RNA [Source:RFAM;Acc:RF00001]
ENSG00000201415	5S_rRNA	ENST00000364545	rRNA	17721935	17722134	-	17722093	41	+	5S ribosomal RNA [Source:RFAM;Acc:RF00001]
ENSG00000201420	5S_rRNA	ENST00000364550	rRNA	110912977	1,11E+08	-	1,11E+08	42	+	5S ribosomal RNA [Source:RFAM;Acc:RF00001]
ENSG00000201469	5S_rRNA	ENST00000364599	rRNA	133300444	1,33E+08	-	1,33E+08	51	+	5S ribosomal RNA [Source:RFAM;Acc:RF00001]
ENSG00000201515	5S_rRNA	ENST00000364645	rRNA	66015482	66015681	-	66015639	42	+	5S ribosomal RNA [Source:RFAM;Acc:RF00001]
ENSG00000201588	5S_rRNA	ENST00000364718	rRNA	228748175	2,29E+08	-	2,29E+08	42	+	5S ribosomal RNA [Source:RFAM;Acc:RF00001]
ENSG00000201595	5S_rRNA	ENST00000364725	rRNA	51728399	51728598	-	51728556	42	+	5S ribosomal RNA [Source:RFAM;Acc:RF00001]
ENSG00000201790	5S_rRNA	ENST00000364920	rRNA	105258610	1,05E+08	-	1,05E+08	36	+	5S ribosomal RNA [Source:RFAM;Acc:RF00001]
ENSG00000201822	5S_rRNA	ENST00000364952	rRNA	179879566	1,8E+08	-	1,8E+08	42	+	5S ribosomal RNA [Source:RFAM;Acc:RF00001]
ENSG00000201876	5S_rRNA	ENST00000365006	rRNA	74195125	74195324	-	74195291	33	+	5S ribosomal RNA [Source:RFAM;Acc:RF00001]
ENSG00000201923	5S_rRNA	ENST00000365053	rRNA	44188316	44188515	-	44188473	42	+	5S ribosomal RNA [Source:RFAM;Acc:RF00001]
ENSG00000201925	5S_rRNA	ENST00000365055	rRNA	228777234	2,29E+08	-	2,29E+08	42	+	5S ribosomal RNA [Source:RFAM;Acc:RF00001]
ENSG00000202110	5S_rRNA	ENST00000365240	rRNA	22069820	22070019	-	22069977	42	+	5S ribosomal RNA [Source:RFAM;Acc:RF00001]
ENSG00000202225	5S_rRNA	ENST00000365355	rRNA	120621408	1,21E+08	-	1,21E+08	42	+	5S ribosomal RNA [Source:RFAM;Acc:RF00001]
ENSG00000202248	5S_rRNA	ENST00000365378	rRNA	11701706	11701905	-	11701863	42	+	5S ribosomal RNA [Source:RFAM;Acc:RF00001]
ENSG00000202257	5S_rRNA	ENST00000365387	rRNA	228779475	2,29E+08	-	2,29E+08	42	+	5S ribosomal RNA [Source:RFAM;Acc:RF00001]
ENSG00000202290	5S_rRNA	ENST00000365420	rRNA	98015156	98015355	-	98015313	42	+	5S ribosomal RNA [Source:RFAM;Acc:RF00001]
ENSG00000202324	5S_rRNA	ENST00000365454	rRNA	99477484	99477683	-	99477641	42	+	5S ribosomal RNA [Source:RFAM;Acc:RF00001]
ENSG00000202356	5S_rRNA	ENST00000365486	rRNA	120903270	1,21E+08	-	1,21E+08	42	+	5S ribosomal RNA [Source:RFAM;Acc:RF00001]
ENSG00000202502	5S_rRNA	ENST00000365632	rRNA	182976005	1,83E+08	-	1,83E+08	42	+	5S ribosomal RNA [Source:RFAM;Acc:RF00001]
ENSG00000202521	5S_rRNA	ENST00000365651	rRNA	228759333	2,29E+08	-	2,29E+08	42	+	5S ribosomal RNA [Source:RFAM;Acc:RF00001]
ENSG00000202526	5S_rRNA	ENST00000365656	rRNA	228772762	2,29E+08	-	2,29E+08	42	+	5S ribosomal RNA [Source:RFAM;Acc:RF00001]

ENSEMBL55 GeneID	Gene Symbol	ENSEMBL55 TranscriptID	Biotype	Gene start	Gene end	Strand	Motif start	TSS offset	Motif strand	Description
ENSG00000206882	5S_rRNA	ENST00000384155	rRNA	22561676	22561875	-	22561833	42	+	5S ribosomal RNA [Source:RFAM;Acc:RF00001]
ENSG00000212171	5S_rRNA	ENST00000390869	rRNA	20601280	20601479	-	20601443	36	+	5S ribosomal RNA [Source:RFAM;Acc:RF00001]
ENSG00000212276	5S_rRNA	ENST00000390974	rRNA	109605948	1,1E+08	-	1,1E+08	42	+	5S ribosomal RNA [Source:RFAM;Acc:RF00001]
ENSG00000212320	5S_rRNA	ENST00000391018	rRNA	129814435	1,3E+08	-	1,3E+08	42	+	5S ribosomal RNA [Source:RFAM;Acc:RF00001]
ENSG00000212331	5S_rRNA	ENST00000391029	rRNA	200684	200883	-	200841	42	+	5S ribosomal RNA [Source:RFAM;Acc:RF00001]
ENSG00000212396	5S_rRNA	ENST00000391094	rRNA	95270238	95270437	-	95270395	42	+	5S ribosomal RNA [Source:RFAM;Acc:RF00001]
ENSG00000212499	5S_rRNA	ENST00000391197	rRNA	12966583	12966782	-	12966745	37	+	5S ribosomal RNA [Source:RFAM;Acc:RF00001]
ENSG00000212549	5S_rRNA	ENST00000391247	rRNA	26526467	26526666	-	26526624	42	+	5S ribosomal RNA [Source:RFAM;Acc:RF00001]
ENSG00000212595	5S_rRNA	ENST00000391293	rRNA	150194679	1,5E+08	-	1,5E+08	42	+	5S ribosomal RNA [Source:RFAM;Acc:RF00001]
ENSG00000212601	5S_rRNA	ENST00000391299	rRNA	93953810	93954009	-	93953966	43	+	5S ribosomal RNA [Source:RFAM;Acc:RF00001]
ENSG00000222063	5S_rRNA	ENST00000410131	rRNA	84564972	84565171	-	84565129	42	+	5S ribosomal RNA [Source:RFAM;Acc:RF00001]
ENSG00000222118	5S_rRNA	ENST00000410186	rRNA	54780030	54780229	-	54780186	43	+	5S ribosomal RNA [Source:RFAM;Acc:RF00001]
ENSG00000222199	5S_rRNA	ENST00000410267	rRNA	162291008	1,62E+08	-	1,62E+08	42	+	5S ribosomal RNA [Source:RFAM;Acc:RF00001]
ENSG00000222205	5S_rRNA	ENST00000410273	rRNA	58039122	58039321	-	58039279	42	+	5S ribosomal RNA [Source:RFAM;Acc:RF00001]
ENSG00000222317	5S_rRNA	ENST00000410385	rRNA	210586546	2,11E+08	-	2,11E+08	71	+	5S ribosomal RNA [Source:RFAM;Acc:RF00001]
ENSG00000222346	5S_rRNA	ENST00000410414	rRNA	111593621	1,12E+08	-	1,12E+08	42	+	5S ribosomal RNA [Source:RFAM;Acc:RF00001]
ENSG00000222354	5S_rRNA	ENST00000410422	rRNA	107664987	1,08E+08	-	1,08E+08	44	+	5S ribosomal RNA [Source:RFAM;Acc:RF00001]
ENSG00000222378	5S_rRNA	ENST00000410446	rRNA	39619769	39619968	-	39619926	42	+	5S ribosomal RNA [Source:RFAM;Acc:RF00001]
ENSG00000222383	5S_rRNA	ENST00000410451	rRNA	10752960	10753159	-	10753117	42	+	5S ribosomal RNA [Source:RFAM;Acc:RF00001]
ENSG00000222428	5S_rRNA	ENST00000410496	rRNA	68188835	68189034	-	68188993	41	+	5S ribosomal RNA [Source:RFAM;Acc:RF00001]
ENSG00000222459	5S_rRNA	ENST00000410527	rRNA	110681060	1,11E+08	-	1,11E+08	39	+	5S ribosomal RNA [Source:RFAM;Acc:RF00001]
ENSG00000222585	5S_rRNA	ENST00000410653	rRNA	26110937	26111136	-	26111094	42	+	5S ribosomal RNA [Source:RFAM;Acc:RF00001]
ENSG00000222740	5S_rRNA	ENST00000410808	rRNA	127833952	1,28E+08	-	1,28E+08	42	+	5S ribosomal RNA [Source:RFAM;Acc:RF00001]
ENSG00000222747	5S_rRNA	ENST00000410815	rRNA	21943839	21944038	-	21943996	42	+	5S ribosomal RNA [Source:RFAM;Acc:RF00001]
ENSG00000222789	5S_rRNA	ENST00000410857	rRNA	36633176	36633375	-	36633333	42	+	5S ribosomal RNA [Source:RFAM;Acc:RF00001]
ENSG00000223016	5S_rRNA	ENST00000411084	rRNA	49159937	49160136	-	49160094	42	+	5S ribosomal RNA [Source:RFAM;Acc:RF00001]
ENSG00000223086	5S_rRNA	ENST00000411154	rRNA	10117309	10117508	-	10117466	42	+	5S ribosomal RNA [Source:RFAM;Acc:RF00001]
ENSG00000223304	5S_rRNA	ENST00000411372	rRNA	24253510	24253709	-	24253625	84	+	5S ribosomal RNA [Source:RFAM;Acc:RF00001]
ENSG00000223318	5S_rRNA	ENST00000411386	rRNA	165752088	1,66E+08	-	1,66E+08	42	+	5S ribosomal RNA [Source:RFAM;Acc:RF00001]
ENSG00000238379	5S_rRNA	ENST00000459503	rRNA	129202573	1,29E+08	-	1,29E+08	41	+	5S ribosomal RNA [Source:RFAM;Acc:RF00001]
ENSG00000238391	5S_rRNA	ENST00000459153	rRNA	73901671	73901870	-	73901828	42	+	5S ribosomal RNA [Source:RFAM;Acc:RF00001]
ENSG00000238908	5S_rRNA	ENST00000458857	rRNA	39482670	39482869	-	39482827	42	+	5S ribosomal RNA [Source:RFAM;Acc:RF00001]
ENSG00000238967	5S_rRNA	ENST00000384062	rRNA	74011019	74011218	-	74011176	42	+	5S ribosomal RNA [Source:RFAM;Acc:RF00001]
ENSG00000199240	5S_rRNA	ENST00000362370	rRNA	43662088	43662287	+	43662118	30	-	5S ribosomal RNA [Source:RFAM;Acc:RF00001]

ENSEMBL55 GeneID	Gene Symbol	ENSEMBL55 TranscriptID	Biotype	Gene start	Gene end	Strand	Motif start	TSS offset	Motif strand	Description
ENSG00000199315	5S_rRNA	ENST00000362445	rRNA	97528464	97528663	+	97528523	59	-	5S ribosomal RNA [Source:RFAM;Acc:RF00001]
ENSG00000199322	5S_rRNA	ENST00000362452	rRNA	55073297	55073496	+	55073327	30	-	5S ribosomal RNA [Source:RFAM;Acc:RF00001]
ENSG00000199364	5S_rRNA	ENST00000362494	rRNA	112520900	1,13E+08	+	1,13E+08	30	-	5S ribosomal RNA [Source:RFAM;Acc:RF00001]
ENSG00000199386	5S_rRNA	ENST00000362516	rRNA	67726950	67727149	+	67726973	23	-	5S ribosomal RNA [Source:RFAM;Acc:RF00001]
ENSG00000199407	5S_rRNA	ENST00000362537	rRNA	13769796	13769995	+	13769826	30	-	5S ribosomal RNA [Source:RFAM;Acc:RF00001]
ENSG00000199415	5S_rRNA	ENST00000362545	rRNA	104519014	1,05E+08	+	1,05E+08	30	-	5S ribosomal RNA [Source:RFAM;Acc:RF00001]
ENSG00000199480	5S_rRNA	ENST00000362610	rRNA	107092677	1,07E+08	+	1,07E+08	30	-	5S ribosomal RNA [Source:RFAM;Acc:RF00001]
ENSG00000199508	5S_rRNA	ENST00000362638	rRNA	165714942	1,66E+08	+	1,66E+08	30	-	5S ribosomal RNA [Source:RFAM;Acc:RF00001]
ENSG00000199523	5S_rRNA	ENST00000362653	rRNA	165823051	1,66E+08	+	1,66E+08	30	-	5S ribosomal RNA [Source:RFAM;Acc:RF00001]
ENSG00000199525	5S_rRNA	ENST00000362655	rRNA	115014538	1,15E+08	+	1,15E+08	30	-	5S ribosomal RNA [Source:RFAM;Acc:RF00001]
ENSG00000199545	5S_rRNA	ENST00000362675	rRNA	138635541	1,39E+08	+	1,39E+08	30	-	5S ribosomal RNA [Source:RFAM;Acc:RF00001]
ENSG00000199585	5S_rRNA	ENST00000362715	rRNA	212400378	2,12E+08	+	2,12E+08	30	-	5S ribosomal RNA [Source:RFAM;Acc:RF00001]
ENSG00000199592	5S_rRNA	ENST00000362722	rRNA	79346807	79347006	+	79346837	30	-	5S ribosomal RNA [Source:RFAM;Acc:RF00001]
ENSG00000199690	5S_rRNA	ENST00000362820	rRNA	13199086	13199285	+	13199116	30	-	5S ribosomal RNA [Source:RFAM;Acc:RF00001]
ENSG00000199804	5S_rRNA	ENST00000362934	rRNA	24627812	24628011	+	24627843	31	-	5S ribosomal RNA [Source:RFAM;Acc:RF00001]
ENSG00000199929	5S_rRNA	ENST00000363059	rRNA	26040233	26040432	+	26040263	30	-	5S ribosomal RNA [Source:RFAM;Acc:RF00001]
ENSG00000199985	5S_rRNA	ENST00000363115	rRNA	36621963	36622162	+	36621993	30	-	5S ribosomal RNA [Source:RFAM;Acc:RF00001]
ENSG00000199994	5S_rRNA	ENST00000363124	rRNA	150905886	1,51E+08	+	1,51E+08	30	-	5S ribosomal RNA [Source:RFAM;Acc:RF00001]
ENSG00000200028	5S_rRNA	ENST00000363158	rRNA	77000044	77000243	+	77000065	21	-	5S ribosomal RNA [Source:RFAM;Acc:RF00001]
ENSG00000200036	5S_rRNA	ENST00000363166	rRNA	166975196	1,67E+08	+	1,67E+08	30	-	5S ribosomal RNA [Source:RFAM;Acc:RF00001]
ENSG00000200136	5S_rRNA	ENST00000363266	rRNA	34802384	34802583	+	34802414	30	-	5S ribosomal RNA [Source:RFAM;Acc:RF00001]
ENSG00000200225	5S_rRNA	ENST00000363355	rRNA	20883146	20883345	+	20883176	30	-	5S ribosomal RNA [Source:RFAM;Acc:RF00001]
ENSG00000200246	5S_rRNA	ENST00000363376	rRNA	32114012	32114211	+	32114042	30	-	5S ribosomal RNA [Source:RFAM;Acc:RF00001]
ENSG00000200248	5S_rRNA	ENST00000363378	rRNA	117381845	1,17E+08	+	1,17E+08	30	-	5S ribosomal RNA [Source:RFAM;Acc:RF00001]
ENSG00000200275	5S_rRNA	ENST00000363405	rRNA	155272447	1,55E+08	+	1,55E+08	30	-	5S ribosomal RNA [Source:RFAM;Acc:RF00001]
ENSG00000200278	5S_rRNA	ENST00000363408	rRNA	124506355	1,25E+08	+	1,25E+08	30	-	5S ribosomal RNA [Source:RFAM;Acc:RF00001]
ENSG00000200473	5S_rRNA	ENST00000363603	rRNA	69472892	69473091	+	69472922	30	-	5S ribosomal RNA [Source:RFAM;Acc:RF00001]
ENSG00000200558	5S_rRNA	ENST00000363688	rRNA	68776384	68776583	+	68776410	26	-	5S ribosomal RNA [Source:RFAM;Acc:RF00001]
ENSG00000200619	5S_rRNA	ENST00000363749	rRNA	65264226	65264425	+	65264256	30	-	5S ribosomal RNA [Source:RFAM;Acc:RF00001]
ENSG00000200741	5S_rRNA	ENST00000363871	rRNA	56963556	56963755	+	56963586	30	-	5S ribosomal RNA [Source:RFAM;Acc:RF00001]
ENSG00000200839	5S_rRNA	ENST00000363969	rRNA	52439082	52439281	+	52439112	30	-	5S ribosomal RNA [Source:RFAM;Acc:RF00001]
ENSG00000200852	5S_rRNA	ENST00000363982	rRNA	9101869	9102068	+	9101899	30	-	5S ribosomal RNA [Source:RFAM;Acc:RF00001]
ENSG00000200873	5S_rRNA	ENST00000364003	rRNA	72150911	72151110	+	72150941	30	-	5S ribosomal RNA [Source:RFAM;Acc:RF00001]
ENSG00000200911	5S_rRNA	ENST00000364041	rRNA	59765259	59765458	+	59765288	29	-	5S ribosomal RNA [Source:RFAM;Acc:RF00001]

ENSEMBL55 GeneID	Gene Symbol	ENSEMBL55 TranscriptID	Biotype	Gene start	Gene end	Strand	Motif start	TSS offset	Motif strand	Description
ENSG00000200985	5S_rRNA	ENST00000364115	rRNA	22146723	22146922	+	22146753	30	-	5S ribosomal RNA [Source:RFAM;Acc:RF00001]
ENSG00000201039	5S_rRNA	ENST00000364169	rRNA	55578305	55578504	+	55578335	30	-	5S ribosomal RNA [Source:RFAM;Acc:RF00001]
ENSG00000201086	5S_rRNA	ENST00000364216	rRNA	50274640	50274839	+	50274670	30	-	5S ribosomal RNA [Source:RFAM;Acc:RF00001]
ENSG00000201096	5S_rRNA	ENST00000364226	rRNA	76070516	76070715	+	76070546	30	-	5S ribosomal RNA [Source:RFAM;Acc:RF00001]
ENSG00000201148	5S_rRNA	ENST00000364278	rRNA	34578550	34578749	+	34578580	30	-	5S ribosomal RNA [Source:RFAM;Acc:RF00001]
ENSG00000201168	5S_rRNA	ENST00000364298	rRNA	102173592	1,02E+08	+	1,02E+08	30	-	5S ribosomal RNA [Source:RFAM;Acc:RF00001]
ENSG00000201185	5S_rRNA	ENST00000364315	rRNA	4428197	4428396	+	4428227	30	-	5S ribosomal RNA [Source:RFAM;Acc:RF00001]
ENSG00000201274	5S_rRNA	ENST00000364404	rRNA	132184269	1,32E+08	+	1,32E+08	30	-	5S ribosomal RNA [Source:RFAM;Acc:RF00001]
ENSG00000201347	5S_rRNA	ENST00000364477	rRNA	178530048	1,79E+08	+	1,79E+08	30	-	5S ribosomal RNA [Source:RFAM;Acc:RF00001]
ENSG00000201429	5S_rRNA	ENST00000364559	rRNA	66774990	66775189	+	66775020	30	-	5S ribosomal RNA [Source:RFAM;Acc:RF00001]
ENSG00000201523	5S_rRNA	ENST00000364653	rRNA	19438514	19438713	+	19438544	30	-	5S ribosomal RNA [Source:RFAM;Acc:RF00001]
ENSG00000201532	5S_rRNA	ENST00000364662	rRNA	138348018	1,38E+08	+	1,38E+08	30	-	5S ribosomal RNA [Source:RFAM;Acc:RF00001]
ENSG00000201671	5S_rRNA	ENST00000364801	rRNA	31451178	31451377	+	31451208	30	-	5S ribosomal RNA [Source:RFAM;Acc:RF00001]
ENSG00000201686	5S_rRNA	ENST00000364816	rRNA	68892323	68892522	+	68892353	30	-	5S ribosomal RNA [Source:RFAM;Acc:RF00001]
ENSG00000201727	5S_rRNA	ENST00000364857	rRNA	179327849	1,79E+08	+	1,79E+08	30	-	5S ribosomal RNA [Source:RFAM;Acc:RF00001]
ENSG00000201736	5S_rRNA	ENST00000364866	rRNA	40992171	40992370	+	40992201	30	-	5S ribosomal RNA [Source:RFAM;Acc:RF00001]
ENSG00000201763	5S_rRNA	ENST00000364893	rRNA	60368444	60368643	+	60368474	30	-	5S ribosomal RNA [Source:RFAM;Acc:RF00001]
ENSG00000201846	5S_rRNA	ENST00000364976	rRNA	149479099	1,49E+08	+	1,49E+08	30	-	5S ribosomal RNA [Source:RFAM;Acc:RF00001]
ENSG00000201890	5S_rRNA	ENST00000365020	rRNA	15220864	15221063	+	15220894	30	-	5S ribosomal RNA [Source:RFAM;Acc:RF00001]
ENSG00000201920	5S_rRNA	ENST00000365050	rRNA	39874406	39874605	+	39874436	30	-	5S ribosomal RNA [Source:RFAM;Acc:RF00001]
ENSG00000201999	5S_rRNA	ENST00000365129	rRNA	66335712	66335911	+	66335742	30	-	5S ribosomal RNA [Source:RFAM;Acc:RF00001]
ENSG00000202060	5S_rRNA	ENST00000365190	rRNA	41651548	41651747	+	41651578	30	-	5S ribosomal RNA [Source:RFAM;Acc:RF00001]
ENSG00000202187	5S_rRNA	ENST00000365317	rRNA	28658336	28658535	+	28658357	21	-	5S ribosomal RNA [Source:RFAM;Acc:RF00001]
ENSG00000202193	5S_rRNA	ENST00000365323	rRNA	53371365	53371564	+	53371395	30	-	5S ribosomal RNA [Source:RFAM;Acc:RF00001]
ENSG00000202386	5S_rRNA	ENST00000365516	rRNA	106897256	1,07E+08	+	1,07E+08	27	-	5S ribosomal RNA [Source:RFAM;Acc:RF00001]
ENSG00000202411	5S_rRNA	ENST00000365541	rRNA	28387928	28388127	+	28387958	30	-	5S ribosomal RNA [Source:RFAM;Acc:RF00001]
ENSG00000202510	5S_rRNA	ENST00000365640	rRNA	59549315	59549514	+	59549345	30	-	5S ribosomal RNA [Source:RFAM;Acc:RF00001]
ENSG00000212238	5S_rRNA	ENST00000390936	rRNA	129396106	1,29E+08	+	1,29E+08	30	-	5S ribosomal RNA [Source:RFAM;Acc:RF00001]
ENSG00000212312	5S_rRNA	ENST00000391010	rRNA	163353446	1,63E+08	+	1,63E+08	30	-	5S ribosomal RNA [Source:RFAM;Acc:RF00001]
ENSG00000212336	5S_rRNA	ENST00000391034	rRNA	82331917	82332116	+	82331947	30	-	5S ribosomal RNA [Source:RFAM;Acc:RF00001]
ENSG00000212571	5S_rRNA	ENST00000391269	rRNA	30638391	30638590	+	30638420	29	-	5S ribosomal RNA [Source:RFAM;Acc:RF00001]
ENSG00000212576	5S_rRNA	ENST00000391274	rRNA	12217312	12217511	+	12217342	30	-	5S ribosomal RNA [Source:RFAM;Acc:RF00001]
ENSG00000212625	5S_rRNA	ENST00000391323	rRNA	61028968	61029167	+	61028998	30	-	5S ribosomal RNA [Source:RFAM;Acc:RF00001]
ENSG00000222178	5S_rRNA	ENST00000410246	rRNA	36485448	36485647	+	36485475	27	-	5S ribosomal RNA [Source:RFAM;Acc:RF00001]

ENSEMBL55 GeneID	Gene Symbol	ENSEMBL55 TranscriptID	Biotype	Gene start	Gene end	Strand	Motif start	TSS offset	Motif strand	Description
ENSG00000222251	5S_rRNA	ENST00000410319	rRNA	61225887	61226086	+	61225917	30	-	5S ribosomal RNA [Source:RFAM;Acc:RF00001]
ENSG00000222416	5S_rRNA	ENST00000410484	rRNA	94453505	94453704	+	94453535	30	-	5S ribosomal RNA [Source:RFAM;Acc:RF00001]
ENSG00000222741	5S_rRNA	ENST00000410809	rRNA	33217352	33217551	+	33217382	30	-	5S ribosomal RNA [Source:RFAM;Acc:RF00001]
ENSG00000222806	5S_rRNA	ENST00000410874	rRNA	153741573	1,54E+08	+	1,54E+08	30	-	5S ribosomal RNA [Source:RFAM;Acc:RF00001]
ENSG00000222971	5S_rRNA	ENST00000411039	rRNA	146661563	1,47E+08	+	1,47E+08	23	-	5S ribosomal RNA [Source:RFAM;Acc:RF00001]
ENSG00000223013	5S_rRNA	ENST00000411081	rRNA	75645975	75646174	+	75646005	30	-	5S ribosomal RNA [Source:RFAM;Acc:RF00001]
ENSG00000223051	5S_rRNA	ENST00000411119	rRNA	52571512	52571711	+	52571542	30	-	5S ribosomal RNA [Source:RFAM;Acc:RF00001]
ENSG00000223076	5S_rRNA	ENST00000411144	rRNA	61187742	61187941	+	61187772	30	-	5S ribosomal RNA [Source:RFAM;Acc:RF00001]
ENSG00000223113	5S_rRNA	ENST00000411181	rRNA	140070238	1,4E+08	+	1,4E+08	30	-	5S ribosomal RNA [Source:RFAM;Acc:RF00001]
ENSG00000238308	5S_rRNA	ENST00000459262	rRNA	129159800	1,29E+08	+	1,29E+08	29	-	5S ribosomal RNA [Source:RFAM;Acc:RF00001]
ENSG00000238479	5S_rRNA	ENST00000459494	rRNA	58378631	58378830	+	58378661	30	-	5S ribosomal RNA [Source:RFAM;Acc:RF00001]
ENSG00000238965	5S_rRNA	ENST00000459480	rRNA	111799124	1,12E+08	+	1,12E+08	30	-	5S ribosomal RNA [Source:RFAM;Acc:RF00001]
ENSG00000199202	5S_rRNA	ENST00000362332	rRNA	98667287	98667486	+	98667318	31	+	5S ribosomal RNA [Source:RFAM;Acc:RF00001]
ENSG00000199240	5S_rRNA	ENST00000362370	rRNA	43662088	43662287	+	43662119	31	+	5S ribosomal RNA [Source:RFAM;Acc:RF00001]
ENSG00000199315	5S_rRNA	ENST00000362445	rRNA	97528464	97528663	+	97528495	31	+	5S ribosomal RNA [Source:RFAM;Acc:RF00001]
ENSG00000199322	5S_rRNA	ENST00000362452	rRNA	55073297	55073496	+	55073328	31	+	5S ribosomal RNA [Source:RFAM;Acc:RF00001]
ENSG00000199364	5S_rRNA	ENST00000362494	rRNA	112520900	1,13E+08	+	1,13E+08	31	+	5S ribosomal RNA [Source:RFAM;Acc:RF00001]
ENSG00000199386	5S_rRNA	ENST00000362516	rRNA	67726950	67727149	+	67726974	24	+	5S ribosomal RNA [Source:RFAM;Acc:RF00001]
ENSG00000199415	5S_rRNA	ENST00000362545	rRNA	104519014	1,05E+08	+	1,05E+08	31	+	5S ribosomal RNA [Source:RFAM;Acc:RF00001]
ENSG00000199455	5S_rRNA	ENST00000362585	rRNA	129450105	1,29E+08	+	1,29E+08	22	+	5S ribosomal RNA [Source:RFAM;Acc:RF00001]
ENSG00000199480	5S_rRNA	ENST00000362610	rRNA	107092677	1,07E+08	+	1,07E+08	31	+	5S ribosomal RNA [Source:RFAM;Acc:RF00001]
ENSG00000199508	5S_rRNA	ENST00000362638	rRNA	165714942	1,66E+08	+	1,66E+08	31	+	5S ribosomal RNA [Source:RFAM;Acc:RF00001]
ENSG00000199523	5S_rRNA	ENST00000362653	rRNA	165823051	1,66E+08	+	1,66E+08	31	+	5S ribosomal RNA [Source:RFAM;Acc:RF00001]
ENSG00000199525	5S_rRNA	ENST00000362655	rRNA	115014538	1,15E+08	+	1,15E+08	31	+	5S ribosomal RNA [Source:RFAM;Acc:RF00001]
ENSG00000199556	5S_rRNA	ENST00000362686	rRNA	45511554	45511753	+	45511584	30	+	5S ribosomal RNA [Source:RFAM;Acc:RF00001]
ENSG00000199585	5S_rRNA	ENST00000362715	rRNA	212400378	2,12E+08	+	2,12E+08	31	+	5S ribosomal RNA [Source:RFAM;Acc:RF00001]
ENSG00000199592	5S_rRNA	ENST00000362722	rRNA	79346807	79347006	+	79346838	31	+	5S ribosomal RNA [Source:RFAM;Acc:RF00001]
ENSG00000199638	5S_rRNA	ENST00000362768	rRNA	70221164	70221363	+	70221195	31	+	5S ribosomal RNA [Source:RFAM;Acc:RF00001]
ENSG00000199639	5S_rRNA	ENST00000362769	rRNA	43385832	43386031	+	43385863	31	+	5S ribosomal RNA [Source:RFAM;Acc:RF00001]
ENSG00000199690	5S_rRNA	ENST00000362820	rRNA	13199086	13199285	+	13199117	31	+	5S ribosomal RNA [Source:RFAM;Acc:RF00001]
ENSG00000199733	5S_rRNA	ENST00000362863	rRNA	26798518	26798717	+	26798549	31	+	5S ribosomal RNA [Source:RFAM;Acc:RF00001]
ENSG00000199804	5S_rRNA	ENST00000362934	rRNA	24627812	24628011	+	24627844	32	+	5S ribosomal RNA [Source:RFAM;Acc:RF00001]
ENSG00000199809	5S_rRNA	ENST00000362939	rRNA	119267491	1,19E+08	+	1,19E+08	26	+	5S ribosomal RNA [Source:RFAM;Acc:RF00001]
ENSG00000199811	5S_rRNA	ENST00000362941	rRNA	186341760	1,86E+08	+	1,86E+08	31	+	5S ribosomal RNA [Source:RFAM;Acc:RF00001]

ENSEMBL55 GeneID	Gene Symbol	ENSEMBL55 TranscriptID	Biotype	Gene start	Gene end	Strand	Motif start	TSS offset	Motif strand	Description
ENSG00000199929	5S_rRNA	ENST00000363059	rRNA	26040233	26040432	+	26040264	31	+	5S ribosomal RNA [Source:RFAM;Acc:RF00001]
ENSG00000199985	5S_rRNA	ENST00000363115	rRNA	36621963	36622162	+	36621994	31	+	5S ribosomal RNA [Source:RFAM;Acc:RF00001]
ENSG00000199994	5S_rRNA	ENST00000363124	rRNA	150905886	1,51E+08	+	1,51E+08	31	+	5S ribosomal RNA [Source:RFAM;Acc:RF00001]
ENSG00000200028	5S_rRNA	ENST00000363158	rRNA	77000044	77000243	+	77000066	22	+	5S ribosomal RNA [Source:RFAM;Acc:RF00001]
ENSG00000200036	5S_rRNA	ENST00000363166	rRNA	166975196	1,67E+08	+	1,67E+08	31	+	5S ribosomal RNA [Source:RFAM;Acc:RF00001]
ENSG00000200136	5S_rRNA	ENST00000363266	rRNA	34802384	34802583	+	34802415	31	+	5S ribosomal RNA [Source:RFAM;Acc:RF00001]
ENSG00000200225	5S_rRNA	ENST00000363355	rRNA	20883146	20883345	+	20883177	31	+	5S ribosomal RNA [Source:RFAM;Acc:RF00001]
ENSG00000200246	5S_rRNA	ENST00000363376	rRNA	32114012	32114211	+	32114043	31	+	5S ribosomal RNA [Source:RFAM;Acc:RF00001]
ENSG00000200275	5S_rRNA	ENST00000363405	rRNA	155272447	1,55E+08	+	1,55E+08	31	+	5S ribosomal RNA [Source:RFAM;Acc:RF00001]
ENSG00000200278	5S_rRNA	ENST00000363408	rRNA	124506355	1,25E+08	+	1,25E+08	31	+	5S ribosomal RNA [Source:RFAM;Acc:RF00001]
ENSG00000200558	5S_rRNA	ENST00000363688	rRNA	68776384	68776583	+	68776411	27	+	5S ribosomal RNA [Source:RFAM;Acc:RF00001]
ENSG00000200619	5S_rRNA	ENST00000363749	rRNA	65264226	65264425	+	65264257	31	+	5S ribosomal RNA [Source:RFAM;Acc:RF00001]
ENSG00000200741	5S_rRNA	ENST00000363871	rRNA	56963556	56963755	+	56963587	31	+	5S ribosomal RNA [Source:RFAM;Acc:RF00001]
ENSG00000200786	5S_rRNA	ENST00000363916	rRNA	79283425	79283624	+	79283456	31	+	5S ribosomal RNA [Source:RFAM;Acc:RF00001]
ENSG00000200805	5S_rRNA	ENST00000363935	rRNA	115695291	1,16E+08	+	1,16E+08	31	+	5S ribosomal RNA [Source:RFAM;Acc:RF00001]
ENSG00000200839	5S_rRNA	ENST00000363969	rRNA	52439082	52439281	+	52439113	31	+	5S ribosomal RNA [Source:RFAM;Acc:RF00001]
ENSG00000200852	5S_rRNA	ENST00000363982	rRNA	9101869	9102068	+	9101900	31	+	5S ribosomal RNA [Source:RFAM;Acc:RF00001]
ENSG00000200873	5S_rRNA	ENST00000364003	rRNA	72150911	72151110	+	72150942	31	+	5S ribosomal RNA [Source:RFAM;Acc:RF00001]
ENSG00000200911	5S_rRNA	ENST00000364041	rRNA	59765259	59765458	+	59765289	30	+	5S ribosomal RNA [Source:RFAM;Acc:RF00001]
ENSG00000200985	5S_rRNA	ENST00000364115	rRNA	22146723	22146922	+	22146754	31	+	5S ribosomal RNA [Source:RFAM;Acc:RF00001]
ENSG00000201000	5S_rRNA	ENST00000364130	rRNA	141498839	1,41E+08	+	1,41E+08	31	+	5S ribosomal RNA [Source:RFAM;Acc:RF00001]
ENSG00000201039	5S_rRNA	ENST00000364169	rRNA	55578305	55578504	+	55578336	31	+	5S ribosomal RNA [Source:RFAM;Acc:RF00001]
ENSG00000201041	5S_rRNA	ENST00000364171	rRNA	128337493	1,28E+08	+	1,28E+08	30	+	5S ribosomal RNA [Source:RFAM;Acc:RF00001]
ENSG00000201086	5S_rRNA	ENST00000364216	rRNA	50274640	50274839	+	50274671	31	+	5S ribosomal RNA [Source:RFAM;Acc:RF00001]
ENSG00000201096	5S_rRNA	ENST00000364226	rRNA	76070516	76070715	+	76070547	31	+	5S ribosomal RNA [Source:RFAM;Acc:RF00001]
ENSG00000201148	5S_rRNA	ENST00000364278	rRNA	34578550	34578749	+	34578581	31	+	5S ribosomal RNA [Source:RFAM;Acc:RF00001]
ENSG00000201168	5S_rRNA	ENST00000364298	rRNA	102173592	1,02E+08	+	1,02E+08	31	+	5S ribosomal RNA [Source:RFAM;Acc:RF00001]
ENSG00000201185	5S_rRNA	ENST00000364315	rRNA	4428197	4428396	+	4428228	31	+	5S ribosomal RNA [Source:RFAM;Acc:RF00001]
ENSG00000201274	5S_rRNA	ENST00000364404	rRNA	132184269	1,32E+08	+	1,32E+08	31	+	5S ribosomal RNA [Source:RFAM;Acc:RF00001]
ENSG00000201347	5S_rRNA	ENST00000364477	rRNA	178530048	1,79E+08	+	1,79E+08	31	+	5S ribosomal RNA [Source:RFAM;Acc:RF00001]
ENSG00000201429	5S_rRNA	ENST00000364559	rRNA	66774990	66775189	+	66775021	31	+	5S ribosomal RNA [Source:RFAM;Acc:RF00001]
ENSG00000201523	5S_rRNA	ENST00000364653	rRNA	19438514	19438713	+	19438545	31	+	5S ribosomal RNA [Source:RFAM;Acc:RF00001]
ENSG00000201532	5S_rRNA	ENST00000364662	rRNA	138348018	1,38E+08	+	1,38E+08	31	+	5S ribosomal RNA [Source:RFAM;Acc:RF00001]
ENSG00000201567	5S_rRNA	ENST00000364697	rRNA	94837063	94837262	+	94837094	31	+	5S ribosomal RNA [Source:RFAM;Acc:RF00001]

ENSEMBL55 GeneID	Gene Symbol	ENSEMBL55 TranscriptID	Biotype	Gene start	Gene end	Strand	Motif start	TSS offset	Motif strand	Description
ENSG00000201671	5S_rRNA	ENST00000364801	rRNA	31451178	31451377	+	31451209	31	+	5S ribosomal RNA [Source:RFAM;Acc:RF00001]
ENSG00000201686	5S_rRNA	ENST00000364816	rRNA	68892323	68892522	+	68892354	31	+	5S ribosomal RNA [Source:RFAM;Acc:RF00001]
ENSG00000201695	5S_rRNA	ENST00000364825	rRNA	18287940	18288139	+	18287971	31	+	5S ribosomal RNA [Source:RFAM;Acc:RF00001]
ENSG00000201713	5S_rRNA	ENST00000364843	rRNA	25066498	25066697	+	25066526	28	+	5S ribosomal RNA [Source:RFAM;Acc:RF00001]
ENSG00000201727	5S_rRNA	ENST00000364857	rRNA	179327849	1,79E+08	+	1,79E+08	31	+	5S ribosomal RNA [Source:RFAM;Acc:RF00001]
ENSG00000201763	5S_rRNA	ENST00000364893	rRNA	60368444	60368643	+	60368475	31	+	5S ribosomal RNA [Source:RFAM;Acc:RF00001]
ENSG00000201846	5S_rRNA	ENST00000364976	rRNA	149479099	1,49E+08	+	1,49E+08	31	+	5S ribosomal RNA [Source:RFAM;Acc:RF00001]
ENSG00000201920	5S_rRNA	ENST00000365050	rRNA	39874406	39874605	+	39874437	31	+	5S ribosomal RNA [Source:RFAM;Acc:RF00001]
ENSG00000201999	5S_rRNA	ENST00000365129	rRNA	66335712	66335911	+	66335743	31	+	5S ribosomal RNA [Source:RFAM;Acc:RF00001]
ENSG00000202047	5S_rRNA	ENST00000365177	rRNA	98379133	98379332	+	98379164	31	+	5S ribosomal RNA [Source:RFAM;Acc:RF00001]
ENSG00000202060	5S_rRNA	ENST00000365190	rRNA	41651548	41651747	+	41651579	31	+	5S ribosomal RNA [Source:RFAM;Acc:RF00001]
ENSG00000202187	5S_rRNA	ENST00000365317	rRNA	28658336	28658535	+	28658358	22	+	5S ribosomal RNA [Source:RFAM;Acc:RF00001]
ENSG00000202193	5S_rRNA	ENST00000365323	rRNA	53371365	53371564	+	53371396	31	+	5S ribosomal RNA [Source:RFAM;Acc:RF00001]
ENSG00000202264	5S_rRNA	ENST00000365394	rRNA	227748882	2,28E+08	+	2,28E+08	31	+	5S ribosomal RNA [Source:RFAM;Acc:RF00001]
ENSG00000202322	5S_rRNA	ENST00000365452	rRNA	50457212	50457411	+	50457243	31	+	5S ribosomal RNA [Source:RFAM;Acc:RF00001]
ENSG00000202331	5S_rRNA	ENST00000365461	rRNA	150828720	1,51E+08	+	1,51E+08	23	+	5S ribosomal RNA [Source:RFAM;Acc:RF00001]
ENSG00000202383	5S_rRNA	ENST00000365513	rRNA	1333837	1334036	+	1333865	28	+	5S ribosomal RNA [Source:RFAM;Acc:RF00001]
ENSG00000202510	5S_rRNA	ENST00000365640	rRNA	59549315	59549514	+	59549346	31	+	5S ribosomal RNA [Source:RFAM;Acc:RF00001]
ENSG00000212238	5S_rRNA	ENST00000390936	rRNA	129396106	1,29E+08	+	1,29E+08	31	+	5S ribosomal RNA [Source:RFAM;Acc:RF00001]
ENSG00000212242	5S_rRNA	ENST00000390940	rRNA	136951378	1,37E+08	+	1,37E+08	31	+	5S ribosomal RNA [Source:RFAM;Acc:RF00001]
ENSG00000212280	5S_rRNA	ENST00000390978	rRNA	17605627	17605826	+	17605658	31	+	5S ribosomal RNA [Source:RFAM;Acc:RF00001]
ENSG00000212312	5S_rRNA	ENST00000391010	rRNA	163353446	1,63E+08	+	1,63E+08	31	+	5S ribosomal RNA [Source:RFAM;Acc:RF00001]
ENSG00000212336	5S_rRNA	ENST00000391034	rRNA	82331917	82332116	+	82331948	31	+	5S ribosomal RNA [Source:RFAM;Acc:RF00001]
ENSG00000212365	5S_rRNA	ENST00000391063	rRNA	14156532	14156731	+	14156563	31	+	5S ribosomal RNA [Source:RFAM;Acc:RF00001]
ENSG00000212527	5S_rRNA	ENST00000391225	rRNA	163479274	1,63E+08	+	1,63E+08	31	+	5S ribosomal RNA [Source:RFAM;Acc:RF00001]
ENSG00000212542	5S_rRNA	ENST00000391240	rRNA	31701390	31701589	+	31701421	31	+	5S ribosomal RNA [Source:RFAM;Acc:RF00001]
ENSG00000212571	5S_rRNA	ENST00000391269	rRNA	30638391	30638590	+	30638421	30	+	5S ribosomal RNA [Source:RFAM;Acc:RF00001]
ENSG00000212574	5S_rRNA	ENST00000391272	rRNA	13639651	13639850	+	13639682	31	+	5S ribosomal RNA [Source:RFAM;Acc:RF00001]
ENSG00000212576	5S_rRNA	ENST00000391274	rRNA	12217312	12217511	+	12217343	31	+	5S ribosomal RNA [Source:RFAM;Acc:RF00001]
ENSG00000212625	5S_rRNA	ENST00000391323	rRNA	61028968	61029167	+	61028999	31	+	5S ribosomal RNA [Source:RFAM;Acc:RF00001]
ENSG00000222123	5S_rRNA	ENST00000410191	rRNA	25983655	25983854	+	25983686	31	+	5S ribosomal RNA [Source:RFAM;Acc:RF00001]
ENSG00000222165	5S_rRNA	ENST00000410233	rRNA	6890784	6890983	+	6890810	26	+	5S ribosomal RNA [Source:RFAM;Acc:RF00001]
ENSG00000222165	5S_rRNA	ENST00000410233	rRNA	6890784	6890983	+	6890815	31	+	5S ribosomal RNA [Source:RFAM;Acc:RF00001]
ENSG00000222178	5S_rRNA	ENST00000410246	rRNA	36485448	36485647	+	36485476	28	+	5S ribosomal RNA [Source:RFAM;Acc:RF00001]

ENSEMBL55 GeneID	Gene Symbol	ENSEMBL55 TranscriptID	Biotype	Gene start	Gene end	Strand	Motif start	TSS offset	Motif strand	Description
ENSG00000222208	5S_rRNA	ENST00000410276	rRNA	37383140	37383339	+	37383171	31	+	5S ribosomal RNA [Source:RFAM;Acc:RF00001]
ENSG00000222244	5S_rRNA	ENST00000410312	rRNA	79298351	79298550	+	79298382	31	+	5S ribosomal RNA [Source:RFAM;Acc:RF00001]
ENSG00000222251	5S_rRNA	ENST00000410319	rRNA	61225887	61226086	+	61225918	31	+	5S ribosomal RNA [Source:RFAM;Acc:RF00001]
ENSG00000222268	5S_rRNA	ENST00000410336	rRNA	47539343	47539542	+	47539372	29	+	5S ribosomal RNA [Source:RFAM;Acc:RF00001]
ENSG00000222416	5S_rRNA	ENST00000410484	rRNA	94453505	94453704	+	94453536	31	+	5S ribosomal RNA [Source:RFAM;Acc:RF00001]
ENSG00000222418	5S_rRNA	ENST00000410486	rRNA	182913549	1,83E+08	+	1,83E+08	31	+	5S ribosomal RNA [Source:RFAM;Acc:RF00001]
ENSG00000222608	5S_rRNA	ENST00000410676	rRNA	52694281	52694480	+	52694311	30	+	5S ribosomal RNA [Source:RFAM;Acc:RF00001]
ENSG00000222665	5S_rRNA	ENST00000410733	rRNA	43720573	43720772	+	43720594	21	+	5S ribosomal RNA [Source:RFAM;Acc:RF00001]
ENSG00000222665	5S_rRNA	ENST00000410733	rRNA	43720573	43720772	+	43720599	26	+	5S ribosomal RNA [Source:RFAM;Acc:RF00001]
ENSG00000222806	5S_rRNA	ENST00000410874	rRNA	153741573	1,54E+08	+	1,54E+08	31	+	5S ribosomal RNA [Source:RFAM;Acc:RF00001]
ENSG00000222835	5S_rRNA	ENST00000410903	rRNA	91862883	91863082	+	91862913	30	+	5S ribosomal RNA [Source:RFAM;Acc:RF00001]
ENSG00000222955	5S_rRNA	ENST00000411023	rRNA	56657792	56657991	+	56657818	26	+	5S ribosomal RNA [Source:RFAM;Acc:RF00001]
ENSG00000222971	5S_rRNA	ENST00000411039	rRNA	146661563	1,47E+08	+	1,47E+08	24	+	5S ribosomal RNA [Source:RFAM;Acc:RF00001]
ENSG00000222983	5S_rRNA	ENST00000411051	rRNA	31270207	31270406	+	31270238	31	+	5S ribosomal RNA [Source:RFAM;Acc:RF00001]
ENSG00000223051	5S_rRNA	ENST00000411119	rRNA	52571512	52571711	+	52571543	31	+	5S ribosomal RNA [Source:RFAM;Acc:RF00001]
ENSG00000223076	5S_rRNA	ENST00000411144	rRNA	61187742	61187941	+	61187773	31	+	5S ribosomal RNA [Source:RFAM;Acc:RF00001]
ENSG00000223138	5S_rRNA	ENST00000411206	rRNA	9923917	9924116	+	9923947	30	+	5S ribosomal RNA [Source:RFAM;Acc:RF00001]
ENSG00000238308	5S_rRNA	ENST00000459262	rRNA	129159800	1,29E+08	+	1,29E+08	30	+	5S ribosomal RNA [Source:RFAM;Acc:RF00001]
ENSG00000238479	5S_rRNA	ENST00000459494	rRNA	58378631	58378830	+	58378662	31	+	5S ribosomal RNA [Source:RFAM;Acc:RF00001]
ENSG00000238602	5S_rRNA	ENST00000458901	rRNA	415329	415528	+	415360	31	+	5S ribosomal RNA [Source:RFAM;Acc:RF00001]
ENSG00000238677	5S_rRNA	ENST00000459025	rRNA	147665990	1,48E+08	+	1,48E+08	31	+	5S ribosomal RNA [Source:RFAM;Acc:RF00001]
ENSG00000238765	5S_rRNA	ENST00000459463	rRNA	146124158	1,46E+08	+	1,46E+08	31	+	5S ribosomal RNA [Source:RFAM;Acc:RF00001]
ENSG00000238965	5S_rRNA	ENST00000459480	rRNA	111799124	1,12E+08	+	1,12E+08	31	+	5S ribosomal RNA [Source:RFAM;Acc:RF00001]
ENSG00000239184	5S_rRNA	ENST00000459032	rRNA	69607878	69608077	+	69607909	31	+	5S ribosomal RNA [Source:RFAM;Acc:RF00001]
ENSG00000206780	SNORA75	ENST00000384053	snoRNA	17322306	17322505	-	17322463	42	+	Small nucleolar RNA SNORA75 [Source:RFAM;Acc:RF00072]
ENSG00000200623	SNORD18	ENST00000363753	snoRNA	66795453	66795652	-	66795592	60	+	Small nucleolar RNA SNORD18 [Source:RFAM;Acc:RF00093]
ENSG00000212610	U3	ENST00000391308	snoRNA	218715042	2,19E+08	-	2,19E+08	29	+	Small nucleolar RNA U3 [Source:RFAM;Acc:RF00012]
ENSG00000199713	U8	ENST00000362843	snoRNA	56486049	56486248	-	56486191	57	+	U8 small nucleolar RNA [Source:RFAM;Acc:RF00096]
ENSG00000200463	U8	ENST00000363593	snoRNA	8076706	8076905	-	8076838	67	+	U8 small nucleolar RNA [Source:RFAM;Acc:RF00096]
ENSG00000201809	U8	ENST00000364939	snoRNA	74157994	74158193	-	74158125	68	+	U8 small nucleolar RNA [Source:RFAM;Acc:RF00096]
ENSG00000238496	snoU13	ENST00000458859	snoRNA	9789134	9789333	-	9789279	54	+	Small nucleolar RNA U13 [Source:RFAM;Acc:RF01210]
ENSG00000221116	SNORD110	ENST00000408189	snoRNA	2634858	2635057	+	2634938	80	-	Small Nucleolar RNA SNORD110 [Source:RFAM;Acc:RF00610]
ENSG00000199942	SNORD113	ENST00000363072	snoRNA	101442814	1,01E+08	+	1,01E+08	37	-	Small nucleolar RNA SNORD113/SNORD114 family [Source:RFAM;Acc:RF00181]

ENSEMBL55 GeneID	Gene Symbol	ENSEMBL55 TranscriptID	Biotype	Gene start	Gene end	Strand	Motif start	TSS offset	Motif strand	Description
ENSG00000201264	SNORD73	ENST00000364394	snoRNA	152023209	1,52E+08	+	1,52E+08	33	-	Small nucleolar RNA SNORD73 [Source:RFAM;Acc:RF00071]
ENSG00000208797	SNORD73	ENST00000386062	snoRNA	152024979	1,52E+08	+	1,52E+08	32	-	Small nucleolar RNA SNORD73 [Source:RFAM;Acc:RF00071]
ENSG00000200026	U8	ENST00000363156	snoRNA	38147425	38147624	+	38147480	55	-	U8 small nucleolar RNA [Source:RFAM;Acc:RF00096]
ENSG00000200026	U8	ENST00000363156	snoRNA	38147425	38147624	+	38147470	45	-	U8 small nucleolar RNA [Source:RFAM;Acc:RF00096]
ENSG00000200496	U8	ENST00000363626	snoRNA	123172098	1,23E+08	+	1,23E+08	55	-	U8 small nucleolar RNA [Source:RFAM;Acc:RF00096]
ENSG00000200496	U8	ENST00000363626	snoRNA	123172098	1,23E+08	+	1,23E+08	45	-	U8 small nucleolar RNA [Source:RFAM;Acc:RF00096]
ENSG00000212249	U8	ENST00000390947	snoRNA	68169783	68169982	+	68169827	44	-	U8 small nucleolar RNA [Source:RFAM;Acc:RF00096]
ENSG00000212249	U8	ENST00000390947	snoRNA	68169783	68169982	+	68169837	54	-	U8 small nucleolar RNA [Source:RFAM;Acc:RF00096]
ENSG00000238871	snoU13	ENST00000459008	snoRNA	38215874	38216073	+	38215897	23	-	Small nucleolar RNA U13 [Source:RFAM;Acc:RF01210]
ENSG00000238597	snoZ17	ENST00000459083	snoRNA	27050700	27050899	+	27050735	35	-	Small nucleolar RNA Z17 [Source:RFAM;Acc:RF00266]
ENSG00000238527	SNORD112	ENST00000458974	snoRNA	101364257	1,01E+08	+	1,01E+08	35	+	Small nucleolar RNA SNORD112 [Source:RFAM;Acc:RF01169]
ENSG00000238505	SNORD11B	ENST00000459250	snoRNA	203156055	2,03E+08	+	2,03E+08	76	+	Small nucleolar RNA SNORD11B [Source:RFAM;Acc:RF01192]
ENSG00000212618	snoMBII-202	ENST00000391316	snoRNA	18847312	18847511	+	18847353	41	+	Small nucleolar RNA MBII-202 [Source:RFAM;Acc:RF00324]
ENSG00000222137	U2	ENST00000410205	snRNA	166214262	1,66E+08	-	1,66E+08	49	-	U2 spliceosomal RNA [Source:RFAM;Acc:RF00004]
ENSG00000222293	U2	ENST00000410361	snRNA	89037844	89038043	-	89037994	49	-	U2 spliceosomal RNA [Source:RFAM;Acc:RF00004]
ENSG00000222300	U2	ENST00000410368	snRNA	159792298	1,6E+08	-	1,6E+08	49	-	U2 spliceosomal RNA [Source:RFAM;Acc:RF00004]
ENSG00000222328	U2	ENST00000410396	snRNA	62609082	62609281	-	62609232	49	-	U2 spliceosomal RNA [Source:RFAM;Acc:RF00004]
ENSG00000222414	U2	ENST00000410482	snRNA	103124593	1,03E+08	-	1,03E+08	49	-	U2 spliceosomal RNA [Source:RFAM;Acc:RF00004]
ENSG00000222581	U2	ENST00000410649	snRNA	12300252	12300451	-	12300402	49	-	U2 spliceosomal RNA [Source:RFAM;Acc:RF00004]
ENSG00000222598	U2	ENST00000410666	snRNA	115110000	1,15E+08	-	1,15E+08	46	-	U2 spliceosomal RNA [Source:RFAM;Acc:RF00004]
ENSG00000222626	U2	ENST00000410694	snRNA	157403765	1,57E+08	-	1,57E+08	49	-	U2 spliceosomal RNA [Source:RFAM;Acc:RF00004]
ENSG00000222637	U2	ENST00000410705	snRNA	85287606	85287805	-	85287756	49	-	U2 spliceosomal RNA [Source:RFAM;Acc:RF00004]
ENSG00000222644	U2	ENST00000410712	snRNA	76750543	76750742	-	76750693	49	-	U2 spliceosomal RNA [Source:RFAM;Acc:RF00004]
ENSG00000222729	U2	ENST00000410797	snRNA	110886048	1,11E+08	-	1,11E+08	49	-	U2 spliceosomal RNA [Source:RFAM;Acc:RF00004]
ENSG00000222768	U2	ENST00000410836	snRNA	86523057	86523256	-	86523207	49	-	U2 spliceosomal RNA [Source:RFAM;Acc:RF00004]
ENSG00000222810	U2	ENST00000410878	snRNA	71596820	71597019	-	71596970	49	-	U2 spliceosomal RNA [Source:RFAM;Acc:RF00004]
ENSG00000222944	U2	ENST00000411012	snRNA	41464586	41464785	-	41464736	49	-	U2 spliceosomal RNA [Source:RFAM;Acc:RF00004]
ENSG00000222985	U2	ENST00000411053	snRNA	65591061	65591260	-	65591211	49	-	U2 spliceosomal RNA [Source:RFAM;Acc:RF00004]
ENSG00000223078	U2	ENST00000411146	snRNA	24654032	24654231	-	24654182	49	-	U2 spliceosomal RNA [Source:RFAM;Acc:RF00004]
ENSG00000223327	U2	ENST00000411395	snRNA	81497809	81498008	-	81497950	58	-	U2 spliceosomal RNA [Source:RFAM;Acc:RF00004]
ENSG00000223327	U2	ENST00000411395	snRNA	81497809	81498008	-	81497960	48	-	U2 spliceosomal RNA [Source:RFAM;Acc:RF00004]
ENSG00000223336	U2	ENST00000411404	snRNA	46948526	46948725	-	46948676	49	-	U2 spliceosomal RNA [Source:RFAM;Acc:RF00004]
ENSG00000223336	U2	ENST00000411404	snRNA	46948526	46948725	-	46948666	59	-	U2 spliceosomal RNA [Source:RFAM;Acc:RF00004]

ENSEMBL55 GeneID	Gene Symbol	ENSEMBL55 TranscriptID	Biotype	Gene start	Gene end	Strand	Motif start	TSS offset	Motif strand	Description
ENSG00000238973	U2	ENST00000459396	snRNA	69980077	69980276	-	69980227	49	-	U2 spliceosomal RNA [Source:RFAM;Acc:RF00004]
ENSG00000239019	U2	ENST00000459567	snRNA	79858750	79858949	-	79858900	49	-	U2 spliceosomal RNA [Source:RFAM;Acc:RF00004]
ENSG00000202125	U1	ENST00000365255	snRNA	142139012	1,42E+08	-	1,42E+08	95	+	U1 spliceosomal RNA [Source:RFAM;Acc:RF00003]
ENSG00000200274	U4	ENST00000363404	snRNA	110005721	1,1E+08	-	1,1E+08	61	+	U4 spliceosomal RNA [Source:RFAM;Acc:RF00015]
ENSG00000199235	U6	ENST00000362365	snRNA	87359296	87359495	-	87359427	68	+	U6 spliceosomal RNA [Source:RFAM;Acc:RF00026]
ENSG00000200097	U6	ENST00000363227	snRNA	35539781	35539980	-	35539943	37	+	U6 spliceosomal RNA [Source:RFAM;Acc:RF00026]
ENSG00000201517	U6	ENST00000364647	snRNA	48013303	48013502	-	48013419	83	+	U6 spliceosomal RNA [Source:RFAM;Acc:RF00026]
ENSG00000212410	U6	ENST00000391108	snRNA	158842207	1,59E+08	-	1,59E+08	83	+	U6 spliceosomal RNA [Source:RFAM;Acc:RF00026]
ENSG00000206938	U4	ENST00000384211	snRNA	156664772	1,57E+08	+	1,57E+08	49	-	U4 spliceosomal RNA [Source:RFAM;Acc:RF00015]
ENSG00000223245	U4	ENST00000411313	snRNA	42098411	42098610	+	42098460	49	-	U4 spliceosomal RNA [Source:RFAM;Acc:RF00015]
ENSG00000201443	U6	ENST00000364573	snRNA	108069994	1,08E+08	+	1,08E+08	26	-	U6 spliceosomal RNA [Source:RFAM;Acc:RF00026]
ENSG00000206839	U6	ENST00000384112	snRNA	56160657	56160856	+	56160694	37	-	U6 spliceosomal RNA [Source:RFAM;Acc:RF00026]
ENSG00000212382	U6	ENST00000391080	snRNA	27874572	27874771	+	27874609	37	-	U6 spliceosomal RNA [Source:RFAM;Acc:RF00026]
ENSG00000212460	U6	ENST00000391158	snRNA	137452262	1,37E+08	+	1,37E+08	37	-	U6 spliceosomal RNA [Source:RFAM;Acc:RF00026]
ENSG00000222076	U2	ENST00000410144	snRNA	96289033	96289232	+	96289070	37	+	U2 spliceosomal RNA [Source:RFAM;Acc:RF00004]
ENSG00000222198	U2	ENST00000410266	snRNA	18342408	18342607	+	18342445	37	+	U2 spliceosomal RNA [Source:RFAM;Acc:RF00004]
ENSG00000222269	U2	ENST00000410337	snRNA	149959564	1,5E+08	+	1,5E+08	37	+	U2 spliceosomal RNA [Source:RFAM;Acc:RF00004]
ENSG00000222276	U2	ENST00000410344	snRNA	96850961	96851160	+	96850998	37	+	U2 spliceosomal RNA [Source:RFAM;Acc:RF00004]
ENSG00000222355	U2	ENST00000410423	snRNA	53843829	53844028	+	53843866	37	+	U2 spliceosomal RNA [Source:RFAM;Acc:RF00004]
ENSG00000222357	U2	ENST00000410425	snRNA	168192986	1,68E+08	+	1,68E+08	37	+	U2 spliceosomal RNA [Source:RFAM;Acc:RF00004]
ENSG00000222389	U2	ENST00000410457	snRNA	81558627	81558826	+	81558664	37	+	U2 spliceosomal RNA [Source:RFAM;Acc:RF00004]
ENSG00000222440	U2	ENST00000410508	snRNA	91053092	91053291	+	91053129	37	+	U2 spliceosomal RNA [Source:RFAM;Acc:RF00004]
ENSG00000222477	U2	ENST00000410545	snRNA	64915055	64915254	+	64915092	37	+	U2 spliceosomal RNA [Source:RFAM;Acc:RF00004]
ENSG00000222624	U2	ENST00000410692	snRNA	65881499	65881698	+	65881535	36	+	U2 spliceosomal RNA [Source:RFAM;Acc:RF00004]
ENSG00000222716	U2	ENST00000410784	snRNA	25991889	25992088	+	25991926	37	+	U2 spliceosomal RNA [Source:RFAM;Acc:RF00004]
ENSG00000222788	U2	ENST00000410856	snRNA	144944672	1,45E+08	+	1,45E+08	37	+	U2 spliceosomal RNA [Source:RFAM;Acc:RF00004]
ENSG00000222840	U2	ENST00000410908	snRNA	127867711	1,28E+08	+	1,28E+08	37	+	U2 spliceosomal RNA [Source:RFAM;Acc:RF00004]
ENSG00000222923	U2	ENST00000410991	snRNA	115177702	1,15E+08	+	1,15E+08	37	+	U2 spliceosomal RNA [Source:RFAM;Acc:RF00004]
ENSG00000222959	U2	ENST00000411027	snRNA	29769567	29769766	+	29769604	37	+	U2 spliceosomal RNA [Source:RFAM;Acc:RF00004]
ENSG00000223125	U2	ENST00000411193	snRNA	39624208	39624407	+	39624245	37	+	U2 spliceosomal RNA [Source:RFAM;Acc:RF00004]
ENSG00000222563	U6	ENST00000410631	snRNA	69307281	69307480	+	69307354	73	+	U6 spliceosomal RNA [Source:RFAM;Acc:RF00026]
