# Supplementary material for: A tandem sequence motif acts as a distance-dependent enhancer in a set of genes involved in translation by binding the proteins NonO and SFPQ
Source: BMC Genomics. 2011 Dec 20;12:624. doi: 10.1186/1471-2164-12-624 (PMC3262029; doi:10.1186/1471-2164-12-624)
Supplement: Additional file 5 — Supplementary Table S4. Tripartite LTSM-positive genes from a genome-wide search [file 1471-2164-12-624-S5.PDF]

Additional file 5 – Supplementary Table 4. Tripartite LTSM-positive genes from a genome-wide search

ENSEMBL55GeneID	GeneSymbol	ENSEMBL55TranscriptID	Biotype	GeneStart	GeneEnd	GeneStrand	Motifstart	TSSoffset	Motifstrand	Description
ENSG00000221503	AL451073.17	ENST00000408576	miRNA	1,28E+08	1,28E+08	-	1,28E+08	95	+	
ENSG00000222556	Y_RNA	ENST00000410624	misc_RNA	1,86E+08	1,86E+08	-	1,86E+08	98	-	Y RNA [Source:RFAM;Acc:RF00019]
ENSG00000232898	AC112509.4	ENST00000424821	protein_coding	82302772	82302971	-	82302928	21	+	
ENSG00000166845	C18orf54	ENST00000300091	protein_coding	51885171	51885370	+	51885245	74	-	Uncharacterized protein C18orf54 Precursor [Source:UniProtKB/Swiss-Prot;Acc:Q8IYD9]
ENSG00000166845	C18orf54	ENST00000382911	protein_coding	51885171	51885370	+	51885245	74	-	Uncharacterized protein C18orf54 Precursor [Source:UniProtKB/Swiss-Prot;Acc:Q8IYD9]
ENSG00000164989	C9orf93	ENST00000430161	protein_coding	15864588	15864787	+	15864688	100	-	Uncharacterized protein C9orf93 [Source:UniProtKB/Swiss-Prot;Acc:Q6TFL3]
ENSG00000106078	COBL	ENST00000333663	protein_coding	51288103	51288302	-	51288202	78	-	Protein cordon-bleu [Source:UniProtKB/Swiss-Prot;Acc:O75128]
ENSG00000106078	COBL	ENST00000457460	protein_coding	51288103	51288302	-	51288202	78	-	Protein cordon-bleu [Source:UniProtKB/Swiss-Prot;Acc:O75128]
ENSG00000106078	COBL	ENST00000430962	protein_coding	51288103	51288302	-	51288202	78	-	Protein cordon-bleu [Source:UniProtKB/Swiss-Prot;Acc:O75128]
ENSG00000185842	DNAH14	ENST00000453375	protein_coding	2,25E+08	2,25E+08	+	2,25E+08	47	+	Dynein heavy chain 14, axonemal (Axonemal beta dynein heavy chain 14)(Ciliary dynein heavy chain 14) [Source:UniProtKB/Swiss-Prot;Acc:Q0VDD8]
ENSG00000174628	IQCK	ENST00000308214	protein_coding	19727778	19727977	+	19727829	51	-	IQ domain-containing protein K [Source:UniProtKB/Swiss-Prot;Acc:Q8N0W5]
ENSG00000174628	IQCK	ENST00000320394	protein_coding	19727778	19727977	+	19727829	51	-	IQ domain-containing protein K [Source:UniProtKB/Swiss-Prot;Acc:Q8N0W5]
ENSG00000164308	LRAP	ENST00000379904	protein_coding	96211644	96211843	+	96211670	26	-	Endoplasmic reticulum aminopeptidase 2 (EC 3.4.11.-)(Leukocyte-derived arginine aminopeptidase)(L-RAP) [Source:UniProtKB/Swiss-Prot;Acc:Q6P179]
ENSG00000164308	LRAP	ENST00000437043	protein_coding	96211644	96211843	+	96211670	26	-	Endoplasmic reticulum aminopeptidase 2 (EC 3.4.11.-)(Leukocyte-derived arginine aminopeptidase)(L-RAP) [Source:UniProtKB/Swiss-Prot;Acc:Q6P179]
ENSG00000164308	LRAP	ENST00000343361	protein_coding	96211644	96211843	+	96211670	26	-	Endoplasmic reticulum aminopeptidase 2 (EC 3.4.11.-)(Leukocyte-derived arginine aminopeptidase)(L-RAP) [Source:UniProtKB/Swiss-Prot;Acc:Q6P179]
ENSG00000164308	LRAP	ENST00000414384	protein_coding	96211644	96211843	+	96211670	26	-	Endoplasmic reticulum aminopeptidase 2 (EC 3.4.11.-)(Leukocyte-derived arginine aminopeptidase)(L-RAP) [Source:UniProtKB/Swiss-Prot;Acc:Q6P179]
ENSG00000100316	RPL3	ENST00000427905	protein_coding	39715401	39715600	-	39715525	53	-	60S ribosomal protein L3 (HIV-1 TAR RNA-binding protein B)(TARBP-B) [Source:UniProtKB/Swiss-Prot;Acc:P39023]
ENSG00000100316	RPL3	ENST00000420536	protein_coding	39715429	39715628	-	39715525	81	-	60S ribosomal protein L3 (HIV-1 TAR RNA-binding protein B)(TARBP-B) [Source:UniProtKB/Swiss-Prot;Acc:P39023]

ENSEMBL55GeneID	GeneSymbol	ENSEMBL55TranscriptID	Biotype	GeneStart	GeneEnd	GeneStrand	Motifstart	TSSoffset	Motifstrand	Description
ENSG00000100316	RPL3	ENST00000437815	protein_coding	39715429	39715628	-	39715525	81	-	60S ribosomal protein L3 (HIV-1 TAR RNA-binding protein B)(TARBP-B) [Source:UniProtKB/Swiss-Prot;Acc:P39023]
ENSG00000100316	RPL3	ENST00000402527	protein_coding	39715429	39715628	-	39715525	81	-	60S ribosomal protein L3 (HIV-1 TAR RNA-binding protein B)(TARBP-B) [Source:UniProtKB/Swiss-Prot;Acc:P39023]
ENSG00000134419	RPS15A	ENST00000322989	protein_coding	18801457	18801656	-	18801578	56	-	40S ribosomal protein S15a [Source:UniProtKB/Swiss-Prot;Acc:P62244]
ENSG00000134419	RPS15A	ENST00000396261	protein_coding	18801457	18801656	-	18801578	56	-	40S ribosomal protein S15a [Source:UniProtKB/Swiss-Prot;Acc:P62244]
ENSG00000138326	RPS24	ENST00000401656	protein_coding	79793623	79793822	+	79793689	66	+	40S ribosomal protein S24 [Source:UniProtKB/Swiss-Prot;Acc:P62847]
ENSG00000189091	SF3B3	ENST00000310750	protein_coding	70557746	70557945	+	70557795	49	+	Splicing factor 3B subunit 3 (Spliceosome-associated protein 130)(SAP 130)(Pre-mRNA-splicing factor SF3b 130 kDa subunit)(SF3b130)(STAF130) [Source:UniProtKB/Swiss-Prot;Acc:Q15393]
ENSG00000189091	SF3B3	ENST00000302516	protein_coding	70557701	70557900	+	70557795	94	+	Splicing factor 3B subunit 3 (Spliceosome-associated protein 130)(SAP 130)(Pre-mRNA-splicing factor SF3b 130 kDa subunit)(SF3b130)(STAF130) [Source:UniProtKB/Swiss-Prot;Acc:Q15393]
ENSG00000088386	SLC15A1	ENST00000313260	protein_coding	99378556	99378755	-	99378668	65	-	Solute carrier family 15 member 1 (Peptide transporter 1)(Oligopeptide transporter, small intestine isoform)(Intestinal H(+)/peptide cotransporter) [Source:UniProtKB/Swiss-Prot;Acc:P46059]
ENSG00000125835	SNRPB	ENST00000303103	protein_coding	2451218	2451417	-	2451349	46	-	Small nuclear ribonucleoprotein-associated proteins B and B' (snRNP-B)(Sm protein B/B')(Sm-B/Sm-B')(SmB/SmB') [Source:UniProtKB/Swiss-Prot;Acc:P14678]
ENSG00000125835	SNRPB	ENST00000339610	protein_coding	2451218	2451417	-	2451349	46	-	Small nuclear ribonucleoprotein-associated proteins B and B' (snRNP-B)(Sm protein B/B')(Sm-B/Sm-B')(SmB/SmB') [Source:UniProtKB/Swiss-Prot;Acc:P14678]
ENSG00000125835	SNRPB	ENST00000336808	protein_coding	2451218	2451417	-	2451349	46	-	Small nuclear ribonucleoprotein-associated proteins B and B' (snRNP-B)(Sm protein B/B')(Sm-B/Sm-B')(SmB/SmB') [Source:UniProtKB/Swiss-Prot;Acc:P14678]
ENSG00000235190	RP1-197O17.3	ENST00000438615	pseudogene	1,31E+08	1,31E+08	+	1,31E+08	91	-	
ENSG00000235018	RP11-11M20.2	ENST00000442115	pseudogene	60612813	60613012	-	60612963	27	-	
ENSG00000222440	U2	ENST00000410508	snRNA	91053092	91053291	+	91053139	47	+	U2 spliceosomal RNA [Source:RFAM;Acc:RF00004]
ENSG00000222536	U2	ENST00000410604	snRNA	72948935	72949134	+	72948986	51	+	U2 spliceosomal RNA [Source:RFAM;Acc:RF00004]
ENSG00000222598	U2	ENST00000410666	snRNA	1,15E+08	1,15E+08	-	1,15E+08	44	-	U2 spliceosomal RNA [Source:RFAM;Acc:RF00004]
ENSG00000222800	U2	ENST00000410868	snRNA	27077560	27077759	-	27077690	47	-	U2 spliceosomal RNA [Source:RFAM;Acc:RF00004]
ENSG00000222810	U2	ENST00000410878	snRNA	71596820	71597019	-	71596950	47	-	U2 spliceosomal RNA [Source:RFAM;Acc:RF00004]
ENSG00000206613	U6	ENST00000383886	snRNA	1,7E+08	1,7E+08	-	1,7E+08	30	+	U6 spliceosomal RNA [Source:RFAM;Acc:RF00026]
