# Supplementary material for: A tandem sequence motif acts as a distance-dependent enhancer in a set of genes involved in translation by binding the proteins NonO and SFPQ
Source: BMC Genomics. 2011 Dec 20;12:624. doi: 10.1186/1471-2164-12-624 (PMC3262029; doi:10.1186/1471-2164-12-624)
Supplement: Additional file 6 — Supplementary Table S5. Result of the Gene Ontology (GO) enrichment analysis The table shows the results of the Gene Ontology (GO) enrichment analysis using Otologizer. The first two columns denote the GO ID and GO name. The third column denotes the absolute number of GO terms as annotated in GO. The fourth and fifth column denote the enrichment p-value, without and with adjustments for multiple testing, respectively. [file 1471-2164-12-624-S6.PDF]

Additional file 6 – Supplementary Table 5. Result of the Gene Ontology (GO) enrichment analysis

The table shows the results of the Gene Ontology (GO) enrichment analysis using Otologizer. The first two columns denote the GO ID and GO name. The third column denotes the absolute number of GO terms as annotated in GO. The fourth and fifth column denote the enrichment p-value, without and with adjustments for multiple testing, respectively.

GO ID	GO category	GO terms in category	p	p.adjusted
GO:0006414	translational.elongation	87	4,97E-17	1,25E-13
GO:0005840	ribosome	169	5,22E-12	6,59E-09
GO:0044445	cytosolic.part	125	2,45E-10	2,06E-07
GO:0006412	translation	337	3,14E-09	1,63E-06
GO:0033279	ribosomal.subunit	106	3,23E-09	1,63E-06
GO:0005198	structural.molecule.activity	518	6,94E-09	2,92E-06
GO:0005829	cytosol	983	2,56E-08	9,23E-06
GO:0003735	structural.constituent.of.the.ribosome	135	7,32E-08	2,31E-05
GO:0030529	ribonucleoprotein.complex	396	1,75E-06	4,91E-04
GO:0043228	non-membrane-bounded.organelle	2096	4,65E-05	0.01
GO:0043232	intracellular.non-membrane-bounded.organelle	2096	4,70E-05	0.01
GO:0022626	cytosolic.ribosome	69	8,88E-05	0.02
GO:0022625	cytosolic.large.ribosomal.subunit	34	2,31E-04	0.04
