# Supplementary material for: A tandem sequence motif acts as a distance-dependent enhancer in a set of genes involved in translation by binding the proteins NonO and SFPQ
Source: BMC Genomics. 2011 Dec 20;12:624. doi: 10.1186/1471-2164-12-624 (PMC3262029; doi:10.1186/1471-2164-12-624)
Supplement: Additional file 7 — Supplementary Figure S2. EMSA experiment with LTSM-positive probes of three genes - complete picture The first lane of each probe contains the labelled doublestranded DNA, in the second lane nuclear extract (NE) was added. Unlabelled specific competitors (SC) were added to block specific protein binding in the third lane. In the last three lanes unspecific competitor (UC) sequence of LTSM-negative RPS6 was added. Specific binding is indicated by black arrows. [file 1471-2164-12-624-S7.PDF]

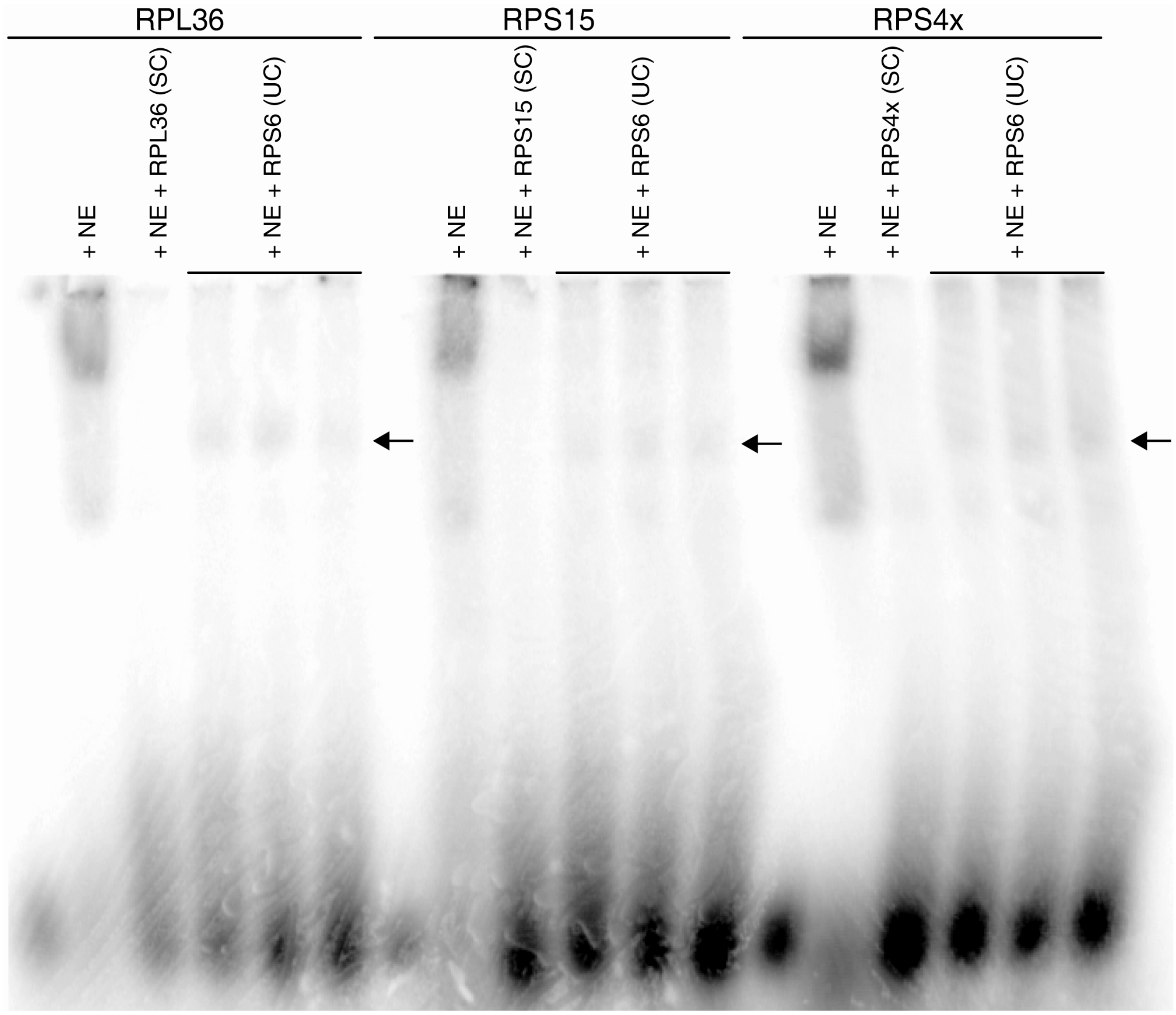

Additional file 7 – Supplementary Figure 2. EMSA experiment with LTSM-positive probes of three genes – complete picture

The first lane of each probe contains the labelled doublestranded DNA, in the second lane nuclear extract (NE) was added. Unlabelled specific competitors (SC) were added to block specific protein binding in the third lane. In the last three lanes unspecific competitor (UC) sequence of LTSM-negative RPS6 was added. Specific binding is indicated by black arrows.
