# Supplementary material for: A tandem sequence motif acts as a distance-dependent enhancer in a set of genes involved in translation by binding the proteins NonO and SFPQ
Source: BMC Genomics. 2011 Dec 20;12:624. doi: 10.1186/1471-2164-12-624 (PMC3262029; doi:10.1186/1471-2164-12-624)
Supplement: Additional file 8 — Supplementary Figure S3. Identification of LTSM-specific binding proteins Pull-down experiment using biotinylated LTSM-positive probes of RPL36, nuclear extract (NE) of SHP77 cells and 1000-fold molar excess of unspecific competitor (UC) sequence of LTSM-negative RPS6. The three dominant bands detected by Coomassie staining (indicated by black arrows) were analyzed using mass spectrometry (n = 2). For identified proteins compare Additional file 9. [file 1471-2164-12-624-S8.PDF]

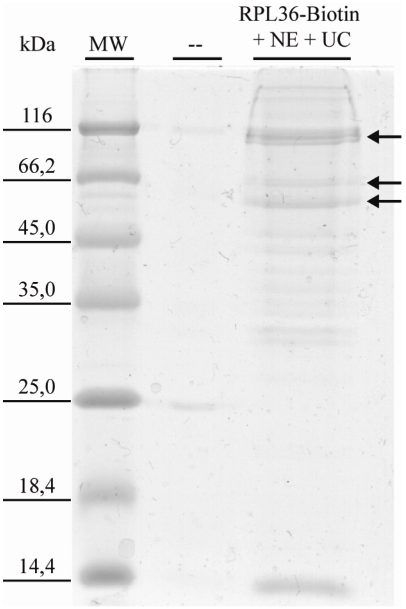

Additional file 8 – Supplementary Figure 3. Identification of LTSM-specific binding proteins

Pull-down experiment using biotinylated LTSM-positive probes of RPL36, nuclear extract (NE) of SHP77 cells and 1000-fold molar excess of unspecific competitor (UC) sequence of LTSM-negative RPS6. The three dominant bands detected by Coomassie staining (indicated by black arrows) were analyzed using mass spectrometry (n = 2). For identified proteins compare **Additional file 9.**
