# Supplementary material for: A tandem sequence motif acts as a distance-dependent enhancer in a set of genes involved in translation by binding the proteins NonO and SFPQ
Source: BMC Genomics. 2011 Dec 20;12:624. doi: 10.1186/1471-2164-12-624 (PMC3262029; doi:10.1186/1471-2164-12-624)
Supplement: Additional file 9 — Supplementary Table S6. List of proteins in LTSM pull-down samples Mass spectrometry (MALDI-TOF) analysis of proteins pulled by biotinylated LTSM-positive probes of RPL36 (compare Additional file 8) and two different unspecific, LTSM-negative competitors (RPS6 and RPL13A). The unspecific competitors (UC) were of the same position as LTSM. H.s: Homo sapiens. [file 1471-2164-12-624-S9.PDF]

**Additional file 9 – Supplementary Table 6. List of proteins in LTSM pull-down samples**

Mass spectrometry (MALDI-TOF) analysis of proteins pulled by biotinylated LTSM-positive probes of RPL36 (compare **Additional file 8**) and two different unspecific, LTSM-negative competitors (RPS6 and RPL13A). The unspecific competitors (UC) were of the same position as LTSM. H.s: *Homo sapiens*.

| Name | Accession | Mass [Da] | Score | Peptides | Protein |
|---|---|---|---|---|---|
| **RPL36 with RPS6 100kDa** | gi\|22902366 | 112.984 | 1191 | 18 | poly(ADP-ribosyl)transferase [H.s] |
| (mixture of 3 proteins) | gi\|29881667 | 76.141 | 698 | 11 | **splicing factor proline/glutamine rich (polypyrimidine tract binding protein associated) [H.s]** |
| | gi\|32358 | 88.890 | 119 | 2 | hnRNP U protein [H.s] |
| **RPL36 with RPS6 65kDa** | gi\|460789 | 51.040 | 173 | 3 | transformation upregulated nuclear protein [H.s] |
| (mixture of 2 proteins) | gi\|28592 | 69.321 | 150 | 3 | serum albumin [H.s] |
| **RPL36 with RPS6 60kDa** | gi\|34932414 | 54.197 | 849 | 16 | **non-POU domain containing, octamer-binding [H.s]** |

| Name | Accession | Mass [Da] | Score | Peptides | Protein |
|---|---|---|---|---|---|
| **RPL36 with RPL13A 100kDa** | gi\|337424 | 113.083 | 587 | 10 | poly(ADP-ribose)-synthetase |
| (mixture of 2 proteins) | gi\|29881667 | 76.141 | 578 | 11 | **splicing factor proline/glutamine rich (polypyrimidine tract binding protein associated) [H.s]** |
| **RPL36 with RPL13A 65kDa** | gi\|28592 | 69.321 | 322 | 5 | serum albumin [H.s] |
| (mixture of 3 proteins) | gi\|226021 | 66.881 | 152 | 3 | growth regulated nuclear 68 protein |
| | gi\|5730027 | 48.197 | 81 | 2 | KH domain containing, RNA binding, signal transduction associated 1 [H.s] |
| **RPS36 with RPL13A 60kDa** | gi\|34932414 | 54.197 | 558 | 11 | **non-POU domain containing, octamer-binding [H.s]** |
| (mixture of 2 proteins) | gi\|190167 | 113.011 | 88 | 2 | poly(ADP-ribose)-polymerase |
| **RPL36 with RPL13A 35kDa** | gi\|356168 | 21.721 | 182 | 3 | histone H1b |
| (mixture of 2 proteins) | gi\|4885381 | 22.566 | 116 | 2 | H1 histone family, member 5 [H.s] |
