# Supplementary material for: A tandem sequence motif acts as a distance-dependent enhancer in a set of genes involved in translation by binding the proteins NonO and SFPQ
Source: BMC Genomics. 2011 Dec 20;12:624. doi: 10.1186/1471-2164-12-624 (PMC3262029; doi:10.1186/1471-2164-12-624)
Supplement: Additional file 10 — Supplementary Figure S6. NonO and SFPQ bind to tripartite LTSMs Pull-down assays with subsequent Western blotting analysis using biotinylated DNA oligonucleotides containing the tripartite LTSMs of the three RP genes RPS15A, RPL17 and RPL24. Unspecific competitor (UC) sequence of LTSM-negative derived from RPS6 was applied in 1000-fold molecular excess. Non-biotinylated LTSM-positive RPL36 probes were used as control. Specific protein signals are indicated by black arrowheads. Proteins were detected using both specific antibodies in conjunction. [file 1471-2164-12-624-S10.PDF]

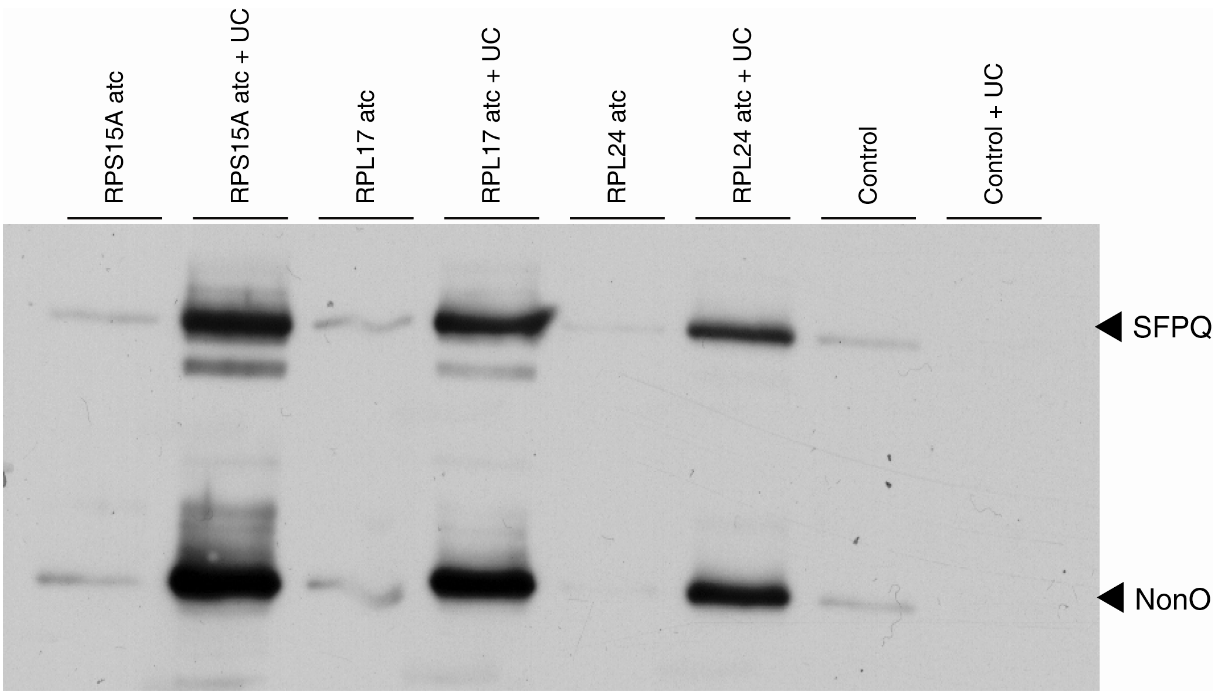

Additional file 10 – Supplementary Figure 6. NonO and SFPQ bind to tripartite LTSMs
Pull-down assays with subsequent Western blotting analysis using biotinylated DNA oligonucleotides containing the tripartite LTSMs of the three RP genes RPS15A, RPL17 and RPL24. Unspecific competitor (UC) sequence of LTSM-negative derived from RPS6 was applied in 1000-fold molecular excess. Non-biotinylated LTSM-positive RPL36 probes were used as control. Specific protein signals are indicated by black arrowheads. Proteins were detected using both specific antibodies in conjunction.
