# Supplementary material for: A tandem sequence motif acts as a distance-dependent enhancer in a set of genes involved in translation by binding the proteins NonO and SFPQ
Source: BMC Genomics. 2011 Dec 20;12:624. doi: 10.1186/1471-2164-12-624 (PMC3262029; doi:10.1186/1471-2164-12-624)
Supplement: Additional file 11 — Supplementary Figure S4. Influence of NonO and SFPQ on RPL12 promotor activity using luciferase assays (A) Schematic view of a part of the human RPL12 gene indicating the amplified region. (B,C) Dual Luciferase vector psiCHECK2 with the original SV40 promotor and vector psiRPL12 with the original promotor replaced by the proximal promoter region and part of exon 1 of RPL12. (D) Cotransfection of psiRPL12 and eukaryotic expression vectors containing the genes of NonO and SFPQ (pTarget-NonO and pTarget-SFPQ) did not result in a significantly enhanced promoter activity. GFP expression vector alone served as a control. [file 1471-2164-12-624-S11.PDF]

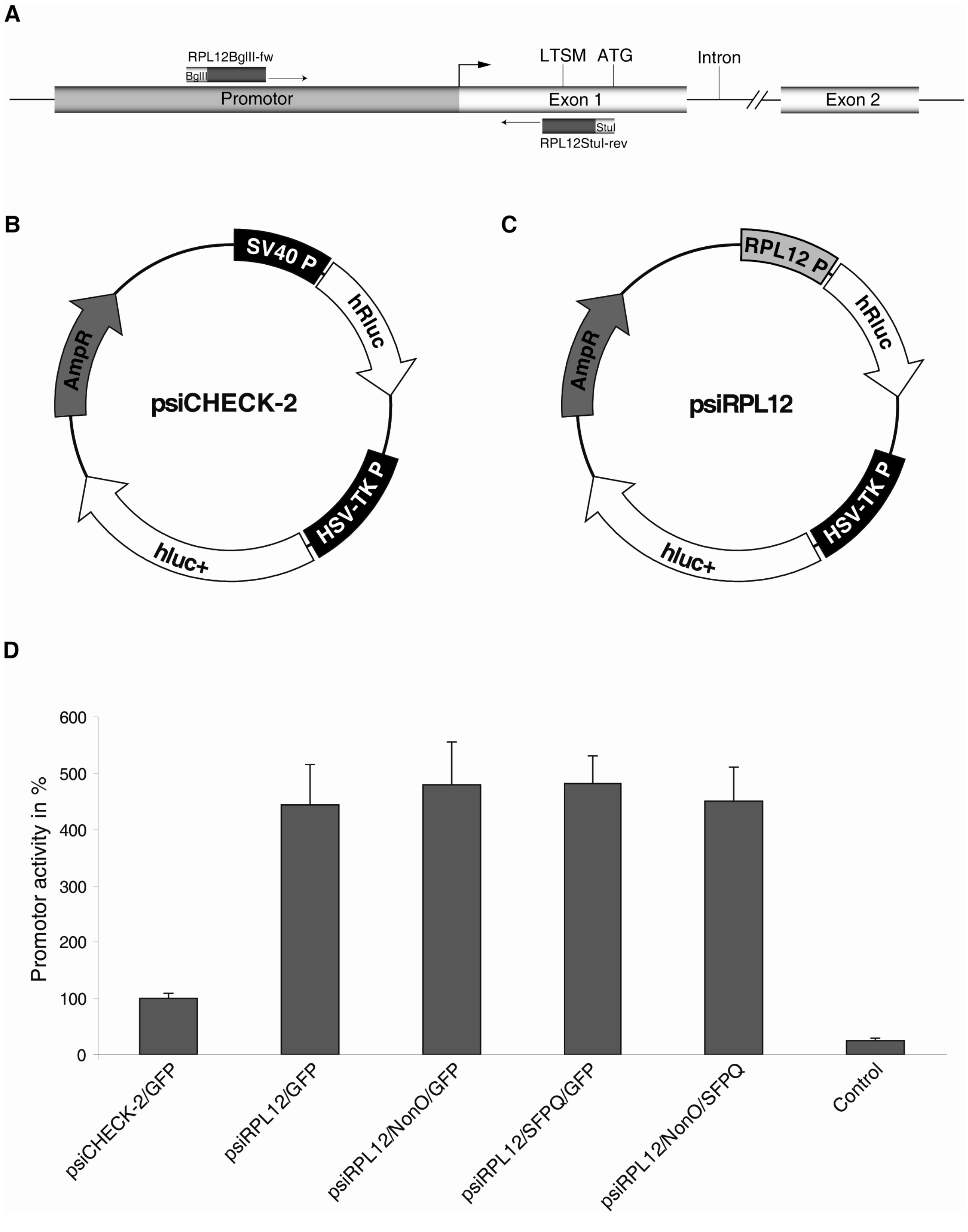

Additional file 11 – Supplementary Figure 4. Influence of NonO and SFPQ on RPL12 promotor activity using luciferase assays

(A) Schematic view of a part of the human RPL12 gene indicating the amplified region. (B,C) Dual Luciferase vector psiCHECK2 with the original SV40 promotor and vector psiRPL12 with the original promotor replaced by the proximal promoter region and part of exon 1 of RPL12. (D) Cotransfection of psiRPL12 and eukaryotic expression vectors containing the genes of NonO and SFPQ (pTarget-NonO and pTarget-SFPQ) did not result in a significantly enhanced promoter activity. GFP expression vector alone served as a control.
