# Supplementary material for: A tandem sequence motif acts as a distance-dependent enhancer in a set of genes involved in translation by binding the proteins NonO and SFPQ
Source: BMC Genomics. 2011 Dec 20;12:624. doi: 10.1186/1471-2164-12-624 (PMC3262029; doi:10.1186/1471-2164-12-624)
Supplement: Additional file 12 — Supplementary Figure S5. Distance of LTSM to TSS determines the efficiency of transcription Complete picture of Northern blotting experiment detecting β- Galactosidase transcripts via β-Galactosidase specific DNA probe at 5'-end of the transcript (Refer to Figure 5B). [file 1471-2164-12-624-S12.PDF]

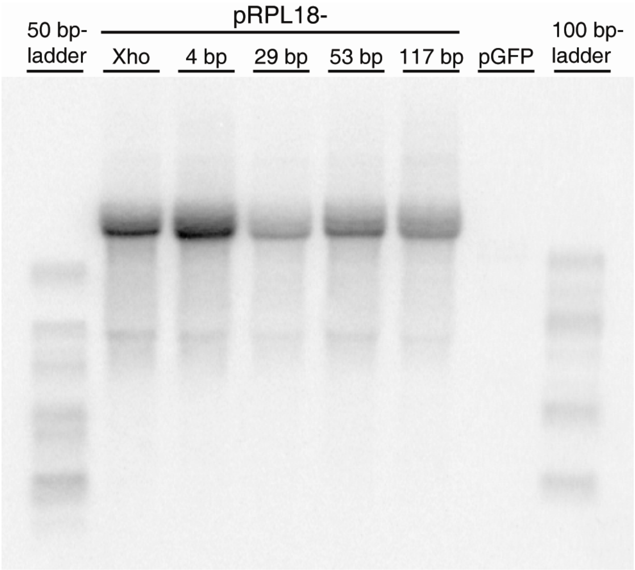

Additional file 12 – Supplementary Figure 5. Distance of LTSM to TSS determines the efficiency of transcription

Complete picture of Northern blotting experiment detecting β- Galactosidase transcripts via β-Galactosidase specific DNA probe at 5'-end of the transcript (Refer to Figure 5B).
