# Supplementary material for: A tandem sequence motif acts as a distance-dependent enhancer in a set of genes involved in translation by binding the proteins NonO and SFPQ
Source: BMC Genomics. 2011 Dec 20;12:624. doi: 10.1186/1471-2164-12-624 (PMC3262029; doi:10.1186/1471-2164-12-624)
Supplement: Additional file 13 — Supplementary Table S7. Sequences of single stranded DNA oligonucleotides used in EMSA experiments The LTSMs are highlighted in grey on the forward strand. [file 1471-2164-12-624-S13.PDF]

Additional file 13 – Supplementary Table 7. Sequences of single stranded DNA oligonucleotides used in EMSA experiments

The LTSMs are highlighted in grey on the forward strand.

RPL36-fw	5’-CTG CCG GTA TCC GCC GCC ATC CGG A-3’
RPL36-rev	5’-GGG AGT CCG GAT GGC GGC GGA TAC C-3’
RPL32-fw	5’-GAA GTG TTA ATC CGT GGC AAT CCG CA-3’
RPL32-rev	5’-AGG AAT GCG GAT TGC CAC GGA TTA A-3’
RPL18-fw	5’-GCT GGA TAA TCC GCT GCC ATC CGC C-3’
RPL18-rev	5’-GGA CCA CGG CGG ATG GCA GCG GAT TAT-3’
RPL12-fw	5’-GAA TCC GGG TTC ATC CGA CAC CAG-3’
RPL12-rev	5’-GTG TCG GAT GAA CCC GGA TTC GGG ACG-3’
RPL11-fw	5’-GGC TTT CCT TTA TCC GTC GCC ATC CAT-3’
RPL11-rev	5’-GGC TGC CAT GGA TGG CGA CGG ATA AAG-3’
RPL7a-fw	5’-GGC TGT ATC CGC TGC CAT CCT CCT CC-3’
RPL7a-rev	5’-GCG CCT GGA GGA GGA TGG CAG CGG ATA-3’
RPS15-fw	5’-GGG CCT ATC CGG CTC CAT CCA ACC TCT-3’
RPS15-rev	5’-CGG TCA GAG GTT GGA TGG AGC CGG ATA-3’
RPS4x-fw	5’-GGA CGT ATC CGC CTC CAT CCT CCC C-3’
RPS4x-rev	5’-GGC CGG GGG AGG ATG GAG GCG GAT A-3’
RPS6-fw	5’-GGT AGG TGA TGG TGG CGA GTG TTA G-3’
RPS6-rev	5’-CCA GTC TAA CAC TCG CCA CCA TCA C-3’
RPL13A-fw	5’-CCG CGC GGG CCG GGG CGG CAA GGG G-3’
RPL13A-rev	5’-CCC GGC CCC TTG CCG CCC CGG CCC G-3’
