# Supplementary material for: A tandem sequence motif acts as a distance-dependent enhancer in a set of genes involved in translation by binding the proteins NonO and SFPQ
Source: BMC Genomics. 2011 Dec 20;12:624. doi: 10.1186/1471-2164-12-624 (PMC3262029; doi:10.1186/1471-2164-12-624)
Supplement: Additional file 14 — Supplementary Table S8. Sequences of biotinylated DNA oligonucleotides used in pull down experiments The LTSMs are highlighted in grey on the forward strand. [file 1471-2164-12-624-S14.PDF]

Additional file 14 – Supplementary Table 8. Sequences of biotinylated DNA oligonucleotides used in pull down experiments

The LTSMs are highlighted in grey on the forward strand.

RPL36-fw-Bio	5’-GGT ATC CGC CGC CAT CCG GAC TCC C-3’
RPL36-rev-Bio	5’-Biotin-GGG AGT CCG GAT GGC GGC GGA TAC C-3’
RPL18-fw-Bio	5’-GAT AAT CCG CTG CAA TCC GCC GTG G-3’
RPL18-rev-Bio	5’-Biotin-CCA CGG CGG ATT GCA GCG GAT TAT C -3’
RPL12-fw-Bio	5’-CCG AAT CCG GGT TCA TCC GAC ACC A-3’
RPL12-rev-Bio	5’-Biotin-TGG TGT CGG ATG AAC CCG GAT TCG G -3’
RPS15-fw-Bio	5’-GCC TAT CCG GCT CCA TCC AAC CTC T-3’
RPS15-rev-Bio	5’-Biotin-AGA GGT TGG ATG GAG CCG GAT AGG C-3’
RPS4x-fw-Bio	5’-ACG TAT CCG CCT CCA TCC TCC CCC G-3’
RPS4x-rev-Bio	5’-Biotin-CGG GGG AGG ATG GAG GCG GAT ACG T-3’
RPS24_atc-fw-Bio	5’-GTC ATC TGC CGC GTA TCC GAG CCA TCC GTG GTC CC-3’
RPS24_atc-rev-Bio	5’-Biotin-GGG ACC ACG GAT GGC TCG GAT ACG CGG CAG ATG AC-3’
RPS15A_atc-fw-Bio	5’-TTC ATC CGT CTG CCA TCG GCG CCA TCC TGC AAT CTA-3’
RPS15A_atc-rev-Bio	5’-Biotin-TAG ATT GCA GGA TGG CGC CGA TGG CAG ACG GAT GAA-3’
RPL17-atc-fw-Bio	5’-CCA ATC CTC CTG CCA TCG CCG CCA TCC TGG CTT CGG-3’
RPL17-atc-rev-Bio	5’-Biotin-CCG AAG CCA GGA TGG CGC CAA TGG CAG GAG GAT TGG-3’
