# Supplementary material for: A tandem sequence motif acts as a distance-dependent enhancer in a set of genes involved in translation by binding the proteins NonO and SFPQ
Source: BMC Genomics. 2011 Dec 20;12:624. doi: 10.1186/1471-2164-12-624 (PMC3262029; doi:10.1186/1471-2164-12-624)
Supplement: Additional file 15 — Supplementary Table S9. Sequences of employed PCR primers and DNA linker oligonucleotides [file 1471-2164-12-624-S15.PDF]

Additional file 15 – Supplementary Table 9. Sequences of employed PCR primers and DNA linker oligonucleotides

RPL12BglII-fw	5'-AGA TCT CAT AAA GTC ACG CAG ACC GG-3'
RPL12StuI-rev	5'-AGG CCT GGT GGA GGC GGC TGG TG-3'
NONO	5'-GTT GAG GGT GCA AAA ATG C-3'
NONOas	5'-ATT CTG GCT AGT CCA AAA AGG-3'
SFPQ_Xho-fw	5'-CTG GAG GAG CGT CTT CTT CGC TTT TGC CTC-3'
SFPQ_AatII-rev	5'-GAC GTC CTG CCC AAA CAG ACC ATT TAC-3'
GEO1	5'-GAA GGG GCC TTG ATG GAA GG-3'
GEO2	5'-TTT GTT AGG GTG TCT TCT TCT TTG G-3'
GEO3	5'-AGA AGA AGA CAC CCT AAC AAA GAG G -3'
GEO4	5'-CGT GCA TCT GCC AGT TTG AGG-3'
RPL18_HD	5'-AAG CTT AAA TAT TGA CCT CAG CAG TTT TAT TAA C-3'
RPL18_XH2	5'-AAG CTT CCA CGG CGG ATG GCA GCG GAT TAT CCA CTC GAG AGC GCC GCA AAG CGA GCT CAC CAT GAT GGC-3'
Geo-link 25-fw	5'-TCG AGA TAT CAG GAT GCC ATT GCA ACG TA-3'
Geo-link 25-rev	5'-TCG ATA CGT TGC AAT GGC ATC CTG ATA TC-3'
Geo-link 50-fw	5'-TCG AGA TAT CAG GAT GCC ATT GCA ACG TAC GAA TCA GGA TGC CAT TGC AAC ATC-3'
Geo-link 50-rev	5'-TCG AGA TGT TGC AAT GGC ATC CTG ATT CGT ACG TTG CAA TGG CAT CCT GAT ATC-3'
Geo-link 75-fw	5'-TCG AGA TAT CAG GAT GCC ATT GCA ACG TAC GAA TCA GGA TGC CAT TGC AAC GTA CGA ATC AGG ATG CCA TTG CAA CAC C-3'
Geo-link 75-rev	5'-TCG AGG TGT TGC AAT GGC ATC CTG ATT CGT ACG TTG CAA TGG CAT CCT GAT TCG TAC GTT GCA ATG GCA TCC TGA TAT C-3'
