# Supplementary material for: A tandem sequence motif acts as a distance-dependent enhancer in a set of genes involved in translation by binding the proteins NonO and SFPQ
Source: BMC Genomics. 2011 Dec 20;12:624. doi: 10.1186/1471-2164-12-624 (PMC3262029; doi:10.1186/1471-2164-12-624)
Supplement: Additional file 16 — Supplementary Figure S7. Sequence verification of inserted linkers Sequences obtained by sequencing the described RPL18 region in pGEM-T Easy vector using primer M13 rev (-49). Please, refer to Figure 5 in the main text. Translation start (ATG) is shown in bold letters, inserted Xho I restriction site with insertion of linkers of different lengths (bold letters) is highlighted in dark grey and the LTSM is highlighted in light grey. [file 1471-2164-12-624-S16.PDF]

> pGEM-RPL18-Xho

CCCATTTTCCCCCCCCCACCCCAGCACGGAGTCTCCATTTTGCCTCCCCTATGAAACTCTGTCTCCCTCTTTCTA
GGTCAGGGAAGGGCCATCTAGGGGCCGGGGGAGAAGGAGAAGCGGAACTCAAAGGATGGGAATACAGGATGCTAT
AGATTGAGGTGCAGCAAGATCATCTCGGGTTAGACCTTAAGTAGGACTTCCCAAGCAGCGGCTGCGCCTACGAAG
TGCAGCGCGAGATCAGGGAGCGGTGAACCTGATAAGGAAACGCCCGAAGAGTGGGGTCGGAAATTTCCAGATGCC
CCGACTAGCCCTGCCAGCCCCGCCTCCTCACCTCCAACACTGGGCCCGCCGGGTCCTCCGGGAGCCACTGAGAGG
CCGTCGGCGTCGGGCCAGAGCGTCACCGCGCGACGCATCGTGCTAGCTACACGCATCTCGCTCCCAGATCCCTTT
CTTCCGGAAAGCTATCGAGAACACGGCCTGGGTAGGGCCAGAGAGGCCCCCGACGTGCTGGCCCTTCCCTCTTGG
ACGTTGCGCTTGTTCCTGCGCTCTATGCTCTCTGCCGTTATCGCCCGGCTAGTCAGTCGTCCAACTCACCACAGA
GAAGTCCGGATCGTGGTAGAGCGCCGCGTCGCACCCATGTGACGTCACGGCGGCGCCACTCGCTTGAGGCTTTCC
CCGCCCACCCCAGCCCGTTCTCTCTTTCCGGACCTGGCCGAGCAGGAGGCGCCATC**ATG**GTGAGCTCGCTTTGCG
GCGCTCTCGAGTGGATAATCCGCTGCCATCCGCCGTGGAAGCTTAAATATTGACCTCAGCAGTTTTATTAAGCCA
TCATGGTGAGCTCGCTTTGCGGCGCTGAATCACTAGTGAATTCGCGGCCGCCTGCAGGTCGACCATATGGGAGAG
CTCCCAACGCGTTGGATGCATAGCTTGAGTATTCTATAGTGTCACCTAAATAGCTGGCGTAATCATGCGGCGTCG
GGCCAGAGCGTCACCGCGCGACGCATCGTGCTAGCTACACGCATCTCGCTCCCAGATCCCTTTCTTCCGGAAAGC
TATCGAGAACACGGCCTGGGTAGGGCCAGAGAGGCCCCCGACGTGCTGGCCCTTCCCTCTTGGACGTTGCGCTTG
TTCCTGCGCTCTATGCTCTCTGCCGTTATCGCCCGGCTAGTCAGTCGTCCAACTCACCACAGAGAAGTCCGGATC
GTGGTAGAGCGCCGCGTCGCACCCATGTGACGTCACGGCGGCGCCACTCGCTTGAGGCTTTCCCCGCCCACCCCA
GCCCGTTCTCTCTTTCCGGACCTGGCCGAGCAGGAGGCGCCATCATGGTGAGCTCGCTTTGGCGGCGCTCTCGAG
TGGATAATCCGCTTGCCATCCGCCGTGGAAGCTTGGAATTCATGGGAAGAGGAACCGAAAGTATGTTTTTCAGAT
GTTCTTTCTCAGAAATAGGAGTTTGCGGAGGTTGGAGTGTGTGTTGTAGGACACGAACCCCAGGGTGGAGGAGAC
TGGAGGA

> pGem-RPL18-4 bp

TTCCATTTCCCCCCCCCACCCCAAGCCACGGAGTCTCCATTTTGCCTCCCCTATGAAACTCTGTCTCCCTCTTTC
TAGGTCAGGGAAGGGCCATCTAGGGGCCGGGGGAGAAGGAGAAGCGGAACTCAAAGGATGGGAATACAGGATGCT
ATAGATTGAGGTGCAGCAAGATCATCTCGGGTTAGACCTTAAGTAGGACTTCCCAAGCAGCGGCTGCGCCTACGA
AGTGCAGCGCGAGATCAGGGAGCGGTGAACCTGATAAGGAAACGCCCGAAGAGTGGGGTCGGAAATTTCCAGATG
CCCCGACTAGCCCTGCCAGCCCCGCCTCCTCACCTCCAACACTGGGCCCGCCGGGTCCTCCGGGAGCCACTGAGA
GGCCGTCGGCGTCGGGCCAGAGCGTCACCGCGCGACGCATCGTGCTAGCTACACGCATCTCGCTCCCAGATCCCT
TTCTTCCGGAAAGCTATCGAGAACACGGCCTGGGTAGGGCCAGAGAGGCCCCCGACGTGCTGGCCCTTCCCTCTT
GGACGTTGCGCTTGTTCCTGCGCTCTATGCTCTCTGCCGTTATCGCCCGGCTAGTCAGTCGTCCAACTCACCACA
GAGAAGTCCGGATCGTGGTAGAGCGCCGCGTCGCACCCATGTGACGTCACGGCGGCGCCACTCGCTTGAGGCTTT
CCCCGCCCACCCCAGCCCGTTCTCTCTTTCCGGACCTGGCCGAGCAGGAGGCGCCATC**ATG**GTGAGCTCGCTTTG
CGGCGCTCTC**GATC**GAGTGGATAATCCGCTGCCATCCGCCGTGGAAGCTTAAATATTGACCTCAGCAGTTTTATT
AAGCCATCATGGTGAGCTCGCTTTGCGGCGCTGAATCACTAGTGAATTCGCGGCCGCCTGCAGGTCGACCATATG
GGAGAGCTCCCAACGCGTTGGATGCATAGCTTGAGTATTCTATAGTGTCACCTAAATAGCTGGCGTAATCATG

> pGem-RPL18-29 bp

AACCAGCACGGAGTCTCCATTTGCCTCCCCTATGAAACTCTGTCTCCCTCTTTCTAGGTCAGGGAAGGGCCATCT
AGGGGCCGGGGGAGAAGGAGAAGCGGAACTCAAAGGATGGGAATACAGGATGCTATAGATTGAGGTGCAGCAAGA
TCATCTCGGGTTAGACCTTAAGTAGGACTTCCCAAGCAGCGGCTGCGCCTACGAAGTGCAGCGCGAGATCAGGGA
GCGGTGAACCTGATAAGGAAACGCCCGAAGAGTGGGGTCGGAAATTTCCAGATGCCCCGACTAGCCCTGCCAGCC
CCGCCTCCTCACCTCCAACACTGGGCCCGCCGGGTCCTCCGGGAGCCACTGAGAGGCCGTCGGCGTCGGGCCAGA
GCGTCACCGCGCGACGCATCGTGCTAGCTACACGCATCTCGCTCCCAGATCCCTTTCTTCCGGAAAGCTATCGAG
AACACGGCCTGGGTAGGGCCAGAGAGGCCCCCGACGTGCTGGCCCTTCCCTCTTGGACGTTGCGCTTGTTCCTGC
GCTCTATGCTCTCTGCCGTTATCGCCCGGCTAGTCAGTCGTCCAACTCACCACAGAGAAGTCCGGATCGTGGTAG
AGCGCCGCGTCGCACCCATGTGACGTCACGGCGGCGCCACTCGCTTGAGGCTTTCCCCGCCCACCCCAGCCCGTT
CTCTCTTTCCGGACCTGGCCGAGCAGGAGGCGCCATC**ATG**GTGAGCTCGCTTTGCGGCGCTCTC**GATACGTTGCA**
**ATGGCATCCTGATATCTC**GAGTGGATAATCCGCTGCCATCCGCCGTGGAAGCTTAAATATTGACCTCAGCAGTTT
TATTAAGCCATCATGGTGAGCTCGCTTTGCGGCGCTGAATCACTAGTGAATTCGCGGCCGCCTGCAGGTCGACCA
TATGGGAGAGCTCCCAACGCGTTGGATGCATAGCTTGAGTATTCTATAGTGTCACCTAAATAGCTGGCGTAATCA
TG

> pGem-RPL18-53 bp

TCCATTTGCCTCCCCTATGAAACTCTGTCTCCCTCTTTCTAGGTCAGGGAAGGGCCATCTAGGGGCCGGGGGAGA
AGGAGAAGCGGAACTCAAAGGATGGGAATACAGGATGCTATAGATTGAGGTGCAGCAAGATCATCTCGGGTTAGA
CCTTAAGTAGGACTTCCCAAGCAGCGGCTGCGCCTACGAAGTGCAGCGCGAGATCAGGGAGCGGTGAACCTGATA
AGGAAACGCCCGAAGAGTGGGGTCGGAAATTTCCAGATGCCCCGACTAGCCCTGCCAGCCCCGCCTCCTCACCTC
CAACACTGGGCCCGCCGGGTCCTCCGGGAGCCACTGAGAGGCCGTCGGCGTCGGGCCAGAGCGTCACCGCGCGAC
GCATCGTGCTAGCTACACGCATCTCGCTCCCAGATCCCTTTCTTCCGGAAAGCTATCGAGAACACGGCCTGGGTA
GGGCCAGAGAGGCCCCCGACGTGCTGGCCCTTCCCTCTTGGACGTTGCGCTTGTTCCTGCGCTCTATGCTCTCTG
CCGTTATCGCCCGGCTAGTCAGTCGTCCAACTCACCACAGAGAAGTCCGGATCGTGGTAGAGCGCCGCGTCGCAC
CCATGTGACGTCACGGCGGCGCCACTCGCTTGAGGCTTTCCCCGCCCACCCCAGCCCGTTCTCTCTTTCCGGACC
TGGCCGAGCAGGAGGCGCCATC**ATG**GTGAGCTCGCTTTGCGGCATCCTGATATCTC**GAGATGTTGCAATGGCATC**
**CTGATTCGTACGTTGCAATGGCATCCTGATACTC**GAGTGGATAATCCGCTGCCATCCGCCGTGGAAGCTTAAATA
TTGACCTCAGCAGTTTTATTAAGCCATCATGGTGAGCTCGCTTTGCGGCGCTGAATCACTAGTGAATTCGCGGCC
GCCTGCAGGTCGACCATATGGGAGAGCTCCCAACGCGTTGGATGCATAGCTTGAGTATTCTATAGTGTCACCTAA
ATAGCTGGCGTAATC

> pGem-RPL18-117 bp

TGAAACTCTGTCTCCCTCTTTCTAGGTCAGGGAAGGGCCATCTAGGGGCCGGGGGAGAAGGAGAAGCGGAACTCA
AAGGATGGGAATACAGGATGCTATAGATTGAGGTGCAGCAAGATCATCTCGGGTTAGACCTTTAAGTAGGACTTC
CCAAGCAGCGGCTGCGCCTACGAAGTGCAGCGCGAGATCAGGGAGCGGTGAACCTGATAAGGAAACGCCCGAAGA
GTGGGGTCGGAAATTTCCAGATGCCCCGACTAGCCCTGCCAGCCCCGCCTCCTCACCTCCAACACTGGGCCCGCC
GGGTCCTCCGGGAGCCACTGAGAGGCCGTCGGCGTCGGGCCAGAGCGTCACCGCGCGACGCATCGTGCTAGCTAC
ACGCATCTCGCTCCCAGATCCCTTTCTTCCGGAAAGCTATCGAGAACACGGCCTGGGTAGGGCCAGAGAGGCCCC
CGACGTGCTGGCCCTTCCCTCTTGGACGTTGCGCTTGTTCCTGCGCTCTATGCTCTCTGCCGTTATCGCCCGGCT
AGTCAGTCGTCCAACTCACCACAGAGAAGTCCGGATCGTGGTAGAGCGCCGCGTCGCACCCATGTGACGTCACGG
CGGCGCCACTCGCTTGAGGCTTTCCCCGCCCACCCCAGCCCGTTCTCTCTTTCCGGACCTGGCCGAGCAGGAGGC
GCCATC**ATG**GTGAGCTCGCTTTGCGGCGTTGCAATGGCAT**CCTGATTCGTACGTTGGCAATGGCATCCTGATATC**
**TCGAGGTGTTGCAATGGCATCCTGATTCGTACGTTGCAATGGCATCCTGATTCGTACGTTGCAATGGCATCCTGG**
**ATATCTC**GAGTGGATAATCCGCTGCCATCCGCCGTGGAAGCTTAAATATTGACCTCAGCAGTTTTATTAAGCCAT
CATGGTGAGCTCGCTTTGCGGCGCTGAATCACTAGTGAATTCGCGGCCGCCTGCAGGTCGACCATATGGGAGAGC
TCCCAACGCGTTGGATGCATAGCTTGAGTATTCTATAGTGTCACCTAAATAGCTGGCGTAATCAT

**Additional file 16 – Supplementary Figure 7. Sequence verification of inserted linkers**

Sequences obtained by sequencing the described RPL18 region in pGEM-T Easy vector using primer M13 rev (-49). Please, refer to Figure 5 in the main text. Translation start (ATG) is shown in bold letters, inserted *Xho* I restriction site with insertion of linkers of different lengths (bold letters) is highlighted in dark grey and the LTSM is highlighted in light grey.
