# Supplementary material for: A tandem sequence motif acts as a distance-dependent enhancer in a set of genes involved in translation by binding the proteins NonO and SFPQ
Source: BMC Genomics. 2011 Dec 20;12:624. doi: 10.1186/1471-2164-12-624 (PMC3262029; doi:10.1186/1471-2164-12-624)
Supplement: Additional file 1 — Supplementary Figure S1. Proximal promoters of 51 zebrafish ribosomal protein genes Annotations of the promoter sequences (range: -80 to +100 around the TSS) with sequence motifs: LTSM (cyan underlined), CT-stretch (red box), YY1 (blue box), and TATA (orange box). Exons are marked by a grey background and the ATG translation start codon by an orange background. All sequences are aligned to their predicted TSS. Pink, yellow and green boxes mark G/T-rich, A/G-rich and A/T-rich sequence motifs of low specificity, respectively (identified automatically by motif search). [file 1471-2164-12-624-S1.PDF]

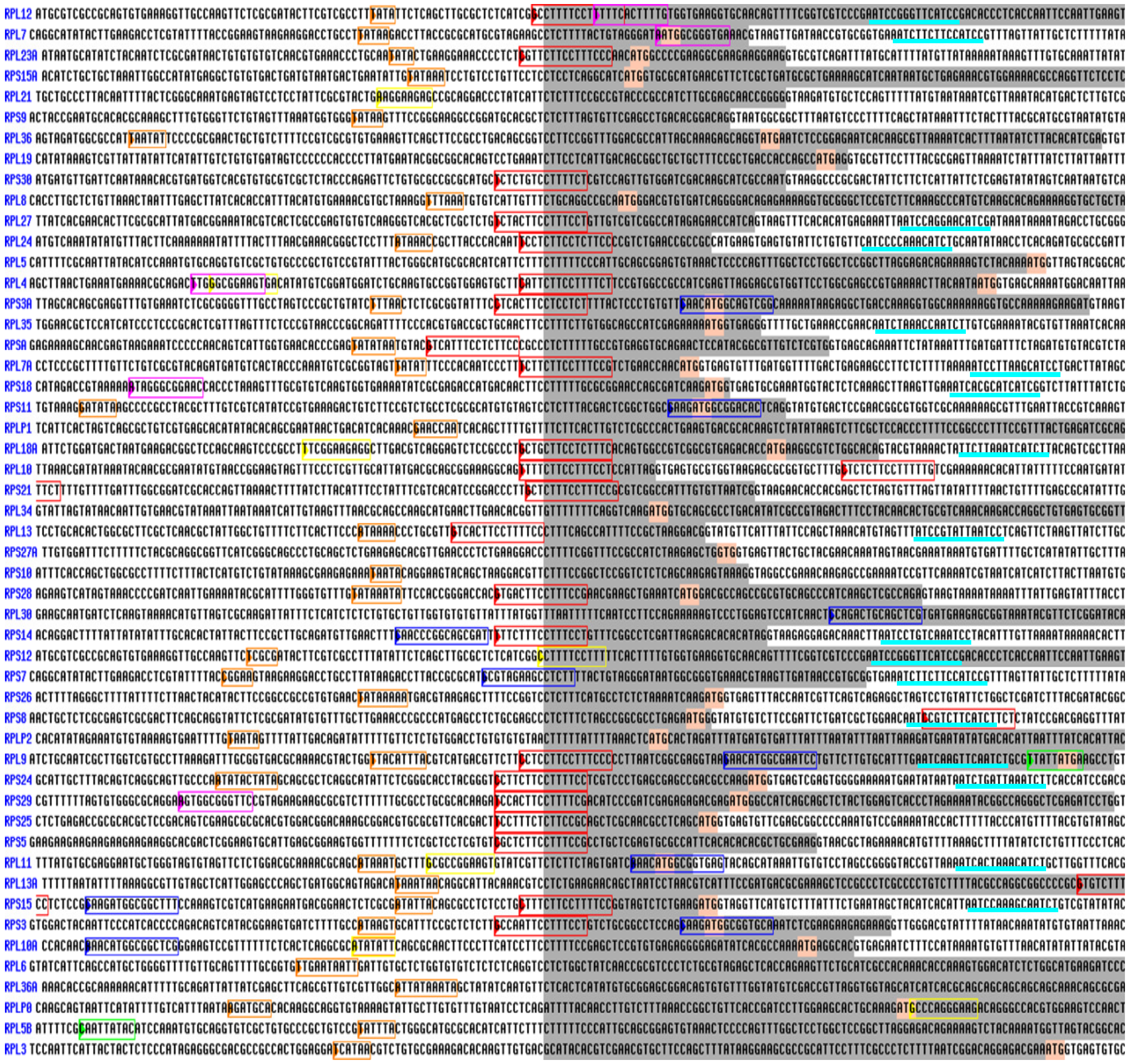

Additional file 1 – Supplementary Figure 1. Proximal promoters of 51 zebrafish ribosomal protein genes

Annotations of the promoter sequences (range: -80 to +100 around the TSS) with sequence motifs: LTSM (cyan underlined), CT-stretch (red box), YY1 (blue box), and TATA (orange box). Exons are marked by a grey background and the ATG translation start codon by an orange background. All sequences are aligned to their predicted TSS. Pink, yellow and green boxes mark G/T-rich, A/G-rich and A/T-rich sequence motifs of low specificity, respectively (identified automatically by motif search).
